# Collective synthesis of aspulvinone and its analogues by vinylogous aldol condensation of substituted tetronic acids with aldehydes†

**DOI:** 10.1039/d2ra08133d

**Published:** 2023-02-07

**Authors:** Xiaotan Yu, Xiaoxia Gu, Yunpeng Zhao, Fengqing Wang, Weiguang Sun, Changxing Qi, Lianghu Gu, Yonghui Zhang

**Affiliations:** a Hubei Key Laboratory of Natural Medicinal Chemistry and Resource Evaluation, School of Pharmacy, Tongji Medical College, Huazhong University of Science and Technology Wuhan 430030 People's Republic of China zhangyh@mails.tjmu.edu.cn gulianghu@hust.edu.cn qichangxing@hust.edu.cn

## Abstract

A mild, modular and efficient synthetic method with broad substrate scope was developed for aspulvinones. Nine natural aspulvinones were synthesized, six of which were for the first time. The aldol condensation delivered *Z*-configuration products predominantly in MeCN. Meanwhile, alkoxy exchange occurred in MeOH and EtOH. Aspulvinone O and E, and substrate 49, 50, and 51 exhibited modest anti-SARS-CoV-2 activity in a high-throughput screening and enzyme kinetics assay.

## Abstract 2

A mild, modular and efficient synthetic method with broad substrate scope was developed for aspulvinones. Some of them exhibited anti-SARS-CoV-2 activity in a high-throughput screening and enzyme kinetics assay.

## Graphical abstract

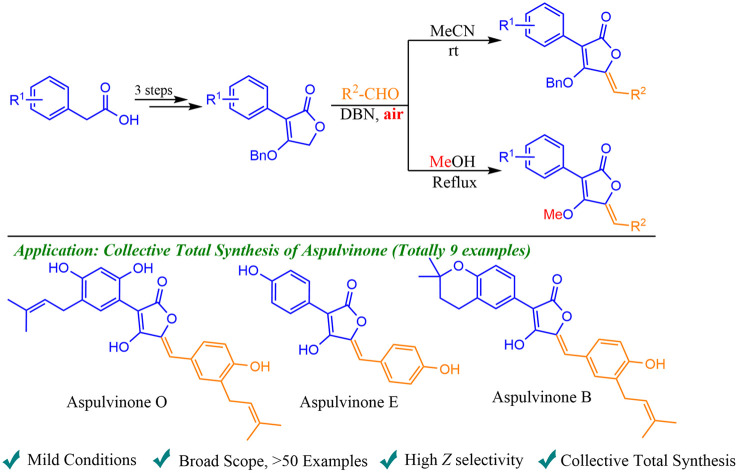

## Introduction

Aspulvinones, mainly isolated from fungus metabolites, were first reported in the 19th century.^1^ Up to now, more than 40 aspulvinones have been reported. The core structure of aspulvinone is characterized by a tetronic acid ring, a 5-membered heterocycle connecting two substituted aromatic rings. Aspulvinones exhibit a wide range of bioactivities, such as antibacterial,^2^ anti-inflammatory,^3^ anti-DPPH radical,^4^ anti-fungal,^5^ and anti-α-glucosidase effects.^6^ Recently, a study showed that some aspulvinones could inhibit SARS-CoV-2 infection and reduce inflammatory reactions caused by SARS-CoV-2.^7^

**Scheme 1:**
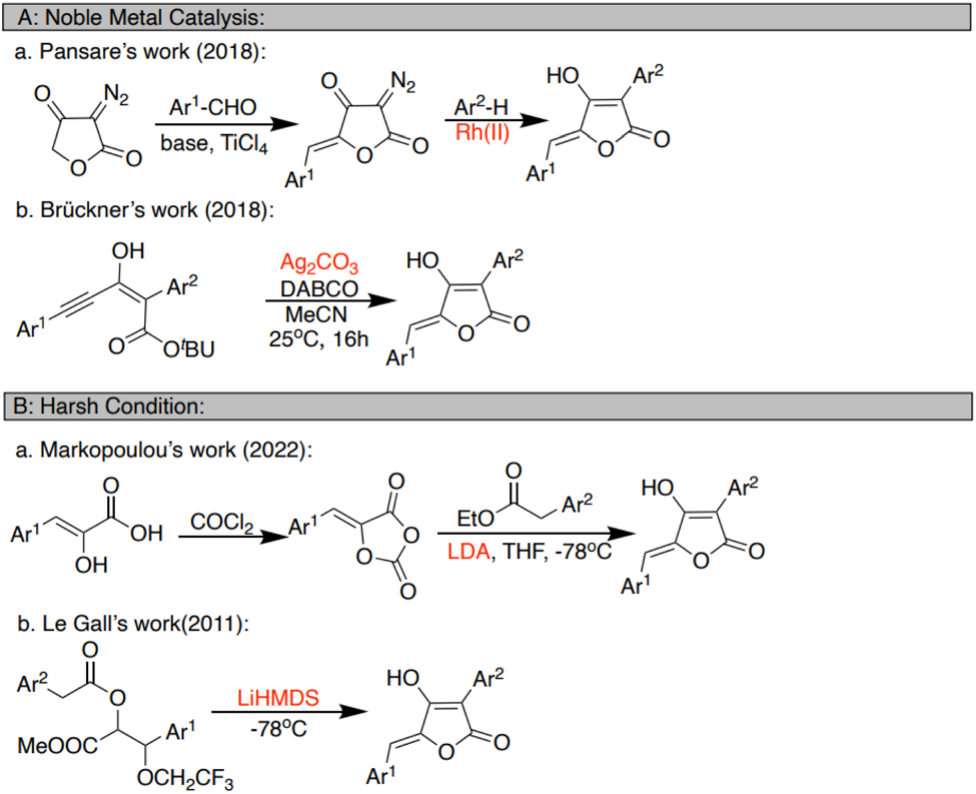
Previous synthetic approaches for the aspulvinone.

As part of our work, aspulvinone O and H were isolated from the rice culture of *Aspergillus terreus* and identified as potent bioactive inhibitors of GOT1 and novel anti-tumor agents for PDAC therapy.^8,9^ However, the poor isolated yield and limited structure variation restricted our further study of their pharmaceutical properties. The diverse activities of aspulvinone have attracted extensive attention from synthetic chemists. The synthetic approaches can be divided into three categories: noble metal catalysis,^10–13^ harsh conditions,^14–16^ and other conditions.^2,17,18^ Some of the methods reported after 2010 are listed in Scheme 1. However, noble metal catalysts often face the problem of high cost and scarce availability. The use of organolithium reagents requires low temperature (−78 °C) and the Schlenk technique, which is relatively hard to operate in labs and factories.

**Scheme 2:**
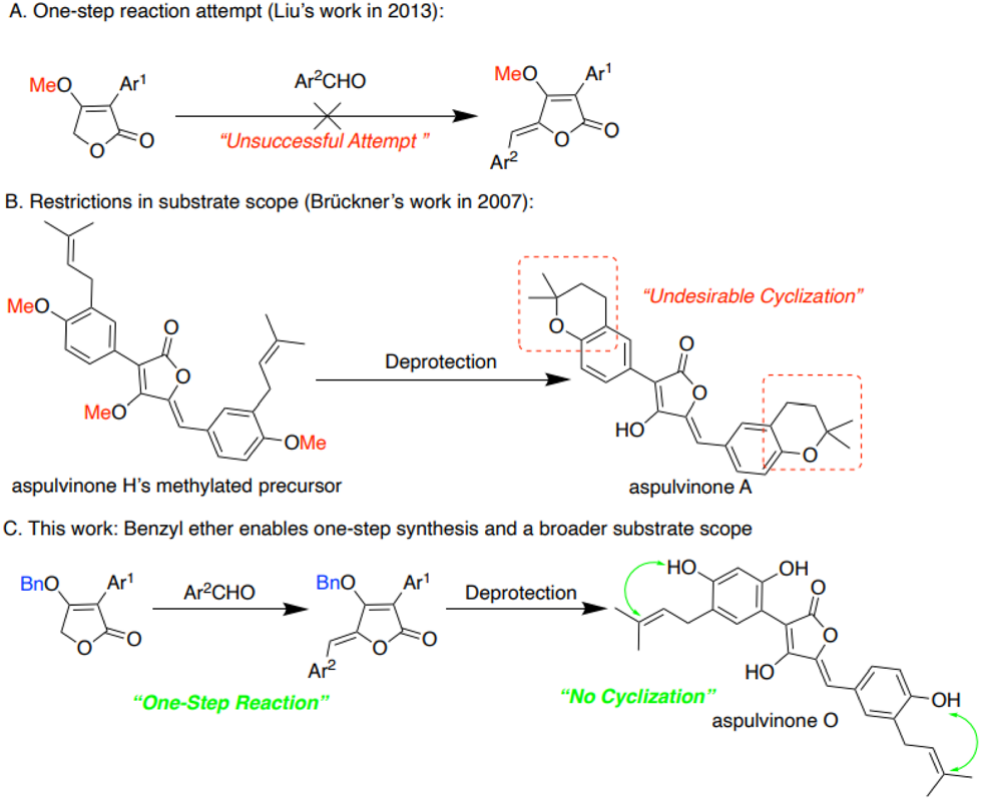
Synthetic Strategies of the aspulvinone.

Our group aims to develop a new method which is economical and easy to operate in a short route. In Liu's work, a two-step method was developed to synthesize the precursor of aspulvinone in a mild condition (Scheme 2A).^2^ Besides, in synthesizing aspulvinone H, Brückner and co-workers obtained unexpected aspulvinone A after deprotection. They assumed that deprotection of methyl ether using BBr_3_ resulted in the cyclization of the prenyl chain with the neighboring phenol group (Scheme 2B).^13^ Based on their work, we modified the starting material to 4-(benzyloxy)-3-phenyl-5-furan-2(5*H*)-one, hoping to find a one-step method for the aldol condensation, which can also be used in a broad substrate scope (Scheme 2C).

## Results and discussion

**Scheme 3:**
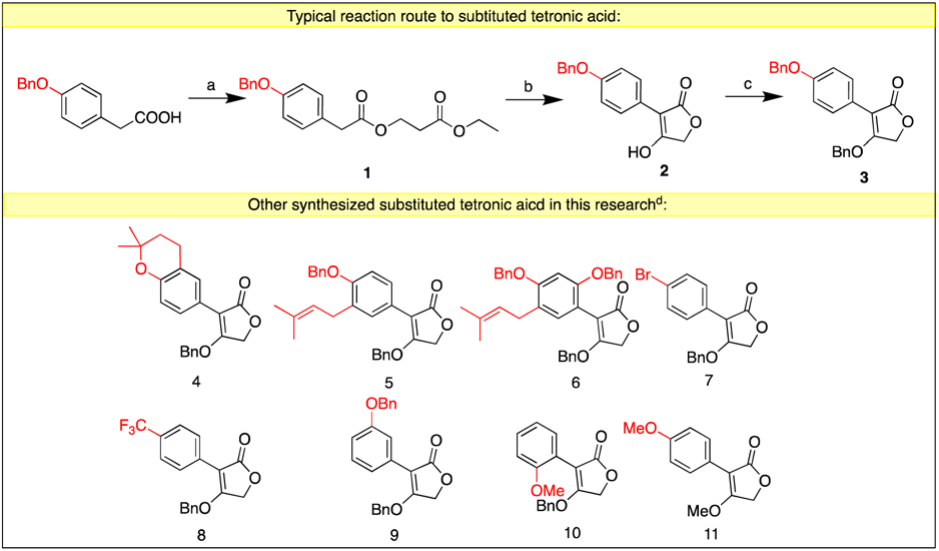
The synthesis of substituted tetronic acid. Reagents and conditions: ^*a*^ethyl chloroacetate, Et_3_N, THF, reflux, 12 h, 90%. ^*b*^*t*-BuOK, DMF, 0 °C to rt, 2 h, 82%. ^*c*^K_2_CO_3_, BnBr, DMF, rt, 12 h, 35% or BnOH, Ph_3_P, DEAD, rt, 12 h, 65%. ^*d*^Detailed procedures and yields are in ESI.†

Our work started from the synthesis of the substituted tetronic acid, 4-(benzyloxy)-3-(4-(benzyloxy) phenyl)furan-2(5*H*)-one (3). The commercially available *p*-benzyloxy phenylacetic acid was treated with ethyl chloroacetate and triethylamine to afford the acyclic ester (1), which then underwent an intramolecular cyclization by *t*-BuOK to afford the 4-hydroxy-3-(2-methoxyphenyl)furan-2(5*H*)-one (2). It was protected by the use of BnBr or BnOH to afford compound 3. Other substituted tetronic acids were synthesized using the same route from the different starting materials (Scheme 3).

Next, optimization of the aldol condensation between tetronic acid 3 and 4-(benzyloxy)benzaldehyde was performed. In Chopin's work, they proposed a method for aldol condensation using 1,8-diazabicyclo[5.4.0]undec-7-ene (DBU) as the base in acetonitrile (MeCN) at 65 °C.^19^ Initially, we used this method for aspulvinone's synthesis and successfully gained the desired product (Table 1, Entry 1). Then, the reactions were conducted at 65 °C employing DBU (2 eq.) as the base in various solvents, including tetrahydrofuran (THF), dichloromethane (DCM), methanol (MeOH) and isopropanol (*i*-PrOH) (Table 1, entry 2–5). With these tested solvents, MeOH afforded a methylated product, and *i*-PrOH afforded a mixture of *Z*/*E* products. MeCN showed the best yield in all solvents. To be noted, these results were quite different from Chopin's work, probably due to the difference in substrates. Next, several bases, including triethylamine (Et_3_N), 1,5-diazabicyclo[4.3.0]non-5-ene (DBN), 4-dimethylaminopyridine (DMAP), potassium *tert*-butoxide (*t*-BuOK), sodium hydroxide (NaOH), and potassium carbonate (K_2_CO_3_), were tested for this reaction (Table 1, entry 6–11), among which DBN showed a slight increase in yield. Then, different temperatures were tested (Table 1, entry 12–16), and the reaction at 30 °C gave the best yield. High and low temperatures lead to low yield, which is an interesting phenomenon that deserves more study. Finally, different times and equivalents of reactants and reagents were checked (Table 1, entry 17–20). It was found that extending reaction time, decreasing DBN, and increasing the aldehyde could increase the reaction yield (Table 1, entry 20). The reaction of 1.0 g scale was also performed in the same condition and gained the same yield.

**Table 1:** Screening of the Reaction Solvent and Base^a^

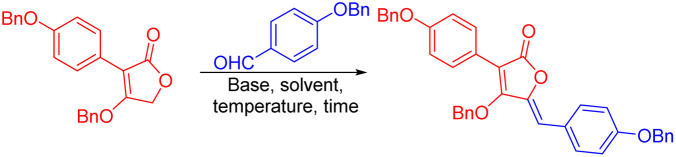						
Entry	Temperature (°C)	Time (h)	Solvent		Base (eq.)	Yield (%)^b^
1	65	12	MeCN	DBU (2)		34
2	65	12	THF	DBU (2)		21
3	40	12	DCM	DBU (2)		6
4	65	12	MeOH	DBU (2)		36^c^
5	65	12	*i*-PrOH	DBU (2)		28^d^
6	65	12	MeCN	Et_3_N (2)		Trace
7	65	12	MeCN	DBN (2)		38
8	65	12	MeCN	DMAP (2)		Trace
9	65	12	MeCN	*t*-BuOK (2)		16
10	65	12	MeCN	NaOH (2)		25
11	65	12	MeCN	K_2_CO_3_ (2)		12
12	80	12	MeCN	DBN (2)		10
13	45	12	MeCN	DBN (2)		42
14	30	12	MeCN	DBN (2)		46
15	15	12	MeCN	DBN (2)		24
16	0	12	MeCN	DBN (2)		5
17	30	24	MeCN	DBN (2)		49
18	30	48	MeCN	DBN (2)		55
19	30	48	MeCN	DBN (1)		55
20^e^	30	48	MeCN	DBN (1)		65

a Standard condition: tetronic acid (50 mg), aldehyde (57 mg, 2 eq.), solvent (1 ml), base (2 eq.).

b Isolated yields.

c Methylated product.

d *Z*/*E* = 1.7 : 1.

e The equivalent of aldehyde is 3.

**Scheme 4:**
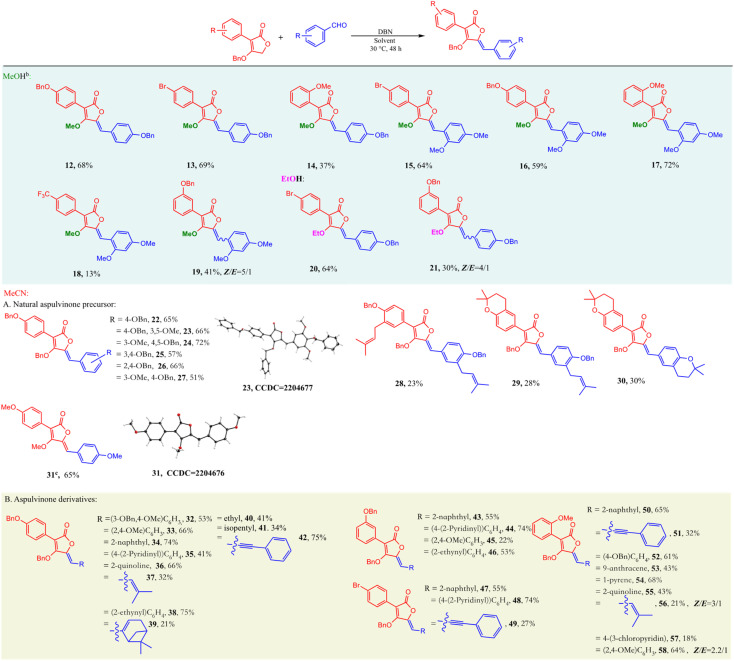
Substrate scope. ^*a*^Isolated yields. ^*b*^60 °C. ^*c*^Compound 31 is a natural product.^20^

After optimization, the modular condensation between different substituted tetronic acids and aldehydes to afford various aspulvinone-skeleton products was investigated. Firstly, a series of fully methylated products (12–19) and partly ethylated ones (20–21) were synthesized using MeOH or EtOH as solvent. Then, some natural products protected by benzyl groups (22–30) were synthesized with similar conditions using MeCN as solvent. Some of them can be purified by filtration since precipitation of the products formed in the solvent. Next, more aspulvinone derivatives were synthesized from hetero cyclic aldehyde and alkyl ones (32–58). Most compounds are pure *Z* isomers except some derivatives from compound 10 (Scheme 4). Next, we conducted an investigation to gain insights into the factors controlling the reaction.

**Scheme 5:**
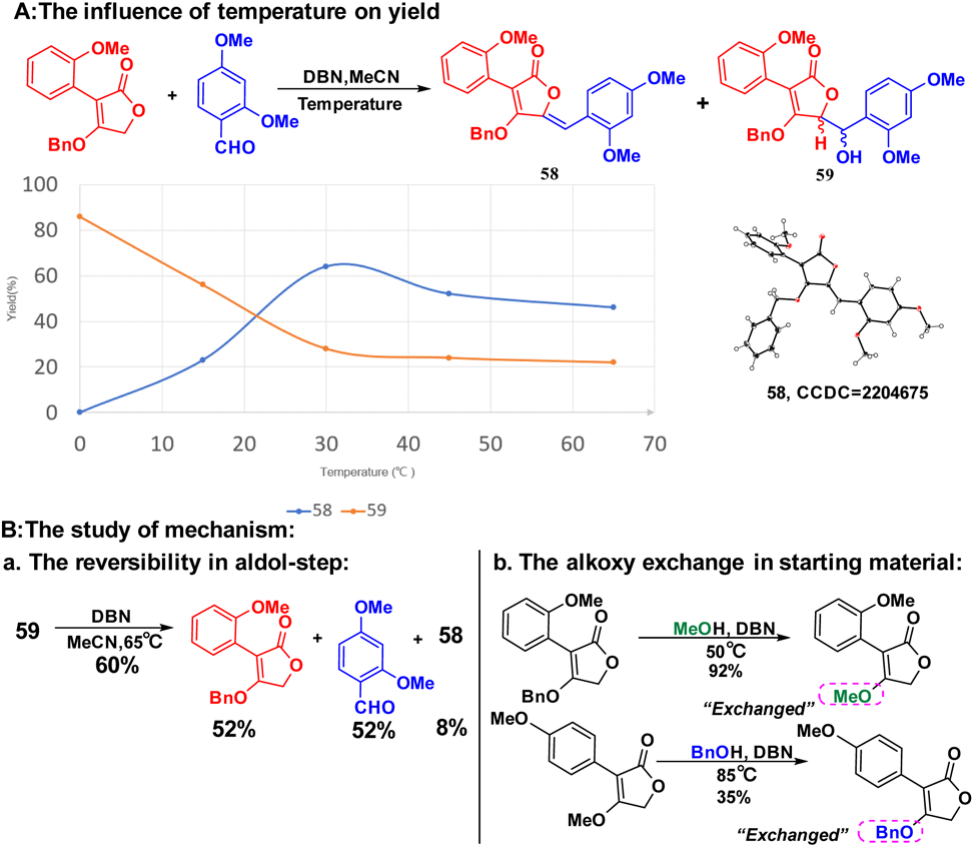
The study of mechanism.

**Scheme 6:**
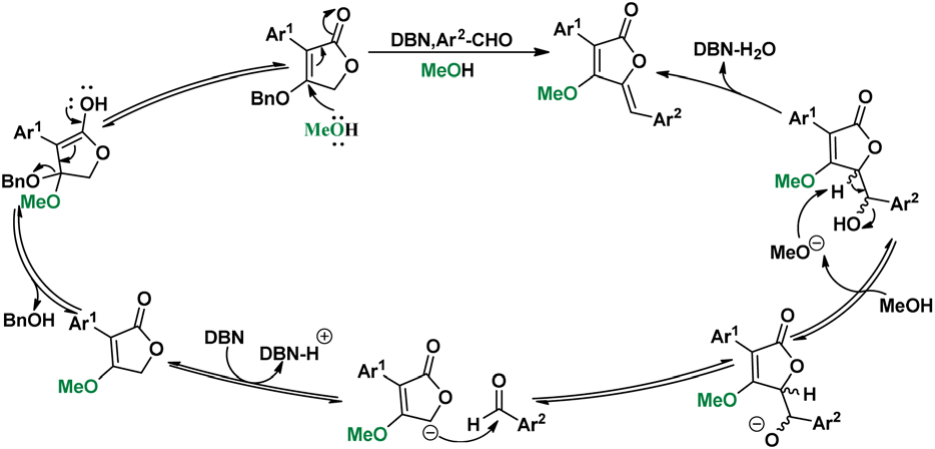
Plausible Mechanism.

In the substrate exploration, the reaction for synthesizing compound 58 is formed with by-product 59, which is the only reaction where an addition intermediate can be obtained. Then, the influence of temperatures was studied on the yield of 58 and 59 (Scheme 5, A). It was found that the yield of 58 was gradually increased from 0 to 30 °C and then decreased from 30 to 65 °C. While the best yield of 59 was at 0 °C, its yield decreased as the temperature increased. Additional controlled experiments using 59 further illustrated this (Scheme 5, Ba). However, no elimination product can be found in low temperatures, indicating the necessity of high-temperature for elimination. Interestingly, a unique equilibrium occurred at 30 °C, where both the aldol step and the elimination proceeded smoothly and afforded the best yield. Then, the alkoxy exchange mechanism was studied by using the starting material in MeOH condition (Scheme 5, Bb). We found that the exchange only took place in the starting material stage, thus proposing a mechanism containing both alkoxy exchange and aldol condensation (Scheme 6).

**Scheme 7:**
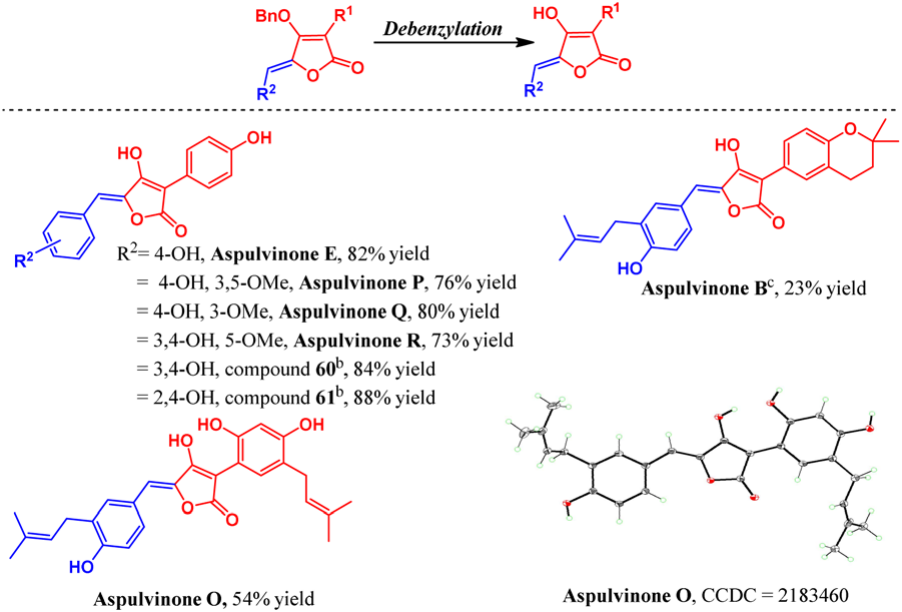
The deprotection and synthesis of natural aspulvinones in this study. ^*a*^ isolated yields. ^*b*^Unnamed natural aspulvinones.^26^^*c*^Aspulvinone B use Cy_2_NMe and BCl_3_ for deprotection.^13^

Next, the natural aspulvinone precursors were deprotected, and eight new natural aspulvinones were obtained. Six of them, including aspulvinone O, P, Q, R, compound 60 and 61, were synthesized for the first time. Pd/C and H_2_ were used for most compounds to cleave the *O*-benzyl bond. However, the deprotection of aspulvinone B precursor using this method invariably resulted in partial hydrogenation of the prenyl double bond. Finally, the Lewis acid BCl_3_ was used for deprotection and successfully delivered aspulvinone B (Scheme 7).^13^

In the optimization process, we found that the mixture of *Z*/*E* products can be formed in aspulvinone E precursor using *i*-PrOH in reflux condition. Through screening, two natural product precursors were formed with *Z*/*E* mixed configurations and transformed to natural aspulvinones of different configurations by deprotecting them. Isoaspulvinone have been observed in some researches.^18,21–23^ Our NMR data for isoaspulvinone E are consistent with those reported in Gao's work.^21^ The *E* configuration of aspulvinone E and P showed similar NMR changes compared to the *Z* configuration, which is consistent with Campbell's work.^18^

M^pro^ and PL^pro^ are two important proteases for SARS-CoV-2 and other coronaviruses and essential for viral replication and transcription.^24,25^ These aspulvinone analogues exhibited modest antiviral activity in the high-throughput screening assay. Aspulvinone E and 49 inhibited SARS-CoV-2 M^pro^ activity with IC_50_ of 39.93 ± 2.42 μM and 28.25 ± 2.37 μM, respectively (Fig. S1B, Table S1†). 51 and 50 inhibited SARS-CoV-2 PL^pro^ activity with IC_50_ of 23.05 ± 0.07 μM and 17.43 ± 2.60 μM, respectively (Fig. S1A, Table S2†). Notably, aspulvinone O exhibited dual-antiviral activity towards SARS-CoV-2 M^pro^ and PL^pro^, with IC_50_ of 12.41 ± 2.40 μM and 21.34 ± 0.94 μM, respectively (Fig. S1A and S1B, Table S1 and S2†).

**Fig. 1:**
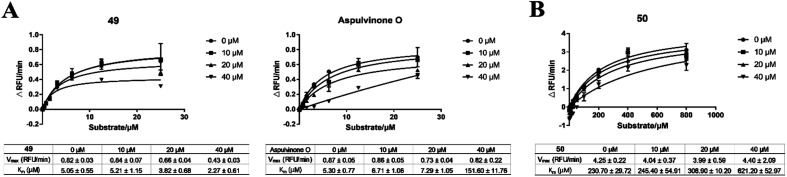
Antiviral activity of aspulvinone analogues. (A) Mechanisms of action of 49 and aspulvinone O against SARS-CoV-2 M^pro^. 49 inhibited M^pro^ in an uncompetitive way, while aspulvinone O in a competitive way. (B) The mechanism of action of 50 against SARS-CoV-2 PL^pro^, which inhibited PL^pro^ in a competitive way.

Further enzyme kinetics assays revealed that aspulvinone E (Fig. S1C†) and 49 (Fig. 1A) showed uncompetitive inhibition against SARS-CoV-2 M^pro^, with reduced *V*_max_ and *K*_m_ values. In contrast, aspulvinone O showed competitive inhibition against SARS-CoV-2 M^pro^, with almost unchanged *V*_max_ values and gradually increased *K*_m_ values (Fig. 1A). The same assays were also performed against SARS-CoV-2 PL^pro^, which revealed that aspulvinone O, 51, and 50 all behaved as competitive inhibitors, consistent with GRL0617 (Fig. 1B, S1D†). These results gave substantial evidence for the docking analysis in Hawary's work.^27^

## Conclusions

In conclusion, we have developed a method with high yield, mild conditions, and wide substrate tolerance to synthesize aspulvinones and their derivatives. This is a useful protocol for researchers who are interested in aspulvinones to quickly obtain their desired products. We found an interesting alkoxy exchange mechanism during the synthesis, further broadening the substrate scope. By using *i*-PrOH, we successfully synthesized aspulvinones with *E* configuration after separation by HPLC. In addition, our synthetic aspulvinone O, E, and derivatives 49, 50, and 51 exhibited modest anti-SARS-CoV-2 activity, which may provide new ideas for the design of lead compounds to treat COVID-19 in the future.

## Author contributions

Xiaotan Yu and Xiaoxia Gu contributed equally to this work. Xiaotan Yu completed the research of the organic synthesis part, and Xiaoxia Gu completed the research of the pharmacological part.

## Conflicts of interest

There are no conflicts to declare.

## Supplementary Material

RA-013-D2RA08133D-s001

RA-013-D2RA08133D-s002
