# Supplementary material for: Collective synthesis of aspulvinone and its analogues by vinylogous aldol condensation of substituted tetronic acids with aldehydes
Source: RSC Adv. 2023 Feb 7;13(7):4859–64. doi: 10.1039/d2ra08133d (PMC9903353; doi:10.1039/d2ra08133d)
Supplement: RA-013-D2RA08133D-s001 [file RA-013-D2RA08133D-s001.pdf]

Electronic Supplementary Material (ESI) for RSC Advances.
This journal is © The Royal Society of Chemistry 2023

Supporting Information

Collective Synthesis of Aspulvinones and their Analogues by Vinylogous Aldol Condensation of Substituted Tetronic Acids with Aldehydes

Xiaotan Yu[1,a], Xiaoxia Gu[1,a], Yunpeng Zhao[a], Fengqing Wang[a], Weiguang Sun[a], Changxing Qi*[,a], Lianghu Gu,*[,a] and Yonghui Zhang*[,a]

[a]Hubei Key Laboratory of Natural Medicinal Chemistry and Resource Evaluation, School of Pharmacy, Tongji Medical College, Huazhong University of Science and Technology, Wuhan 430030, People's Republic of China

Corresponding Author

*E-mail: zhangyh@mails.tjmu.edu.cn
*E-mail: gulianghu@hust.edu.cn
*Email: qichangxing@hust.edu.cn

Table of contents

1 General information 4

2 Procedures for the preparation of substituted tetronic acid **3-11**... 5

2.1 The preparation of compound **3, 7, 8, 9, 10, 11**.. 5

2.1.1 The benzylation of phenolic group... 5

2.1.2 The synthesis of tetronic acids... 6

2.1.3 The benzylation of tetronic acids... 7

2.1.4 The methylation of tetronic acids 10

2.2 The preparation of compound **4, 5** 10

2.2.1 The preparation of compound **4**... 10

2.2.2 The preparation of compound **5**... 12

2.3 The preparation of compound **6** 13

2.3.1 The preparation of compound **6b** 13

2.3.2 The preparation of compound **6d** 14

2.3.3 The preparation of compound **6f** 15

2.3.4 The preparation compound **6**.. 15

3 Synthesis of the precursor for aspulvinone O and B 17

4 Synthesis of the aspulvinone derivatives... 18

4.1 General procedures... 18

4.2 Products in MeOH condition.. 18

4.3 Products in EtOH condition.. 21

4.4 Products in MeCN condition.. 22

4.4.1 Natural aspulvinone precursors 22

4.4.2 Aspulvinone derivatives 25

5 Mechanism study.. 36

5.1 The preparation of compound **59** 36

5.2 The exchange of methyl and benzyl groups 36

6 Deprotection of natural aspulvinone precursors.. 38

6.1 general procedure for aspulvinone E, P, Q, R, O and unnamed aspulvinone **62, 63**..... 38

6.2 Procedures for aspulvinone B... 40

7 Synthesis of the isoaspulvinone.. 42

8 Pharmacological results and methods 44
8.1 Results 44
8.2 Method... 46
8.2.1 Protein Expression and Purification 46
8.2.2 Enzymatic activity and inhibition assays.. 47
9 NMR spectra.. 44
10 X-ray crystallographic analysis 131
10.1 X-ray structure of **23** 131
10.2 X-ray structure of **31** 132
10.3 X-ray structure of **58** 134
10.4 X-ray structure of aspulvinone O 135
11 Reference.. 137

1 General information

All reagents and solvents were purchased from Adamas-beta, Sigma-Aldrich, Bidepharm, TCI, Alfa Aesar, or Innochem and used without purification. Column chromatography was performed using silica gel (100 – 200 mesh and 200 – 300 mesh, Qingdao Marine Chemical Inc., Qingdao, China). Reactions were monitored by thin layer chromatography (TLC) using silica gel 60 F254 (Yantai Chemical Industry Research Institute). NMR spectra were measured on 400 MHz and 600 MHz Bruker using $CDCl_3$, CD_3OD, DMSO-d_6, and Pyridine-d_5 as the solvent at room temperature. Chemical shifts (δ) are given in parts per million relatives to the solvent peak, and coupling constants (J) are given in hertz. High-resolution electrospray ionization mass spectroscopy (HRESIMS) was conducted on a microOTOF-Q-II acquisition parameter (Bruker, Germany). The crystallographic experiments were performed on a D8 Quest (Bruker, Germany) and an XtaLAB PRO MM007HF (Rigaku, Japan) using Cu Kα radiation. Semipreparative HPLC separations were conducted on an Agilent 1220 HPLC system with a UV detector using a reversed-phase C18 column (5 μm, 10 × 250 mm, YMCpack ODS-A). ^{1}H ,^{13}C spectra, and HRMS data were provided for all new compounds. Known compounds were provided with their ^{1}H spectra data and the reference in experimental details section.

2 Procedures for the preparation of substituted tetronic acid 3-11

2.1 The preparation of compound 3, 7, 8, 9, 10, 11

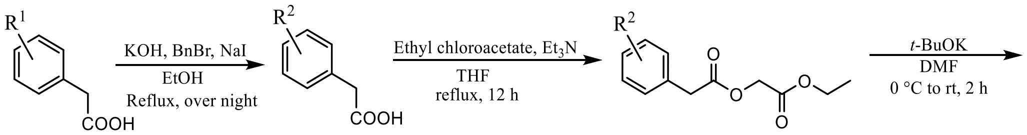

R^1 = 4-hydroxy, **3aa**
= 3-hydroxy, **9aa**

R^2 = 4-*O*-benzyl, **3a**
= 4-Br, **7a**
= 4-CF_3, **8a**
= 3-*O*-benzyl, **9a**
= 2-OMe, **10a**
= 4-OMe, **11a**

R^2 = 4-*O*-benzyl, **3b**
= 4-Br, **7b**
= 4-CF_3, **8b**
= 3-*O*-benzyl, **9b**
= 2-OMe, **10b**
= 4-OMe, **11b**

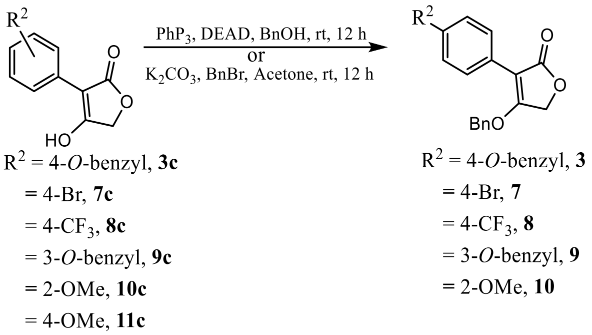

R^2 = 4-*O*-benzyl, **3c**
= 4-Br, **7c**
= 4-CF_3, **8c**
= 3-*O*-benzyl, **9c**
= 2-OMe, **10c**
= 4-OMe, **11c**

R^2 = 4-*O*-benzyl, **3**
= 4-Br, **7**
= 4-CF_3, **8**
= 3-*O*-benzyl, **9**
= 2-OMe, **10**

2.1.1 The benzylation of phenolic group

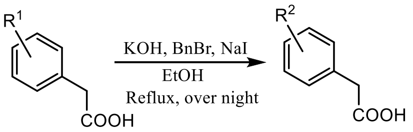

R^1 = 4-hydroxy, **3aa**
= 3-hydroxy, **9aa**

R^2 = 4-*O*-benzyl, **3a,** 64%
= 3-*O*-benzyl, **9a,** 62%

3aa and **9aa** (15.2 g, 100 mmol), benzyl bromide (18.8 g, 110 mmol), KOH (14 g, 250 mmol), and NaI (299.8 mg, 2 mmol) were dissolved in EtOH (500 mL) and refluxed for 20 h. The solution was allowed to stand at room temperature and then treated with 3 N aq HCl (450 mL). The precipitate was filtered, washed with H_2O (300 mL), and dried under vacuum to give the compound **3a** and **9a** as colorless powders. The products were used directly for next step without purification.[1]

2.1.2 The synthesis of tetronic acids

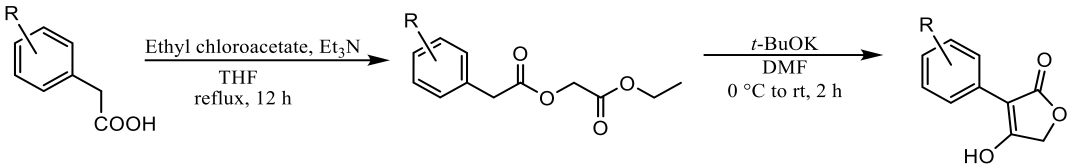

R = 4-*O*-benzyl, **3a**	R = 4-*O*-benzyl, **3b**	R = 4-*O*-benzyl, **3c,** 74%
= 4-Br, **7a**	= 4-Br, **7b**	= 4-Br, **7c,** 77%
= 4-CF_3, **8a**	= 4-CF_3, **8b**	= 4-CF_3, **8c,** 72%
= 3-*O*-benzyl, **9a**	= 3-*O*-benzyl, **9b**	= 3-*O*-benzyl, **9c,** 71%
= 2-OMe, **10a**	= 2-OMe, **10b**	= 2-OMe, **10c,** 78%
= 4-OMe, **11a**	= 4-OMe, **11b**	= 4-OMe, **11c,** 74%

Triethylamine (5.34 g, 52.8 mmol) and ethyl chloroacetate (6.47 g, 52.8 mmol) were added to the solution of phenylacetic acid **3a, 7a, 8a, 9a, 10a, 11a** (44.1 mmol) in dry THF (200 mL). The reaction mixture was stirred for 6-7 hour at about 65 °C. The reaction was monitored using TLC. After reaction completed, the solid was filtered off, and the solvent was removed on rotary evaporator. The residue was then diluted with H_2O (400 ml) and extracted by diethyl ether (3 × 200 mL). The organic phase was dried over anhydrous Na_2SO_4, and the solvent removed. The crude product was not purified and applied directly to the next step. Potassium tert-butoxide (9.90 g, 88.2 mmol) was added into the solution of compound **3b, 7b, 8b, 9b, 10b, 11b** (44.1 mmol) in dry DMF (30 mL) in six portions over 0.5 hours at 0 °C. The reaction mixture was then warmed to room temperature, stirred for another 2-4 hours. After the reaction completed, the mixture was added 45 mL water and acidified by diluted hydrochloric acid to pH of 3-4. Then 600 mL water was added to the solution, the precipitate yielded and was filtered and dried to give the target compounds. Compound **3c, 7c, 8c, 9c, 10c, 11c** are all known compounds according to the literature. [2-5]

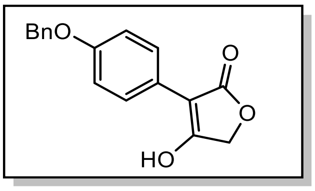

3-(4-(benzyloxy) phenyl)-4-hydroxyfuran-2(5*H*)-one: Compound **3c** was isolated as a white solid (9.2 g, 74% yield). **^{1}H NMR** (400 MHz, DMSO-*d*$_6$) *δ* 7.87 – 7.81 (m, 2H), 7.48 – 7.27 (m, 6H), 7.05 – 6.98 (m, 2H), 5.09 (s, 2H), 4.72 (s, 2H).

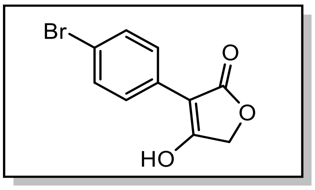

3-(4-bromophenyl)-4-hydroxyfuran-2(5*H*)-one: Compound **7c** was isolated as a white solid (8.66 g, 77% yield). **^{1}H NMR** (400 MHz, DMSO-*d*$_6$) *δ* 7.94 – 7.86 (m, 2H), 7.60 – 7.52 (m, 2H), 4.76 (s, 2H).

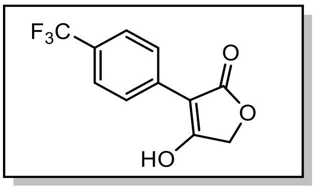

4-hydroxy-3-(4-(trifluoromethyl) phenyl) furan-2(5*H*)-one: Compound **8c** was isolated as a white solid (7.75 g, 72% yield). ^{1}**H NMR** (400 MHz, DMSO-d_6) δ 8.16 (d, J = 8.2 Hz, 2H), 7.72 (d, *J* = 8.3 Hz, 2H), 4.80 (s, 2H). ^{19}**F NMR** (565 MHz, DMSO-d_6) δ -60.81.

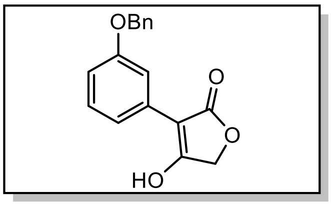

3-(3-(benzyloxy) phenyl)-4-hydroxyfuran-2(5*H*)-one: Compound **9c** was isolated as a white solid (8.83 g, 71% yield). ^{1}**H NMR** (400 MHz, DMSO-d_6) δ 7.61 (dt, *J* = 3.0, 1.5 Hz, 1H), 7.54 (dq, *J* = 7.9, 1.3 Hz, 1H), 7.49 – 7.43 (m, 2H), 7.43 – 7.24 (m, 4H), 6.96 – 6.82 (m, 1H), 5.09 (s, 2H), 4.76 (s, 2H).

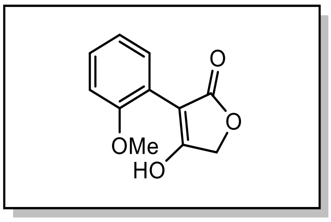

4-hydroxy-3-(2-methoxyphenyl) furan-2(5*H*)-one: Compound **10c** was isolated as a yellow oil (7.08 g, 78% yield). ^{1}**H NMR** (600 MHz, DMSO-d_6) δ 7.32 (ddd, *J* = 8.9, 7.4, 1.8 Hz, 1H), 7.17 (dq, *J* = 7.6, 1.7 Hz, 1H), 7.03 (dd, *J* = 8.4, 1.0 Hz, 1H), 6.95 (td, *J* = 7.4, 1.2 Hz, 1H), 4.74 (d, *J* = 1.5 Hz, 2H), 3.74 (s, 3H).

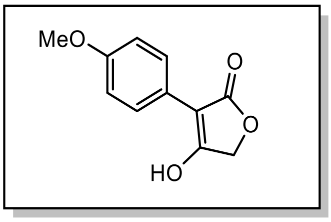

4-hydroxy-3-(4-methoxyphenyl)furan-2(5*H*)-one:Compound **11c** was isolated as a yellow solid (6.72 g, 74% yield). ^{1}**H NMR** (600 MHz, Methanol-d_4) δ 7.86 – 7.81 (m, 2H), 6.94 – 6.89 (m, 2H), 4.72 (t, *J* = 1.3 Hz, 2H), 3.79 (d, *J* = 1.1 Hz, 3H).

2.1.3 The benzylation of tetronic acids[6]

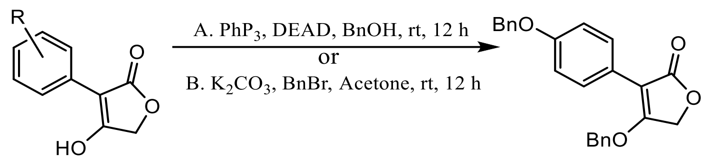

R = 4-*O*-benzyl, **3c**
= 4-Br, **7c**
= 4-CF_3, **8c**
= 3-*O*-benzyl, **9c**
= 2-OMe, **10c**

R = 4-*O*-benzyl, **3**, 68% or 36%
= 4-Br, **7**, 64% or 32%
= 4-CF_3, **8**, 56% or 22%
= 3-*O*-benzyl, **9,** 61% or 31%
= 2-OMe, **10,** 51% or 40%

Method A: Hexadecanol (290 mg, l.2 mmol), 1 mmol of tetronic acid and 314 mg (l.2 mmol) of PPh_3 were dissolved in 10 ml of dry THF and 0.19 ml (1.2 mmol) of DEAD in 5 ml of THF was added. The reaction was stirred overnight at room temperature. The reaction mixture was then concentrated in vacuum and the residue was chromatographed on silica gel with PE/EA (2: l) as the eluant.
Method B: Potassium carbonate (13.8 g, 100 mmol) was added to a solution of tetronic acid (50 mmol) in acetone (100 mL). After stirring for 45 min at room temperature, benzyl bromide (6.54 mL, 50 mmol) was added. Acetone was removed under vacuum after stirring 12 h at room temperature, then ethyl acetate was added to the residue obtained. The organic phase was washed with water and separated by a separatory funnel. The organic phases were dried over $MgSO_4$, filtered, and concentrated under vacuum. The crude product was purified by silica gel chromatography with PE/EA (2: l) as the eluant.

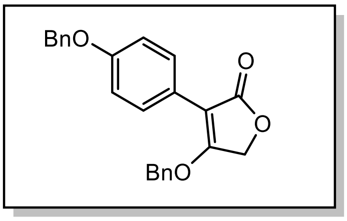

4-(benzyloxy)-3-(4-(benzyloxy)phenyl)furan-2(5*H*)-one: Compound **3** was isolated as a white solid (6.70 g, 36% yield). **^{1}H NMR** (600 MHz, Chloroform-*d*) δ 7.88 – 7.82 (m, 2H), 7.45 – 7.41 (m, 2H), 7.44 – 7.38 (m, 2H), 7.41 – 7.32 (m, 4H), 7.35 – 7.25 (m, 2H), 7.03 – 6.97 (m, 2H), 5.17 (s, 2H), 5.08 (s, 2H), 4.80 (s, 2H); **^{13}C NMR** (151 MHz, Chloroform-*d*) δ 172.7, 170.7, 158.2, 136.9, 134.7, 129.2, 129.1, 129.1, 128.6, 128.0, 127.5, 127.3, 122.0, 114.7, 103.3, 72.6, 70.0, 65.0; **HRMS** (ESI-TOF) *m/z* calculated for $C_{24}H_{20}O_4$ $(M + Na)^+$: 395.1259, found 395.1264.

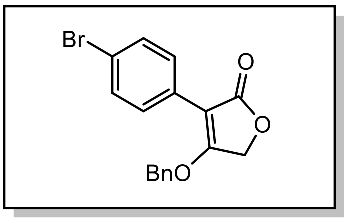

3-(4-bromophenyl)-4-hydroxyfuran-2(5*H*)-one: Compound **7** was isolated as a yellow solid (4.05 g, 32% yield). **^{1}H NMR** (400 MHz, Chloroform-*d*) δ 7.87 – 7.80 (m, 2H), 7.54 – 7.48 (m, 2H), 7.51 – 7.35 (m, 3H), 7.35 (dd, J = 7.4, 2.2 Hz, 2H), 5.21 (s, 2H), 4.84 (s, 2H); **^{13}C NMR** (101 MHz, Chloroform-*d*) δ 172.5, 172.2, 134.5, 131.7,

129.5, 129.4, 129.4, 128.4, 127.5, 121.8, 102.7, 73.1, 65.1; **HRMS** (ESI-TOF) *m/z* calculated for $C_{17}H_{13}BrO_3$ (M + Na) $^+$: 366.9946, found 366.9940.

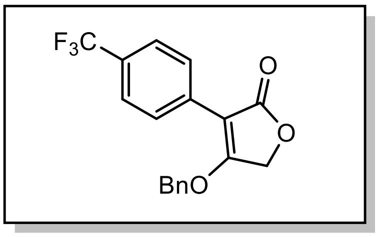

4-(benzyloxy)-3-(4-(trifluoromethyl)phenyl)furan-2(5*H*)-one: Compound **8** was isolated as a yellow solid (3.67 g, 22% yield). **^{1}H NMR** (400 MHz, Chloroform-*d*) δ 8.10 – 8.03 (m, 2H), 7.65 – 7.58 (m, 2H), 7.47 – 7.35 (m, 3H), 7.34 (dd, J = 7.3, 2.3 Hz, 2H), 5.23 (s, 2H), 4.87 (s, 2H);**^{13}C NMR** (101 MHz, Chloroform-*d*) δ 173.9, 172.3, 134.6, 133.3, 129.9, 129.8, 128.2, 127.8, 126.0, 125.8, 125.7 (q, *J* = 3.9 Hz), 125.7, 125.6, 102.8, 73.6, 65.4; **^{19}F NMR** (565 MHz, Chloroform-*d*) δ -61.0; **HRMS** (ESI-TOF) *m/z* calculated for $C_{18}H_{13}F_3O_3$ (M + Na)$^+$: 357.0714, found 357.0710.

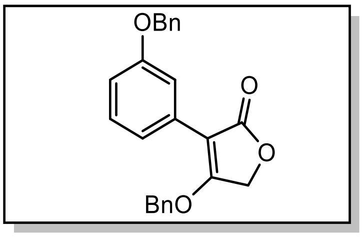

4-(benzyloxy)-3-(3-(benzyloxy)phenyl)furan-2(5*H*)-one: Compound **9** was isolated as a white solid (5.76 g, 31% yield). **^{1}H NMR** (400 MHz, Chloroform-*d*) δ 7.61 – 7.56 (m, 1H), 7.52 (dt, *J* = 7.8, 1.2 Hz, 1H), 7.45 – 7.23 (m, 11H), 6.93 (ddd, *J* = 8.3, 2.6, 1.0 Hz, 1H), 5.16 (s, 2H), 5.04 (s, 2H), 4.80 (s, 2H); **^{13}C NMR** (101 MHz, Chloroform-*d*) δ 172.8, 172.6, 159.1, 137.6, 135.1, 131.0, 129.8, 129.7, 129.6, 129.1, 128.4, 128.0, 127.8, 121.1, 115.3, 114.4, 103.8, 73.3, 70.4, 65.4; **HRMS** (ESI-TOF) *m/z* calculated for $C_{24}H_{20}O_4$ (M + Na)$^+$: 395.1259, found 395.1264.

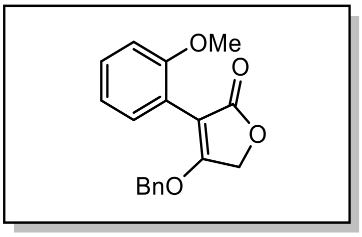

4-(benzyloxy)-3-(2-methoxyphenyl)furan-2(5*H*)-one: Compound **10** was isolated as a yellow oil (4.59 g, 40% yield). **^{1}H NMR** (600 MHz, Chloroform-*d*) δ 7.38 – 7.31 (m, 2H), 7.32 (t, *J* = 1.9 Hz, 2H), 7.27 – 7.22 (m, 1H), 7.18 – 7.13 (m, 2H), 7.02 – 6.96 (m, 1H), 6.93 (d, *J* = 8.3 Hz, 1H), 4.99 (d, *J* = 1.5 Hz, 2H), 4.74 (d, *J* = 1.5 Hz, 2H), 3.80 (d, *J* = 1.4 Hz, 3H); **^{13}C NMR** (101 MHz, Chloroform-*d*) δ 173.8, 172.2, 157.8, 135.2, 132.2, 130.3, 128.9, 128.9, 127.8, 120.8, 118.8, 111.2, 101.0, 73.1, 67.2, 55.7; **HRMS** (ESI-TOF) *m/z* calculated for $C_{18}H_{16}O_4$ (M + Na)$^+$: 319.0946, found 319.0938.

2.1.4 The methylation of tetronic acid

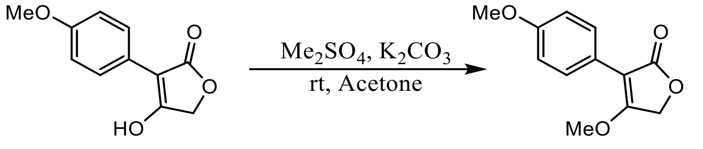

11c **11**

To the solution of compound **11c** (5.0 g, 24.3mmol), in acetone (50 ml), anhydrous potassium carbonate (4.36 g, 31.6 mmol) was mixtured at room temperature. Then a solution of dimethyl sulfate (3.68 g, 29.16mmol) in acetone (30 mL) was added in dropwise. The reaction was monitored by TLC. After the reaction completed, the solvent was removed under reduced pressure. Then 250 mL water was added to dissolve the excess anhydrous potassium carbonate, and the residue was extracted using EtOAc (50ml × 3). The organic phase was dried over anhydrous Na_2SO_4, and the solvent was removed. The residue was purified by column chromatography (PE: EA=4:1) to give the compound **11**. Compound **11** is a known compound.[2]

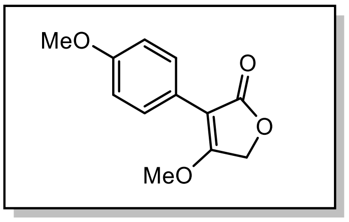

4-methoxy-3-(4-methoxyphenyl) furan-2(5*H*)-one: Compound **11** was isolated as a yellow solid (4.81g, 90% yield). **^{1}H NMR** (600 MHz, Chloroform-*d*) δ 7.83 – 7.77 (m, 2H), 6.95 – 6.88 (m, 2H), 4.84 (s, 2H), 3.96 (s, 3H), 3.82 (s, 3H).

2.2 The preparation of compound 4, 5

2.2.1 The preparation of compound 4

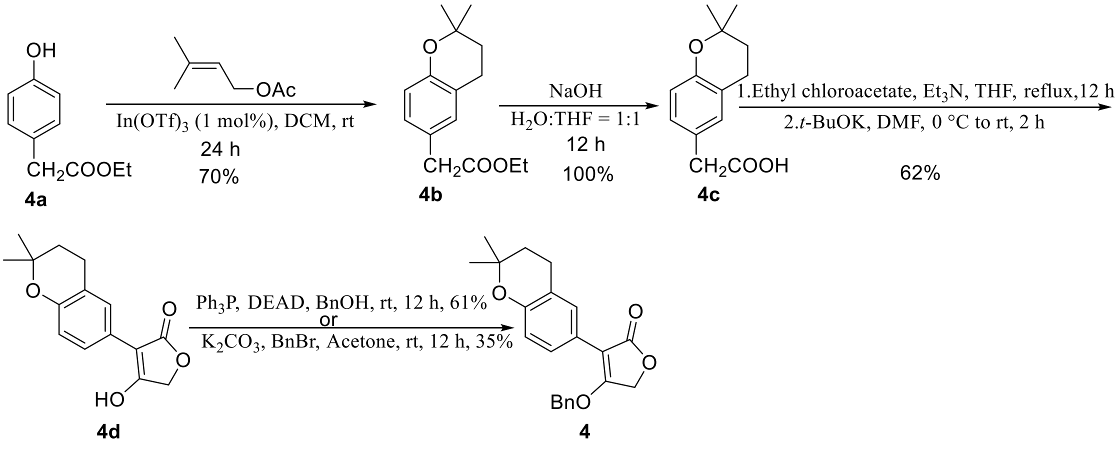

2.2.1.1 The preparation of compound 4b[7]

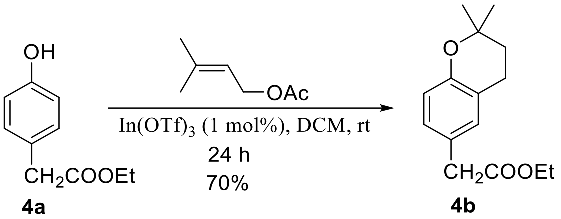

4a (30.0 mmol), prenyl acetate (30.0 mmol) and In(OTf)$_3$ (0.3 mmol) were stirred in DCM (100mL) at 25 °C for 24 h. At the end of reaction, the crude mixture was added to aqueous 1M NaOH (300 mL). The aqueous layer was extracted with Et$_2$O (3 × 50 mL). The combined organic layers were washed with aqueous 1M NaOH (3 × 100 mL), 1M HCl (3 × 100 mL) and saturated aqueous solution of NaCl (100 mL), dried with magnesium sulfate and concentrated under reduced pressure. Final products were simply purified by column chromatography to remove **4a** on silica gel (PE: EA=10:1).

2.2.1.2 The preparation of compound 4

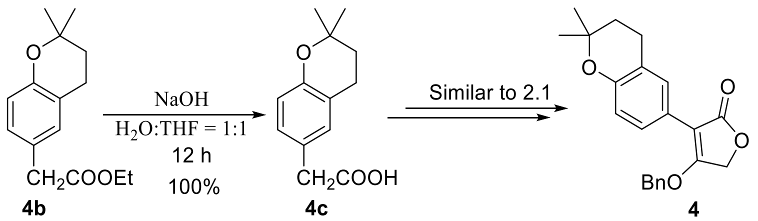

4b (8.0 mmol) was dissolved in THF/water (1/1, 100 mL) followed by addition of NaOH (640mg, 16 mmol). The resulted mixture was heated at 60 °C for 12 h. The resulting reaction mixture was then cooled to room temperature, quenched with 100 mL of 10% aqueous HCl, extracted with EtOAc three times (100.0 mL each), washed with brine and dried over anhydrous Na$_2$SO$_4$. The organic layer was concentrated on a rotary evaporator under vacuum. The crude product was directly used for next steps without purification. The steps for **4c** to **4** are as same as the previous steps.

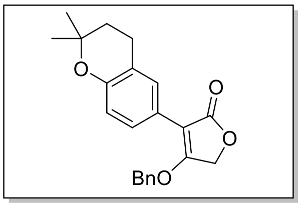

4-(benzyloxy)-3-(2,2-dimethylchroman-6-yl) furan-2(5*H*)-one: Compound **4** was isolated as a white solid (1.89 g, 35% yield). **^{1}H NMR** (400 MHz, Chloroform-*d*) δ 7.64 – 7.55 (m, 2H), 7.43 – 7.31 (m, 5H), 6.79 (d, J = 8.5 Hz, 1H), 5.17 (s, 2H), 4.78 (s, 2H), 2.79 (t, J = 6.7 Hz, 2H), 1.81 (t, J = 6.7 Hz, 2H), 1.34 (s, 6H); **^{13}C NMR** (101 MHz, Chloroform-*d*) δ 173.1, 170.6, 153.9, 135.0, 129.2, 129.2, 129.2, 127.5, 127.4, 121.0, 120.8, 117.3, 103.8, 74.7, 72.7, 65.2, 33.0, 27.1, 22.7. **HRMS** (ESI-TOF) *m/z* calculated for $C_{22}H_{22}O_4$ (M + Na)$^+$: 373.1416, found 373.1422.

2.2.2 The preparation of compound 5

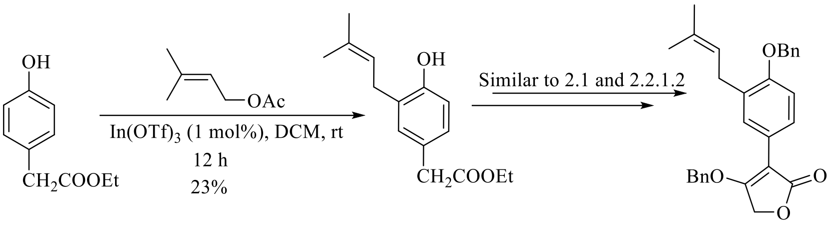

4a (30.0 mmol), prenyl acetate (30.0 mmol) and $In(OTf)_3$ (0.3 mmol) were stirred in CH_2Cl_2 (100 mL) at 25 °C for 12 h. At the end of reaction, the crude mixture was added to aqueous 1M NaOH (300 mL). The aqueous layer was extracted with Et_2O (3 × 50 mL). The combined organic layers were washed with aqueous 1M NaOH (3 × 100 mL), 1M HCl (3 × 100 mL) and saturated aqueous solution of NaCl (100 mL), dried with magnesium sulfate and concentrated under reduced pressure. Final products were purified by column chromatography on silica gel (PE/EA =10:1) to afford **5b**. The steps after **5b** are similar to those described above.

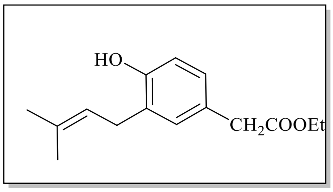

Ethyl 2-(4-hydroxy-3-(3-methylbut-2-en-1-yl) phenyl) acetate: Compound **5b** was isolated as a white solid (3.62 g, 23% yield). **^{1}H NMR** (600 MHz, Chloroform-*d*) δ 7.02 – 6.98 (m, 2H), 6.73 (dd, J = 8.8, 2.4 Hz, 1H), 5.31 (ddq, J = 8.7, 5.8, 1.5 Hz, 1H), 4.14 (q, J = 7.1 Hz, 2H), 3.51 (s, 2H), 3.33 (d, J = 7.2 Hz, 2H), 1.77 (t, J = 1.4 Hz, 6H), 1.25 (t, J = 7.1 Hz, 3H); **^{13}C NMR** (101 MHz, Chloroform-*d*) δ 171.7, 168.0, 153.4, 130.5, 128.4, 124.5, 121.2, 117.6, 74.4, 61.6, 61.2, 40.1, 32.9, 27.1, 22.6, 14.3; **HRMS** (ESI-TOF) *m/z* calculated for $C_{15}H_{20}O_3$ $(M + Na)^+$: 271.1310, found 271.1318.

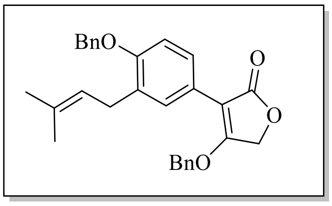

4-(benzyloxy)-3-(4-(benzyloxy)-3-(3-methylbut-2-en-1-yl) phenyl) furan-2(5*H*)-one: Compound **5** was isolated as a white solid (1.46 g, 30% yield). **^{1}H NMR** (400 MHz, Chloroform-*d*) δ 7.68 (d, J = 8.1 Hz, 2H), 7.50 – 7.27 (m, 10H), 6.91 (d, J = 8.3 Hz, 1H), 5.32 (ddq, J = 8.8, 5.9, 1.4 Hz, 1H), 5.15 (s, 2H), 5.10 (s, 2H), 4.80 (s, 2H), 3.39 (d, J = 7.4 Hz, 2H), 1.69 (d, J = 1.4 Hz, 3H), 1.63 (d, J = 1.3 Hz, 3H); **^{13}C NMR** (101 MHz, Chloroform-*d*) δ 173.0, 170.7, 156.1, 137.5, 134.9, 132.7, 130.5, 129.6, 129.2, 129.2, 128.7, 128.0, 127.5, 127.4, 126.9, 122.6, 121.9, 111.6, 103.8, 72.7, 70.1, 65.3, 29.0, 26.0, 18.0; **HRMS** (ESI-TOF) *m/z* calculated for $C_{29}H_{28}O_4$ $(M + Na)^+$:

463.1885, found 463.1880.

2.3 The preparation of compound 6

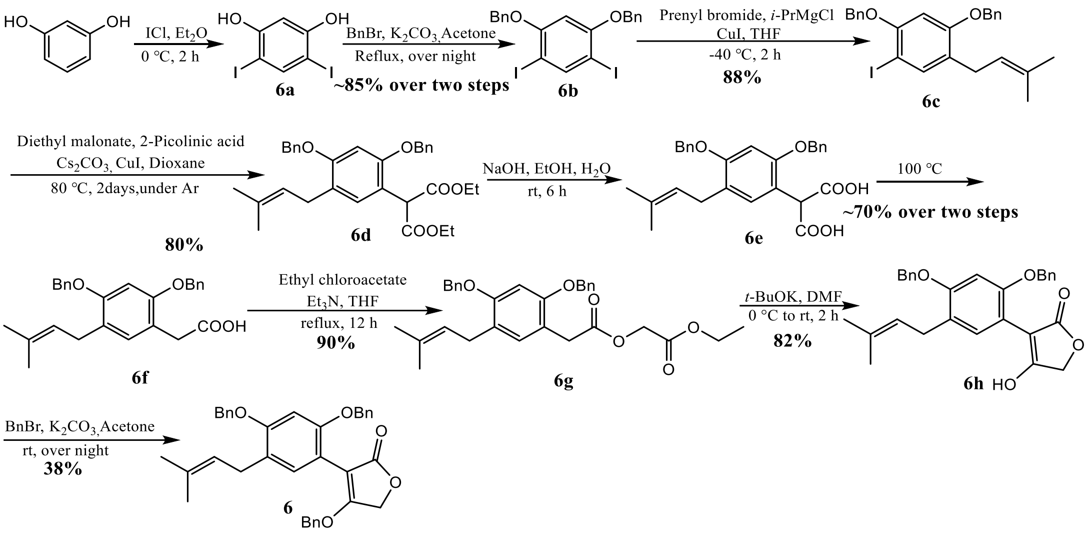

2.3.1 The preparation of compound 6b8

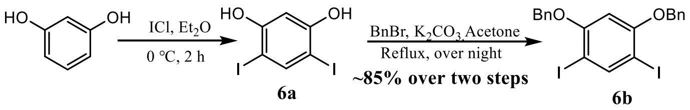

To iodine monochloride (10.46 mL, 200 mmol) in 50 mL of dry ether, resorcinol (10 g, 90.6 mmol) in 100 mL of dry ether was added dropwise at 0 °C. The mixture was stirred at room temperature for 2 hours. After cooling to 0 °C, excess of $Na_2S_2O_3$ was added slowly and stirred for 30 min. The reaction mixture was extracted with diethyl ether and deionized water one time, and then dried over anhydrous Na_2SO_4. After removing diethyl ether in a rotary evaporator, the resulting compound was washed with deionized water. The resulting precipitate was extracted with ethyl acetate and deionzed water once, and then dried over anhydrous Na_2SO_4. After removing ethyl acetate in a rotary evaporator, 30g pale red solid was yielded. Compound **6a** (30 g, 83 mmol), benzyl bromide (24.6 mL, 207.5 mmol) and potassium carbonate (28.7 g, 207.5 mmol) were dissolved in 500 mL of dry acetone. The reaction mixture was heated to reflux for 12 h under N_2 atmosphere. The solvent was removed by a rotary evaporator. The resulting mixture was extracted with dichloromethane and deionized water three times, and then dried over anhydrous Na_2SO_4. After removing dichloromethane in a rotary evaporator, the resulting compound was simply purified by a silica gel column chromatography using PE/EA = 4:1 as the eluent, to yield 41.7 g (85%) of a white solid. Compound **6b** is a known compound. [8]

2.3.2 The preparation of compound 6d

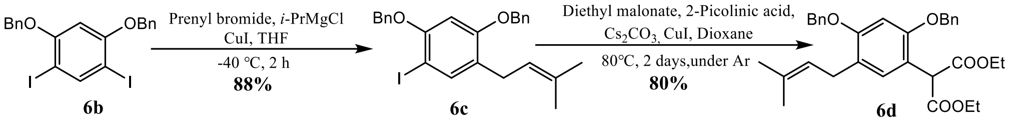

At –40 °C, a solution of 2 M *i*-PrMgCl in THF (33.3 mmol, 1.0 equiv) was added to a solution of 2,4-diiodoanisole (12.0 g, 33.3 mmol) in THF (120 mL) dropwise. The mixture was stirred at –40 °C for another 30 min. CuI (1.27 g, 6.66 mmol, 20 mol-%) was added and the mixture was allowed to reach –20 °C. After stirring for 30 min, 1-bromo-3-methylbut-2-ene (3.85 mL, 33.3 mmol, 1.0 equiv) was added dropwise. The mixture was stirred at –20 °C for 1 h. The mixture was quenched with sat. aq NH_4Cl (150 mL). The aqueous layer was extracted with Et_2O (3 × 50 mL). The combined organic layers were washed with brine (50 mL) and dried over Na_2SO_4. The solvent was removed in vacuum. The silica gel chromatography was used to simply purify the crude product to yield 8.88g colorless oil (88%).[9]

Cesium carbonate (3.68 g, 11.4 mmol) was added to a solution of **6c** (1.83 g, 3.78 mmol) in dioxane (20.00 mL). Copper iodide (35.0 mg, 5 mol%) and picolinic acid (46.0 mg, 10 mol%) were added in one portion. The solution was stirred at room temperature for 5 min and diethyl malonate (1.20 g, 7.56 mmol) was added in one portion and the solution was heated at reflux for 48 h. On completion the reaction was filtered through a short silica pad, the silica was washed with DCM (3 × 25 mL). The solution was dried using $MgSO_4$, filtered and concentrated in vacuo to give a viscous oil. Column chromatography, eluting with 80:20 (PE/EA), gave **6d** (1.38 g, 2.67 mmol, 71%) as a white solid. [10]

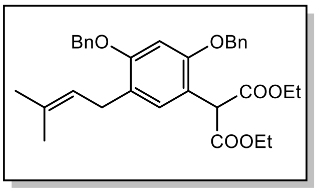

Diethyl 2-(2,4-bis(benzyloxy)-5-(3-methylbut-2-en-1-yl) phenyl) malonate: Compound **6d** was isolated as a yellow solid (0.79g, 71% yield). **^{1}H NMR** (400 MHz, Chloroform-*d*) δ 7.41 – 7.30 (m, 10H), 7.08 (s, 1H), 6.52 (s, 1H), 5.28 (t, J = 7.4 Hz, 1H), 5.05 (s, 1H), 5.03 – 4.99 (m, 4H), 4.19 (q, J = 7.1 Hz, 4H), 3.30 (d, J = 7.3 Hz, 2H), 1.70 (s, 3H), 1.63 (s, 3H), 1.24 (t, J = 7.1 Hz, 6H); **^{13}C NMR** (151 MHz, Chloroform-*d*) δ 168.9, 156.9, 155.1, 137.1, 136.9, 132.2, 130.1, 128.5, 128.5, 127.9, 127.8, 127.7, 127.2, 127.2, 122.9, 122.8, 114.1, 98.1, 70.7, 70.2, 61.7, 61.5, 51.3, 28.1, 25.8, 17.8, 14.0; **HRMS** (ESI-TOF) *m/z* calculated for $C_{32}H_{36}O_6$ $(M + Na)^+$: 539.2410, found 539.2406.

2.3.3 The preparation of compound 6f

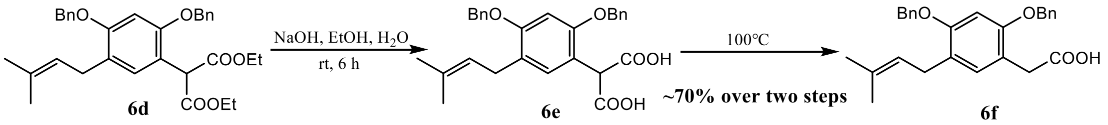

The aqueous NaOH (1.60 g, 40 mmol, 4.0 equiv.) solved in H_2O (15 mL) was slowly added to the EtOH (40 mL) solution of the compound **6d** (5.16 g, 10 mmol). The solution was stirred at room temperature for 5 h. The reaction mixture was cooled down to 0 °C, acidized with HCl (3.0 M) until pH=1, and extracted with diethyl ether (3 × 50 mL). The combined organic phase was dried over anhydrous Na_2SO_4. After removing the solvent, the crude diacid was used for the next step without further purification. After the diacid is ground into powder, it is placed in a round-bottom flask, heated to 100 °C for 2 h, and the reaction is monitored by TLC. The resulting compound **6f** was purified by a silica gel column chromatography using PE/EA = 4:1 as the eluent, to yield 2.91 g (70%) of a white solid.

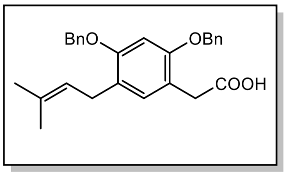

2-(2,4-bis(benzyloxy)-5-(3-methylbut-2-en-1-yl) phenyl) acetic acid: Compound **6f** was isolated as a white solid (2.91g, 70% yield). **^{1}H NMR** (400 MHz, DMSO-*d*$_6$) δ 7.44 – 7.28 (m, 10H), 6.90 (s, 1H), 6.80 (s, 1H), 5.23 – 5.15 (m, 1H), 5.09 (d, J = 4.6 Hz, 4H), 3.43 (s, 2H), 3.18 (d, J = 7.3 Hz, 2H), 1.64 (s, 3H), 1.59 (s, 3H); **^{13}C NMR** (101 MHz, DMSO-*d*$_6$) δ 173.5, 156.0, 155.7, 137.8, 131.9, 131.3, 128.9, 128.9, 128.2, 128.1, 128.0, 127.6, 123.7, 121.5, 116.2, 99.1, 70.1, 70.0, 35.6, 28.2, 26.0, 18.1; **HRMS** (ESI-TOF) *m/z* calculated for $C_{27}H_{28}O_4$ $(M + Na)^+$: 439.1885, found 439.1888.

2.3.4 The preparation compound 6

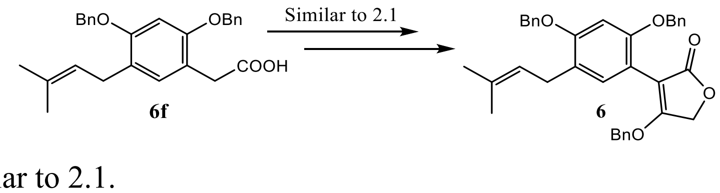

Steps are similar to 2.1.

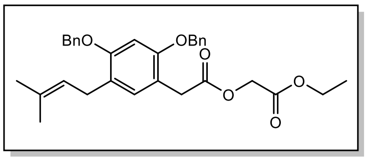

2-ethoxy-2-oxoethyl 2-(2,4-bis(benzyloxy)-5-(3-methyl but-2-en-1-yl) phenyl) acetate: Compound **6g** was isolated as a yellow oil (3g, 90% yield). **^{1}H NMR** (400 MHz, Chloroform-*d*) δ 7.44 – 7.25 (m, 10H), 6.99 (s, 1H), 6.52 (s, 1H), 5.28 (tt, J = 7.3,

1.5 Hz, 1H), 5.01 (d, J = 3.6 Hz, 4H), 4.54 (s, 2H), 4.19 (q, J = 7.1 Hz, 2H), 3.71 (s, 2H), 3.29 (d, J = 7.3 Hz, 2H), 1.72 (d, J = 1.5 Hz, 3H), 1.65 (s, 3H), 1.24 (t, J = 7.1 Hz, 3H); **^{13}C NMR** (101 MHz, Chloroform-*d*) δ 171.8, 168.1, 156.6, 155.7, 137.5, 137.4, 132.2, 131.9, 128.7, 128.7, 128.0, 128.0, 127.4, 127.3, 123.1, 122.9, 114.9, 98.5, 70.7, 70.5, 61.5, 61.1, 35.2, 28.2, 26.0, 18.0, 14.3. **HRMS** (ESI-TOF) *m/z* calculated for $C_{31}H_{34}O_6$ (M + Na)$^+$: 525.2253, found 525.2258.

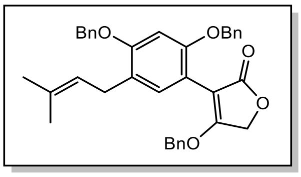

4-(benzyloxy)-3-(2,4-bis(benzyloxy)-5-(3-methylbut-2-en-1-yl) phenyl) furan-2(5*H*)-one: Compound **6** was isolated as a yellow oil (2.3g, 32% yield). **^{1}H NMR** (400 MHz, Chloroform-*d*) δ 7.44 – 7.23 (m, 13H), 7.15 – 7.07 (m, 2H), 7.00 (s, 1H), 6.55 (s, 1H), 5.32 – 5.24 (m, 1H), 5.05 – 4.96 (m, 6H), 4.68 (s, 2H), 3.30 (d, J = 7.3 Hz, 2H), 1.72 (d, J = 1.5 Hz, 3H), 1.64 (s, 3H); **^{13}C NMR** (151 MHz, Chloroform-*d*) δ 173.9, 171.7, 157.6, 156.0, 137.1, 137.0, 135.1, 132.4, 132.4, 128.7, 128.6, 128.6, 127.9, 127.8, 127.7, 127.2, 127.1, 123.1, 122.6, 110.9, 100.9, 98.8, 72.8, 71.0, 70.2, 67.0, 28.0, 25.8, 17.9, 17.8; **HRMS** (ESI-TOF) *m/z* calculated for $C_{36}H_{34}O_5$ (M + Na)$^+$: 569.2304, found 569.2342.

3 Synthesis of the precursor for aspulvinone O and B

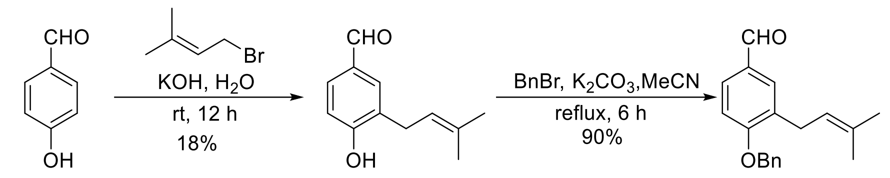

6i **6j**

KOH (5.6 g, 0.1 mol), *p*-Hydroxybenzaldehyde (12.3 g, 100 mmol) and pure water (120 mL) were mixed. Then, bromoisopentene (12.5 g, 120 mmol) was added dropwise with stirring at room temperature. The reaction was monitored with TLC. After the reaction was completed, the pH was adjusted to 3 with 3 mol/L hydrochloric acid, and then the reaction mixture was extracted with ethyl acetate (EA, 3 × 100 mL). The organic phases were combined, washed with saturated sodium carbonate solution and saturated brine, dried over anhydrous Na_2SO_4, and concentrated under reduced pressure to obtain a yellow oil, which was then subjected to a silica gel column chromatography with PE/EA (8:1) as the mobile phase to obtain 3.42 g of **6i** in 18% yield.
In a round bottom flask, **6i** (3.4 g, 17.9 mmol), K_2CO_3 (3.0 g, 21.5 mmol), benzyl bromide (3.7 g, 21.5 mol), and 20 mL of acetone were mixed, refluxed for 2 h, and filtered off. The filtrate was concentrated under reduced pressure. The residue was then purified by silica gel column chromatography with PE/EA (10:1) as the mobile phase to obtain 4.51 g of a colorless oil, with a yield of 90%. Compound **6j** is a known compound.[11]

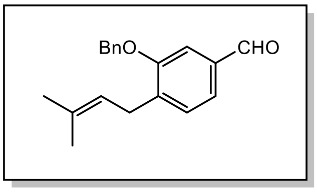

3-(benzyloxy)-4-(3-methylbut-2-en-1-yl) benzaldehyde: Compound **6j** was isolated as a colorless oil (4.51g, 90% yield). **^{1}H NMR** (400 MHz, Chloroform-*d*) δ 9.86 (s, 1H), 7.70 (d, J = 7.8 Hz, 2H), 7.47 – 7.30 (m, 5H), 7.00 (d, J = 8.2 Hz, 1H), 5.32 (ddq, J = 8.9, 5.8, 1.5 Hz, 1H), 5.18 (s, 2H), 3.41 (d, J = 7.4 Hz, 2H), 1.75 (s, 3H), 1.66 (s, 3H).

4 Synthesis of the aspulvinone derivatives

4.1 general procedures

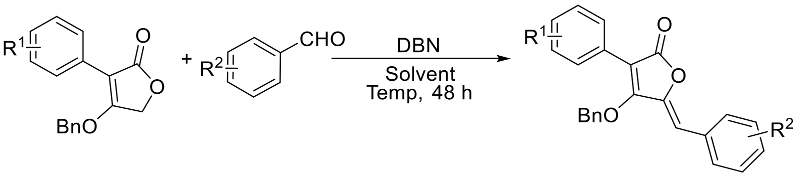

An oven dried glass bottle, which contained a stirring bar, was charged with the tetronic acid (50 mg), the aldehyde (3 eq) and dry solvent (1 ml). Then, the DBN (1 eq) was added dropwise via syringe into the bottle, and the reaction was run on the room temperature (30 °C) in MeCN or on the 60 °C in MeOH/EtOH for 48 h and was monitored by TLC (PE/EA= 2:1). At 365 nm on TLC plate, the product was appeared as a yellow-green fluorescent dot typically. After the reaction, the silica gel (100-200 mesh) and reaction solution were mixed, and the solvent was evaporated by the rotary evaporator. The crude product was purified by column chromatography with petroleum ether and ethyl acetate (20:1) to gain the target products in moderate to good yields.

4.2 Products in MeOH condition

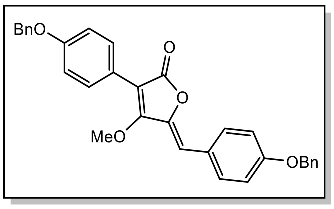

(*Z*)-5-(4-(benzyloxy) benzylidene)-3-(4-(benzyloxy) phenyl) -4-methoxyfuran-2(5*H*)-one: Compound **12** was isolated as a yellow solid (44.8 g, 36% yield). **^{1}H NMR** (600 MHz, Chloroform-*d*) δ 7.77 – 7.72 (m, 2H), 7.49 – 7.30 (m, 12H), 7.05 – 6.96 (m, 4H), 6.24 (s, 1H), 5.09 (d, J = 5.3 Hz, 4H), 3.85 (s, 3H); **^{13}C NMR** (101 MHz, Chloroform-*d*) δ 169.8, 163.8, 159.8, 159.4, 141.7, 137.2, 137.1, 132.7, 132.2, 131.8, 131.4, 129.2, 128.7, 128.6, 128.6, 128.0, 128.0, 126.4, 122.2, 115.7, 115.3, 115.1, 108.2, 105.7, 70.6, 70.6, 61.2; **HRMS** (ESI-TOF) *m/z* calculated for $C_{32}H_{26}O_5$ (M + Na) $^{+}$: 513.1678, found 513.1676.

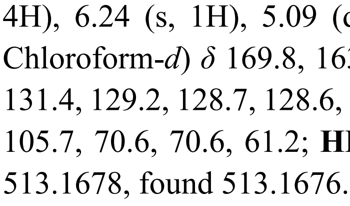

(Z)-5-(4-(benzyloxy)benzylidene)-3-(4-bromophenyl)-4-methoxyfuran-2(5*H*)-one ：

Compound **13** was isolated as a yellow solid (46mg, 69% yield). **^{1}H NMR** (600 MHz,

Chloroform-*d*) *δ* 7.78 – 7.72 (m, 2H), 7.58 – 7.51 (m, 2H), 7.46 – 7.36 (m, 6H), 7.36 – 7.31 (m, 1H), 7.03 – 6.95 (m, 2H), 6.29 (s, 1H), 5.10 (s, 2H), 3.85 (s, 3H); **^{13}C NMR** (151 MHz, Chloroform-*d*) *δ* 168.6, 164.1, 159.5, 140.8, 136.5, 132.3, 131.6, 131.6, 128.7, 128.3, 128.1, 127.5, 125.6, 122.7, 115.2, 108.7, 104.1, 70.1, 61.0, 60.8; **HRMS** (ESI-TOF) *m/z* calculated for $C_{25}H_{19}BrO_4$ (M + Na)$^+$: 485.0364, found 485.0369.

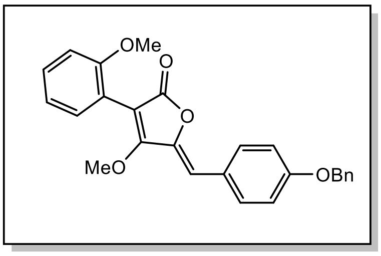

(*Z*)-5-(4-(benzyloxy) benzylidene)-4-methoxy-3-(2-methoxyphenyl) furan-2(5*H*)-one: Compound **14** was isolated as a yellow solid (25.9mg, 37% yield). **^{1}H NMR** (400 MHz, Chloroform-*d*) *δ* 7.74 (d, *J* = 8.8 Hz, 2H), 7.46 – 7.29 (m, 9H), 6.99 (d, *J* = 8.8 Hz, 2H), 6.24 (s, 1H), 5.10 (s, 2H), 3.84 (s, 3H), 3.76 (s, 3H); **^{13}C NMR** (151 MHz, Chloroform-*d*) *δ* 169.5, 164.5, 159.4, 158.0, 141.5, 136.8, 132.2, 130.4, 128.8, 128.8, 127.9, 127.7, 127.2, 120.8, 120.8, 115.3, 111.1, 107.4, 100.0, 70.2, 59.3, 55.8; **HRMS** (ESI-TOF) *m/z* calculated for $C_{26}H_{22}O_5$ (M + Na)$^+$: 437.1365, found 437.1358.

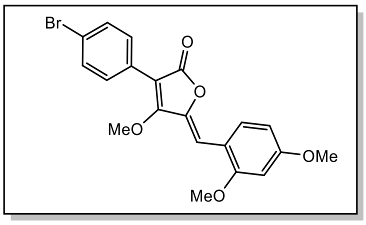

(*Z*)-3-(4-bromophenyl)-5-(2,4-dimethoxybenzylidene)-4-methoxyfuran-2(5*H*)-one: Compound **15** was isolated as a yellow solid (39mg, 64% yield). **^{1}H NMR** (600 MHz, Chloroform-*d*) *δ* 8.16 (d, *J* = 8.7 Hz, 1H), 7.58 – 7.52 (m, 2H), 7.48 – 7.40 (m, 2H), 6.81 (s, 1H), 6.56 (dd, *J* = 8.7, 2.4 Hz, 1H), 6.45 (d, *J* = 2.5 Hz, 1H), 3.92 – 3.84 (m, 9H); **^{13}C NMR** (101 MHz, Chloroform-*d*) *δ* 169.0, 164.7, 162.2, 159.2, 140.7, 133.0, 131.7, 131.7, 128.8, 122.7, 114.9, 105.7, 104.1, 103.1, 98.4, 61.2, 55.9, 55.7; **HRMS** (ESI-TOF) *m/z* calculated for $C_{20}H_{14}BrO_5$ (M + Na)$^+$: 439.0157, found 439.0149.

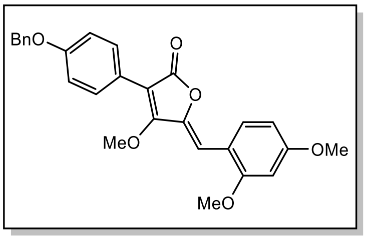

(*Z*)-3-(4-(benzyloxy)phenyl)-5-(2,4-dimethoxybenzylidene)-4-methoxyfuran-2(5H)-one: Compound **16** was isolated as a yellow solid (35mg, 59% yield). **^{1}H NMR** (400 MHz, Chloroform-*d*) *δ* 8.16 (d, J = 8.8 Hz, 1H), 7.43 – 7.32 (m, 7H), 6.92 (d, J = 8.8 Hz, 2H), 6.75 (s, 1H), 6.55 (dd, J = 8.8, 1.5 Hz, 1H), 6.45 (d, J = 1.5 Hz, 1H), 5.09 (s, 2H), 3.88 – 3.84 (m, 9H); **^{13}C NMR** (101 MHz, Chloroform-*d*) *δ* 169.7, 163.9, 161.9, 159.1, 141.1, 137.0, 132.9, 132.2, 131.4, 131.0, 128.9, 128.3, 127.7, 115.1, 115.0, 111.1,

105.6, 102.1, 98.4, 70.3, 60.9, 55.9, 55.7; **HRMS** (ESI-TOF) *m/z* calculated for $C_{27}H_{24}O_6$ (M + Na) $^+$: 467.1471, found 467.1479.

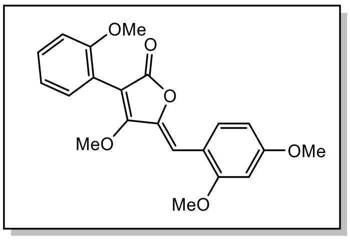

(*Z*)-5-(2,4-dimethoxybenzylidene)-4-methoxy-3-(2-methoxyphenyl) furan-2(5*H*)-one: Compound **17** was isolated as a yellow solid (45mg, 72% yield). **^{1}H NMR** (400 MHz, Chloroform-*d*) δ 8.15 (d, J = 8.7 Hz, 1H), 7.38 – 7.26 (m, 2H), 6.99 (t, J = 7.5 Hz, 1H), 6.91 (d, J = 8.2 Hz, 1H), 6.72 (s, 1H), 6.52 (dd, J = 8.8, 2.5 Hz, 1H), 6.43 (d, J = 2.4 Hz, 1H), 3.89 – 3.79 (m, 9H), 3.74 (s, 3H); **^{13}C NMR** (101 MHz, Chloroform-*d*) δ 169.7, 164.8, 161.7, 159.0, 158.0, 141.2, 132.8, 132.5, 130.3, 120.7, 119.1, 115.1, 111.1, 105.5, 101.5, 99.6, 98.3, 59.2, 55.8, 55.7, 55.7; **HRMS** (ESI-TOF) *m/z* calculated for $C_{21}H_{20}O_6$ (M + Na) $^+$: 391.1158, found 391.1155.

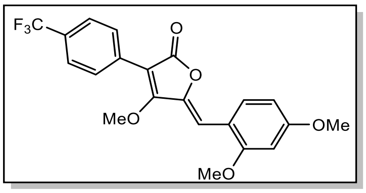

(*Z*)-5-(2,4-dimethoxybenzylidene)-4-methoxy-3-(4-(trifluoromethyl) phenyl) furan-2(5*H*)-one: Compound **18** was isolated as a white solid (7.9mg, 13% yield). **^{1}H NMR** (600 MHz, Chloroform-*d*) δ 8.17 (d, J = 8.7 Hz, 1H), 7.72 – 7.65 (m, 4H), 6.85 (s, 1H), 6.57 (dt, J = 8.9, 2.1 Hz, 1H), 6.46 (d, J = 2.1 Hz, 1H), 3.91 – 3.87 (m, 6H), 3.86 (s, 3H); **^{13}C NMR** (101 MHz, Chloroform-*d*) δ 169.2, 165.6, 162.7, 159.7, 140.8, 133.4, 130.8, 125.8, 125.8 (q, J = 3.9 Hz), 125.7, 125.7, 115.1, 106.1, 104.0, 98.7, 61.7, 56.2, 56.0; **^{19}F NMR** (376 MHz, Chloroform-*d*) δ -62.70. **HRMS** (ESI-TOF) *m/z* calculated for $C_{21}H_{17}F_3O_5$ (M + Na)$^+$: 429.0926, found 429.0933.

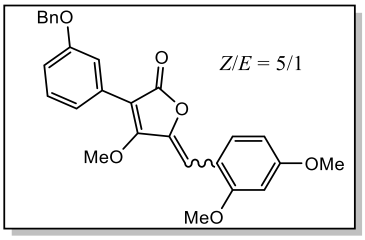

3-(3-(benzyloxy)phenyl)-5-(2,4-dimethoxybenzylidene)-4-methoxyfuran-2(5*H*)-one: Compound **19** was isolated as a yellow solid (25mg, 41% yield). **^{1}H NMR** (400 MHz, Chloroform-*d*) δ 8.14 (dd, J = 8.8, 2.3 Hz, 1.2H, *Z*/*E*), 7.44 – 7.41 (m, 2.4H, *Z*/*E*), 7.39 – 7.35 (m, 2.4H, *Z*/*E*), 7.33 – 7.29 (m, 2.4H, *Z*/*E*), 7.15 – 7.08 (m, 2.4H, *Z*/*E*), 6.97 (ddd, J = 8.3, 2.5, 1.1 Hz, 1.2H, *Z*/*E*), 6.79 (s, 0.2H, *E*), 6.75 (s, 1H, *Z*), 6.54 (dd, J = 8.8, 2.4 Hz, 1.2H, *Z*/*E*), 6.43 (d, J = 2.5 Hz, 1.2H, *Z*/*E*), 5.08 (s, 2H, *Z*), 5.02 (s, 0.4H, *E*), 3.85 (s, 3.6H, *Z*/*E*), 3.83 (d, J = 0.9 Hz, 3.6H, *Z*/*E*), 3.77 (s, 3.6H, *Z*/*E*); **HRMS** (ESI-TOF) *m/z* calculated for $C_{27}H_{24}O_6$ (M + Na)$^+$: 467.1471, found 467.1465.

4.3 Products in EtOH condition

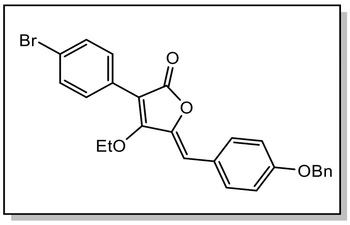

(*Z*)-5-(4-(benzyloxy)benzylidene)-3-(4-bromophenyl)-4-ethoxyfuran-2(5*H*)-one: Compound **20** was isolated as a yellow solid (44mg, 64% yield). **^{1}H NMR** (400 MHz, Chloroform-*d*) δ 7.80 – 7.72 (m, 2H), 7.55 (d, J = 8.5 Hz, 2H), 7.43 (ddd, J = 7.4, 3.8, 1.8 Hz, 5H), 7.41 – 7.39 (m, 1H), 7.36 – 7.33 (m, 1H), 7.00 (d, J = 8.9 Hz, 2H), 6.29 (s, 1H), 5.11 (s, 2H), 4.08 (q, J = 7.0 Hz, 2H), 1.33 (t, J = 7.0 Hz, 3H); **^{13}C NMR** (101 MHz, Chloroform-*d*) δ 169.2, 164.0, 160.0, 141.8, 137.1, 132.8, 132.1, 131.9, 129.2, 128.7, 128.0, 126.2, 123.2, 115.8, 115.0, 109.0, 105.0, 70.6, 70.2, 15.7; **HRMS** (ESI-TOF) *m/z* calculated for $C_{26}H_{21}BrO_4$ (M + Na)$^+$: 499.0521, found 499.0514.

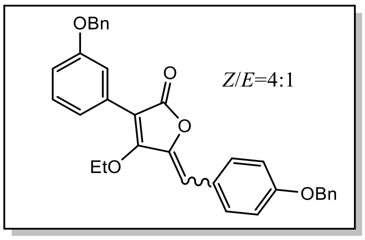

5-(4-(benzyloxy) benzylidene)-3-(3-(benzyloxy) phenyl) -4-ethoxyfuran-2(5*H*)-one: Compound **21** was isolated as a yellow oil (20mg, 30% yield). **^{1}H NMR** (400 MHz, Chloroform-*d*) δ 7.84 (d, J = 8.8 Hz, 0.5H, *E*), 7.78 – 7.74 (m, 2H, *Z*), 7.45 – 7.42 (m, 5H, *Z*/*E*), 7.40 (dt, J = 6.9, 1.8 Hz, 5H, *Z*/*E*), 7.34 (s, 2.5H, *Z*/*E*), 7.14 – 7.08 (m, 2.5H, *Z*/*E*), 7.04 – 6.93 (m, 5H, *Z*/*E*), 6.26 (d, J = 3.8 Hz, 1.25H, *Z*/*E*), 5.10 (d, J = 1.9 Hz, 4H, *Z*), 5.06 – 5.04 (m, 1H, *E*), 4.02 (q, J = 7.0 Hz, 2H, *Z*), 3.82 (q, J = 7.0 Hz, 0.5H, *E*), 1.27 (t, J = 7.0 Hz, 3H, *Z*), 1.04 (t, J = 7.0 Hz, 0.75H, *E*); **HRMS** (ESI-TOF) *m/z* calculated for $C_{33}H_{28}O_5$ (M + Na)$^+$: 527.1834, found 527.1828

4.4 Products in MeCN condition

4.4.1 Natural aspulvinone precursor

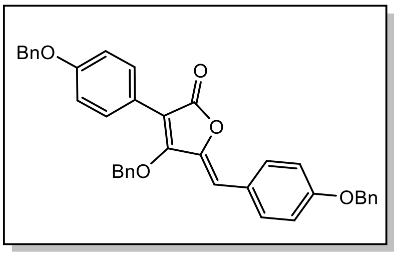

(*Z*)-4-(benzyloxy)-5-(4-(benzyloxy) benzylidene)-3-(4-(benzyloxy) phenyl) furan-2(5*H*)-one: Compound **22** was isolated as a yellow solid (49mg, 65% yield). **^{1}H NMR** (600 MHz, Chloroform-*d*) *δ* 7.76 – 7.71 (m, 2H), 7.53 – 7.49 (m, 2H), 7.48 – 7.38 (m, 8H), 7.35 (h, *J* = 3.9 Hz, 5H), 7.22 (dd, *J* = 6.6, 3.0 Hz, 2H), 7.06 – 7.02 (m, 2H), 7.01 – 6.97 (m, 2H), 6.24 (s, 1H), 5.11 (d, *J* = 8.1 Hz, 4H), 5.06 (s, 2H); **^{13}C NMR** (151 MHz, Chloroform-*d*) *δ* 169.2, 162.3, 159.3, 159.0, 141.6, 136.7, 136.6, 135.2, 132.2, 131.9, 131.2, 130.9, 128.8, 128.7, 128.4, 128.3, 128.2, 128.1, 127.9, 127.5, 127.5, 127.5, 125.8, 121.8, 115.2, 114.9, 114.9, 114.5, 107.9, 106.3, 74.4, 70.1, 70.1; **HRMS** (ESI-TOF) *m/z* calculated for $C_{38}H_{30}O_5$ (M + H)$^+$: 567.2166, found 567.2164.

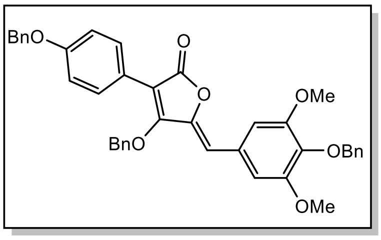

(*Z*)-4-(benzyloxy)-5-(4-(benzyloxy)-3,5-dimethoxy benzylidene)-3-(4-(benzyloxy) phenyl) furan-2(5*H*)-one: Compound **23** was isolated as a yellow solid (56mg, 66% yield). **^{1}H NMR** (600 MHz, Chloroform-*d*) *δ* 7.49 (dd, *J* = 8.4, 6.4 Hz, 4H), 7.45 (d, *J* = 7.1 Hz, 2H), 7.40 (t, *J* = 7.6 Hz, 2H), 7.37 – 7.25 (m, 8H), 7.21 (dd, *J* = 6.6, 3.1 Hz, 2H), 7.07 – 7.02 (m, 2H), 7.00 (s, 2H), 6.18 (s, 1H), 5.11 (s, 2H), 5.06 (s, 4H), 3.87 (s, 6H); **^{13}C NMR** (151 MHz, Chloroform-*d*) *δ* 168.9, 162.1, 159.1, 153.6, 142.4, 137.9, 137.6, 136.6, 135.1, 131.2, 130.7, 128.8, 128.7, 128.5, 128.5, 128.4, 128.4, 128.2, 128.2, 128.1, 128.0, 127.9, 127.5, 121.6, 114.9, 108.0, 107.8, 75.1, 74.5, 70.1, 56.3, 56.0. **HRMS** (ESI-TOF) *m/z* calculated for $C_{40}H_{34}O_7$ (M + Na)$^+$: 649.2196, found 649.2205.

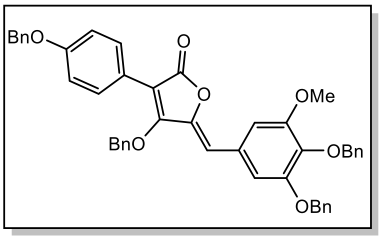

(*Z*)-4-(benzyloxy)-3-(4-(benzyloxy)phenyl)-5-(3,4-bis(benzyloxy)-5-methoxy benzylidene)furan-2(5*H*)-one: Compound **24** was isolated as a yellow solid (68mg,

72% yield). **^{1}H NMR** (600 MHz, Chloroform-*d*) δ 7.53 – 7.23 (m, 20H), 7.22 – 7.18 (m, 2H), 7.09 (d, J = 1.9 Hz, 1H), 7.06 – 7.02 (m, 2H), 6.99 (d, J = 1.9 Hz, 1H), 6.15 (s, 1H), 5.13 (s, 2H), 5.11 (s, 2H), 5.08 (s, 2H), 5.05 (s, 2H), 3.87 (s, 3H); **^{13}C NMR** (151 MHz, Chloroform-*d*) δ 168.8, 162.1, 159.0, 153.7, 152.7, 142.4, 138.6, 137.6, 136.9, 136.6, 135.1, 131.2, 128.8, 128.7, 128.5, 128.2, 128.1, 128.0, 127.9, 127.9, 127.7, 127.5, 121.6, 114.9, 109.7, 108.2, 107.9, 106.5, 75.1, 74.4, 71.2, 70.1, 56.3; **HRMS** (ESI-TOF) *m/z* calculated for $C_{46}H_{38}O_7$ $(M + Na)^+$: 725.2509, found 725.2514.

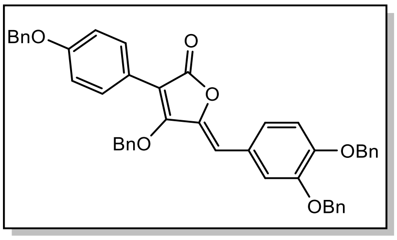

(*Z*)-4-(benzyloxy)-3-(4-(benzyloxy)phenyl)-5-(3,4-bis(benzyloxy)benzylidene) furan-2(5*H*)-one: Compound **25** was isolated as a yellow solid (51mg, 57% yield). **^{1}H NMR** (600 MHz, Chloroform-*d*) δ 7.54 – 7.27 (m, 21H), 7.23 (dd, J = 8.4, 2.1 Hz, 1H), 7.22 – 7.16 (m, 2H), 7.06 – 7.02 (m, 2H), 6.90 (d, J = 8.4 Hz, 1H), 6.15 (s, 1H), 5.20 (d, J = 9.0 Hz, 4H), 5.11 (s, 2H), 5.04 (s, 2H); **^{13}C NMR** (151 MHz, Chloroform-*d*) δ 169.0, 162.2, 159.0, 149.8, 148.8, 141.7, 137.0, 136.9, 136.7, 135.2, 131.1, 128.8, 128.7, 128.5, 128.5, 128.1, 127.9, 127.9, 127.8, 127.7, 127.5, 127.2, 126.4, 124.8, 121.8, 116.4, 114.9, 114.9, 114.3, 108.0, 106.4, 74.4, 71.3, 70.9, 70.1; **HRMS** (ESI-TOF) *m/z* calculated for $C_{45}H_{36}O_6$ $(M + Na)^+$: 695.2404, found 695.2410.

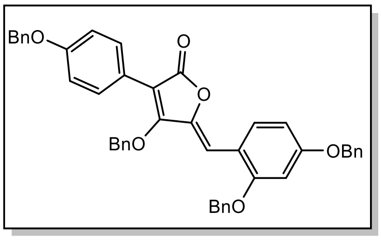

(*Z*)-4-(benzyloxy)-3-(4-(benzyloxy)phenyl)-5-(2,4-bis(benzyloxy)benzylidene) furan-2(5*H*)-one: Compound **26** was isolated as a yellow solid (60mg, 66% yield). **^{1}H NMR** (600 MHz, Chloroform-*d*) δ 8.18 (d, J = 8.7 Hz, 1H), 7.56 (d, J = 8.3 Hz, 2H), 7.47 – 7.32 (m, 16H), 7.30 (t, J = 7.4 Hz, 1H), 7.27 – 7.19 (m, 3H), 7.15 (s, 1H), 7.03 (d, J = 8.3 Hz, 2H), 6.88 (s, 1H), 6.65 (dd, J = 8.8, 2.4 Hz, 1H), 6.59 (d, J = 2.4 Hz, 1H), 5.11 (s, 2H), 5.08 (s, 4H), 5.03 (s, 2H); **^{13}C NMR** (151 MHz, Chloroform-*d*) δ 169.3, 162.6, 160.8, 158.9, 158.0, 141.4, 136.7, 136.6, 136.5, 135.3, 132.7, 130.9, 128.7, 128.6, 128.6, 128.6, 128.6, 128.2, 128.2, 128.1, 128.0, 127.7, 127.6, 127.5, 127.2, 122.0, 115.6, 114.9, 106.8, 106.5, 102.2, 100.4, 74.4, 70.4, 70.2, 70.1; **HRMS** (ESI-TOF) *m/z* calculated for $C_{45}H_{36}O_6$ $(M + Na)^+$: 695.2404, found 695.2411.

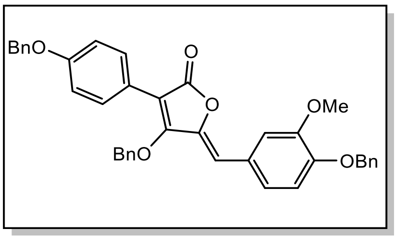

(*Z*)-4-(benzyloxy)-5-(4-(benzyloxy)-3-methoxybenzylidene)-3-(4-(benzyloxy) phenyl)furan-2(5*H*)-one: Compound **27** was isolated as a yellow solid (41mg, 51% yield). **^{1}H NMR** (600 MHz, Chloroform-*d*) δ 7.53 – 7.47 (m, 2H), 7.47 – 7.28 (m, 14H), 7.22 (ddd, *J* = 15.5, 7.2, 2.2 Hz, 3H), 7.06 – 7.01 (m, 2H), 6.87 (d, *J* = 8.4 Hz, 1H), 6.20 (s, 1H), 5.19 (s, 2H), 5.11 (s, 2H), 5.05 (s, 2H), 3.94 (s, 3H); **^{13}C NMR** (151 MHz, Chloroform-*d*) δ 169.0, 162.2, 159.0, 149.6, 149.0, 141.7, 136.7, 136.7, 135.2, 131.2, 128.8, 128.7, 128.6, 128.1, 128.0, 127.9, 127.5, 127.3, 126.3, 124.2, 121.8, 114.9, 113.5, 113.4, 108.1, 106.3, 74.4, 70.8, 70.1, 56.2; **HRMS** (ESI-TOF) *m/z* calculated for $C_{39}H_{32}O_6$ (M + Na)$^+$: 619.2091, found 619.2101.

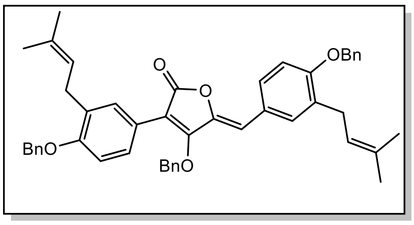

(*Z*)-4-(benzyloxy)-5-(4-(benzyloxy)-3-(3-methylbut-2-en-1-yl)benzylidene)-3-(4-(benzyloxy)-3-(3-methylbut-2-en-1-yl)phenyl)furan-2(5*H*)-one: Compound **28** was isolated as a yellow solid (19mg, 23% yield). **^{1}H NMR** (400 MHz, Chloroform-*d*) δ 7.44 – 7.31 (m, 14H), 7.23 – 7.18 (m, 5H), 6.82 (d, *J* = 7.0 Hz, 2H), 6.19 (s, 1H), 5.35 – 5.24 (m, 2H), 5.16 (s, 2H), 5.11 (s, 2H), 5.03 (s, 2H), 3.39 (d, *J* = 7.6 Hz, 2H), 3.36 (d, *J* = 7.6 Hz, 2H), 1.73 (s, 6H), 1.64 (s, 6H); **^{13}C NMR** (101 MHz, Chloroform-*d*) δ 167.8, 161.4, 151.4, 149.7, 136.4, 136.1, 133.8, 130.5, 130.4, 128.5, 128.5, 128.1, 127.9, 127.4, 127.4, 125.2, 122.5, 114.7, 106.9, 101.2, 70.5, 70.4, 70.3, 28.5, 28.4, 26.0, 18.1; **HRMS** (ESI-TOF) *m/z* calculated for $C_{48}H_{46}O_5$ (M + Na)$^+$: 725.3243, found 725.3248.

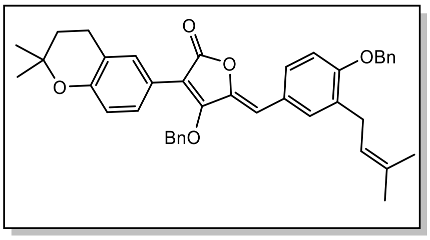

(*Z*)-4-(benzyloxy)-5-(4-(benzyloxy)-3-(3-methyl but-2-en-1-yl)benzylidene)-3-(2,2-dimethylchroman-6-yl)furan-2(5*H*)-one: Compound **29** was isolated as a yellow solid (24mg, 28% yield). **^{1}H NMR** (400 MHz, Chloroform-*d*) δ 7.44 – 7.30 (m, 11H), 7.22 (s, 1H), 7.20 (s, 2H), 6.89 (d, *J* = 8.6 Hz, 1H), 6.82 (d, *J* = 8.6 Hz, 1H), 6.19 (s, 1H), 5.30 (t, *J* = 7.6 Hz, 1H), 5.10 (s, 2H), 5.04 (s, 2H), 3.37 (d, *J* = 7.6 Hz, 2H), 2.76 (t, *J* = 7.0 Hz, 2H), 1.81 (t, *J* = 7.0 Hz, 2H), 1.73 (s, 3H), 1.66 (s, 3H), 1.34 (s, 6H); **^{13}C NMR** (101 MHz, Chloroform-*d*) δ 169.9, 162.6, 157.6, 155.0, 142.1, 137.5, 136.0, 133.4, 132.6, 131.4, 131.4, 131.1, 130.3, 129.5, 129.2, 129.1, 129.1, 129.1, 128.8, 128.7, 128.4, 127.8, 127.7, 126.2, 122.7, 121.5, 120.9, 117.8, 112.4, 111.6, 108.6, 107.2,, 75.3, 74.8, 70.5, 33.2, 30.2, 29.3, 27.5, 26.3, 23.0, 18.4; **HRMS** (ESI-TOF) *m/z* calculated for $C_{41}H_{40}O_5$ (M + Na)$^+$: 635.2773, found 635.2779.

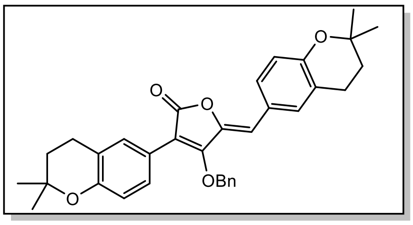

(*Z*)-4-(benzyloxy)-3-(2,2-dimethylchroman-6-yl)-5-((2,2-dimethylchroman-6-yl) methylene) furan-2(5*H*)-one： Compound **30** was isolated as a yellow oil (22mg, 30% yield). **¹H NMR** (400 MHz, Chloroform-*d*) δ 7.40 – 7.30 (m, 7H), 7.20 (d, *J* = 7.0 Hz, 2H), 6.82 (d, *J* = 7.0 Hz, 2H), 6.17 (s, 1H), 5.04 (s, 2H), 2.77 (td, *J* = 7.0, 4.0 Hz, 4H), 1.81 (td, *J* = 7.0, 4.0 Hz, 4H), 1.33 (d, *J* = 4.8 Hz, 12H); **¹³C NMR** (101 MHz, Chloroform-*d*) δ 169.7, 162.4, 155.3, 154.6, 141.4, 135.7, 132.1, 131.1, 130.5, 129.1, 128.8, 128.8, 128.6, 128.5, 128.0, 124.9, 121.6, 121.2, 120.6, 117.9, 117.5, 108.4, 106.7,75.2, 74.9, 74.5, 32.9, 27.2, 22.7; **HRMS** (ESI-TOF) *m/z* calculated for $C_{34}H_{34}O_5$ (M + Na)⁺: 545.2304, found 545.2298.

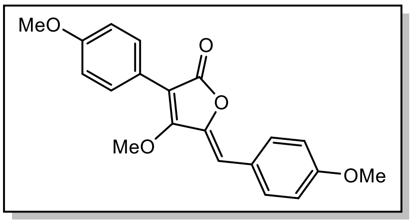

(*Z*)-4-methoxy-5-(4-methoxybenzylidene)-3-(4-methoxyphenyl)furan-2(5*H*)-one ： Compound **31** was isolated as a yellow solid (50mg, 65% yield). **¹H NMR** (400 MHz, Chloroform-*d*) δ 7.79 – 7.71 (m, 2H), 7.51 – 7.43 (m, 2H), 7.02 – 6.88 (m, 4H), 6.25 (s, 1H), 3.92 – 3.74 (m, 9H); **¹³C NMR** (101 MHz, Chloroform-*d*) δ 169.5, 163.5, 160.4, 159.9, 141.3, 132.3, 131.5, 125.8, 121.7, 114.5, 114.1, 107.9, 105.4, 60.9, 55.6, 55.5; **HRMS** (ESI-TOF) *m/z* calculated for $C_{20}H_{18}O_5$ (M + Na)⁺: 361.1046, found 361.1054.

4.4.2 Aspulvinone derivatives

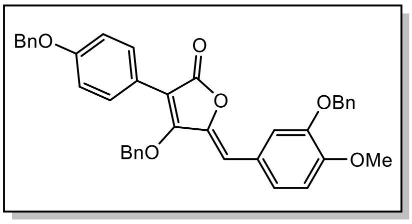

(*Z*)-4-(benzyloxy)-5-(3-(benzyloxy)-4-methoxybenzylidene)-3-(4-(benzyloxy) phenyl) furan-2(5*H*)-one: Compound **32** was isolated as a yellow solid (42mg, 53% yield). **¹H NMR** (400 MHz, Chloroform-*d*) δ 7.45 – 7.30 (m, 17H), 7.06 (d, *J* = 7.0 Hz, 1H), 6.96 (d, *J* = 7.0 Hz, 1H), 6.94 – 6.89 (m, 3H), 6.16 (s, 1H), 5.22 (s, 2H), 5.11 (s, 2H), 5.04 (s, 2H), 3.91 (s, 3H); **¹³C NMR** (101 MHz, Chloroform-*d*) δ 169.2, 162.4,

159.2, 149.0, 148.4, 141.8, 136.8, 136.8, 136.1, 131.1, 130.5, 130.3, 128.9, 128.5, 128.5, 128.1, 127.9, 127.7, 127.7, 127.6, 120.2, 115.1, 111.8, 111.1, 108.3, 106.6, 74.6, 71.3, 70.3, 56.2; **HRMS** (ESI-TOF) *m/z* calculated for $C_{39}H_{32}O_6$ (M + Na)$^+$: 619.2097, found 619.2099.

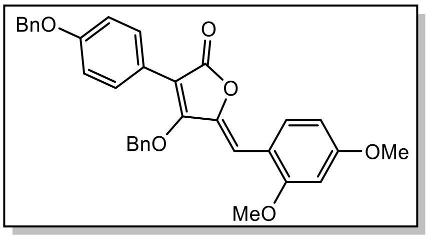

(*Z*)-4-(benzyloxy)-3-(4-(benzyloxy) phenyl)-5-(2,4-dimethoxybenzylidene) furan-2(5*H*)-one: Compound **33** was isolated as a yellow solid (46mg, 66% yield). **^{1}H NMR** (400 MHz, Chloroform-*d*) δ 8.15 (d, J = 8.7 Hz, 1H), 7.54 – 7.50 (m, 2H), 7.45 (d, J = 7.1 Hz, 2H), 7.40 (t, J = 7.7 Hz, 2H), 7.37 – 7.28 (m, 4H), 7.22 (dd, J = 6.7, 2.8 Hz, 2H), 7.05 – 7.00 (m, 2H), 6.78 (s, 1H), 6.55 (dd, J = 8.8, 2.4 Hz, 1H), 6.44 (dd, J = 7.1, 2.3 Hz, 1H), 5.10 (d, J = 11.6 Hz, 4H), 3.85 (s, 6H); **^{13}C NMR** (101 MHz, DMSO-d_6) δ 166.8, 163.7, 160.4, 159.2, 151.4, 149.7, 136.8, 136.1, 130.5, 130.3, 128.8, 128.8, 128.5, 128.1, 127.9, 127.7, 115.1, 114.8, 109.5, 108.0, 106.2, 99.2, 72.7, 70.0, 56.0, 55.6; **HRMS** (ESI-TOF) *m/z* calculated for $C_{33}H_{28}O_6$ (M + Na)$^+$: 543.1784, found 543.1789.

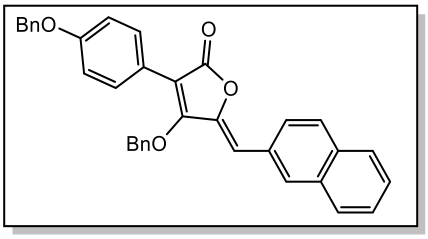

(*Z*)-4-(benzyloxy)-3-(4-(benzyloxy) phenyl)-5-(naphthalen-2-ylmethylene) furan-2(5*H*)-one: Compound **34** was isolated as a yellow solid (51mg, 74% yield). **^{1}H NMR** (600 MHz, Chloroform-*d*) δ 8.18 (s, 1H), 7.96 (dd, J = 8.6, 1.7 Hz, 1H), 7.89 – 7.85 (m, 1H), 7.82 (dd, J = 10.9, 6.8 Hz, 2H), 7.52 (d, J = 8.3 Hz, 2H), 7.52 – 7.45 (m, 3H), 7.41 (t, J = 7.5 Hz, 2H), 7.37 – 7.32 (m, 4H), 7.24 (dd, J = 8.1, 4.3 Hz, 3H), 7.06 (d, J = 8.4 Hz, 2H), 6.44 (s, 1H), 5.11 (d, J = 14.2 Hz, 4H); **^{13}C NMR** (151 MHz, Chloroform-*d*) δ 169.1, 162.2, 159.1, 143.2, 136.7, 135.1, 133.4, 133.2, 131.2, 130.6, 130.4, 128.9, 128.7, 128.7, 128.6, 128.4, 128.1, 128.0, 127.7, 127.5, 127.4, 127.0, 126.5, 121.6, 115.0, 114.9, 108.2, 106.8, 74.5, 70.1; **HRMS** (ESI-TOF) *m/z* calculated for $C_{35}H_{26}O_4$ (M + Na)$^+$: 533.1729, found 533.1735.

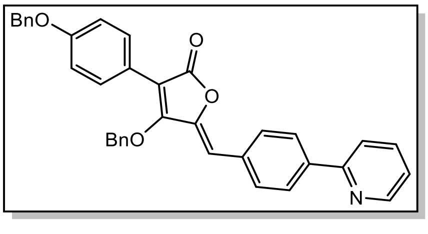

(*Z*)-4-(benzyloxy)-3-(4-(benzyloxy) phenyl)-5-(4-(pyridin-2-yl)benzylidene)furan-2(5*H*)-one: Compound **35** was isolated as a yellow solid (29mg, 41% yield). **^{1}H NMR**

(400 MHz, Chloroform-*d*) δ 8.69 (dt, *J* = 4.8, 1.4 Hz, 1H), 8.05 – 7.98 (m, 2H), 7.90 – 7.83 (m, 2H), 7.79 – 7.70 (m, 2H), 7.53 – 7.46 (m, 2H), 7.46 – 7.27 (m, 8H), 7.22 (ddt, *J* = 9.6, 4.6, 3.4 Hz, 3H), 7.08 – 6.99 (m, 2H), 6.31 (s, 1H), 5.08 (d, *J* = 13.7 Hz, 4H); **^{13}C NMR** (101 MHz, Chloroform-*d*) δ 169.2, 162.3, 159.3, 156.8, 150.0, 143.6, 139.6, 137.0, 136.9, 135.3, 133.6, 131.4, 131.1, 129.1, 128.9, 128.9, 128.3, 128.1, 127.7, 127.4, 122.6, 121.7, 120.8, 115.2, 107.7, 107.2, 74.7, 70.3; **HRMS** (ESI-TOF) *m/z* calculated for $C_{36}H_{27}NO_4$ (M + H)$^+$: 538.2018, found 538.2015.

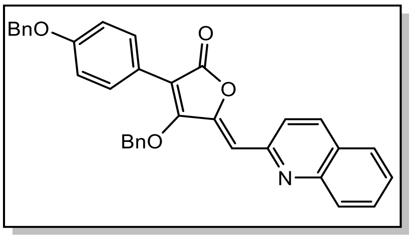

(*Z*)-4-(benzyloxy)-3-(4-(benzyloxy) phenyl)-5-(quinolin-2-ylmethylene)furan-2(5*H*)-one: Compound **36** was isolated as a yellow solid (45mg, 66% yield). **^{1}H NMR** (600 MHz, Chloroform-*d*) δ 8.25 (d, *J* = 8.7 Hz, 1H), 8.08 (d, *J* = 8.7 Hz, 1H), 7.95 (d, *J* = 8.5 Hz, 1H), 7.71 (d, *J* = 8.1 Hz, 1H), 7.62 (t, *J* = 7.7 Hz, 1H), 7.49 – 7.28 (m, 8H), 7.26 (s, 2H), 7.12 (dd, *J* = 6.3, 3.0 Hz, 3H), 6.96 (d, *J* = 8.2 Hz, 2H), 6.67 (s, 1H), 5.02 (d, *J* = 4.3 Hz, 4H); **^{13}C NMR** (151 MHz, Chloroform-*d*) δ 167.5, 161.0, 158.3, 151.8, 147.2, 145.2, 135.5, 135.4, 133.7, 130.3, 128.8, 128.3, 127.9, 127.6, 127.1, 127.0, 126.5, 126.5, 126.2, 126.1, 121.8, 120.1, 113.9, 107.8, 106.4, 73.6, 69.1, 69.1; **HRMS** (ESI-TOF) *m/z* calculated for $C_{34}H_{25}NO_4$ (M + Na)$^+$: 534.1681, found 534.1683.

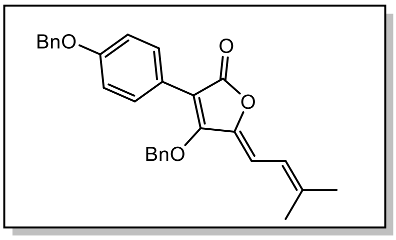

(*Z*)-4-(benzyloxy)-3-(4-(benzyloxy) phenyl)-5-(3-methylbut-2-en-1-ylidene)furan-2(5*H*)-one: Compound **37** was isolated as a yellow solid (19mg, 32% yield). **^{1}H NMR** (600 MHz, Chloroform-*d*) δ 7.56 – 7.50 (m, 2H), 7.44 (d, *J* = 7.6 Hz, 2H), 7.42 – 7.37 (m, 2H), 7.39 – 7.30 (m, 4H), 7.24 – 7.17 (m, 2H), 7.05 – 6.98 (m, 2H), 6.35 (d, *J* = 12.0 Hz, 1H), 6.19 (dd, *J* = 12.0, 1.9 Hz, 1H), 5.10 (d, *J* = 1.8 Hz, 2H), 5.04 (d, *J* = 1.8 Hz, 2H), 1.90 (s, 3H), 1.83 (s, 3H). **^{13}C NMR** (151 MHz, Chloroform-*d*) δ 168.6, 161.4, 158.9, 143.5, 141.7, 136.7, 136.7, 135.3, 130.9, 128.7, 128.7, 128.6, 128.1, 128.1, 127.9, 127.5, 122.0, 122.0, 118.3, 114.9, 107.6, 106.1, 74.4, 70.1, 26.7, 18.9. **HRMS** (ESI-TOF) *m/z* calculated for $C_{29}H_{26}O_4$ (M + Na)$^+$: 461.1729, found 461.1733.

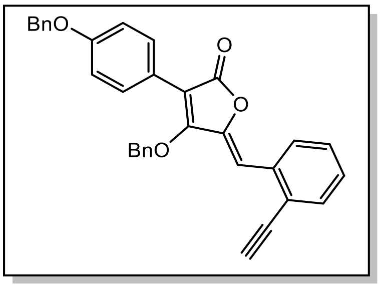

(*Z*)-4-(benzyloxy)-3-(4-(benzyloxy)phenyl)-5-(2-ethynylbenzylidene)furan-2(5*H*)-one: Compound **38** was isolated as a yellow solid (49mg, 75% yield). **^{1}H NMR** (600 MHz, Chloroform-*d*) δ 8.24 (dd, *J* = 8.1, 1.2 Hz, 1H), 7.58 – 7.50 (m, 3H), 7.46 (d, *J* = 7.1 Hz, 2H), 7.43 – 7.37 (m, 3H), 7.35 – 7.29 (m, 3H), 7.27 (td, *J* = 7.6, 1.2 Hz, 1H), 7.26 – 7.21 (m, 2H), 7.08 – 7.02 (m, 2H), 6.98 (s, 1H), 5.12 (d, *J* = 4.8 Hz, 4H), 3.35 (s, 1H). **^{13}C NMR** (151 MHz, Chloroform-*d*) δ 167.7, 161.1, 158.1, 142.9, 135.6, 134.1, 133.6, 131.9, 130.0, 129.2, 128.3, 127.7, 127.6, 127.6, 127.3, 127.1, 126.7, 126.5, 121.5, 120.3, 113.9, 106.7, 104.2, 81.6, 80.7, 73.4, 69.1. **HRMS** (ESI-TOF) *m/z* calculated for $C_{33}H_{24}O_4$ (M + Na)$^+$: 507.1572, found 507.1568.

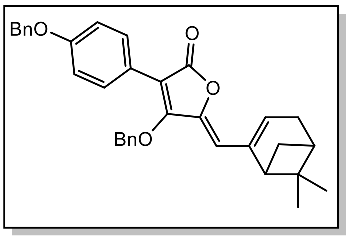

(*Z*)-4-(benzyloxy)-3-(4-(benzyloxy) phenyl)-5-((6,6-dimethylbicyclo[3.1.1]hept-2-en-2-yl)methylene)furan-2(5*H*)-one: Compound **39** was isolated as a yellow solid (14mg, 21% yield). **^{1}H NMR** (400 MHz, Chloroform-*d*) δ 7.46 – 7.30 (m, 10H), 7.16 (dd, *J* = 6.8, 2.9 Hz, 2H), 7.00 (d, *J* = 8.8 Hz, 2H), 6.09 (s, 1H), 5.83 (s, 1H), 5.08 (s, 2H), 4.97 (s, 2H), 3.07 – 3.00 (m, 1H), 2.44 (ddt, *J* = 13.3, 9.9, 3.9 Hz, 3H), 2.08 (d, *J* = 4.0 Hz, 1H), 1.32 (s, 3H), 1.16 (d, *J* = 9.0 Hz, 1H), 0.81 (s, 3H). **^{13}C NMR** (101 MHz, Chloroform-*d*) δ 162.8, 159.4, 143.7, 140.9, 137.2, 133.6, 131.7, 131.6, 129.2, 129.2, 129.1, 129.0, 128.7, 128.6, 128.4, 128.0, 117.8, 115.4, 115.3, 110.2, 74.8, 70.6, 44.4, 40.7, 38.2, 33.5, 31.9, 26.7, 21.6. **HRMS** (ESI-TOF) *m/z* calculated for $C_{33}H_{24}O_4$ (M + Na)$^+$: 527.2198, found 527.2183.

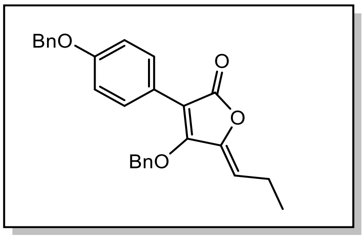

(*Z*)-4-(benzyloxy)-3-(4-(benzyloxy)phenyl)-5-propylidenefuran-2(5*H*)-one: Compound **40** was isolated as a yellow solid (23mg, 41% yield). **^{1}H NMR** (400 MHz, Chloroform-*d*) δ 7.46 – 7.28 (m, 10H), 7.18 – 7.12 (m, 2H), 7.04 – 6.95 (m, 2H), 5.48 (t, *J* = 7.9 Hz, 1H), 5.08 (s, 2H), 4.99 (s, 2H), 2.36 (p, *J* = 7.6 Hz, 2H), 1.06 (t, *J* = 7.5 Hz, 3H). **^{13}C NMR** (101 MHz, Chloroform-*d*) δ 169.0, 160.9, 158.8, 143.5, 136.6, 135.1, 131.0, 128.6, 128.5, 128.5, 128.0, 127.7, 127.4, 121.6, 114.7, 112.8, 107.0, 74.1, 70.0, 19.2, 13.5. **HRMS** (ESI-TOF) *m/z* calculated for $C_{27}H_{24}O_4$ (M + Na)$^+$: 435.1572, found 435.1571.

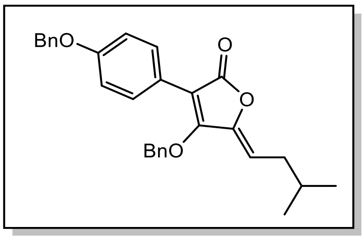

(*Z*)-4-(benzyloxy)-3-(4-(benzyloxy)phenyl)-5-(3-methylbutylidene)furan-2(5*H*)-one: Compound **41** was isolated as a yellow solid (20mg, 34% yield). **^{1}H NMR** (400 MHz, Chloroform-*d*) δ 7.48 – 7.30 (m, 10H), 7.20 – 7.13 (m, 2H), 7.06 – 6.98 (m, 2H), 5.52 (t, *J* = 8.2 Hz, 1H), 5.10 (s, 2H), 5.02 (s, 2H), 2.25 (dd, *J* = 8.1, 6.9 Hz, 2H), 1.76 (dt, *J* = 13.5, 6.7 Hz, 1H), 0.93 (d, *J* = 6.7 Hz, 6H). **^{13}C NMR** (101 MHz, Chloroform-*d*) δ 169.7, 161.3, 159.4, 145.1, 137.2, 135.7, 131.6, 129.3, 129.2, 129.1, 128.6, 128.3, 128.0, 122.2, 115.4, 110.9, 107.8, 74.7, 70.6, 35.2, 29.1, 22.9. **HRMS** (ESI-TOF) *m/z* calculated for $C_{29}H_{28}O_4$ $(M + Na)^+$: 463.1885, found 463.1876.

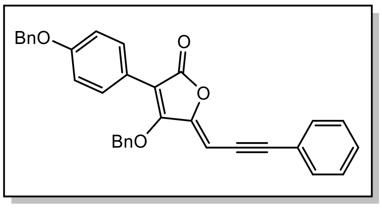

(*Z*)-4-(benzyloxy)-3-(4-(benzyloxy)phenyl)-5-(3-phenylprop-2-yn-1-ylidene)furan-2(5*H*)-one: Compound **42** was isolated as a yellow solid (49mg, 75% yield). **^{1}H NMR** (400 MHz, Chloroform-*d*) δ 7.48 – 7.13 (m, 15H), 7.06 (dd, *J* = 6.7, 2.9 Hz, 2H), 6.97 – 6.90 (m, 2H), 5.63 (s, 1H), 4.98 (d, *J* = 23.2 Hz, 4H); **^{13}C NMR** (101 MHz, Chloroform-*d*) δ 167.9, 160.4, 159.5, 151.5, 136.8, 135.0, 132.1, 131.8, 131.4, 131.4, 129.2, 129.1, 128.9, 128.9, 128.8, 128.6, 128.5, 128.4, 128.4, 128.1, 127.7, 122.9, 121.3, 115.2, 108.7, 101.1, 89.2, 83.3, 74.7, 70.3; **HRMS** (ESI-TOF) *m/z* calculated for $C_{33}H_{24}O_4$ $(M + Na)^+$: 507.1572, found 507.1564.

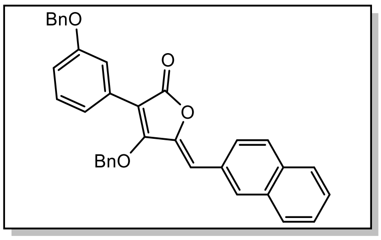

(*Z*)-4-(benzyloxy)-3-(3-(benzyloxy)phenyl)-5-(naphthalen-2-ylmethylene)furan-2(5*H*)-one: Compound **43** was isolated as a yellow solid (38mg, 55% yield). **^{1}H NMR** (600 MHz, Chloroform-*d*) δ 8.20 – 8.17 (m, 1H), 7.96 (dd, *J* = 8.6, 1.8 Hz, 1H), 7.90 – 7.85 (m, 1H), 7.87 – 7.76 (m, 2H), 7.53 – 7.46 (m, 2H), 7.49 – 7.43 (m, 2H), 7.45 – 7.37 (m, 2H), 7.40 – 7.33 (m, 3H), 7.36 – 7.30 (m, 2H), 7.24 – 7.18 (m, 2H), 7.16 (dd, *J* = 7.3, 1.4 Hz, 2H), 7.05 – 7.01 (m, 1H), 6.47 (s, 1H), 5.06 (d, *J* = 16.8 Hz, 4H); **^{13}C NMR** (101 MHz, Chloroform-*d*) δ 169.0, 163.0, 158.8, 143.2, 136.9, 135.2, 133.6, 133.5, 130.9, 130.7, 130.5, 129.8, 129.0, 128.9, 128.8, 128.8, 128.6, 128.3, 128.1, 127.9, 127.8, 127.7, 127.6, 127.2, 126.7, 123.0, 116.4, 115.9, 108.8, 106.6, 74.8, 70.2; **HRMS** (ESI-TOF) *m/z* calculated for $C_{35}H_{26}O_4$ $(M + H)^+$: 511.1909, found 511.1912.

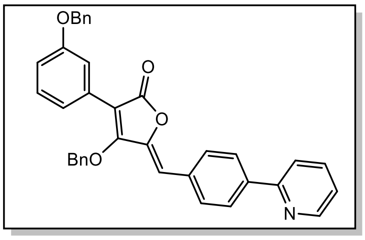

(*Z*)-4-(benzyloxy)-3-(3-(benzyloxy)phenyl)-5-(4-(pyridin-2-yl)benzylidene) furan-2(5*H*)-one: Compound **44** was isolated as a yellow solid (38mg, 74% yield). **^{1}H NMR** (600 MHz, Chloroform-*d*) δ 8.71 (dt, *J* = 4.8, 1.4 Hz, 1H), 8.06 – 7.98 (m, 2H), 7.92 – 7.87 (m, 2H), 7.81 – 7.74 (m, 2H), 7.47 – 7.43 (m, 2H), 7.43 – 7.33 (m, 4H), 7.36 – 7.29 (m, 3H), 7.28 – 7.22 (m, 1H), 7.22 – 7.16 (m, 2H), 7.18 – 7.06 (m, 2H), 7.03 (ddd, *J* = 8.3, 2.5, 1.1 Hz, 1H), 6.36 (s, 1H), 5.07 (s, 2H), 5.03 (s, 2H); ^{13}C **NMR** (101 MHz, Chloroform-*d*) δ 168.6, 162.6, 158.5, 156.4, 149.6, 143.1, 139.4, 136.8, 136.6, 134.9, 133.2, 130.9, 130.7, 130.3, 129.5, 128.7, 128.6, 128.5, 128.5, 128.2, 128.1, 128.0, 127.8, 127.4, 127.1, 126.4, 122.7, 122.3, 120.6, 116.1, 115.6, 107.8, 106.4, 74.5, 69.9; **HRMS** (ESI-TOF) *m/z* calculated for $C_{36}H_{27}NO_4$ (M + Na)$^+$: 560.1838, found 560.1837.

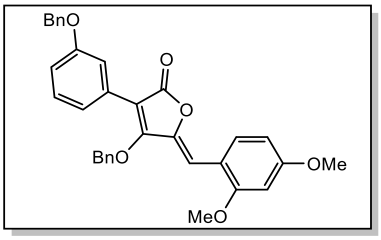

(*Z*)-4-(benzyloxy)-3-(3-(benzyloxy) phenyl)-5-(2,4-dimethoxybenzylidene)furan-2(5*H*)-one: Compound **45** was isolated as a yellow solid (15mg, 22% yield). **^{1}H NMR** (600 MHz, Chloroform-*d*) δ 8.16 (d, *J* = 8.8 Hz, 1H), 7.44 (d, *J* = 7.1 Hz, 2H), 7.39 (t, *J* = 7.6 Hz, 2H), 7.36 – 7.30 (m, 5H), 7.21 – 7.13 (m, 4H), 7.01 – 6.97 (m, 1H), 6.80 (s, 1H), 6.56 – 6.54 (m, 1H), 6.44 (dd, *J* = 5.7, 2.3 Hz, 1H), 5.04 (d, *J* = 4.7 Hz, 4H), 3.85 (s, 6H); **^{13}C NMR** (101 MHz, DMSO-*d$_6$*) δ 166.8, 163.7, 162.7, 159.2, 151.5, 151.4, 149.7, 139.2, 136.9, 136.1, 130.2, 128.8, 128.5, 128.1, 127.9, 127.7, 119.3, 118.8, 115.1, 112.6, 109.5, 108.0, 106.2, 99.2, 72.7, 69.9, 56.0, 55.6; **HRMS** (ESI-TOF) *m/z* calculated for $C_{33}H_{28}O_6$ (M + Na)$^+$: 543.1784, found 543.1773.

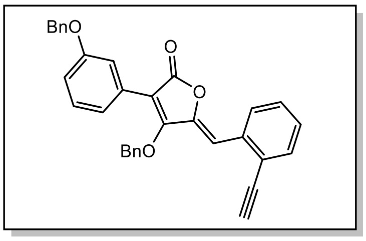

(*Z*)-4-(benzyloxy)-3-(3-(benzyloxy) phenyl)-5-(2-ethynylbenzylidene)furan-2(5*H*)-one: Compound **46** was isolated as a yellow solid (34mg, 53% yield). **^{1}H NMR** (600 MHz, Chloroform-*d*) δ 8.25 (dd, *J* = 8.1, 1.2 Hz, 1H), 7.54 (dd, *J* = 7.8, 1.4 Hz, 1H), 7.46 – 7.43 (m, 2H), 7.42 – 7.31 (m, 7H), 7.28 (dd, *J* = 7.6, 1.2 Hz, 1H), 7.21 – 7.16 (m, 4H), 7.05 – 7.02 (m, 1H), 7.00 (s, 1H), 5.07 (s, 4H), 3.36 (s, 1H); **^{13}C NMR** (101 MHz, Chloroform-*d*) δ 168.7, 163.2, 158.8, 144.0, 136.9, 135.3, 134.7, 133.2, 130.5,

130.4, 129.8, 129.5, 128.9, 128.9, 128.8, 128.7, 128.5, 128.3, 128.2, 127.8, 127.8, 127.7, 122.8, 116.1, 116.0, 107.5, 105.9, 82.8, 81.9, 74.8, 70.2; **HRMS** (ESI-TOF) *m/z* calculated for $C_{33}H_{28}O_6$ (M + Na)$^+$: 507.1572, found 507.1568.

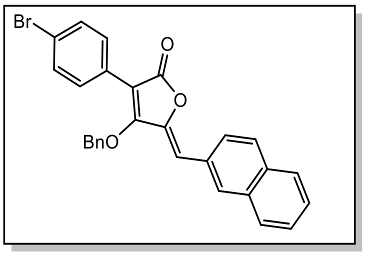

(*Z*)-4-(benzyloxy)-3-(4-bromophenyl)-5-(naphthalen-2-ylmethylene) furan-2(5*H*)-one: Compound **47** was isolated as a yellow solid (38.5mg, 55% yield). **^{1}H NMR** (600 MHz, Chloroform-*d*) *δ* 8.19 (s, 1H), 7.96 (dd, *J* = 8.7, 1.7 Hz, 1H), 7.90 – 7.86 (m, 1H), 7.83 (dd, *J* = 12.1, 7.4 Hz, 2H), 7.59 (d, *J* = 8.1 Hz, 2H), 7.51 (dq, *J* = 6.2, 3.3, 2.2 Hz, 2H), 7.46 (d, *J* = 8.1 Hz, 2H), 7.37 (p, *J* = 4.0, 3.5 Hz, 3H), 7.27 – 7.21 (m, 4H), 6.49 (s, 1H), 5.10 (s, 2H). **^{13}C NMR** (101 MHz, Chloroform-*d*) *δ* 168.7, 163.3, 143.1, 134.9, 133.6, 131.9, 131.7, 131.1, 130.4, 129.2, 129.0, 128.8, 128.7, 128.4, 128.1, 127.9, 127.5, 127.3, 126.8, 123.3, 109.3, 106.1, 75.2. **HRMS** (ESI-TOF) *m/z* calculated for $C_{28}H_{19}BrO_3$ (M + Na)$^+$: 505.0415, found 505.0419.

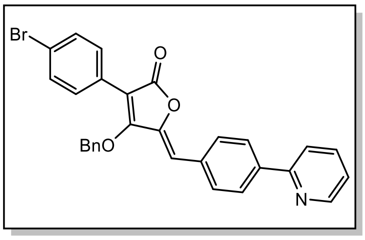

(*Z*)-4-(benzyloxy)-3-(4-bromophenyl)-5-(4-(pyridin-2-yl)benzylidene)furan-2(5*H*)-one: Compound **48** was isolated as a yellow solid (55mg, 74% yield). **^{1}H NMR** (600 MHz, Chloroform-*d*) *δ* 8.73 – 8.69 (m, 1H), 8.06 – 8.02 (m, 2H), 7.89 (d, *J* = 8.2 Hz, 2H), 7.81 – 7.74 (m, 2H), 7.60 – 7.55 (m, 2H), 7.48 – 7.43 (m, 2H), 7.37 (p, *J* = 3.5 Hz, 3H), 7.29 – 7.19 (m, 3H), 6.38 (s, 1H), 5.08 (s, 2H). **^{13}C NMR** (101 MHz, Chloroform-*d*) *δ* 168.6, 163.2, 156.7, 150.0, 143.2, 137.1, 134.9, 133.4, 131.9, 131.9, 131.7, 131.3, 131.3, 130.9, 129.2, 129.0, 129.0, 128.6, 128.5, 128.3, 128.1, 127.4, 126.8, 123.3, 122.7, 120.9, 120.7, 108.7, 106.2,75.2. **HRMS** (ESI-TOF) *m/z* calculated for $C_{28}H_{19}BrO_3$ (M + H)$^+$: 510.0705, found 510.0702.

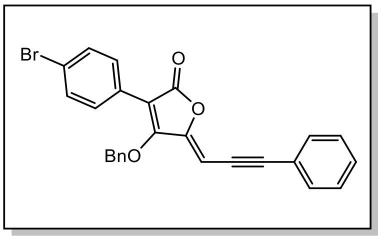

(*Z*)-4-(benzyloxy)-3-(4-bromophenyl)-5-(3-phenylprop-2-yn-1-ylidene)furan-2(5*H*)-one: Compound **49** was isolated as a yellow solid (18mg, 27% yield). **^{1}H NMR** (600 MHz, Chloroform-*d*) *δ* 7.57 (dd, *J* = 8.4, 2.3 Hz, 2H), 7.52 (d, *J* = 6.6 Hz, 2H), 7.48 – 7.42 (m, 2H), 7.35 (d, *J* = 5.4 Hz, 6H), 7.19 – 7.13 (m, 2H), 5.80 (d, *J* = 2.3 Hz,

1H), 5.06 (s, 2H). **^{13}C NMR** (101 MHz, Chloroform-*d*) δ 167.3, 161.4, 151.1, 134.6, 132.1, 132.0, 131.5, 129.4, 129.3, 129.0, 128.6, 128.0, 127.8, 123.6, 122.7, 107.6, 101.8, 90.3, 83.2, 75.2. **HRMS** (ESI-TOF) *m/z* calculated for $C_{26}H_{17}BrO_3$ $(M + H)^+$: 457.0439, found 457.0431.

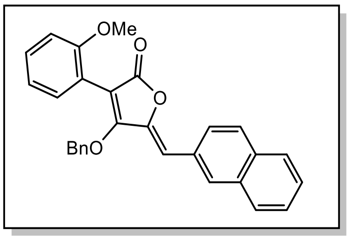

(*Z*)-4-(benzyloxy)-3-(2-methoxyphenyl)-5-(naphthalen-2-ylmethylene) furan-2(5*H*)-one: Compound **50** was isolated as a yellow solid (48mg, 65% yield). **^{1}H NMR** (600 MHz, Chloroform-*d*) δ 8.22 – 8.19 (m, 1H), 7.98 (dd, *J* = 8.6, 1.7 Hz, 1H), 7.87 (dt, *J* = 7.1, 3.0 Hz, 1H), 7.85 – 7.79 (m, 2H), 7.52 – 7.46 (m, 2H), 7.40 (ddd, *J* = 8.4, 7.5, 1.7 Hz, 1H), 7.37 – 7.33 (m, 1H), 7.35 – 7.29 (m, 3H), 7.20 – 7.15 (m, 2H), 7.04 (td, *J* = 7.5, 1.0 Hz, 1H), 6.96 (dd, *J* = 8.4, 1.0 Hz, 1H), 6.49 (s, 1H), 5.04 (s, 2H), 3.83 (s, 3H). **^{13}C NMR** (101 MHz, Chloroform-*d*) δ 169.3, 163.4, 158.0, 143.4, 135.5, 133.6, 133.4, 132.5, 130.7, 130.6, 128.8, 128.8, 128.7, 128.5, 127.9, 127.9, 127.6, 127.0, 126.6, 120.9, 118.7, 111.2, 107.9, 101.4, 73.3, 55.8. **HRMS** (ESI-TOF) *m/z* calculated for $C_{29}H_{22}O_4$ $(M + Na)^+$: 457.1416, found 457.1413.

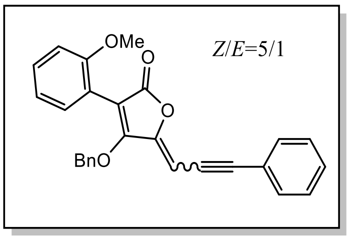

4-(benzyloxy)-3-(2-methoxyphenyl)-5-(3-phenylprop-2-yn-1-ylidene) furan-2(5*H*)-one: Compound **51** was isolated as a yellow solid (22mg, 32% yield). **^{1}H NMR** (600 MHz, Chloroform-*d*) δ 7.51 (dd, *J* = 6.7, 3.0 Hz, 2.4H, *Z*/*E*), 7.41 – 7.38 (m, 1.2H, *Z*/*E*), 7.32 (ddd, *J* = 18.3, 4.8, 1.8 Hz, 6H, *Z*), 7.27 (s, 2.4H, *Z*/*E*), 7.18 – 6.91 (m, 6H, *Z*/*E*), 6.00 (s, 0.2H, *E*), 5.78 (s, 1H, *Z*), 5.02 (s, 0.4H, *E*), 4.98 (s, 2H, *Z*), 3.84 (s, 0.6H, *E*), 3.81 (s, 3H, *Z*). **HRMS** (ESI-TOF) *m/z* calculated for $C_{27}H_{20}O_4$ $(M + Na)^+$: 431.1259, found 431.1259.

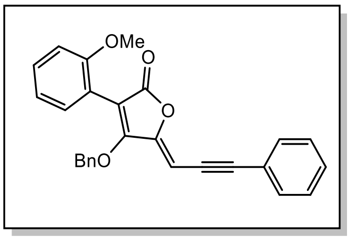

(*Z*)-4-(benzyloxy)-3-(2-methoxyphenyl)-5-(3-phenylprop-2-yn-1-ylidene)furan-2(5*H*)-one: Compound **51** was isolated as a yellow solid (18mg, 27% yield). **^{1}H NMR** (600 MHz, Chloroform-*d*) δ 7.53 – 7.49 (m, 2H), 7.42 – 7.37 (m, 1H), 7.36 – 7.30 (m, 6H), 7.28 (t, *J* = 1.3 Hz, 1H), 7.10 (dd, *J* = 6.4, 2.9 Hz, 2H), 7.02 (t, *J* = 7.5 Hz, 1H), 6.94 (d, *J* = 8.4 Hz, 1H), 5.78 (d, *J* = 1.0 Hz, 1H), 4.98 (s, 2H), 3.81 (d, *J* = 1.0 Hz, 3H).

^{13}C NMR (151 MHz, Chloroform-*d*) *δ* 167.8, 161.4, 157.7, 151.3, 135.1, 132.1, 131.9, 130.7, 128.9, 128.7, 128.6, 128.4, 127.6, 122.8, 120.6, 117.9, 110.9, 102.7, 100.2, 88.5, 83.0, 73.1, 55.5. **HRMS** (ESI-TOF) *m/z* calculated for $C_{27}H_{20}O_4$ (M + Na)$^+$: 431.1259, found 431.1259.

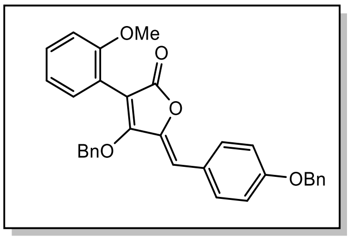

(*Z*)-4-(benzyloxy)-5-(4-(benzyloxy)benzylidene)-3-(2-methoxyphenyl) furan-2(5*H*)-one: Compound **52** was isolated as a yellow solid (50mg, 61% yield). **^{1}H NMR** (400 MHz, Chloroform-*d*) *δ* 7.75 (d, *J* = 8.9 Hz, 2H), 7.46 – 7.34 (m, 6H), 7.33 – 7.28 (m, 4H), 7.15 (dd, *J* = 6.6, 3.0 Hz, 2H), 7.04 – 6.92 (m, 4H), 6.29 (s, 1H), 5.09 (s, 2H), 5.00 (s, 2H), 3.81 (s, 3H). **^{13}C NMR** (101 MHz, Chloroform-*d*) *δ* 169.4, 163.5, 159.4, 158.0, 141.7, 136.8, 135.6, 132.5, 132.3, 130.6, 128.8, 128.8, 128.3, 127.9, 127.7, 126.1, 120.8, 118.9, 115.3, 111.1, 107.6, 100.9, 73.2, 70.2, 55.7. **HRMS** (ESI-TOF) *m/z* calculated for $C_{32}H_{26}O_5$ (M + Na)$^+$: 513.1678, found 513.1674.

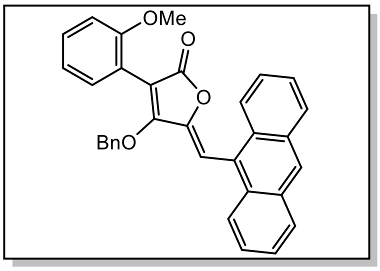

(*Z*)-5-(anthracen-9-ylmethylene)-4-(benzyloxy)-3-(2-methoxyphenyl) furan-2(5*H*)-one: Compound **53** was isolated as a yellow solid (35mg, 43% yield). **^{1}H NMR** (600 MHz, Chloroform-*d*) *δ* 8.46 (s, 1H), 8.32 (dt, *J* = 5.7, 3.6 Hz, 2H), 8.08 (d, *J* = 8.6 Hz, 2H), 8.01 (d, *J* = 8.2 Hz, 2H), 7.80 (dt, *J* = 5.8, 3.5 Hz, 2H), 7.49 (dt, *J* = 21.0, 6.9 Hz, 4H), 7.42 (d, *J* = 8.5 Hz, 1H), 7.30 – 7.22 (m, 3H), 7.05 (t, *J* = 7.4 Hz, 1H), 6.99 (d, *J* = 8.3 Hz, 1H), 5.15 (s, 2H), 3.89 (s, 3H). **^{13}C NMR** (101 MHz, Chloroform-*d*) *δ* 183.4, 168.8, 162.1, 158.0, 145.6, 135.6, 134.3, 133.7, 132.4, 131.5, 130.8, 130.1, 129.0, 128.9, 128.9, 128.3, 128.0, 127.4, 126.2, 126.2, 126.1, 125.5, 120.9, 118.5, 111.2, 104.5, 102.7, 73.4, 55.8. **HRMS** (ESI-TOF) *m/z* calculated for $C_{33}H_{24}O_4$ (M + Na)$^+$: 507.1572, found 507.1570.

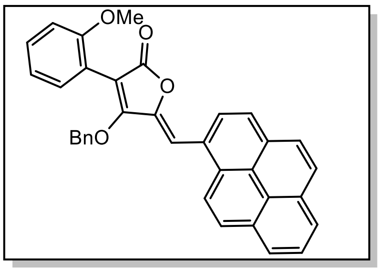

(*Z*)-4-(benzyloxy)-3-(2-methoxyphenyl)-5-(pyren-1-ylmethylene) furan-2(5*H*)-one: Compound **54** was isolated as a yellow solid (58mg, 68% yield). **^{1}H NMR** (600 MHz,

Pyridine-d_5) δ 8.92 (d, J = 8.1 Hz, 1H), 8.51 (d, J = 9.2 Hz, 1H), 8.28 – 8.24 (m, 3H), 8.19 – 8.14 (m, 2H), 8.11 (d, J = 8.9 Hz, 1H), 8.04 (t, J = 7.6 Hz, 1H), 7.66 (dd, J = 7.4, 1.7 Hz, 1H), 7.63 (s, 1H), 7.50 – 7.46 (m, 1H), 7.42 – 7.32 (m, 5H), 7.16 (td, J = 7.4, 1.0 Hz, 1H), 7.06 (dd, J = 8.5, 1.0 Hz, 1H), 5.36 (s, 2H), 3.79 (s, 3H); **^{13}C NMR** (151 MHz, Pyridine-d_5) δ 168.7, 163.2, 158.1, 144.5, 132.6, 131.7, 131.6, 130.9, 130.8, 129.7, 128.8, 128.7, 128.6, 128.5, 128.5, 128.0, 127.7, 126.9, 126.5, 126.1, 125.8, 125.4, 125.0, 124.8, 120.6, 118.9, 111.3, 103.5, 101.8, 73.4, 55.4; **HRMS** (ESI-TOF) *m/z* calculated for $C_{35}H_{24}O_4$ $(M + Na)^+$: 531.1572, found 531.1576.

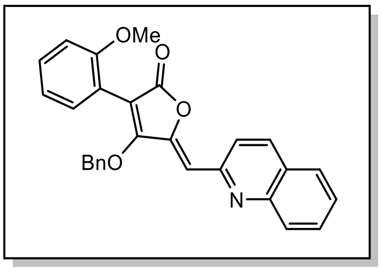

(*Z*)-4-(benzyloxy)-3-(2-methoxyphenyl)-5-(quinolin-2-ylmethylene) furan-2(5*H*)-one: Compound **55** was isolated as a yellow solid (32mg, 43% yield). **^{1}H NMR** (600 MHz, Chloroform-*d*) δ 8.38 (d, J = 8.7 Hz, 1H), 8.18 (d, J = 8.7 Hz, 1H), 8.05 (d, J = 8.6 Hz, 1H), 7.82 (d, J = 8.1 Hz, 1H), 7.72 (t, J = 7.7 Hz, 1H), 7.55 (t, J = 7.5 Hz, 1H), 7.42 (td, J = 7.9, 1.7 Hz, 1H), 7.32 (dt, J = 6.0, 2.4 Hz, 4H), 7.19 – 7.13 (m, 2H), 7.05 (t, J = 7.5 Hz, 1H), 6.97 (d, J = 8.4 Hz, 1H), 6.79 (s, 1H), 5.04 (s, 2H), 3.84 (s, 3H). **^{13}C NMR** (151 MHz, Chloroform-*d*) δ 168.6, 163.2, 157.8, 152.9, 148.2, 146.3, 136.4, 134.9, 132.2, 130.8, 129.8, 129.3, 128.7, 128.6, 127.8, 127.6, 127.2, 127.0, 122.8, 120.7, 118.0, 110.9, 108.4, 101.9, 73.3, 55.6. **HRMS** (ESI-TOF) *m/z* calculated for $C_{28}H_{21}NO_4$ $(M + Na)^+$: 458.1368, found 458.1375.

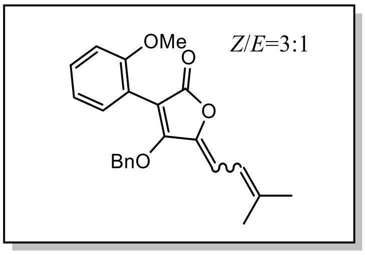

4-(benzyloxy)-3-(2-methoxyphenyl)-5-(3-methylbut-2-en-1-ylidene) furan-2(5*H*)-one: Compound **56** was isolated as a yellow solid (13mg, 21% yield). **^{1}H NMR** (600 MHz, Chloroform-*d*) δ 7.39 – 7.34 (m, 4H, *E*), 7.33 – 7.26 (m, 12H, *Z*), 7.26 – 7.25 (m, 4H, *Z*/*E*), 7.19 – 7.06 (m, 8H, *Z*/*E*), 7.02 – 6.97 (m, 4H, *Z*/*E*), 6.95 – 6.90 (m, 4H, *Z*/*E*), 6.69 (d, J = 12.4, 1.4 Hz, 1H, *E*), 6.51 (d, J = 12.4 Hz, 1H, *E*), 6.36 (d, J = 11.9, 1.4 Hz, 3H, *Z*), 6.25 (d, J = 11.9 Hz, 3H, *Z*), 4.97 (s, 8H, *Z*/*E*), 3.80 (s, 12H, *Z*/*E*), 1.91 (s, 9H, *Z*), 1.86 (s, 9H, *Z*), 1.83 (s, 3H, *E*), 1.81 (s, 3H, *E*). **HRMS** (ESI-TOF) *m/z* calculated for $C_{23}H_{22}O_4$ $(M + Na)^+$: 385.1416, found 385.1409.

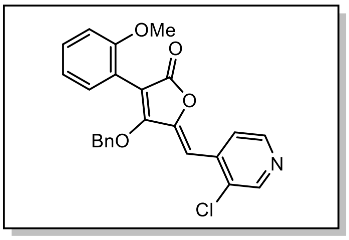

(*Z*)-4-(benzyloxy)-5-((3-chloropyridin-4-yl)methylene)-3-(2-methoxyphenyl) furan-2(5*H*)-one: Compound **57** was isolated as a yellow solid (32mg, 18% yield). **^{1}H NMR** (600 MHz, Chloroform-*d*)*δ* 8.62 (d, J = 2.9 Hz, 1H), 8.49 (t, J = 4.1 Hz, 1H), 8.08 (t, J = 4.3 Hz, 1H), 7.43 (dt, J = 9.5, 4.7 Hz, 1H), 7.32 – 7.31 (m, 3H), 7.11 (d, J = 4.7 Hz, 2H), 7.04 (td, J = 7.7, 2.6 Hz, 1H), 6.96 (dd, J = 8.5, 2.8 Hz, 1H), 6.70 (d, J = 3.0 Hz, 1H), 5.06 (d, J = 2.8 Hz, 2H), 3.82 (d, J = 3.0 Hz, 3H); **^{13}C NMR** (101 MHz, Chloroform-*d*) *δ* 168.2, 162.6, 157.9, 150.0, 148.2, 147.7, 138.2, 135.1, 132.2, 131.2, 128.9, 128.8, 127.7, 124.8, 120.9, 117.8, 111.1, 103.0, 99.8, 73.4, 55.7; **HRMS** (ESI-TOF) *m/z* calculated for $C_{24}H_{18}ClNO_4$ (M + H)$^+$: 420.1003, found 420.1001.

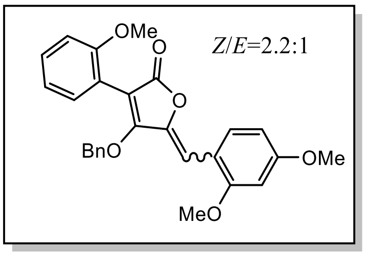

4-(benzyloxy)-5-(2,4-dimethoxybenzylidene)-3-(2-methoxyphenyl)furan-2(5*H*)-one: Compound **58** was isolated as a yellow solid (48mg, 64% yield). **^{1}H NMR** (600 MHz, Chloroform-*d*) *δ* 8.17 (d, *J* = 8.7 Hz, 2.2H, *Z*), 7.43 (dd, *J* = 8.5, 0.8 Hz, 1H, *E*), 7.39 – 7.34 (m, 3.2H, *Z*/*E*), 7.32 – 7.26 (m, 8.8H, *Z*), 7.26 – 7.21 (m, 2.2H, *Z*), 7.21 – 7.16 (m, 2.2H, *Z*), 7.13 (dd, *J* = 6.7, 2.8 Hz, 4.2H, *Z*/*E*), 7.03 – 6.98 (m, 3.2H, *Z*/*E*), 6.95 – 6.91 (m, 3.2H, *Z*/*E*), 6.90 (s, 1H, *E*), 6.87 – 6.84 (m, 2H, *E*), 6.80 (s, 2H, *E*), 6.55 (dd, *J* = 8.8, 2.4 Hz, 2.2H, *Z*), 6.44 (d, *J* = 2.4 Hz, 2.2H, *Z*), 6.40 (d, *J* = 2.4 Hz, 1H, *E*), 6.12 (dd, *J* = 8.6, 2.4 Hz, 1H, *E*), 5.02 (s, 4.4H, *Z*), 4.83 (s, 2H, *E*), 3.85 (s, 13.2H, *Z*), 3.84 – 3.80 (m, 6H, *E*), 3.79 (s, 6.6H, *Z*), 3.75 (s, 3H, *E*); **^{13}C NMR** (101 MHz, Chloroform-*d*) *δ* 169.7, 163.7, 161.8, 159.0, 158.0, 141.5, 135.8, 132.9, 132.5, 131.0, 130.5, 128.7, 128.6, 128.4, 127.9, 127.7, 120.8, 119.1, 115.2, 111.0, 105.9, 105.4, 101.8, 100.6, 98.4, 98.2, 72.9, 55.8, 55.8, 55.7, 55.7; **HRMS** (ESI-TOF) *m/z* calculated for $C_{27}H_{24}O_6$ (M + Na)$^+$: 467.1471, found 467.1475.

5 Mechanism study

5.1 The preparation of compound 59

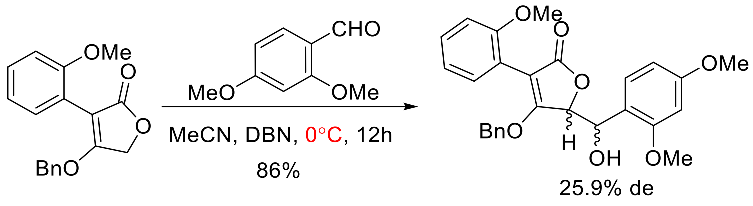

59

The procedures are same as **4.1**.

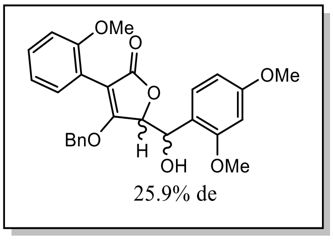

4-(benzyloxy)-5-((2,4-dimethoxyphenyl)(hydroxy) methyl)-3-(2-methoxyphenyl) furan-2(5*H*)-one: Compound **59** was isolated as a white crystal. **^{1}H NMR** (600 MHz, Chloroform-*d*) δ 7.41 (dd, J = 8.4, 0.7 Hz, 1.7H), 7.39 – 7.26 (m, 12.5H), 7.24 (dd, J = 8.4, 0.6 Hz, 1H), 7.10 – 7.05 (m, 3.4H), 7.04 – 6.95 (m, 4.7H), 6.94 – 6.89 (m, 2.7H), 6.86 (dd, J = 8.4, 1.0 Hz, 1H), 6.51 (dd, J = 8.5, 2.4 Hz, 1.7H), 6.45 (d, J = 2.4 Hz, 1H), 6.44 (d, J = 2.4 Hz, 1.7H), 6.40 (dd, J = 8.5, 2.4 Hz, 1H), 5.38 – 5.33 (m, 2.7H), 5.27 (d, J = 3.4 Hz, 1H), 5.10 (d, J = 2.6 Hz, 1.7H), 4.96 (s, 3.4H), 4.78 (q, J = 11.8 Hz, 2H), 3.80 (t, J = 5.6 Hz, 18.3H), 3.78 (s, 3H), 3.72 (s, 3H); **^{13}C NMR** (101 MHz, Chloroform-*d*) δ 173.31, 171.88, 171.82, 160.88, 160.84, 158.10, 157.83, 157.58, 135.49, 135.33, 132.53, 132.46, 130.37, 130.30, 129.25, 128.68, 128.65, 128.56, 128.52, 127.62, 127.49, 120.84, 120.66, 120.03, 119.20, 118.07, 111.12, 111.10, 104.62, 104.56, 101.85, 101.71, 98.77, 80.95, 80.68, 73.26, 73.05, 70.89, 68.12, 55.84, 55.71, 55.61, 55.55; **HRMS** (ESI-TOF) *m/z* calculated for $C_{27}H_{26}O_7$ $(M + Na)^+$: 485.1576, found 485.1579.

5.2 The exchange of methyl and benzyl groups

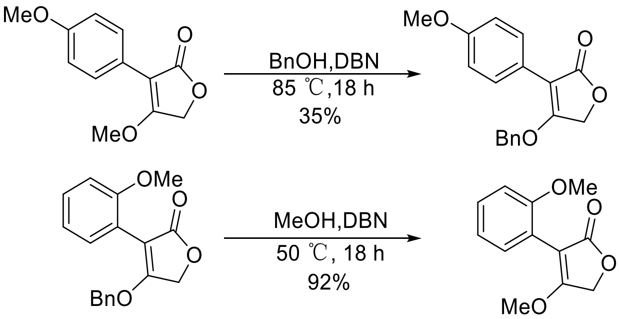

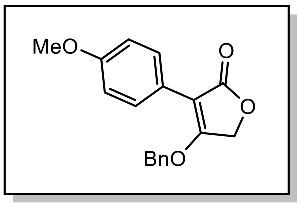

4-(benzyloxy)-3-(4-methoxyphenyl) furan-2(5H)-one: Compound **60** was isolated as a white solid (22 mg, 35% yield). **^{1}H NMR** (600 MHz, Chloroform-*d*) δ 7.88 – 7.82 (m, 2H), 7.44 – 7.32 (m, 5H), 6.96 – 6.90 (m, 2H), 5.18 (s, 2H), 4.81 (s, 2H), 3.82 (s, 3H); **^{13}C NMR** (151 MHz, Chloroform-*d*) δ 172.74, 170.63, 158.97, 134.68, 129.14, 129.09, 129.06, 127.26, 121.75, 113.74, 103.30, 72.58, 64.99, 55.26; **HRMS** (ESI-TOF) *m/z* calculated for $C_{18}H_{16}O_4$ $(M + Na)^+$: 319.0946, found 319.0941.

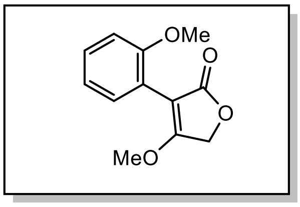

4-methoxy-3-(2-methoxyphenyl) furan-2(5H)-one: Compound **61** was isolated as a white solid (34 mg, 92% yield). **^{1}H NMR** (600 MHz, Chloroform-*d*) δ 7.34 (ddd, J = 8.3, 7.4, 1.8 Hz, 1H), 7.25 (dd, J = 7.5, 1.8 Hz, 1H), 6.99 (td, J = 7.5, 1.1 Hz, 1H), 6.93 (dd, J = 8.3, 1.1 Hz, 1H), 4.72 (s, 2H), 3.83 (s, 3H), 3.75 (s, 3H); **^{13}C NMR** (151 MHz, Chloroform-*d*) δ 173.60, 172.99, 157.62, 132.07, 130.00, 120.52, 118.62, 110.99, 100.14, 66.67, 58.65, 55.58; **HRMS** (ESI-TOF) *m/z* calculated for $C_{12}H_{12}O_4$ $(M + Na)^+$: 243.0633, found 243.0625.

6 Deprotection of natural aspulvinone precursors

6.1 general procedure for aspulvinone E, P, Q, R, O and unnamed aspulvinone 62, 63.

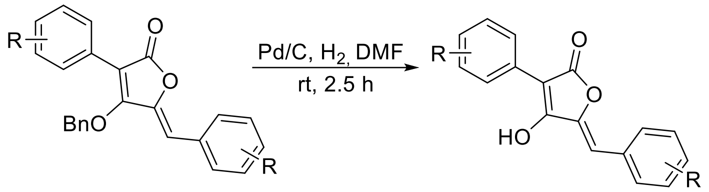

To a suspension of 10% Pd/C (50mg) and compound (50mg) in DMF (1mL) was added two drops of concentrated HCl. The reaction mixture was placed under a hydrogen atmosphere at 1 atm, under vigorous stirring for 2.5 h. Hydrogen was replaced by argon, then the suspension was filtered over a short pad of Celite, which was washed with MeOH. After concentration, DCM (12ml) was added dropwise to the solution that containing DMF and product. The mixture solution of DCM and DMF was left for a day at 0°C and the precipitated product (yellow solid) was isolated by filtration. This product was washed by DCM for several times. The product was finally pure by ^{1}H NMR.

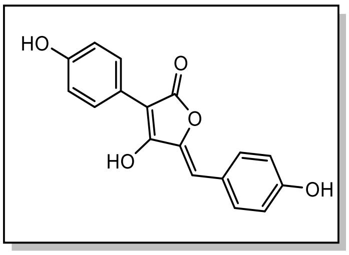

Aspulvinone E: Aspulvinone E was isolated as a yellow solid (21mg, 82% yield). **^{1}H NMR** (400 MHz, DMSO-*d*$_6$) δ 9.90 (s, 1H), 9.53 (s, 1H), 7.80 (d, J = 7.7 Hz, 2H), 7.58 (d, J = 7.7 Hz, 2H), 6.86 (d, J = 7.7 Hz, 2H), 6.82 (d, J = 7.7 Hz, 2H), 6.58 (s, 1H); **^{13}C NMR** (101 MHz, DMSO-*d*$_6$) δ 168.4, 162.7, 158.2, 156.3, 140.5, 131.8, 128.3, 124.0, 121.1, 115.9, 115.1, 107.0, 99.2; **HRMS** (ESI-TOF) *m/z* calculated for $C_{17}H_{12}O_5$ (M + Na)$^{+}$: 319.0577, found 319.0577. NMR data are consistent with the literature.[12]

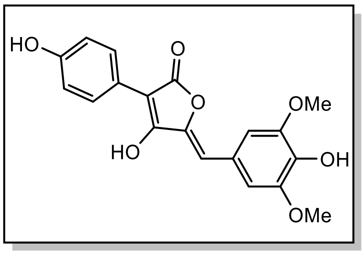

Aspulvinone P: Aspulvinone P was isolated as a yellow solid (22mg, 76% yield). **^{1}H NMR** (400 MHz, DMSO-*d*$_6$) δ 9.56 (s, 1H), 8.95 (s, 1H), δ 7.75 (d, J = 8.4 Hz, 2H),

7.02 (s, 2H), 6.82 (d, J = 8.4 Hz, 2H), 6.54 (s, 1H), 3.81 (s, 6H); **^{13}C NMR** (101 MHz, DMSO-d_6) δ 168.2, 161.9, 156.6, 148.1, 140.6, 137.3, 128.7, 123.2, 120.7, 115.2, 108.1, 107.9, 100.1, 56.1; **HRMS** (ESI-TOF) *m/z* calculated for $C_{19}H_{16}O_7$ (M + Na)$^+$: 379.0794, found 379.0798. NMR data are consistent with the literature.[13]

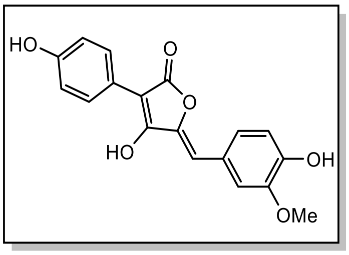

Aspulvinone Q: Aspulvinone Q was isolated as a yellow solid (22mg, 80% yield). **^{1}H NMR** (600 MHz, DMSO-d_6) δ 9.56 (s, 2H), 7.80 – 7.75 (m, 2H), 7.31 (d, J = 2.0 Hz, 1H), 7.19 (dd, J = 8.3, 2.0 Hz, 1H), 6.86 (d, J = 8.2 Hz, 1H), 6.84 – 6.78 (m, 2H), 6.59 (s, 1H), 3.82 (s, 3H); **^{13}C NMR** (101 MHz, DMSO-d_6) δ 168.7, 162.6, 156.9, 148.3, 148.2, 140.8, 129.0, 124.8, 124.7, 121.3, 116.5, 115.6, 114.2, 108.1, 100.2, 56.1; **HRMS** (ESI-TOF) *m/z* calculated for $C_{18}H_{14}O_6$ (M + Na)$^+$: 349.0682, found 349.0682. NMR data are consistent with the literature.[14]

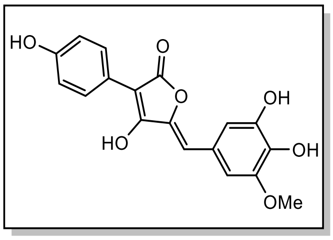

Aspulvinone R: Aspulvinone R was isolated as a yellow solid (18mg, 73% yield). **^{1}H NMR** (400 MHz, DMSO-d_6) δ 9.55 (s, 1H), 9.26 (s, 1H), 8.80 (s, 1H), 7.80 – 7.72 (m, 2H), 6.98 (d, J = 1.9 Hz, 1H), 6.84 – 6.77 (m, 2H), 6.77 (d, J = 2.0 Hz, 1H), 6.49 (s, 1H), 3.78 (s, 3H); **^{13}C NMR** (151 MHz, DMSO-d_6) δ 168.8, 162.5, 156.9, 148.7, 146.4, 140.8, 136.4, 128.9, 123.6, 121.3, 115.6, 111.6, 108.5, 106.8, 100.1, 56.4; **HRMS** (ESI-TOF) *m/z* calculated for $C_{18}H_{14}O_7$ (M + Na)$^+$: 365.0631, found 365.0635. NMR data are consistent with the literature.[14]

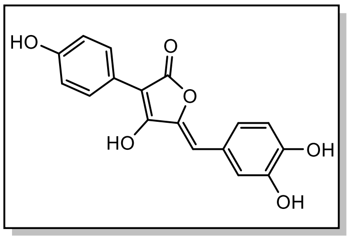

(*Z*)-5-(3,4-dihydroxybenzylidene)-4-hydroxy-3-(4-hydroxyphenyl) furan-2(5*H*)-one: Compound **62** was isolated as a yellow solid (19mg, 84% yield). **^{1}H NMR** (600 MHz, DMSO-d_6) δ 12.00 (s, 1H), 10.05 (s, 1H), 9.79 (s, 1H), 9.53 (s, 1H), 7.78 (dd, J = 8.6, 5.5 Hz, 3H), 6.97 (s, 1H), 6.84 – 6.78 (m, 2H), 6.39 (d, J = 2.4 Hz, 1H), 6.35 (dd, J = 8.7, 2.4 Hz, 1H); **^{13}C NMR** (101 MHz, DMSO-d_6) δ 168.8, 162.4, 160.1, 158.1, 156.8, 139.6, 132.1, 128.9, 121.4, 115.6, 115.5, 112.0, 108.3, 103.0, 102.7, 100.0; **HRMS** (ESI-TOF) *m/z* calculated for $C_{17}H_{12}O_6$ (M + Na)$^+$: 335.0526, found 335.0525.

NMR data was lack in the original literature (1975).[15]

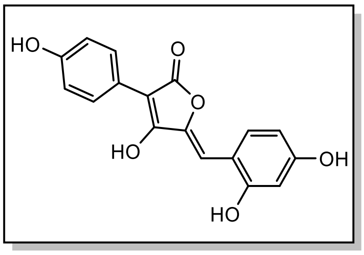

(*Z*)-5-(2,4-dihydroxybenzylidene)-4-hydroxy-3-(4-hydroxyphenyl) furan-2(5*H*)-one: Compound **63** was isolated as a yellow solid (20mg, 88% yield). **^{1}H NMR** (600 MHz, Methanol-*d*$_4$) δ 7.94 (d, J = 8.6 Hz, 1H), 7.77 (d, J = 8.2 Hz, 2H), 6.98 (s, 1H), 6.87 – 6.82 (m, 2H), 6.40 – 6.34 (m, 2H), 3.36 (s, 1H), 2.73 (s, 2H); **^{13}C NMR** (101 MHz, Methanol-*d*$_4$) δ 171.8, 164.0, 161.3, 159.3, 157.9, 141.0, 133.4, 130.3, 122.7, 116.2, 113.7, 109.0, 104.2, 103.2, 102.0; **HRMS** (ESI-TOF) *m/z* calculated for $C_{17}H_{12}O_6$ (M + Na)$^+$: 335.0526, found 335.0528. NMR data was lack in the original literature (1975).[15]

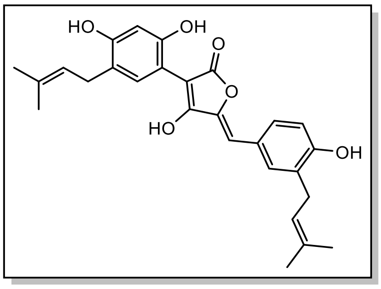

Aspulvinone O: Aspulvinone O was isolated as a yellow solid (15mg, 54% yield). **^{1}H NMR** (600 MHz, Acetone-*d*$_6$) δ 7.70 (s, 1H), 7.59 (d, J = 2.2 Hz, 1H), 7.55 (dd, J = 8.4, 2.3 Hz, 1H), 6.91 (d, J = 8.4 Hz, 1H), 6.59 (s, 1H), 6.27 (s, 1H), 5.35 (ddddd, J = 14.2, 7.3, 5.8, 2.9, 1.5 Hz, 2H), 3.35 (d, J = 7.4 Hz, 2H), 3.27 (d, J = 7.3 Hz, 2H), 1.80 – 1.65 (m, 12H); **^{13}C NMR** (151 MHz, Acetone-*d*$_6$) δ 169.9, 163.3, 157.5, 157.4, 153.4, 142.4, 133.9, 133.1, 132.1, 131.1, 130.2, 126.9, 125.0, 124.3, 122.5, 117.1, 110.4, 108.1, 104.6, 100.6, 29.9, 29.5, 26.8, 18.8, 18.8; **HRMS** (ESI-TOF) *m/z* calculated for $C_{27}H_{28}O_6$ (M + Na)$^+$: 471.1784, found 471.1780. X-ray structure of Aspulvinone O was listed in 10.4.

6.2 Procedures for aspulvinone B.

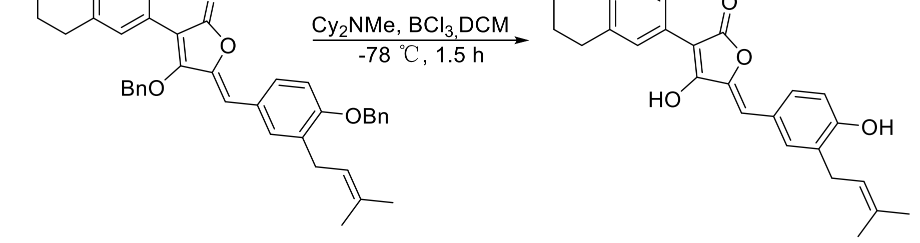

To a solution of **29** (52.3 mg, 0.10mmol) in CH_2Cl_2 (5 mL) was added 1.0 M Cy_2NMe at 0° C in toluene (0.40 mL, 0.40 mmol). The mixture was cooled to –78 °C and 1 M

BCl_3 in hexanes (1.00 mL, 1.0 mmol,) was added dropwise. After 90 min the mixture was transferred into a suspension of CaO (280 mg, 5.0 mmol) in MeOH (5 mL) at – 78 °C, and warmed to r.t. within 45 min. CH_2Cl_2 (20 mL) was added. The mixture was washed with aq 0.05 M HCl (3 × 5 mL). The organic layers were washed with aq phosphate buffer (2 × 10 mL). The combined organic layers were dried (Na_2SO_4) and evaporated in vacuo. The residue was purified by silica gel chromatography (PE–EA, 3:1 with 1% AcOH) furnished the crude title compound. By dropwise addition of a solution of this material in CH_2Cl_2 (2 ml) to vigorously stirred pentane (40 mL), aspulvinone B precipitated. [9]

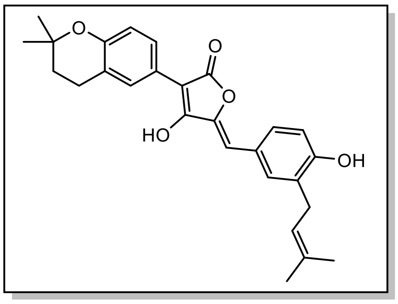

Aspulvinone B: Aspulvinone B was isolated as a yellow solid (8mg, 23% yield). **^{1}H NMR** (400 MHz, DMSO-d_6) δ 7.70 (s, 1H), 7.59 (d, J = 2.2 Hz, 1H), 7.55 (dd, J = 8.4, 2.3 Hz, 1H), 6.91 (d, J = 8.4 Hz, 1H), 6.59 (s, 1H), 6.27 (s, 1H), 5.35 (ddddd, J = 14.2, 7.3, 5.8, 2.9, 1.5 Hz, 2H), 3.35 (d, J = 7.4 Hz, 2H), 3.27 (d, J = 7.3 Hz, 2H), 1.80 – 1.65 (m, 12H); **^{13}C NMR** (101 MHz, DMSO-d_6) δ 168.1, 156.1, 152.7, 139.9, 131.9, 131.7, 129.3, 128.3, 128.1, 126.4, 123.8, 122.2, 121.4, 120.5, 116.6, 115.4, 107.9, 99.6, 74.3, 32.1, 27.8, 26.6, 25.5, 21.9, 17.7; **HRMS** (ESI-TOF) *m/z* calculated for $C_{27}H_{28}O_5$ (M + Na)$^+$: 455.1834, found 455.1830. NMR data are consistent with the literature.[16]

7 Synthesis of the isoaspulvinones

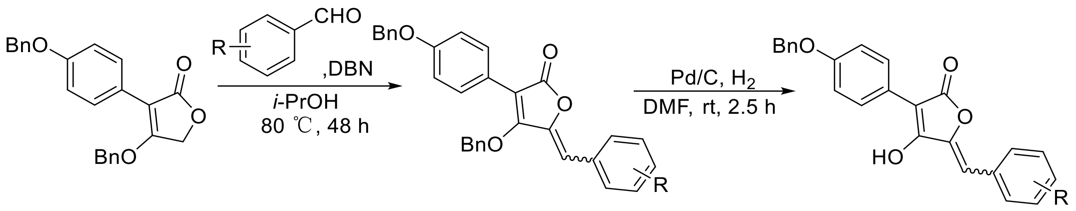

An oven dried glass bottle, which contained a stirring bar, was charged with the tetronic acid (50 mg), the aldehyde (3 eq) and *i*-PrOH (1 ml). Then, the DBN (1 eq) was added dropwise via syringe into the bottle, and the reaction was run on the 80 °C in oil bath for 48 h and was monitored by TLC (PE/EA= 2:1). At 365 nm on TLC plate, the product was appeared as a yellow-green fluorescent dot typically. After the reaction, the silica gel (100-200 mesh) and reaction solution were mixed, and the solvent was evaporated by the rotary evaporator. The crude product was purified by column chromatography with petroleum ether and ethyl acetate (20:1) to gain the target products in moderate yields. The deprotection of products are same as section 6.

The mixture of *Z*/*E* aspulvinones were purified using semi-preparative HPLC eluted with MeCN-H_2O-HCOOH (60:40:0.1, v/v/v,2.0 mL/min).

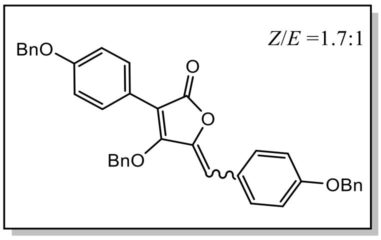

4-(benzyloxy)-5-(4-(benzyloxy) benzylidene)-3-(4-(benzyloxy) phenyl) furan-2(5H)-one: The compound **64** was isolated as a yellow solid (32mg, 42% yield). **^{1}H NMR** (400 MHz, Chloroform-*d*) δ 7.72 (d, *J* = 5.8 Hz, 3.4H, *Z*), 7.67 (d, *J* = 8.8 Hz, 2H, *E*), 7.54 – 7.49 (m, 5.4H, *Z*/*E*), 7.46 – 7.31 (m, 37.1H, *Z*/*E*), 7.21 (dd, *J* = 4.6, 2.0 Hz, 3.4H, *Z*), 7.10 (d, *J* = 8.8 Hz, 2H, *E*), 7.03 (d, *J* = 5.8 Hz, 3.4H, *Z*), 7.00 – 6.95 (m, 5.4H, *Z*/*E*), 6.22 (d, *J* = 3.2 Hz, 2.7H, *Z*/*E*), 5.12 – 5.05 (m, 16.2H, *Z*/*E*); **HRMS** (ESI-TOF) *m/z* calculated for $C_{38}H_{30}O_5$ $(M + H)^+$: 567.2166, found 567.2164.

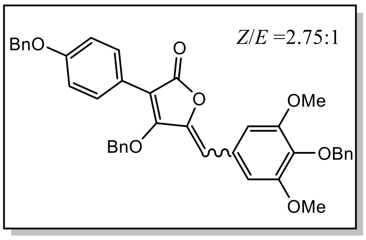

4-(benzyloxy)-5-(4-(benzyloxy)-3,5-dimethoxybenzylidene)-3-(4-(benzyloxy) phenyl) furan-2(5*H*)-one: Compound **65** was isolated as a yellow solid (32mg, 34% yield). **^{1}H NMR** (400 MHz, Chloroform-*d*) δ 7.51 – 7.29 (m, 58.25H, *Z*/*E*), 7.21 (dd, *J* = 4.5, 2.0 Hz, 5.5H, *Z*), 7.08 (d, *J* = 4.0 Hz, 2H, *E*), 7.04 (d, *J* = 5.7 Hz, 5.5H, *Z*), 7.00 (s, 5.5H, *Z*), 6.96 (s, 2H, *E*), 6.18 (d, *J* = 2.3 Hz, 3.75H, *Z*/*E*), 5.11 (s, 5.5H, *Z*), 5.09 (s,

4H, *E*), 5.06 (s, 11H, *Z*), 4.97 (s, 2H, *E*), 3.87 (s, 16.5H, *Z*), 3.84 (s, 6H, *E*); **HRMS** (ESI-TOF) *m/z* calculated for $C_{46}H_{38}O_7$ (M + Na)$^+$: 725.2509, found 725.2514.

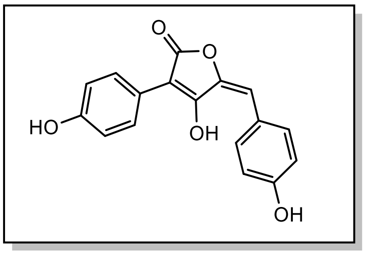

Isoaspulvinone E: Isoaspulvinone E was isolated as a yellow solid (18mg, 71% yield). **^{1}H NMR** (600 MHz, DMSO-d_6) δ 9.84 (s, 1H), 9.65 (s, 1H), 7.63 (d, J = 7.7 Hz, 2H), 7.48 (d, J = 7.6 Hz, 2H), 6.80 (d, J = 7.7 Hz, 2H), 6.75 (d, J = 7.7 Hz, 2H), 6.64 (s, 1H); **^{13}C NMR** (101 MHz, DMSO-d_6) δ 168.7, 161.4, 158.5, 157.3, 142.7, 132.8, 130.2, 120.8, 115.7, 115.6, 115.1, 105.1; **HRMS** (ESI-TOF) *m/z* calculated for $C_{17}H_{12}O_5$ (M + Na)$^+$: 319.0577, found 319.0577. NMR data are consistent with the literature.[12]

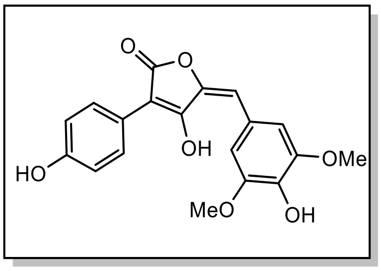

Isoaspulvinone P: Isoaspulvinone P was isolated as a yellow solid (19mg, 64% yield). **^{1}H NMR** (400 MHz, DMSO-d_6) δ 9.61 (s, 1H), 8.82 (s, 1H), 7.45 (d, J = 8.5 Hz, 2H), 7.41 (s, 2H), 6.80 (d, J = 8.5 Hz, 2H), 6.46 (s, 1H), 3.83 (s, 6H); **^{13}C NMR** (151 MHz, DMSO-d_6) δ 168.5, 160.8, 157.5, 147.6, 142.6, 137.5, 130.7, 120.4, 120.0, 115.8, 115.6, 109.3, 106.1, 55.4; **HRMS** (ESI-TOF) *m/z* calculated for $C_{19}H_{16}O_7$ (M + Na)$^+$: 379.0794, found 379.0798.

8 Pharmacological results and methods

8.1 Results

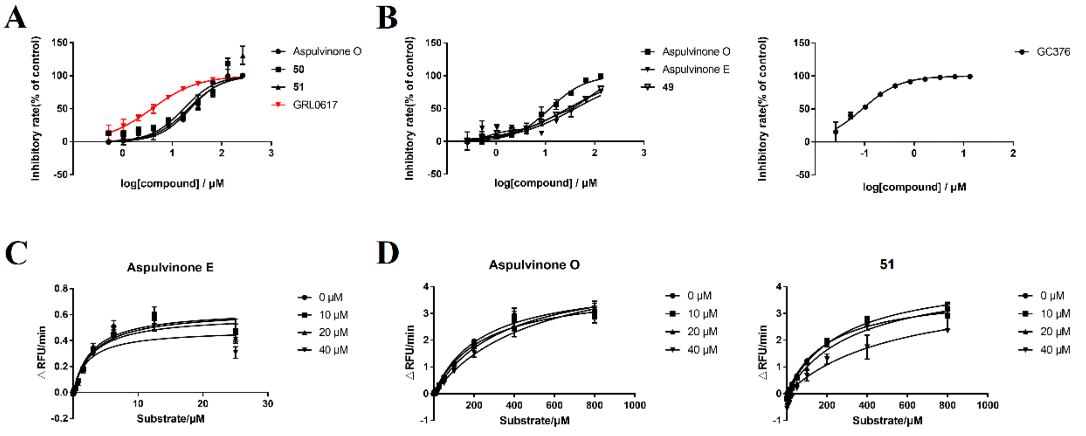

Aspulvinone E	0 μM	10 μM	20 μM	40 μM
V_{max} (RFU/min)	0.64 ± 0.05	0.63 ± 0.04	0.59 ± 0.05	0.48 ± 0.05
K_i' (μM)	2.93 ± 0.68	3.19 ± 0.63	2.92 ± 0.77	2.29 ± 0.74

Aspulvinone O	0 μM	10 μM	20 μM	40 μM
V_{max} (RFU/min)	4.20 ± 0.24	3.96 ± 0.20	4.63 ± 0.14	5.17 ± 0.25
K_m (μM)	237.60 ± 32.81	236.60 ± 29.41	342.70 ± 23.02	502.90 ± 47.00

51	0 μM	10 μM	20 μM	40 μM
V_{max} (RFU/min)	3.81 ± 0.18	4.48 ± 0.33	4.40 ± 0.61	4.07 ± 1.36
K_m (μM)	206.80 ± 25.42	279.40 ± 47.59	337.90 ± 10.18	550.40 ± 34.36

Figure S1. Antiviral activity of aspulvinone analogues. (A). Aspulvinone O, **50** and **51** inhibited SARS-CoV-2 PL^{pro} activity with a micromolar level, and GRL0617 acted as a positive control. (B). Aspulvinone O, aspulvinone E and **49** inhibited SARS-CoV-2 M^{pro} activity with a micromolar level, and GC376 acted as a positive control. (C). The mechanism of action of aspulvinone E against SARS-CoV-2 M^{pro}, which inhibited M^{pro} in an uncompetitive way. (D). The mechanism of action of aspulvinone O and **51** against SARS-CoV-2 PL^{pro}. The two aspulvinone analogues both inhibited PL^{pro} in a competitive way.

Table S1. Aspulvinone analogues inhibited SARS-CoV-2 M^{pro} activity with a micromolar level.

**Compound**	**IC_{50} (μM)**
Aspulvinone O	12.41 ± 2.40
Aspulvinone E	39.93 ± 2.42
**49**	28.25 ± 2.37
GC376	0.09 ± 0.01

Table S2. Aspulvinone analogues inhibited SARS-CoV-2 PL^{pro} activity with a micromolar level.

**Compound**	**IC_{50} (μM)**
Aspulvinone O	21.34 ± 0.94
**51**	23.05 ± 0.07
**50**	17.43 ± 2.60
GRL0617	3.92 ± 0.87

8.2 Method

8.2.1 Protein Expression and Purification

The genes that encode SARS-CoV-2 M^{pro} (GenBank: MN908947.3, residues 3258-3569) and PL^{pro} (GenBank: MN908947.3, residues 1564-1878) were optimized and synthesized for *E. coli* expression (Genewiz). To obtain the authentic SARS-CoV-2 M^{pro}, five residues SAVLQ were added to the N-terminus, and two residues GP plus a 6xHis tag to the C-terminus according to the published protocol.[17] The vector pGEX-6p-1 and pET 28a SUMO were used for expression of SARS-CoV-2 M^{pro} and PL^{pro}, respectively.

The *E. coli* strain BL21 (DE3) was used for recombinant protein production. When the OD_{600} reached 0.6-0.8, 0.4 mM IPTG was added to the cell culture to induce expression. After further cultured for 16 h at 18 °C, cells were harvested by centrifugation (3000 g, 10 min). The cell pellets were resuspended in pre-chilling lysis buffer (50 mM Tris-HCl pH 8.0, 300 mM NaCl, 10 mM imidazole, 1% v/v Triton X-100, 10% v/v glycerol, 5 mM β-Mercaptoethanol, 1mM PMSF), and then lysed by sonication. The insoluble debris was separated with recombinant protein by ultracentrifugation at 10000 g for 1 h. The supernatant containing recombinant protein was then applied to Ni-NTA affinity column (Qiagen), then washed by lysis buffer for 10 column volumes and eluted in lysis buffer except supplied with 300 mM imidazole. PL^{pro} was further cleaved by the sumo protease ULP1 to remove the sumo tag. The final protein exhibited high purity (>95%), which was verified by SDS-PAGE.

8.2.2 Enzymatic activity and inhibition assays

Two fluorescent substrates (MCA–AVLQSGFR-K(Dnp)K and Z-RLRGG-AMC) harbored the corresponding cleavage sites of SARS-CoV-2 M^{pro} and PL^{pro}, respectively, and were employed to monitor the enzymatic activity of M^{pro} and PL^{pro} as well as its inhibition by compounds. After released from its corresponding acceptors, donor fluorophores could be excited by a light source and measured quantitatively, and the fluorescence signal was proportional to the remaining enzymatic activity of M^{pro} and PL^{pro}. The excitation/emission wavelengths of MCA–AVLQSGFR-K(Dnp)K and Z-RLRGG-AMC were 320/405 nm and 360/460 nm, respectively.

For M^{pro}, 1 μl compound or DMSO was mixed with 48 μl M^{pro} in reaction buffer (50 mM Tris-HCl pH 8.0, 100 mM NaCl, 0.1 mg/ml BSA, 0.01% v/v Triton X-100, 5 mM β-Mercaptoethanol) for incubation of 30 min (room temperature). The reaction was triggered by adding 1 μl substrate and monitored for 30 mins at 37 °C. For preliminary high-throughput screening, the final mixture contained 40 μM compound, 150 nM M^{pro}, 30 μM substrate. For enzyme kinetics assay, the final concentration of substrate was ranged from 25 μM to 0.01 μM by serial dilution, and the final concentration of compounds was used as indicated.

Assays for PL^{pro} was almost set identical with M^{pro} except that the final concentration of PL^{pro} was 50 nM, and the final concentration of substrate was ranged from 800 μM to 0.39 μM in enzyme kinetics assay.

All experimental data was analysed using GraphPad Prism, and all experiments were performed in triplicate.

9 NMR spectra

^{1}H NMR (400 MHz, DMSO-d_6)

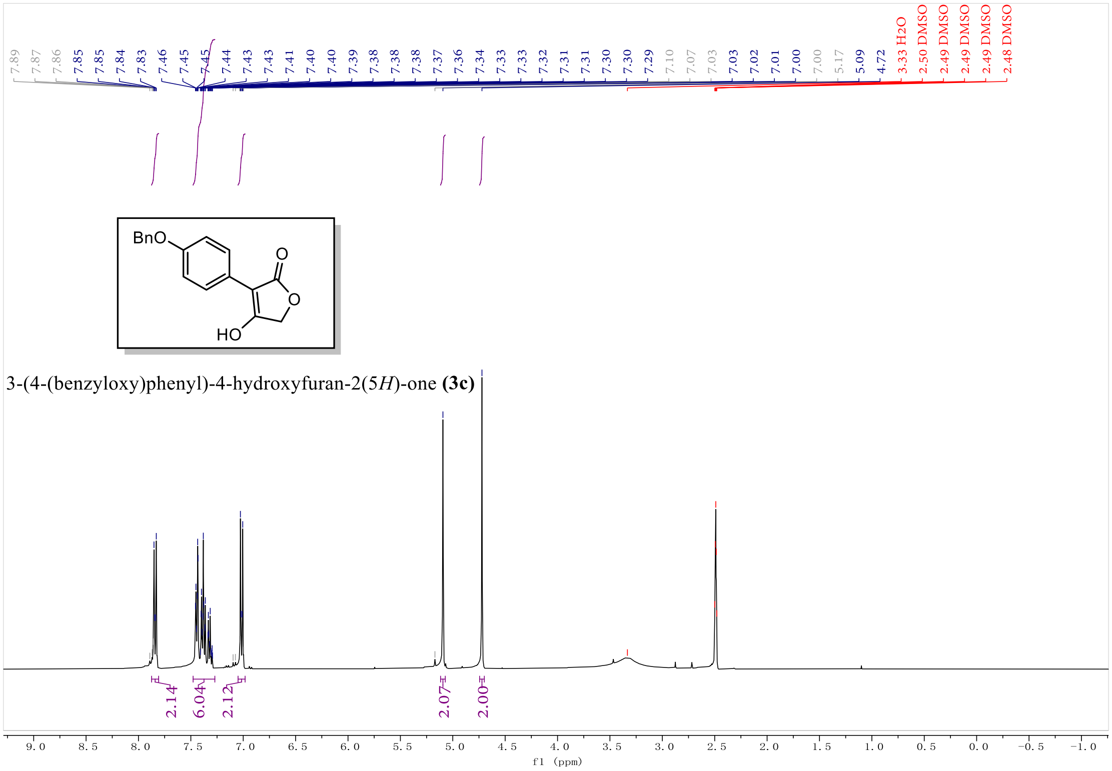

3-(4-(benzyloxy)phenyl)-4-hydroxyfuran-2(5*H*)-one **(3c)**

^{1}H NMR (400 MHz, DMSO-d_6)

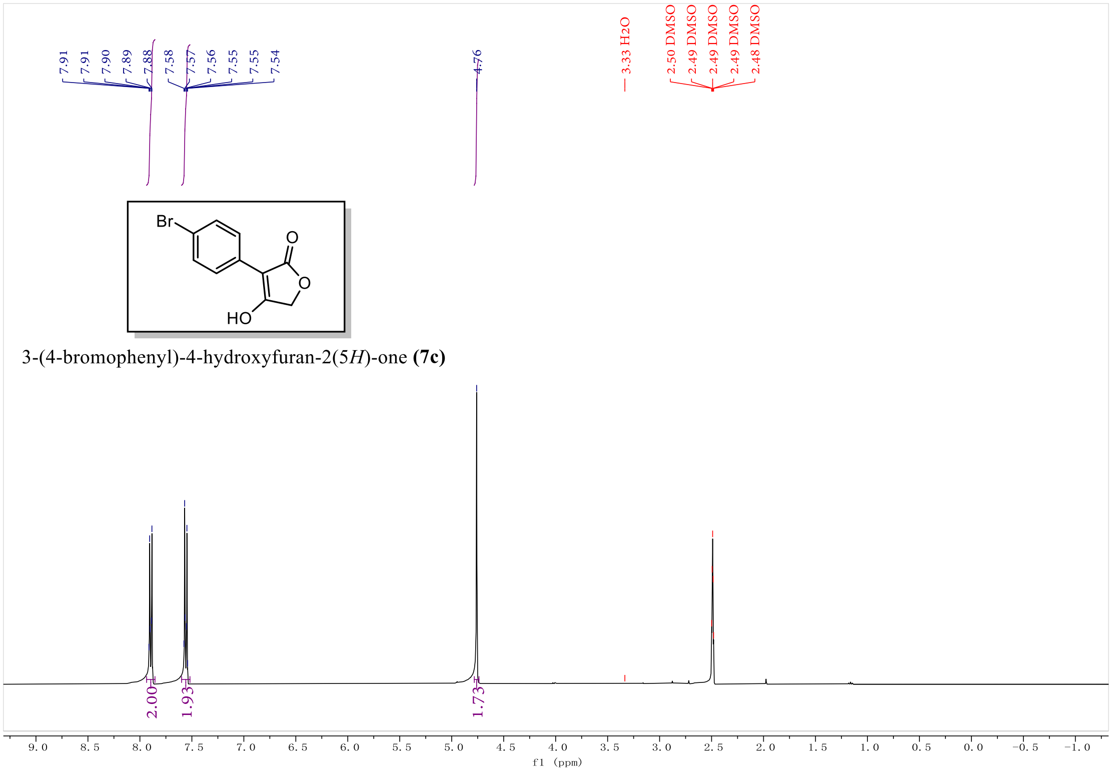

3-(4-bromophenyl)-4-hydroxyfuran-2(5*H*)-one **(7c)**

^{1}H NMR (400 MHz, DMSO-d_6)

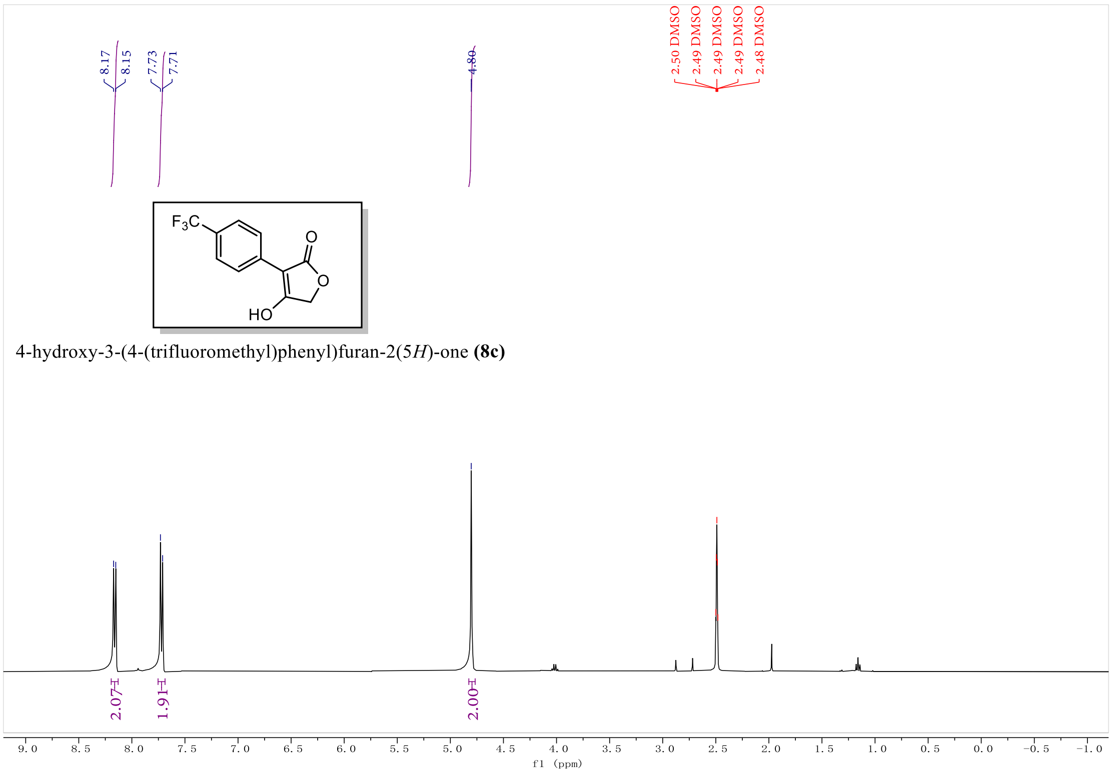

4-hydroxy-3-(4-(trifluoromethyl)phenyl)furan-2(5*H*)-one **(8c)**

^{19}F NMR (565 MHz, DMSO-d_6)

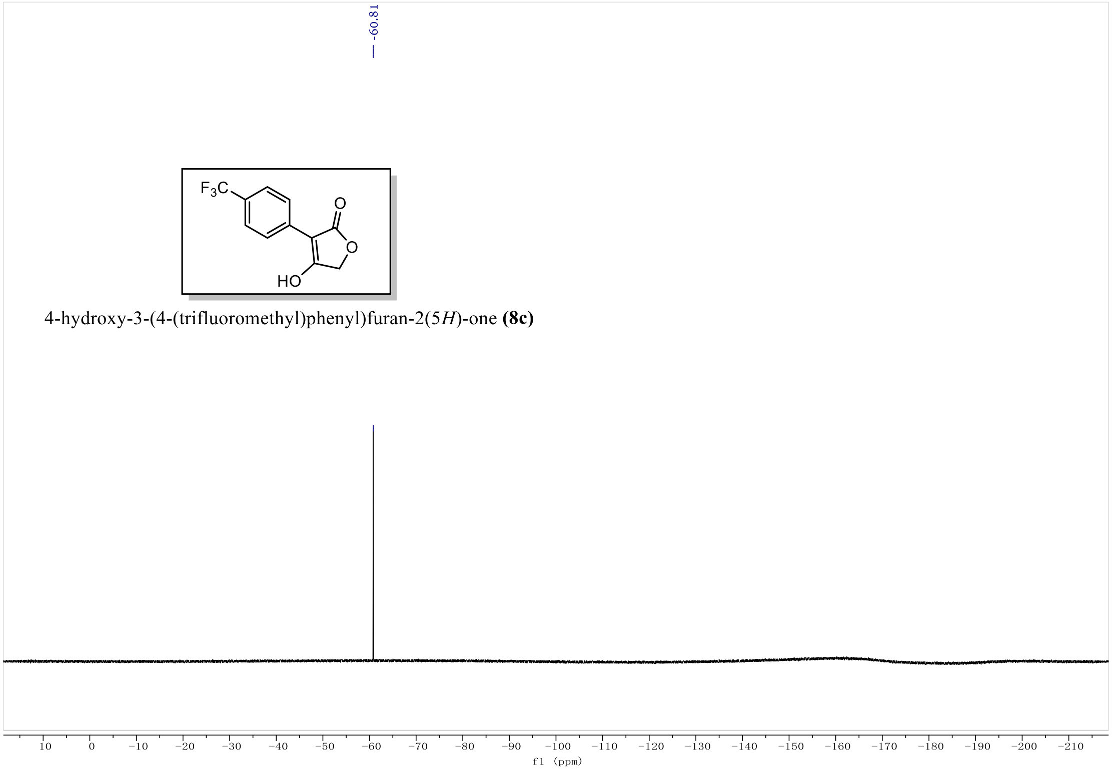

4-hydroxy-3-(4-(trifluoromethyl)phenyl)furan-2(5*H*)-one **(8c)**

^{1}H NMR (400 MHz, DMSO-d_6)

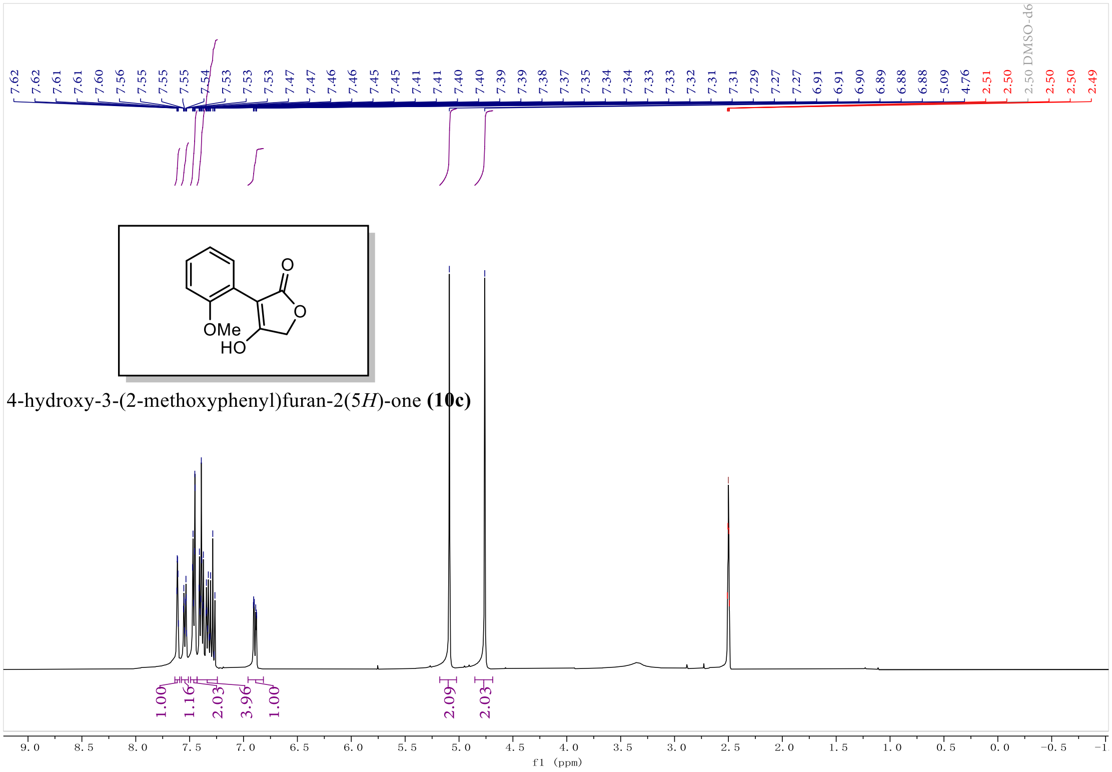

4-hydroxy-3-(2-methoxyphenyl)furan-2(5*H*)-one **(10c)**

^{1}H NMR (600 MHz, DMSO-d_6)

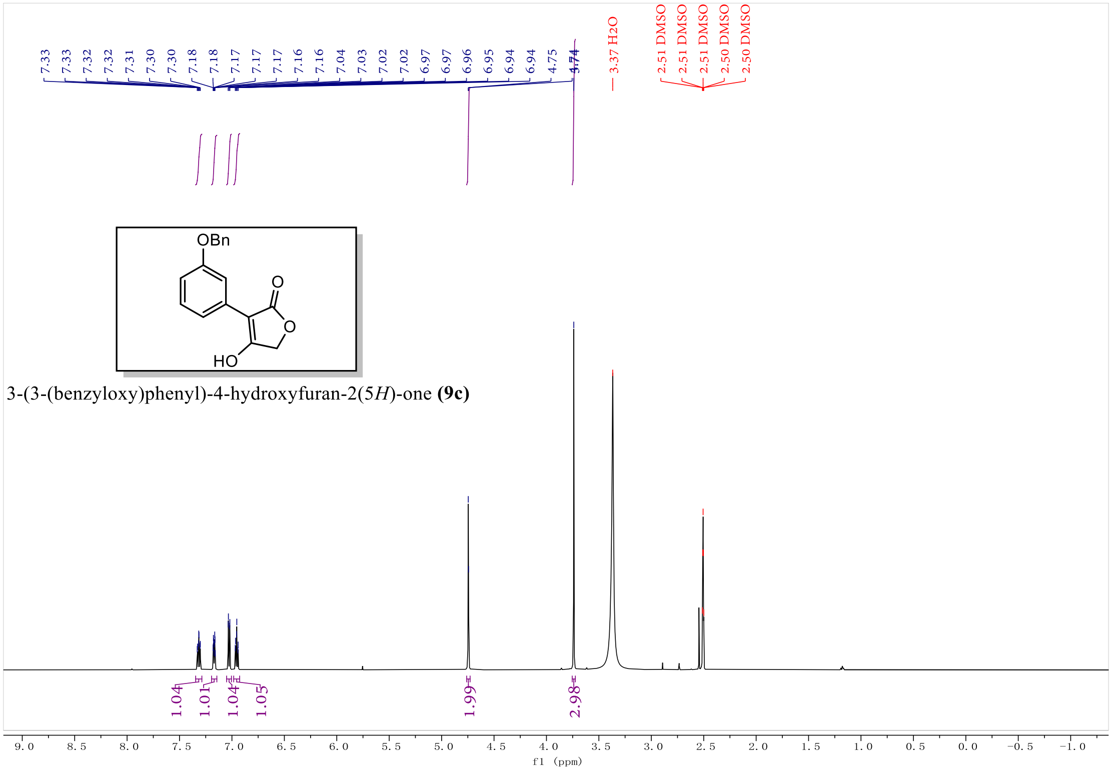

3-(3-(benzyloxy)phenyl)-4-hydroxyfuran-2(5*H*)-one **(9c)**

^{1}H NMR (600 MHz, Methanol-d_4)

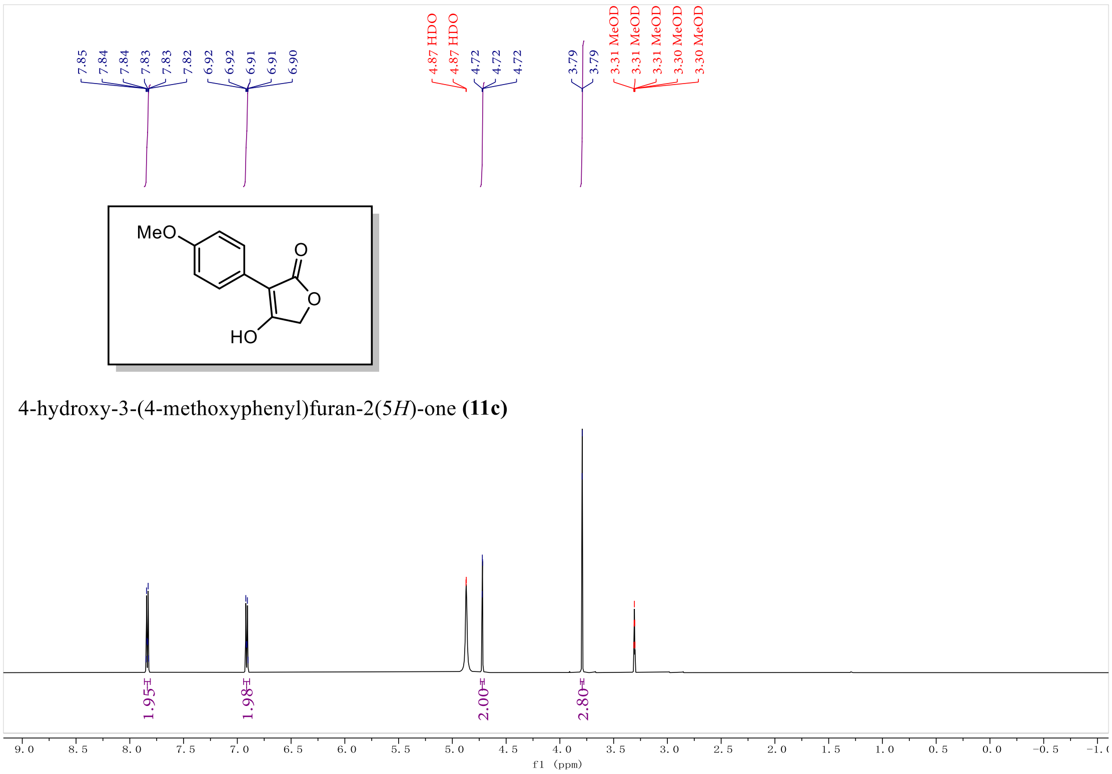

4-hydroxy-3-(4-methoxyphenyl)furan-2(5*H*)-one **(11c)**

^{1}H NMR (600 MHz, Chloroform-*d*)

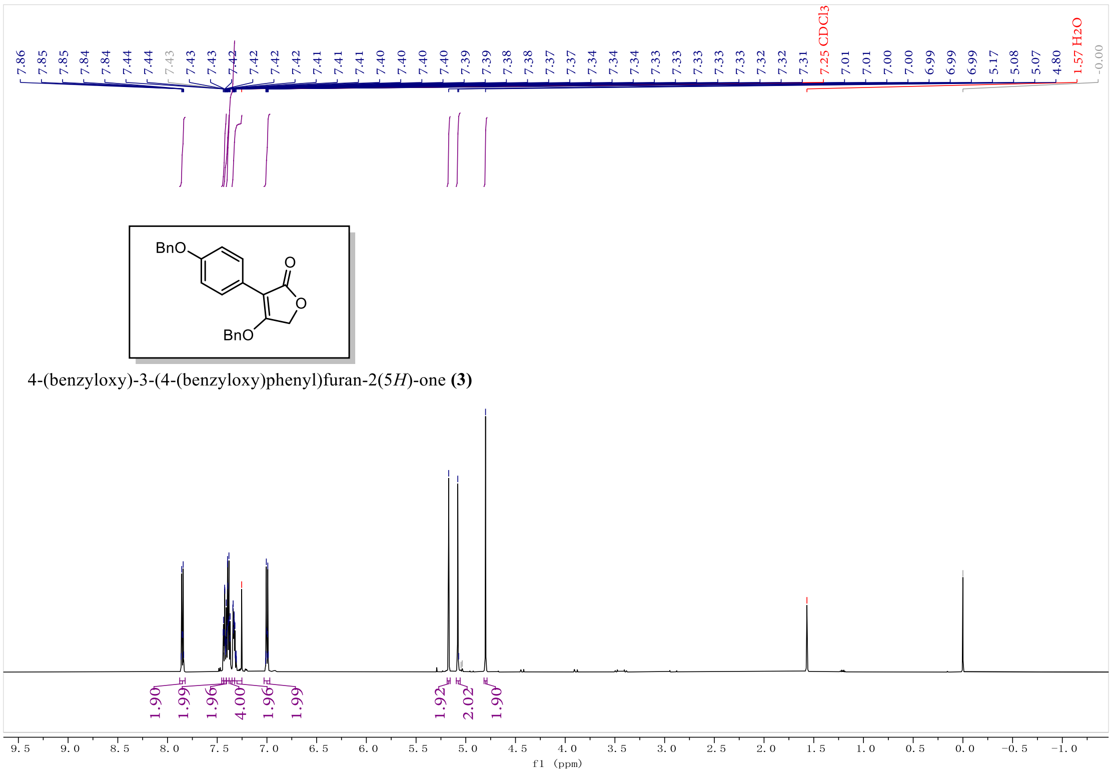

4-(benzyloxy)-3-(4-(benzyloxy)phenyl)furan-2(5*H*)-one **(3)**

^{13}C NMR (151 MHz, Chloroform-*d*)

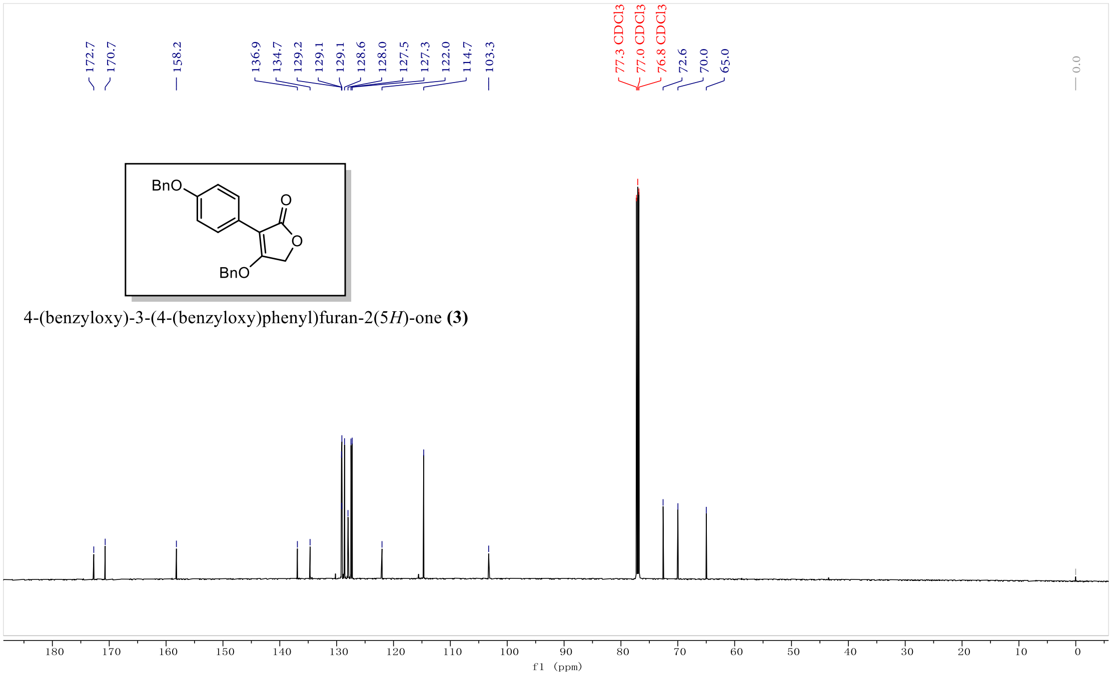

4-(benzyloxy)-3-(4-(benzyloxy)phenyl)furan-2(5*H*)-one **(3)**

^{1}H NMR (400 MHz, Chloroform-*d*)

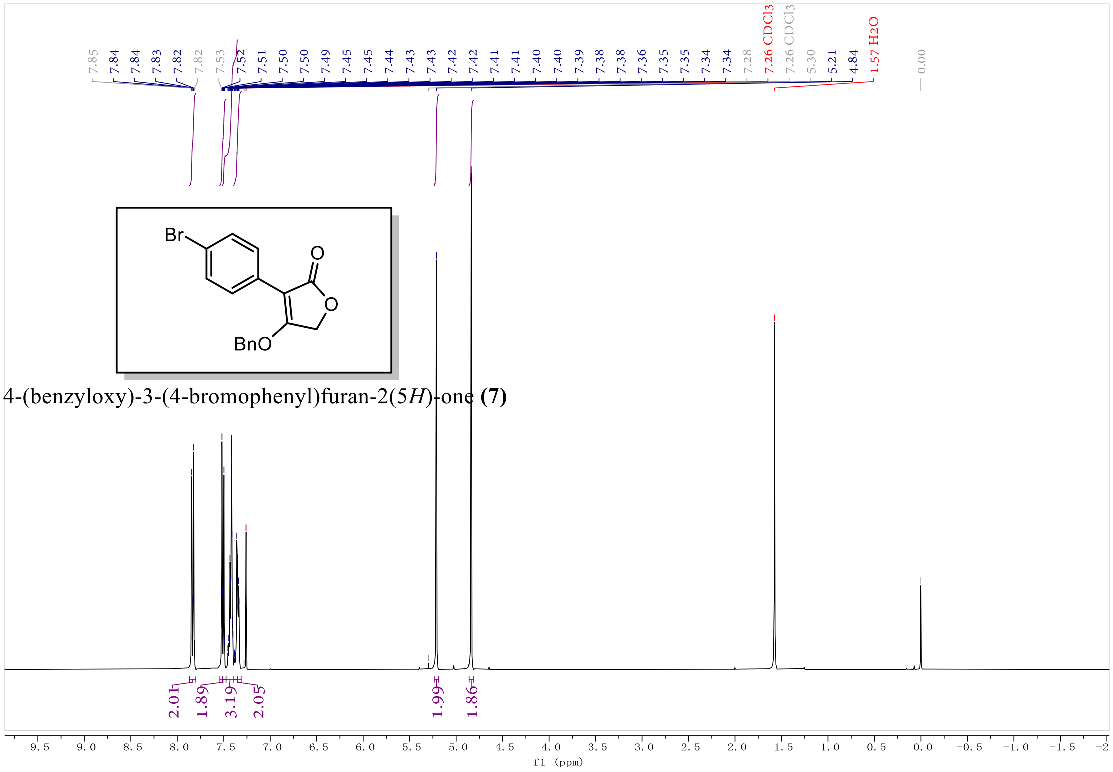

4-(benzyloxy)-3-(4-bromophenyl)furan-2(5*H*)-one **(7)**

^{13}C NMR (101 MHz, Chloroform-*d*)

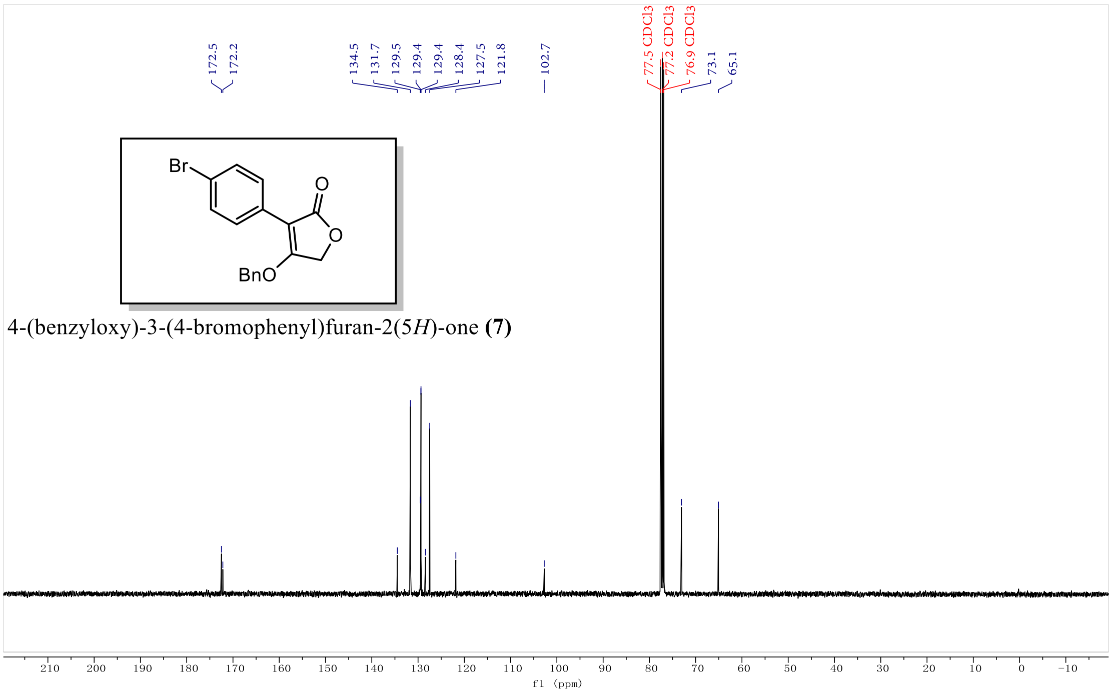

4-(benzyloxy)-3-(4-bromophenyl)furan-2(5*H*)-one **(7)**

^{1}H NMR (400 MHz, Chloroform-*d*)

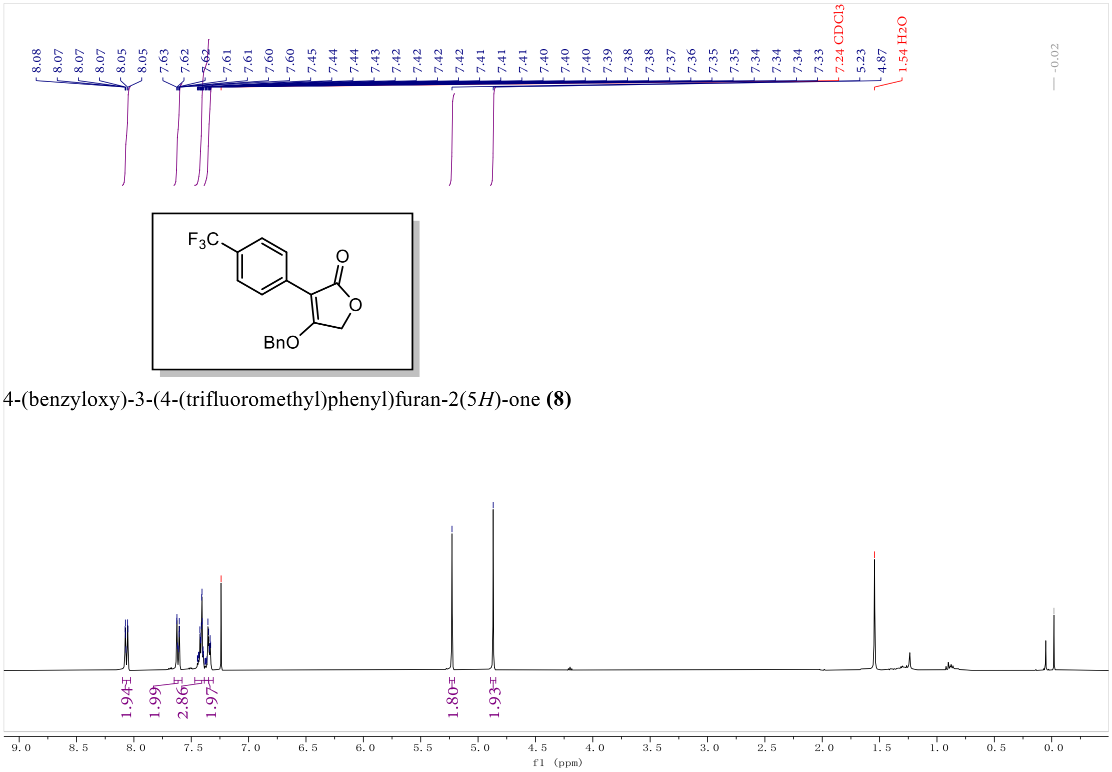

4-(benzyloxy)-3-(4-(trifluoromethyl)phenyl)furan-2(5*H*)-one **(8)**

^{13}C NMR (101 MHz, Chloroform-*d*)

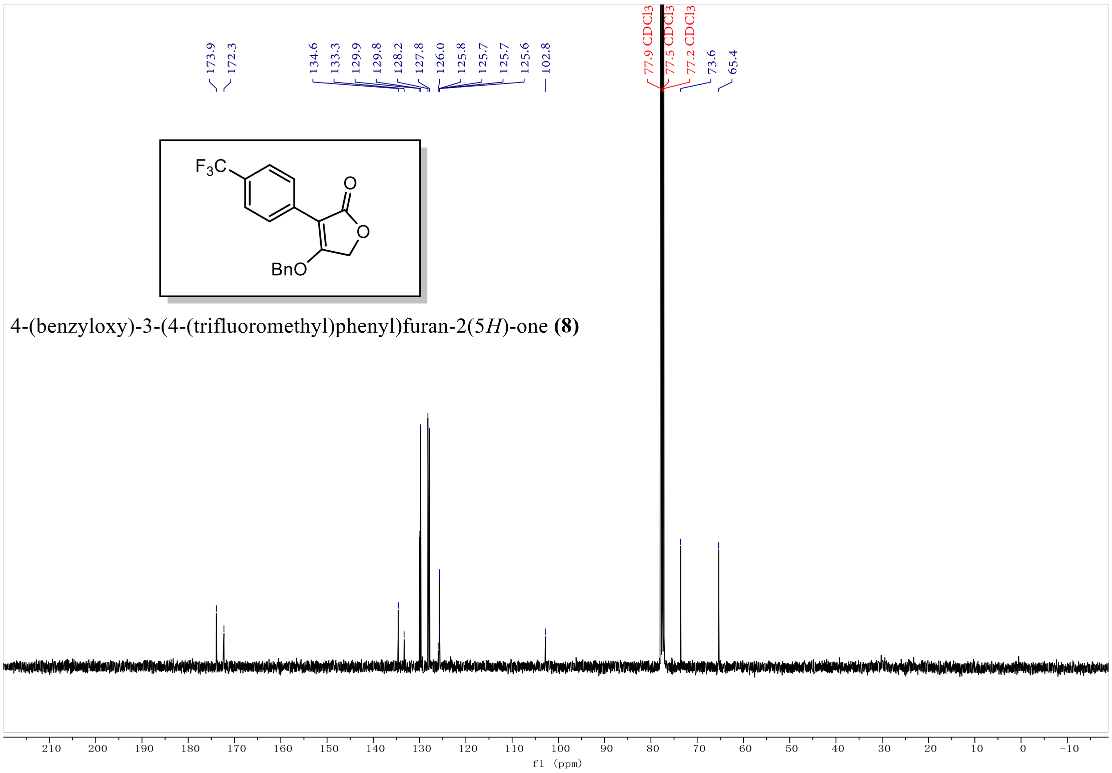

4-(benzyloxy)-3-(4-(trifluoromethyl)phenyl)furan-2(5*H*)-one **(8)**

^{19}F NMR (565 MHz, Chloroform-*d*)

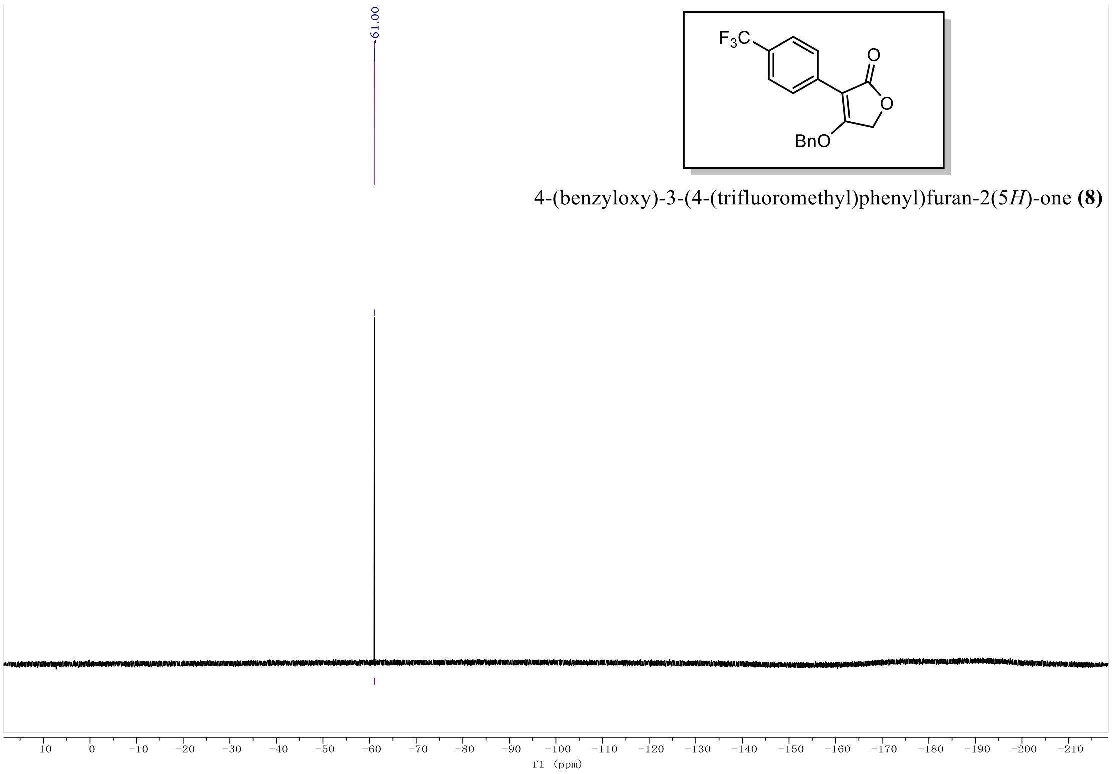

4-(benzyloxy)-3-(4-(trifluoromethyl)phenyl)furan-2(5*H*)-one **(8)**

^{1}H NMR (400 MHz, Chloroform-*d*)

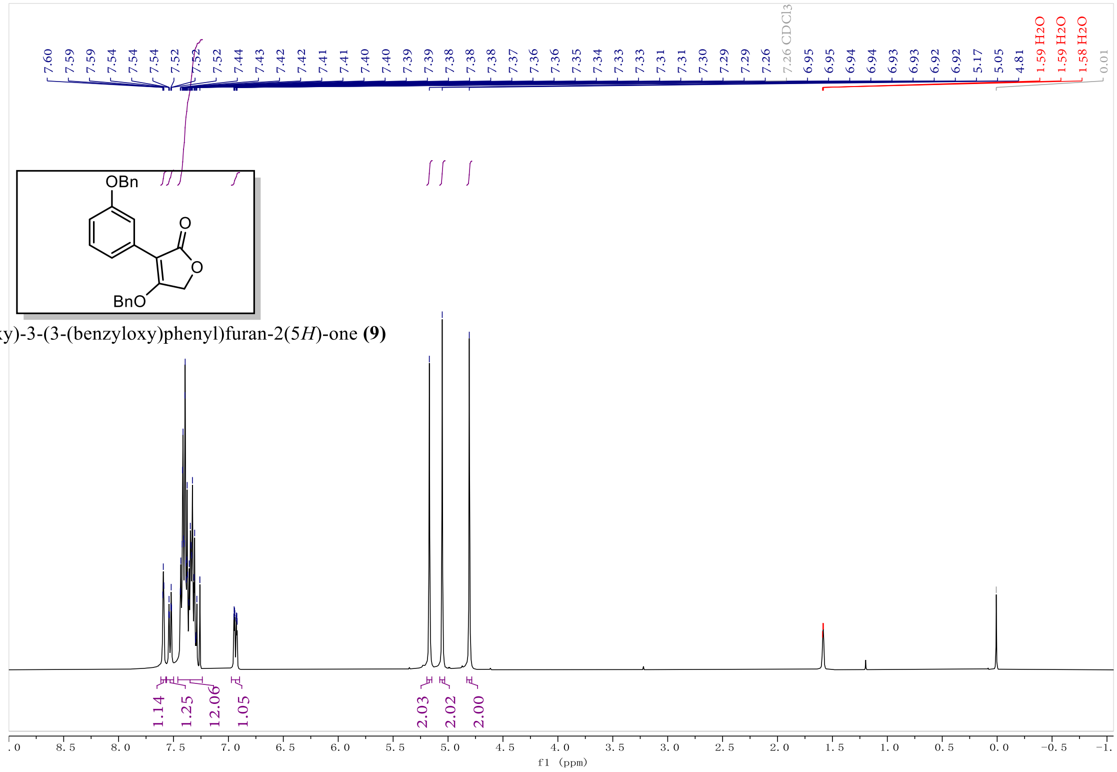

4-(benzyloxy)-3-(3-(benzyloxy)phenyl)furan-2(5*H*)-one **(9)**

^{13}C NMR (101 MHz, Chloroform-*d*)

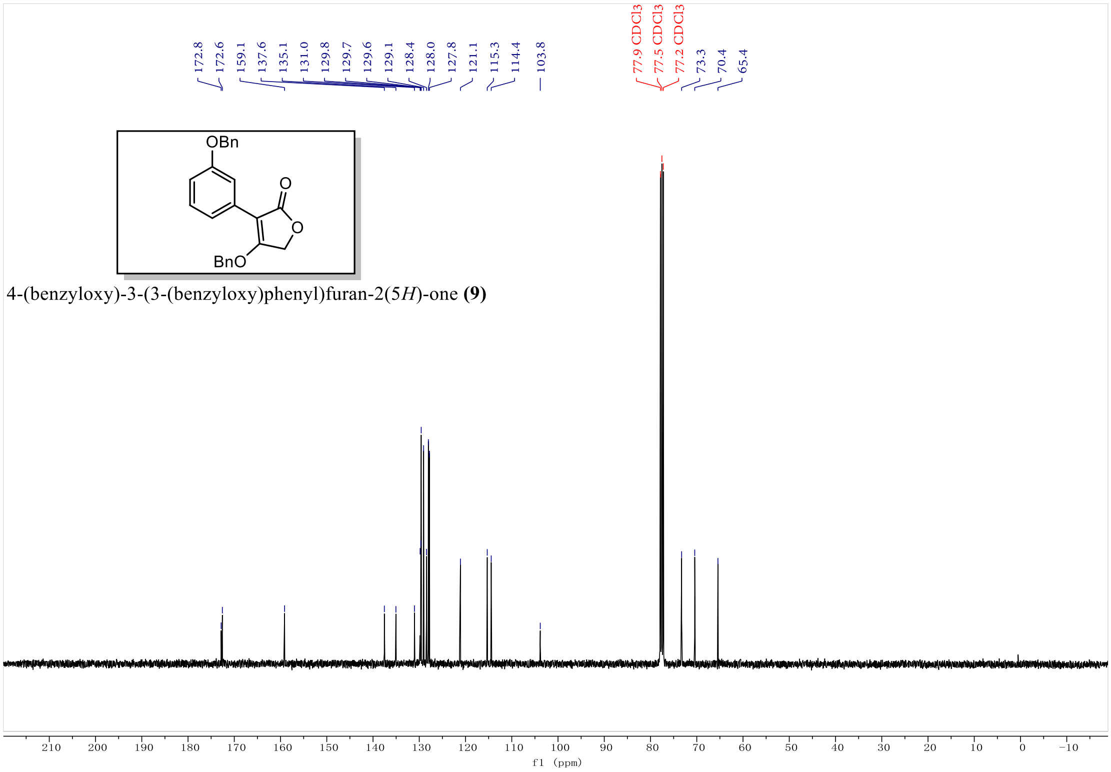

4-(benzyloxy)-3-(3-(benzyloxy)phenyl)furan-2(5*H*)-one **(9)**

^{1}H NMR (600 MHz, Chloroform-d)

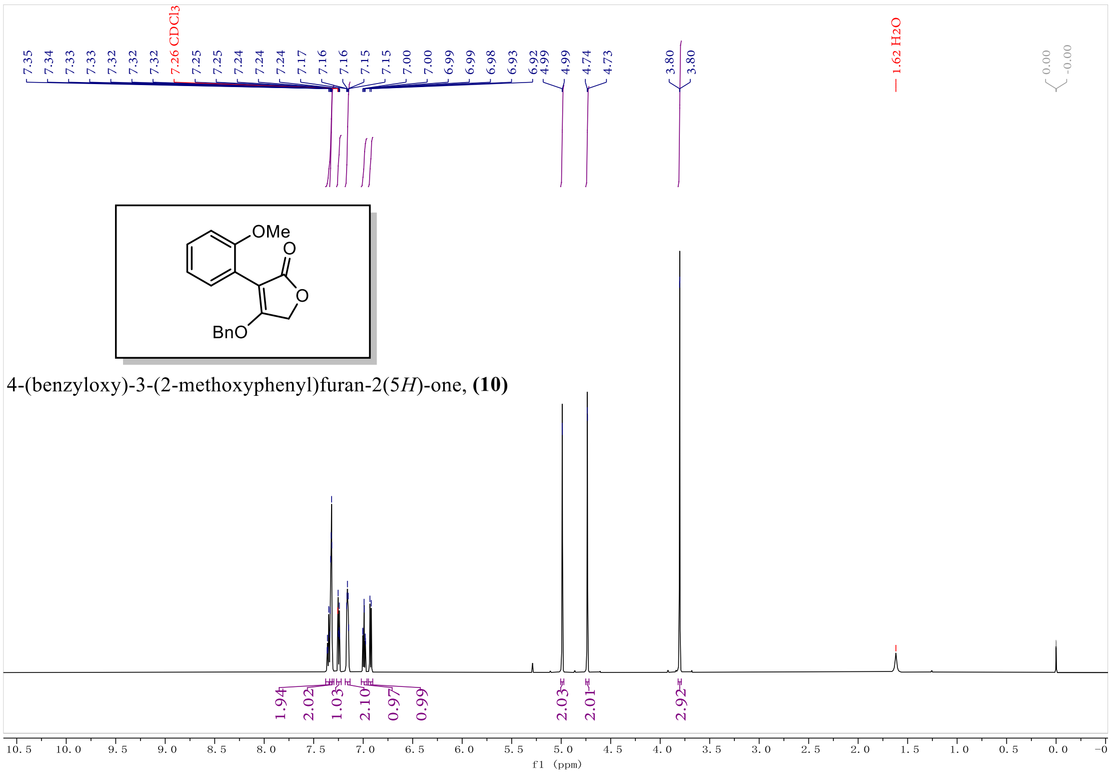

^{13}C NMR (101 MHz, Chloroform-d)

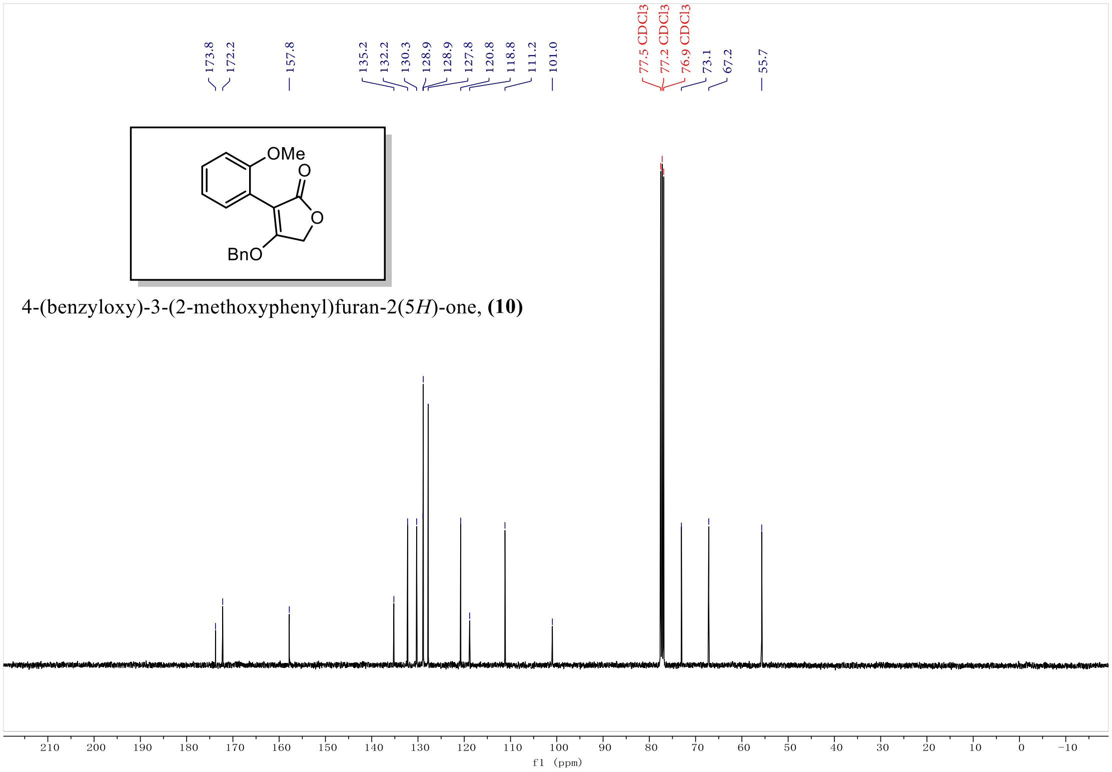

^{1}H NMR (600 MHz, Chloroform-d)

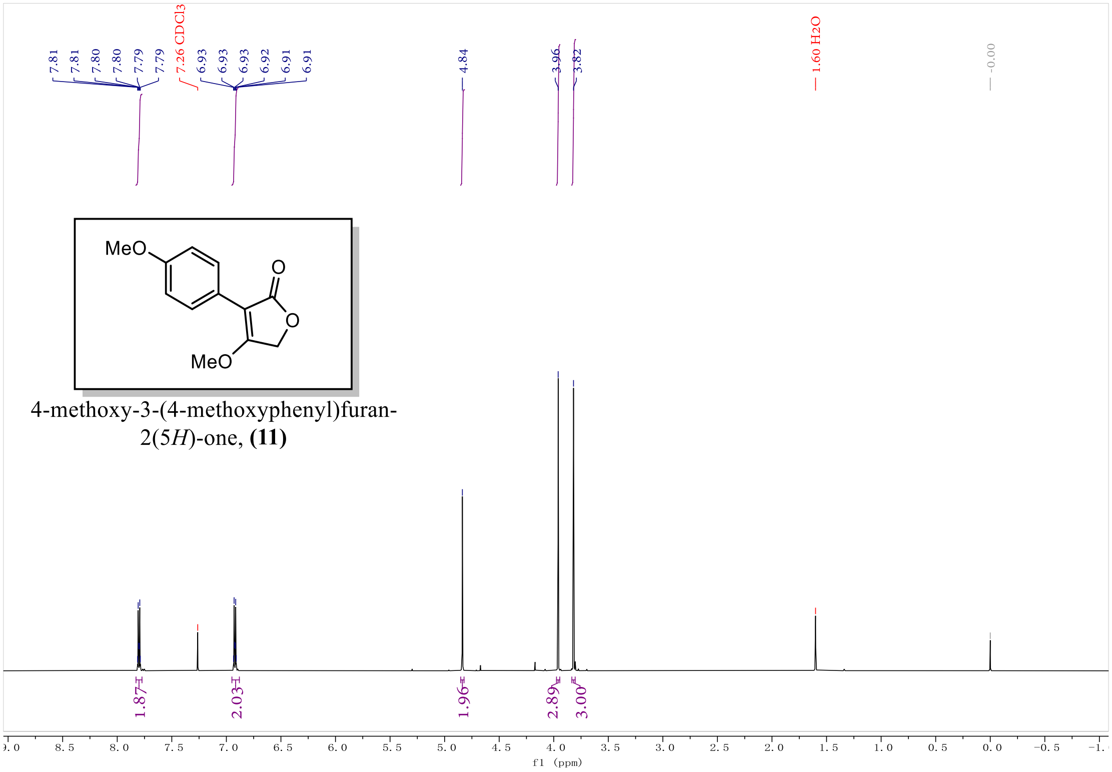

4-methoxy-3-(4-methoxyphenyl)furan-2(5*H*)-one, **(11)**

^{1}H NMR (400 MHz, Chloroform-*d*)

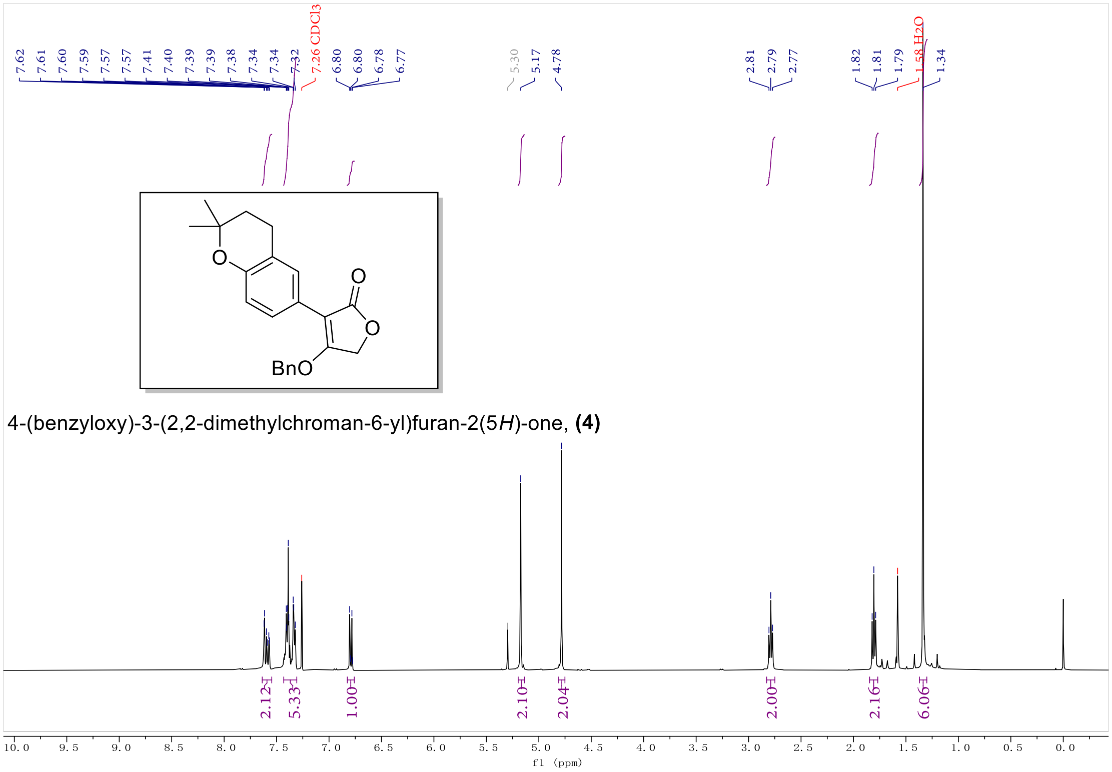

^{13}C NMR (101 MHz, Chloroform-*d*)

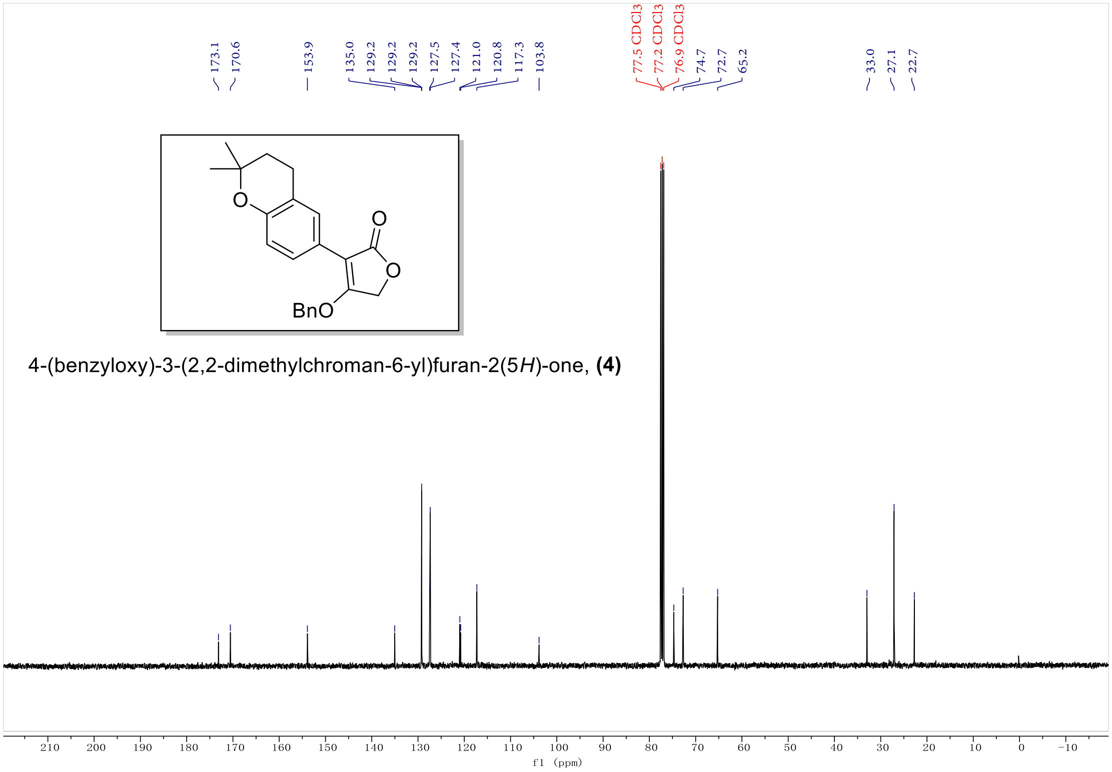

^{1}H NMR (600 MHz, Chloroform-*d*)

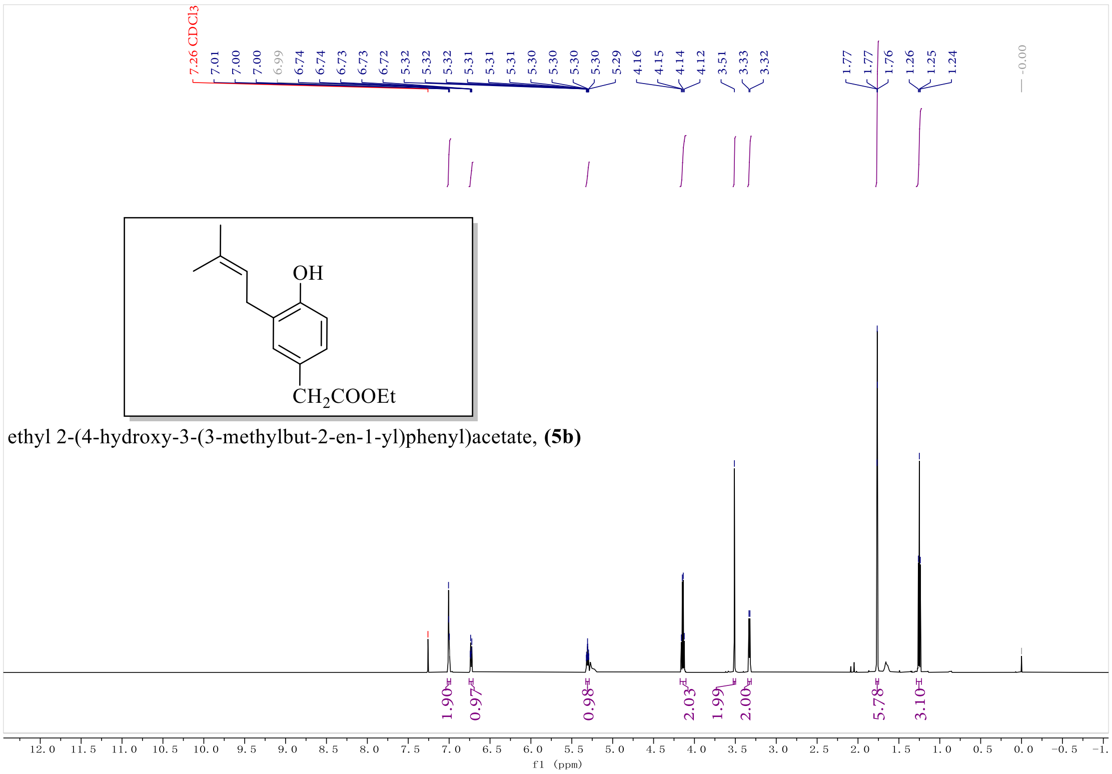

ethyl 2-(4-hydroxy-3-(3-methylbut-2-en-1-yl)phenyl)acetate, **(5b)**

^{13}C NMR (101 MHz, Chloroform-*d*)

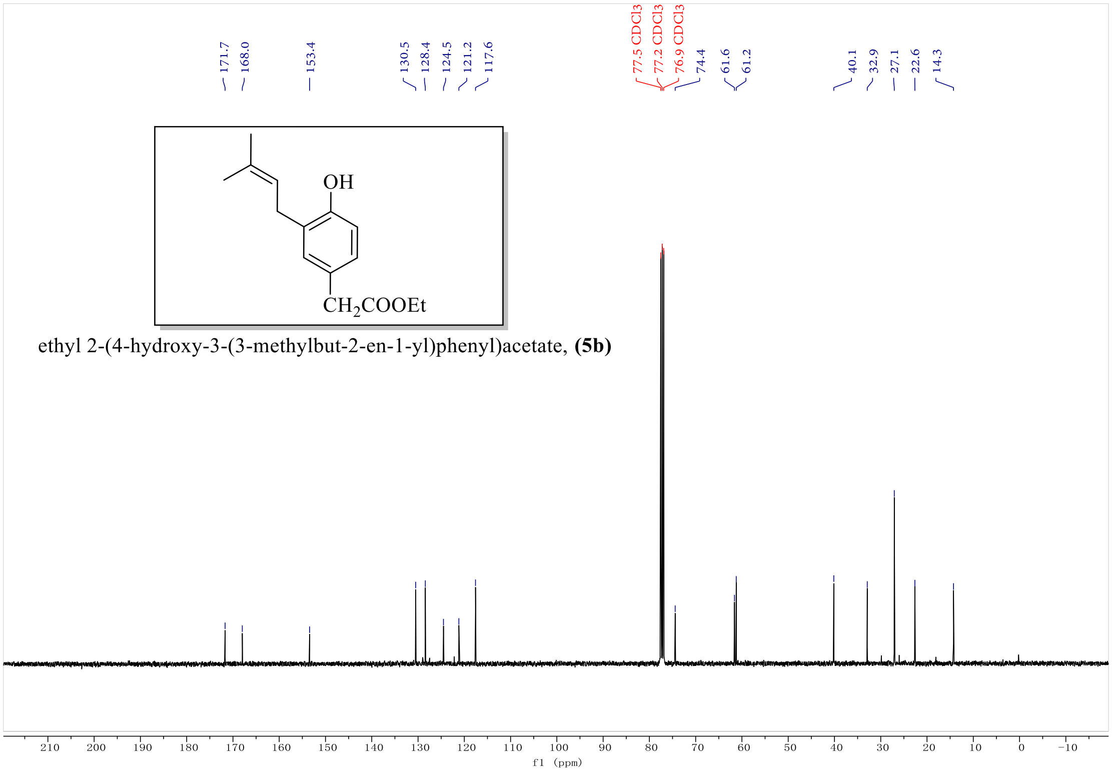

ethyl 2-(4-hydroxy-3-(3-methylbut-2-en-1-yl)phenyl)acetate, **(5b)**

^{1}H NMR (400 MHz, Chloroform-*d*)

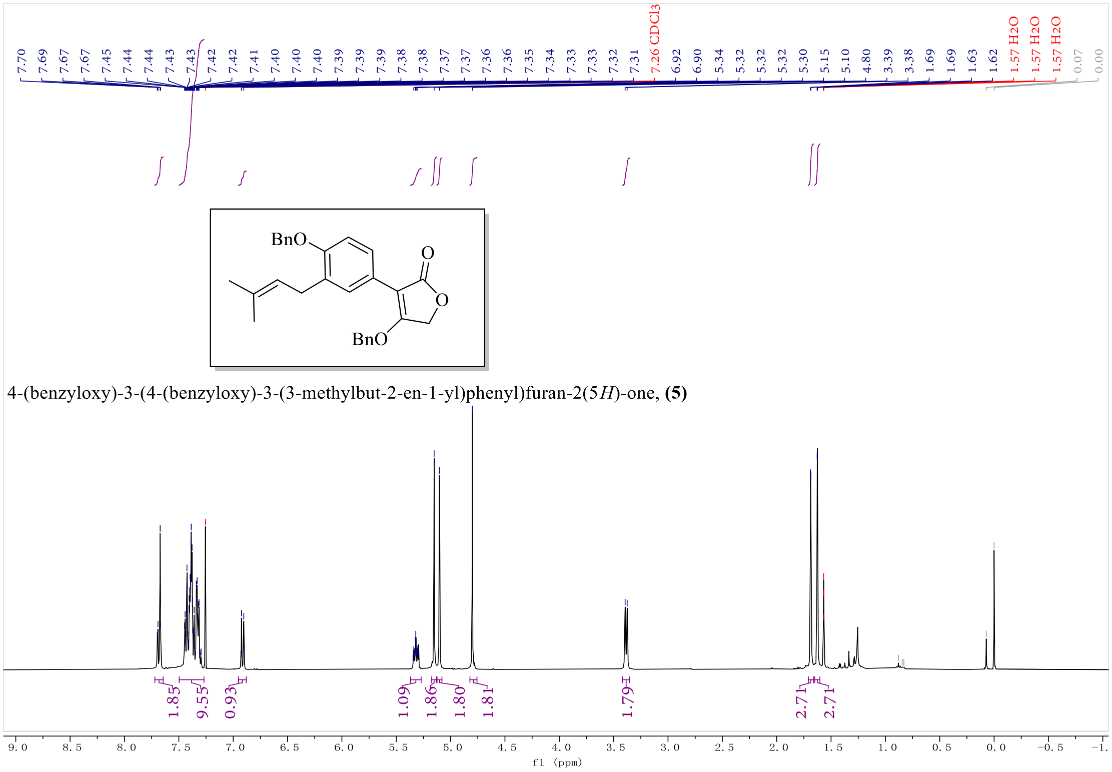

4-(benzyloxy)-3-(4-(benzyloxy)-3-(3-methylbut-2-en-1-yl)phenyl)furan-2(5*H*)-one, **(5)**

^{13}C NMR (101 MHz, Chloroform-*d*)

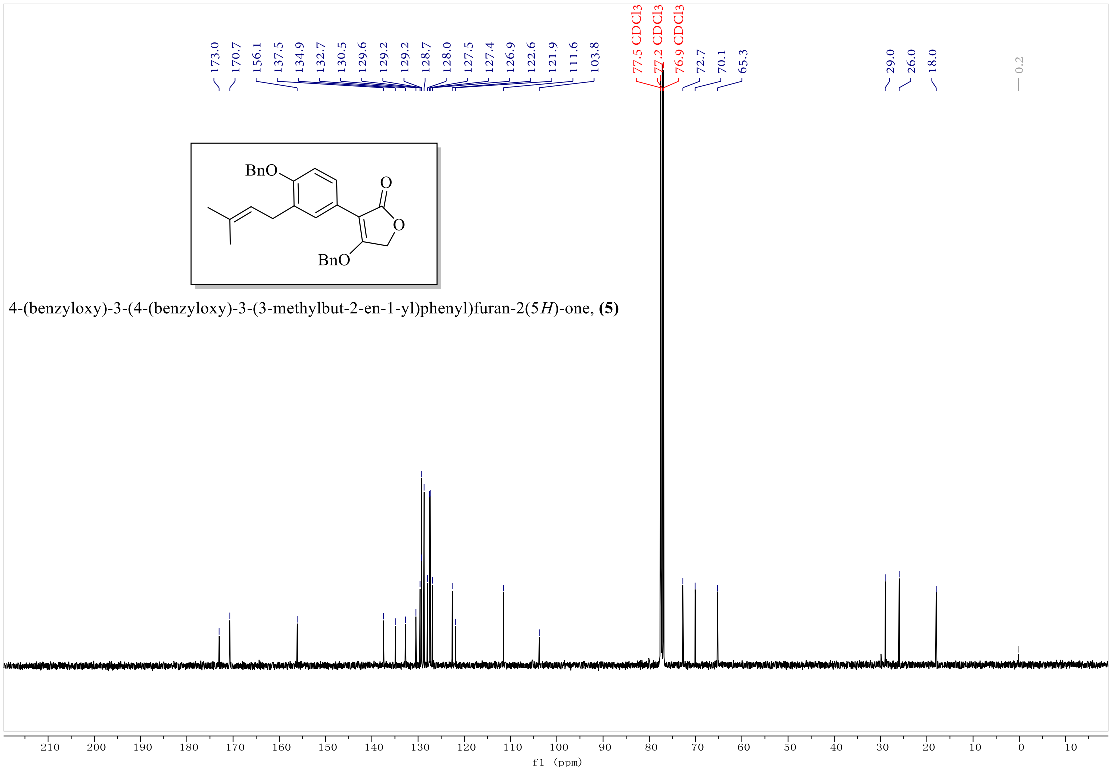

4-(benzyloxy)-3-(4-(benzyloxy)-3-(3-methylbut-2-en-1-yl)phenyl)furan-2(5*H*)-one, **(5)**

^{1}H NMR (400 MHz, Chloroform-*d*)

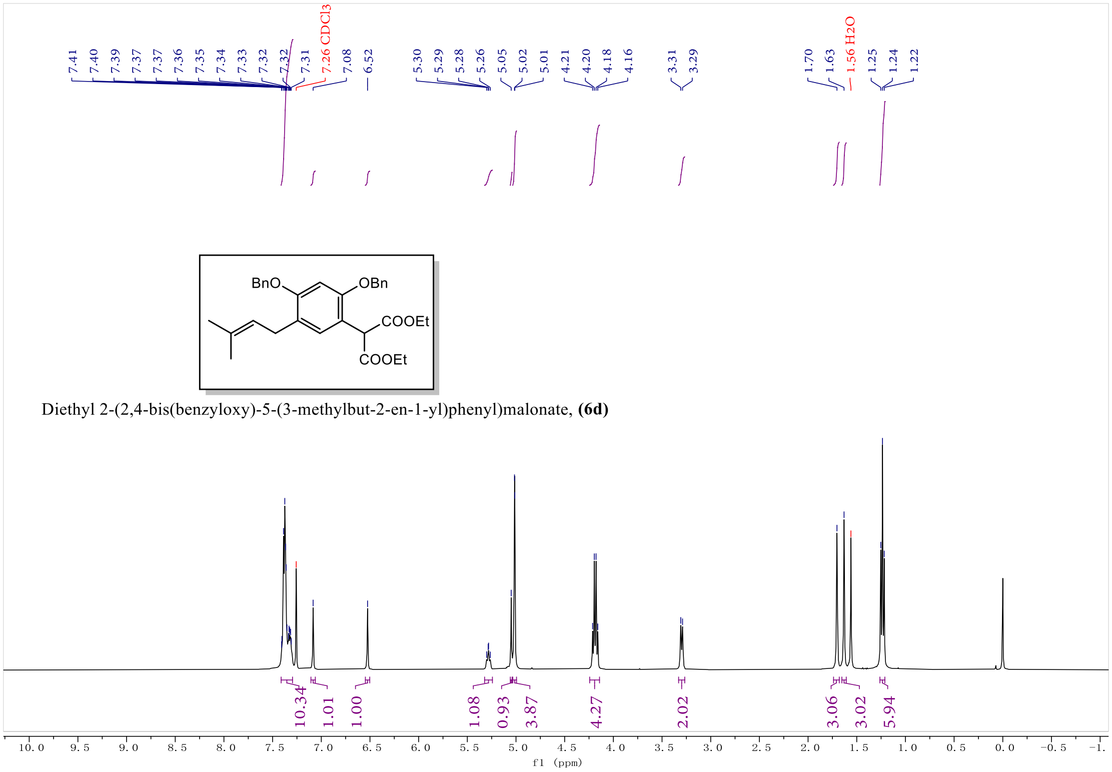

Diethyl 2-(2,4-bis(benzyloxy)-5-(3-methylbut-2-en-1-yl)phenyl)malonate, **(6d)**

^{13}C NMR (151 MHz, Chloroform-*d*)

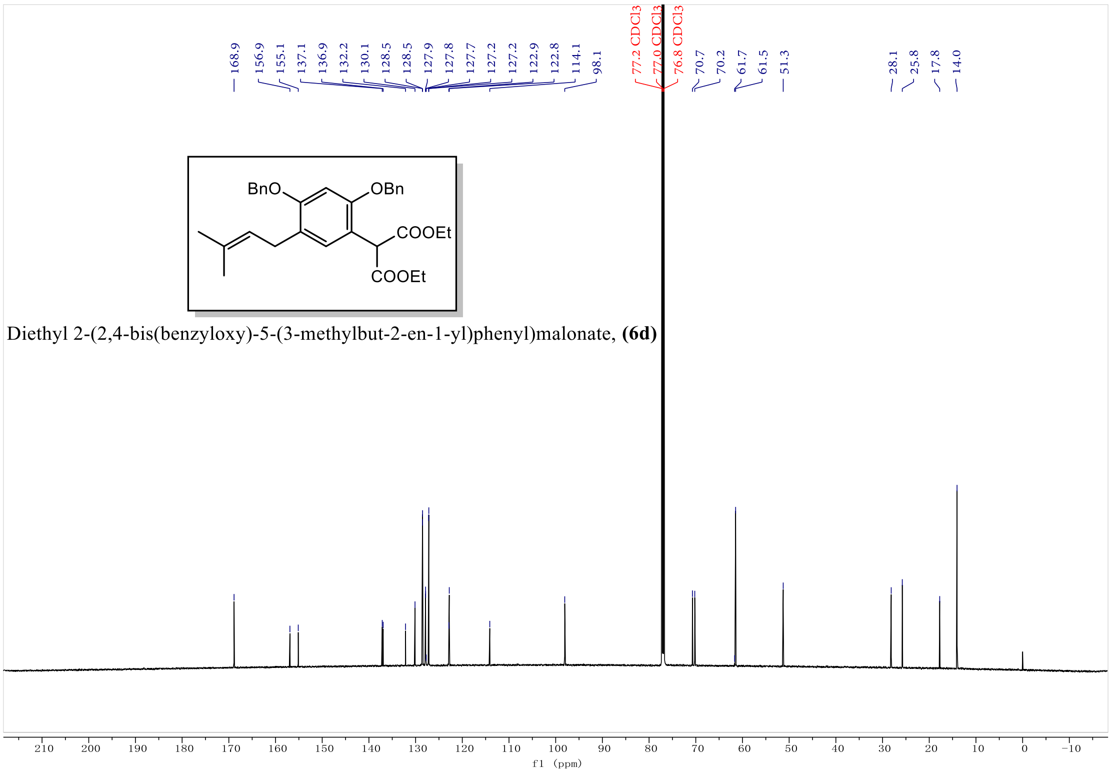

Diethyl 2-(2,4-bis(benzyloxy)-5-(3-methylbut-2-en-1-yl)phenyl)malonate, **(6d)**

^{1}H NMR (400 MHz, DMSO-d_6)

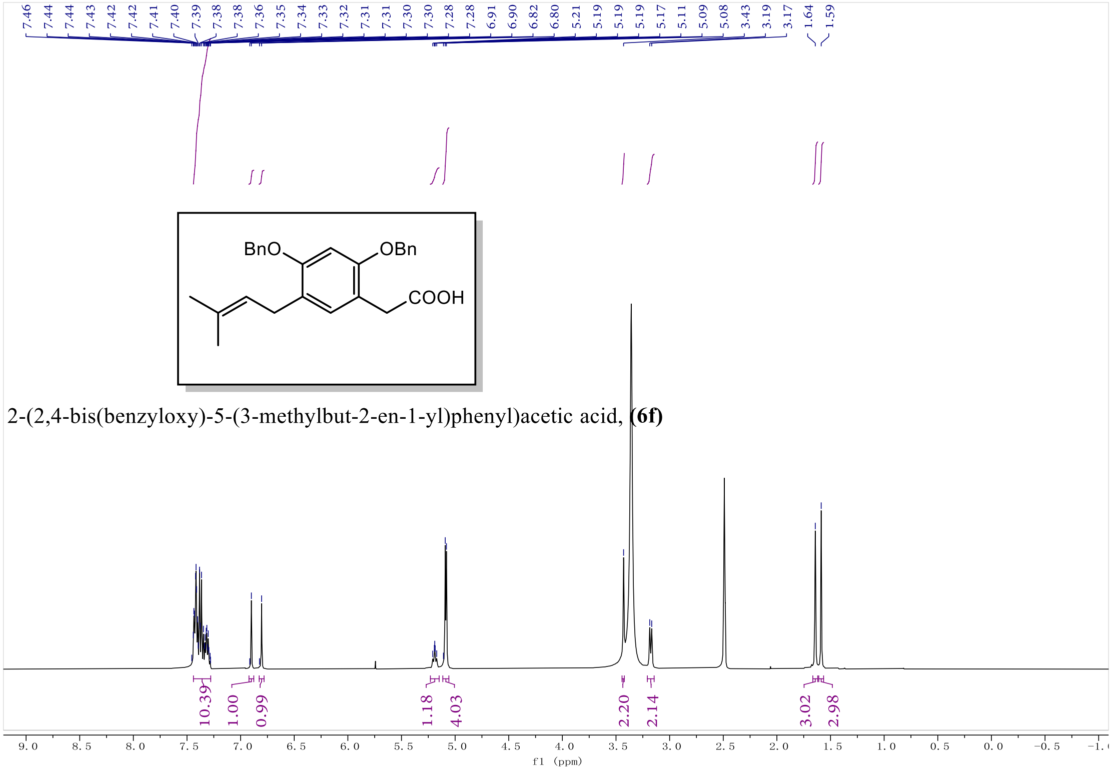

2-(2,4-bis(benzyloxy)-5-(3-methylbut-2-en-1-yl)phenyl)acetic acid, **(6f)**

^{13}C NMR (101 MHz, DMSO-d_6)

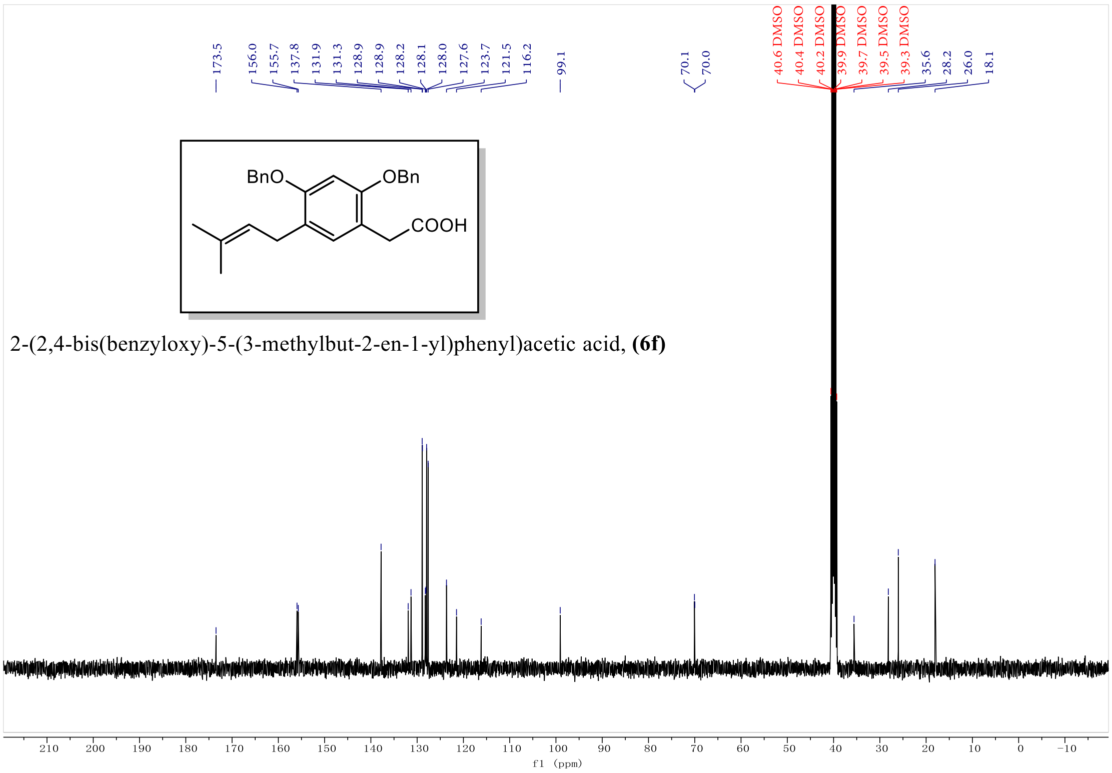

2-(2,4-bis(benzyloxy)-5-(3-methylbut-2-en-1-yl)phenyl)acetic acid, **(6f)**

^{1}H NMR (400 MHz, Chloroform-*d*)

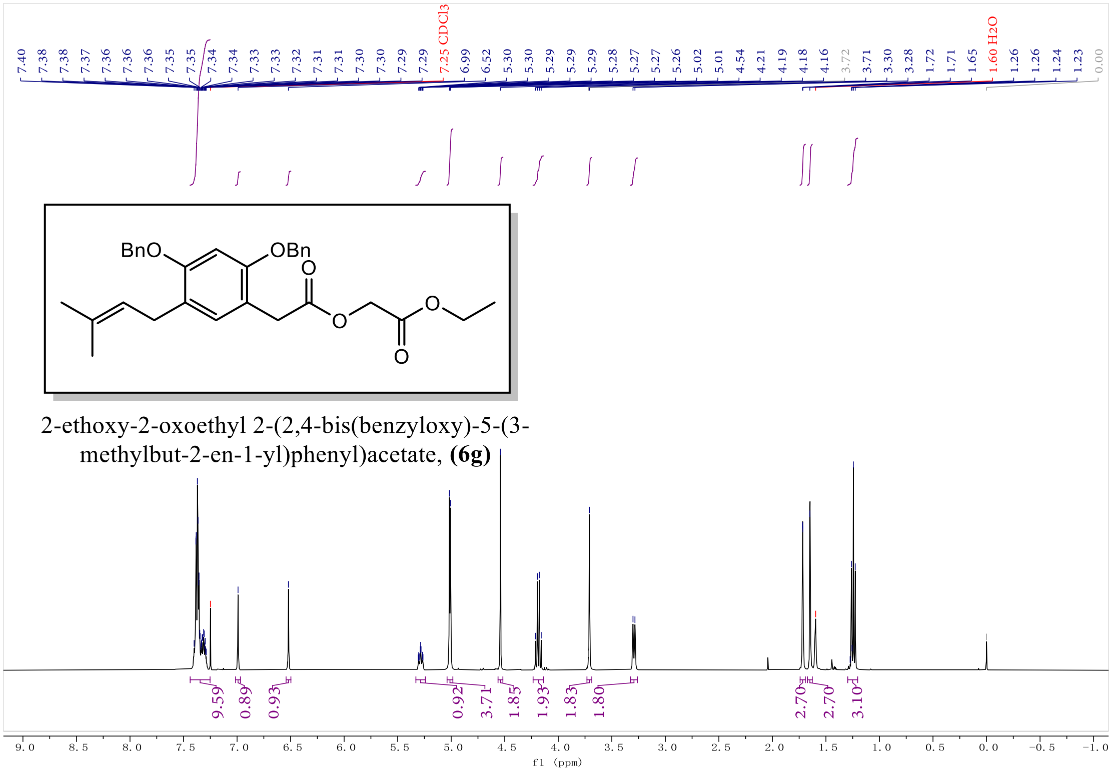

2-ethoxy-2-oxoethyl 2-(2,4-bis(benzyloxy)-5-(3-methylbut-2-en-1-yl)phenyl)acetate, **(6g)**

^{13}C NMR (101 MHz, Chloroform-*d*)

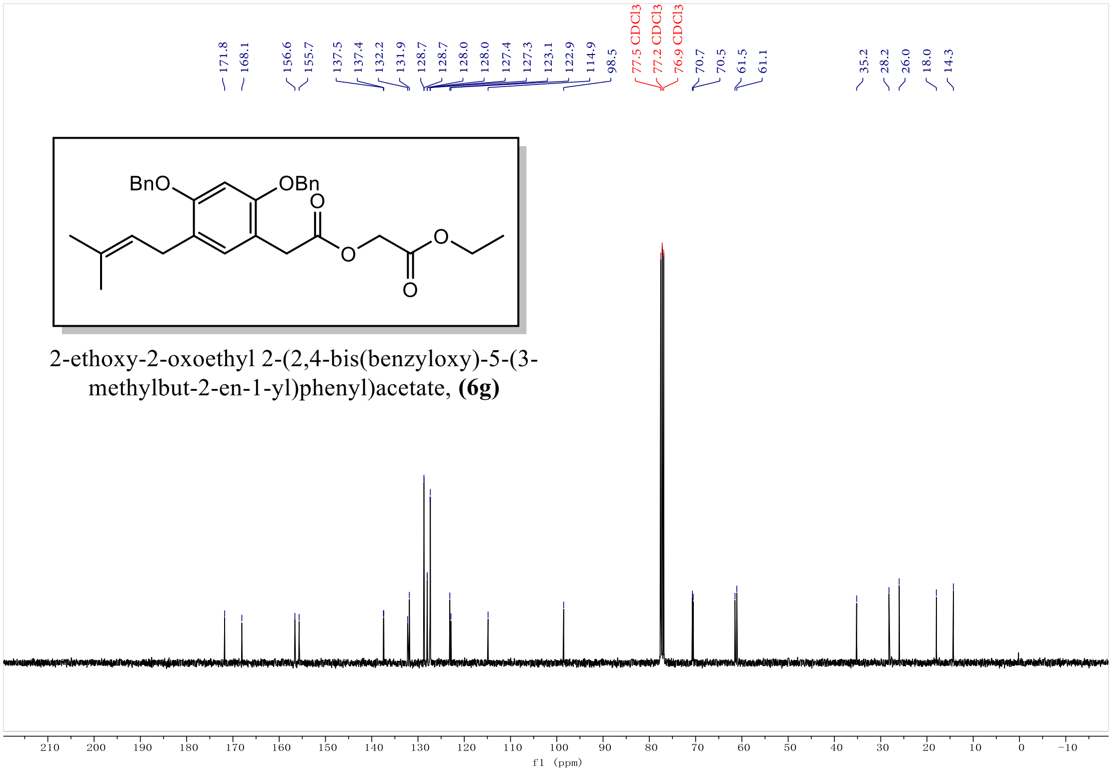

2-ethoxy-2-oxoethyl 2-(2,4-bis(benzyloxy)-5-(3-methylbut-2-en-1-yl)phenyl)acetate, **(6g)**

^{1}H NMR (400 MHz, Chloroform-*d*)

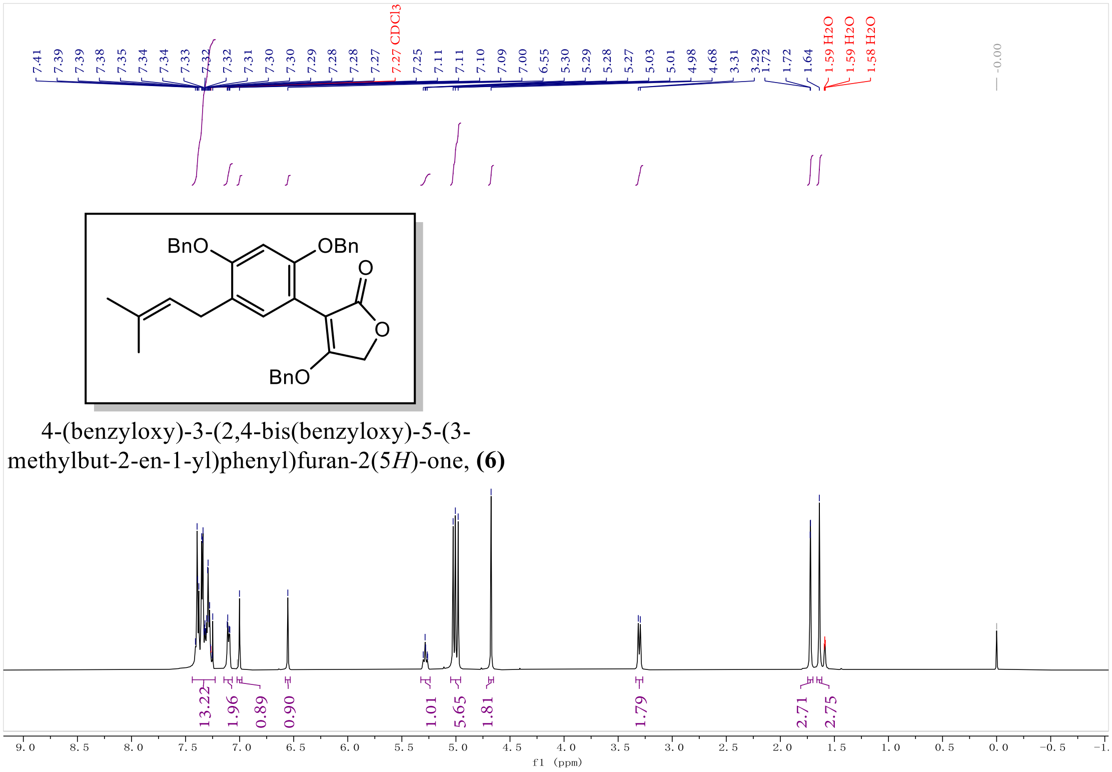

4-(benzyloxy)-3-(2,4-bis(benzyloxy)-5-(3-methylbut-2-en-1-yl)phenyl)furan-2(5*H*)-one, **(6)**

^{13}C NMR (151 MHz, Chloroform-*d*)

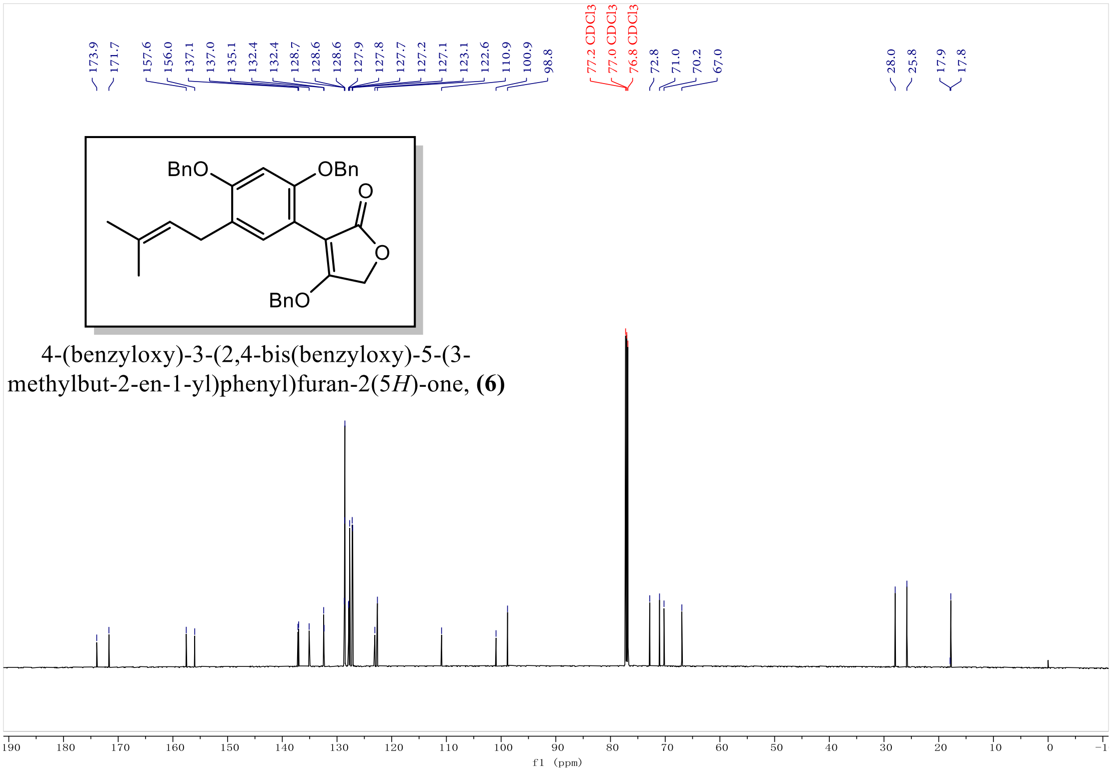

4-(benzyloxy)-3-(2,4-bis(benzyloxy)-5-(3-methylbut-2-en-1-yl)phenyl)furan-2(5*H*)-one, **(6)**

^{1}H NMR (400 MHz, Chloroform-*d*)

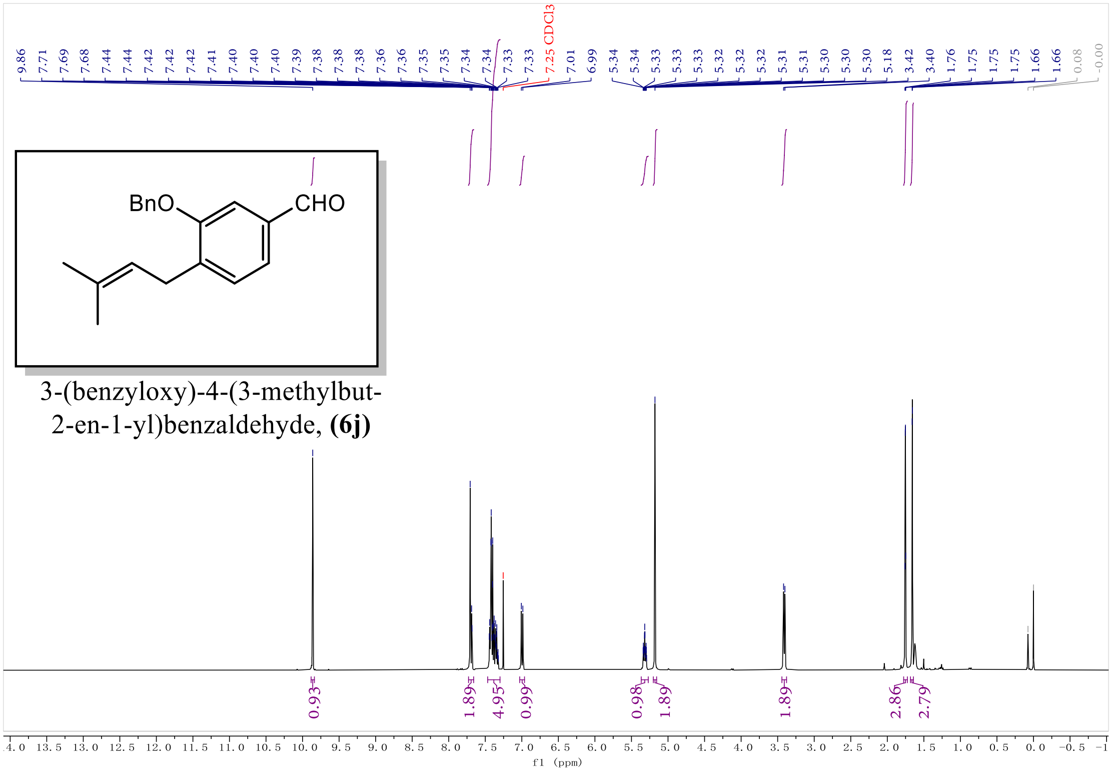

3-(benzyloxy)-4-(3-methylbut-2-en-1-yl)benzaldehyde, **(6j)**

^{1}H NMR (600 MHz, Chloroform-*d*)

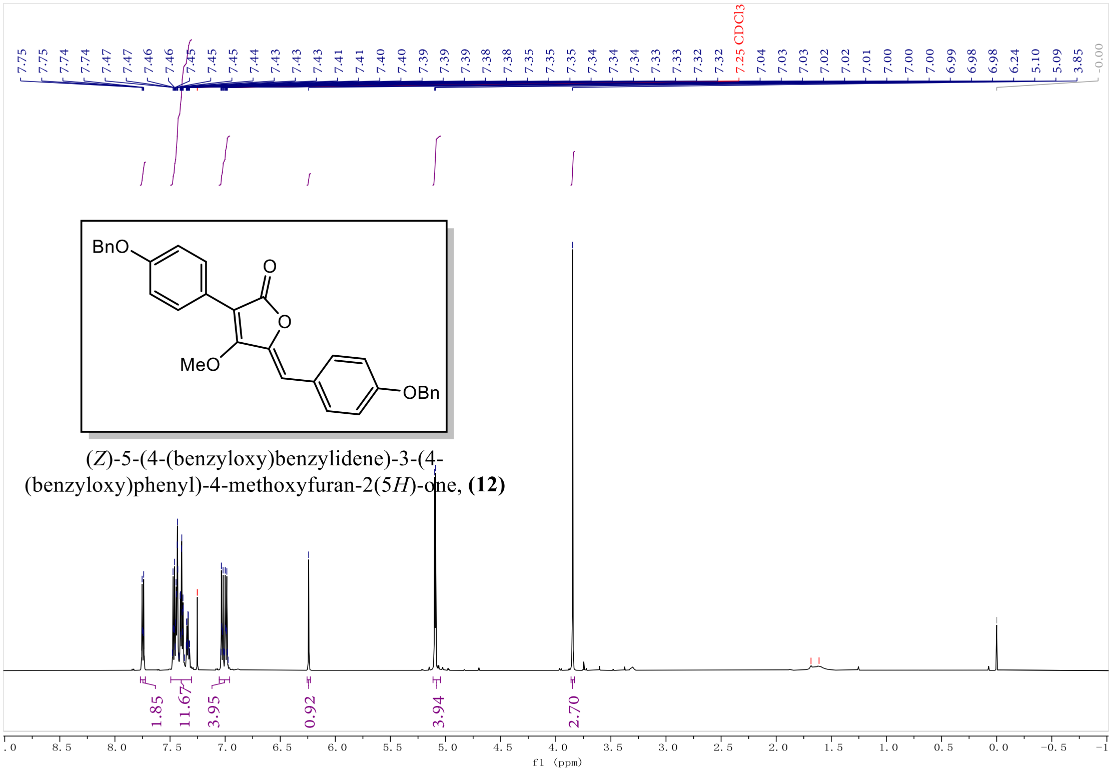

(*Z*)-5-(4-(benzyloxy)benzylidene)-3-(4-(benzyloxy)phenyl)-4-methoxyfuran-2(5*H*)-one, **(12)**

^{13}C NMR (101 MHz, Chloroform-*d*)

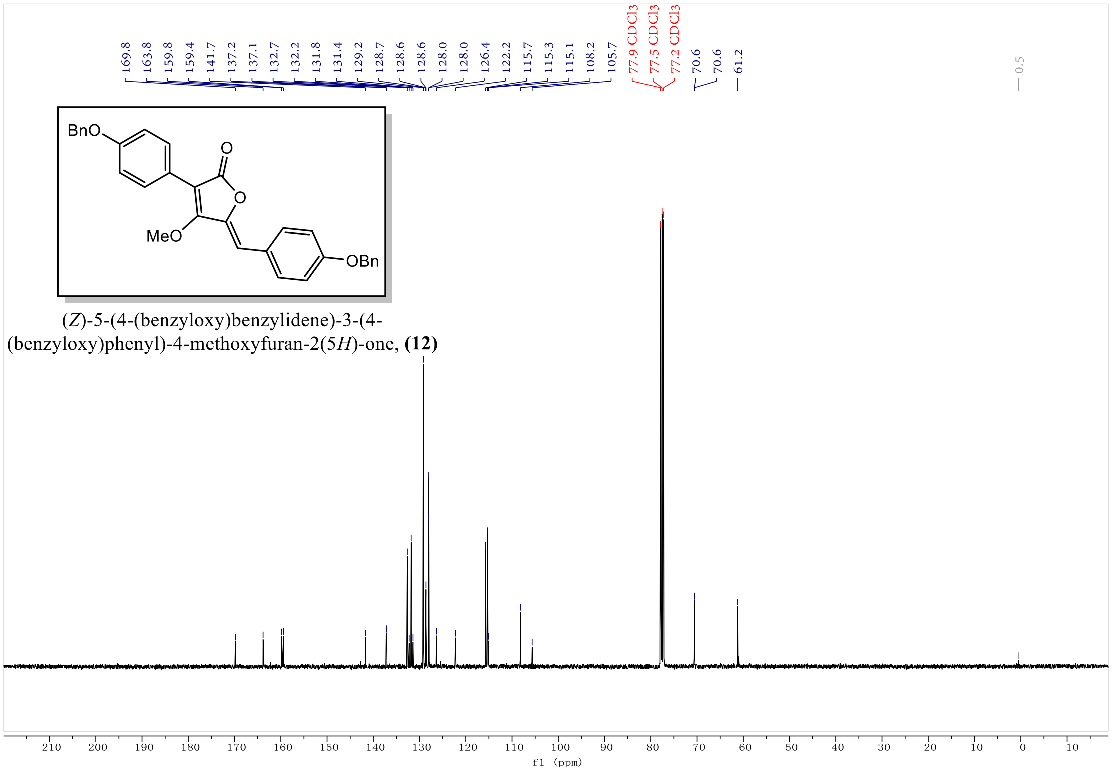

(*Z*)-5-(4-(benzyloxy)benzylidene)-3-(4-(benzyloxy)phenyl)-4-methoxyfuran-2(5*H*)-one, **(12)**

^{1}H NMR (600 MHz, Chloroform-*d*)

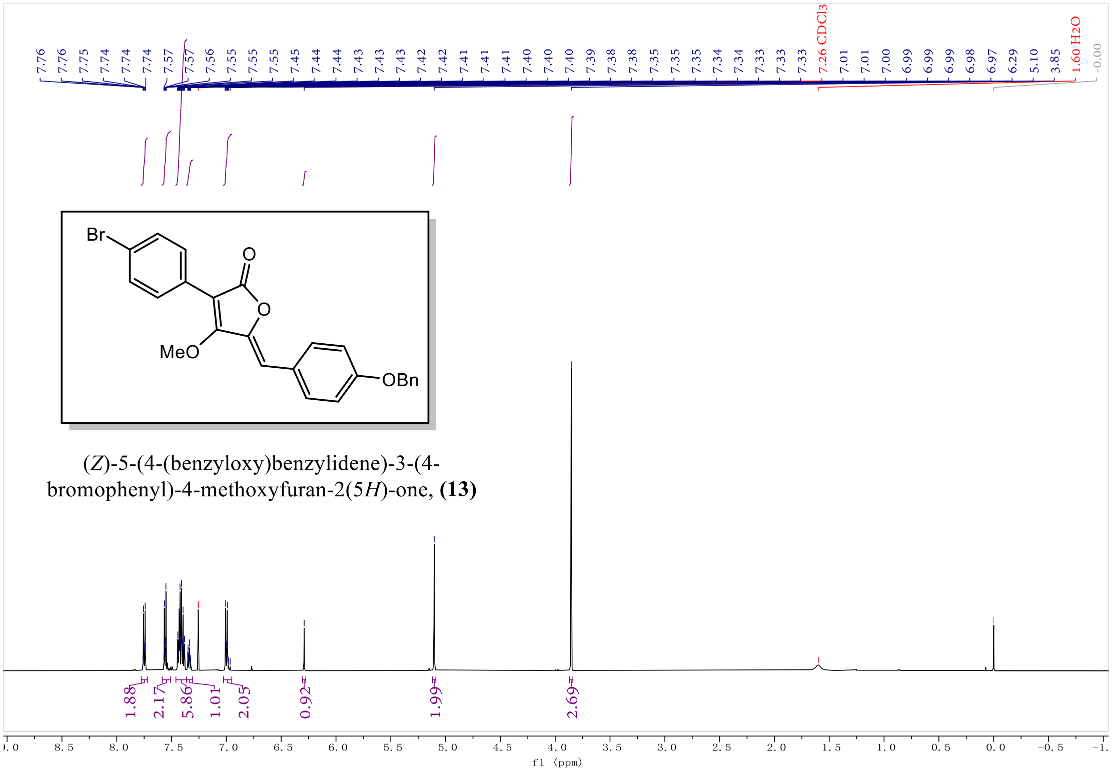

(*Z*)-5-(4-(benzyloxy)benzylidene)-3-(4-bromophenyl)-4-methoxyfuran-2(5*H*)-one, **(13)**

^{13}C NMR (151 MHz, Chloroform-*d*)

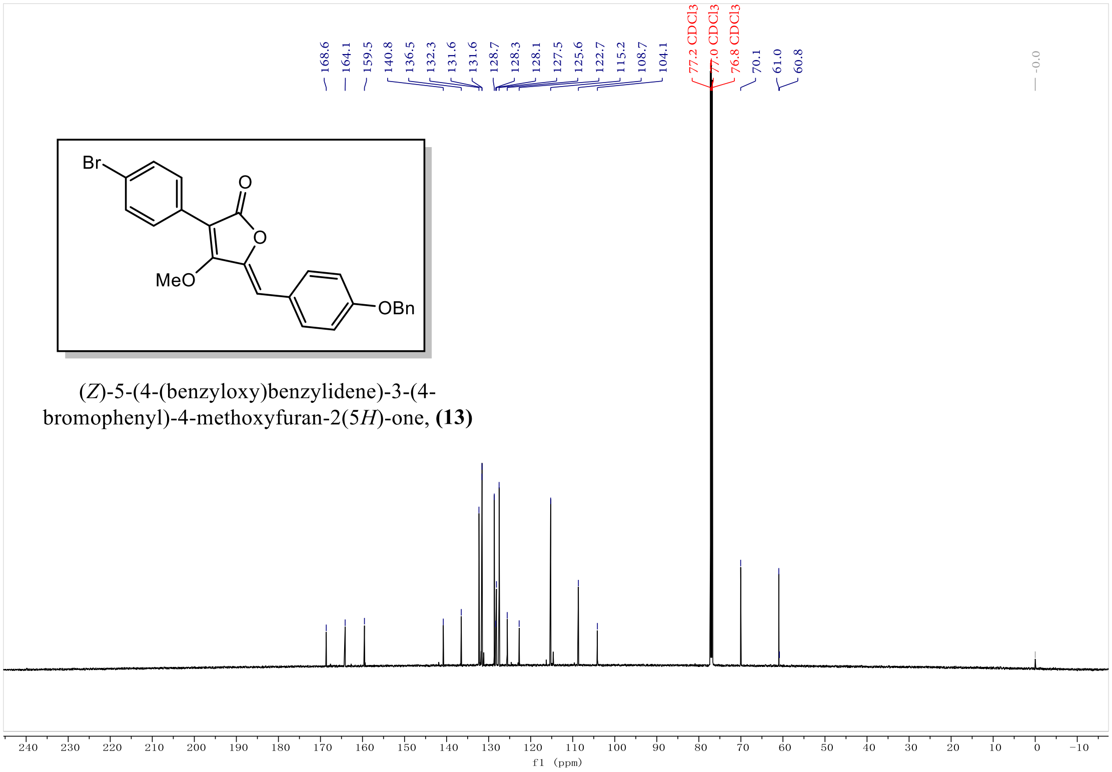

(*Z*)-5-(4-(benzyloxy)benzylidene)-3-(4-bromophenyl)-4-methoxyfuran-2(5*H*)-one, **(13)**

^{1}H NMR (400 MHz, Chloroform-*d*)

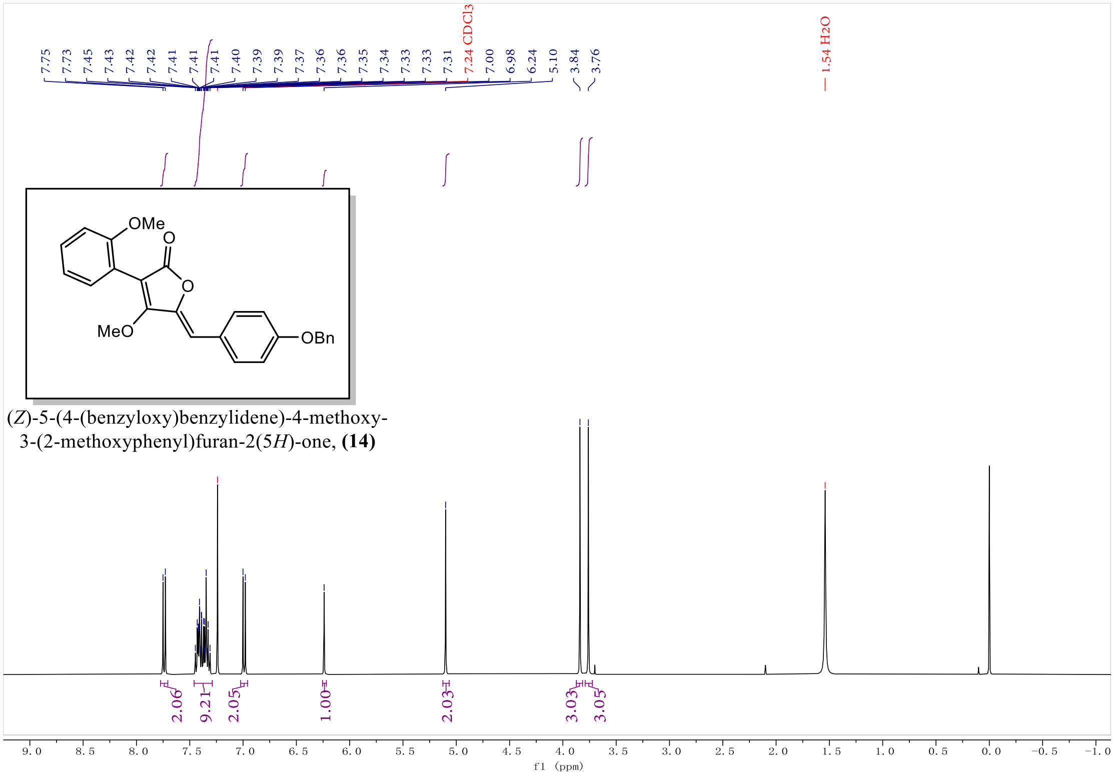

(*Z*)-5-(4-(benzyloxy)benzylidene)-4-methoxy-3-(2-methoxyphenyl)furan-2(5*H*)-one, **(14)**

^{13}C NMR (101 MHz, Chloroform-*d*)

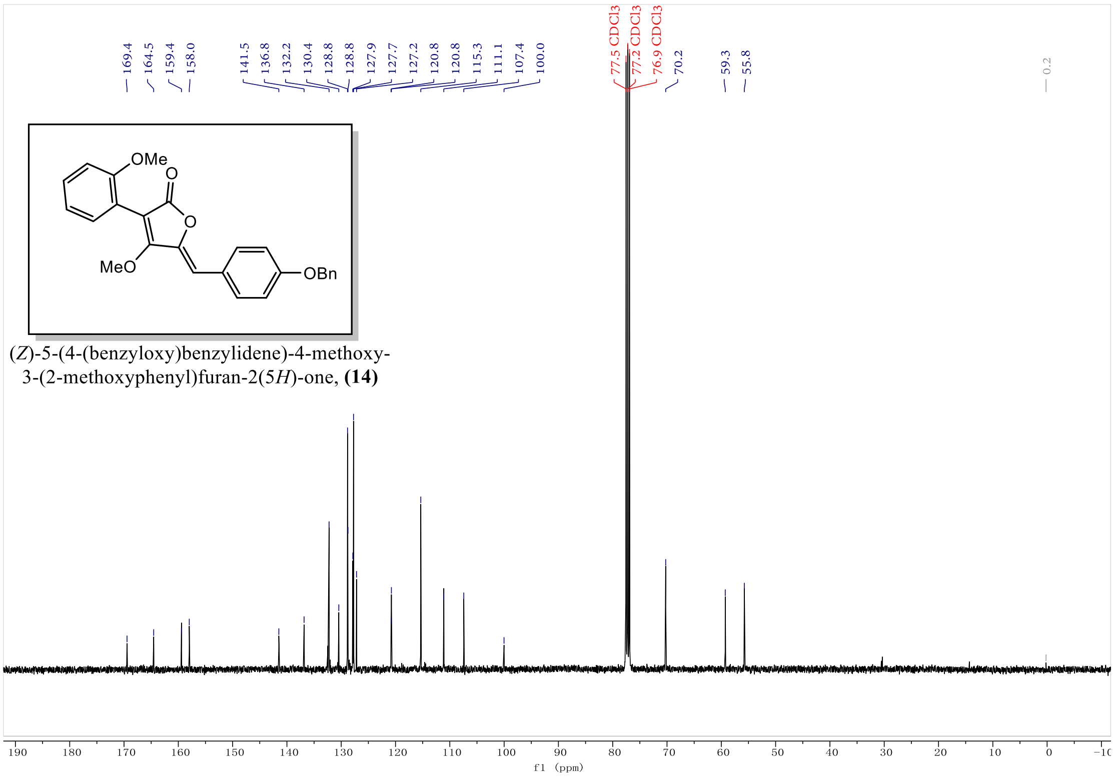

(*Z*)-5-(4-(benzyloxy)benzylidene)-4-methoxy-3-(2-methoxyphenyl)furan-2(5*H*)-one, **(14)**

^{1}H NMR (600 MHz, Chloroform-*d*)

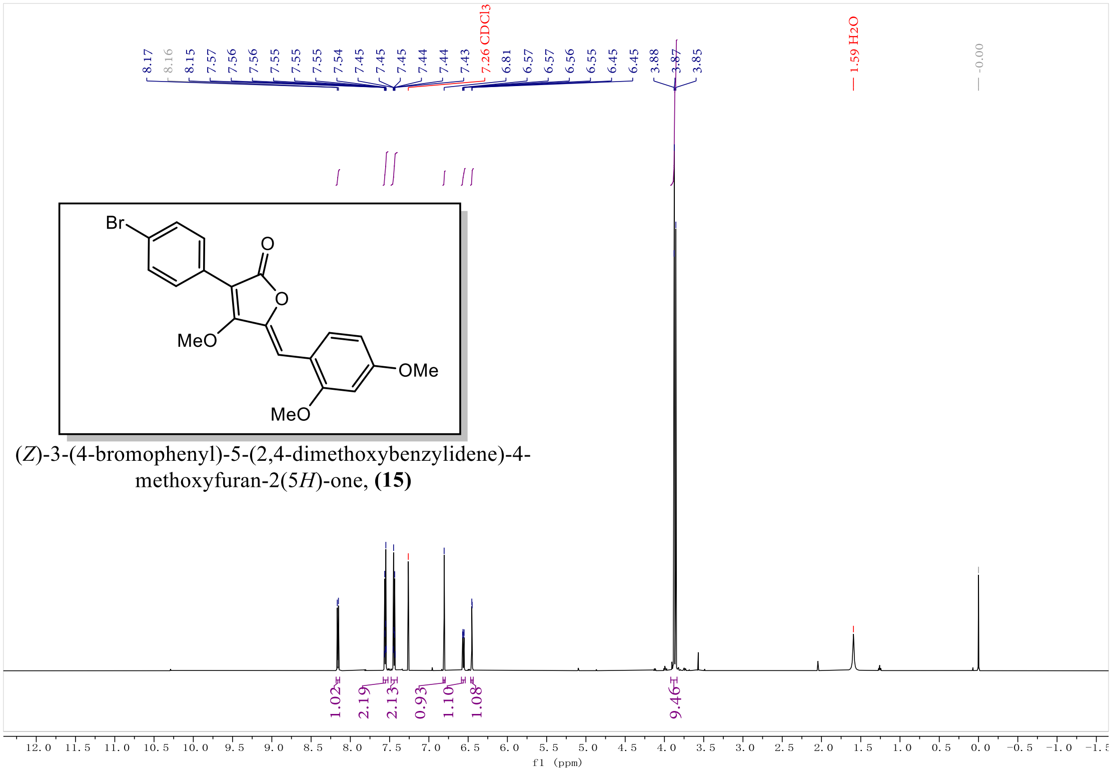

^{13}C NMR (101 MHz, Chloroform-*d*)

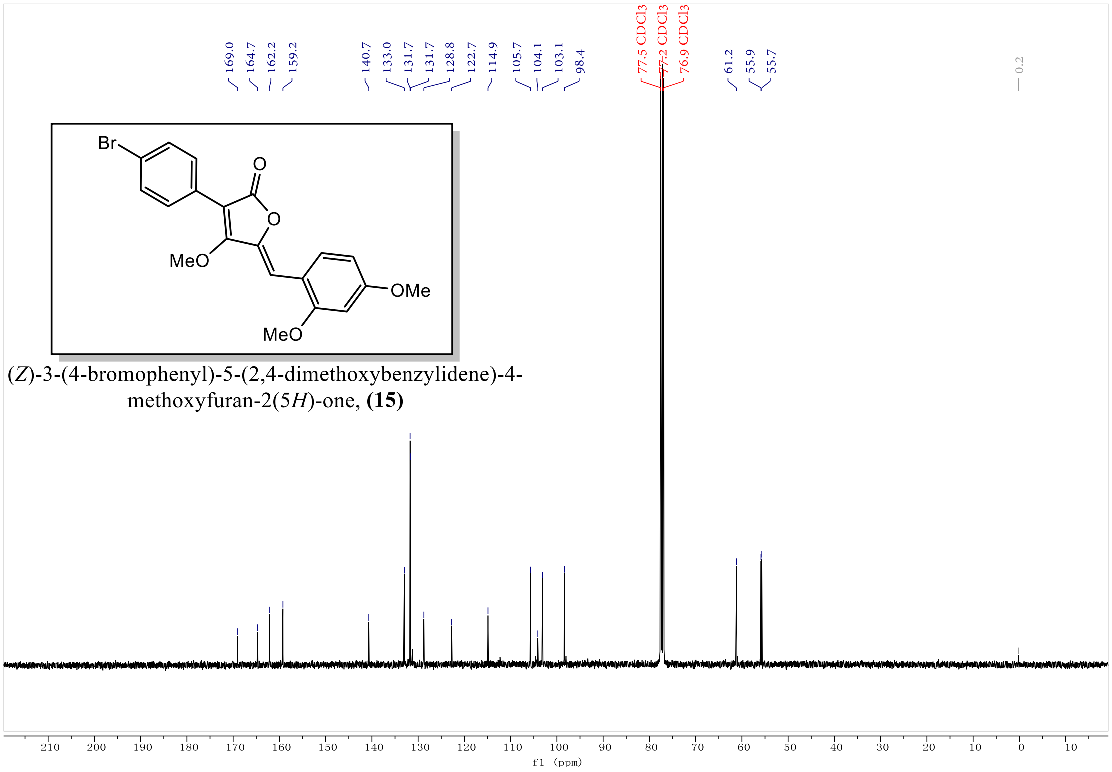

^{1}H NMR (400 MHz, Chloroform-*d*)

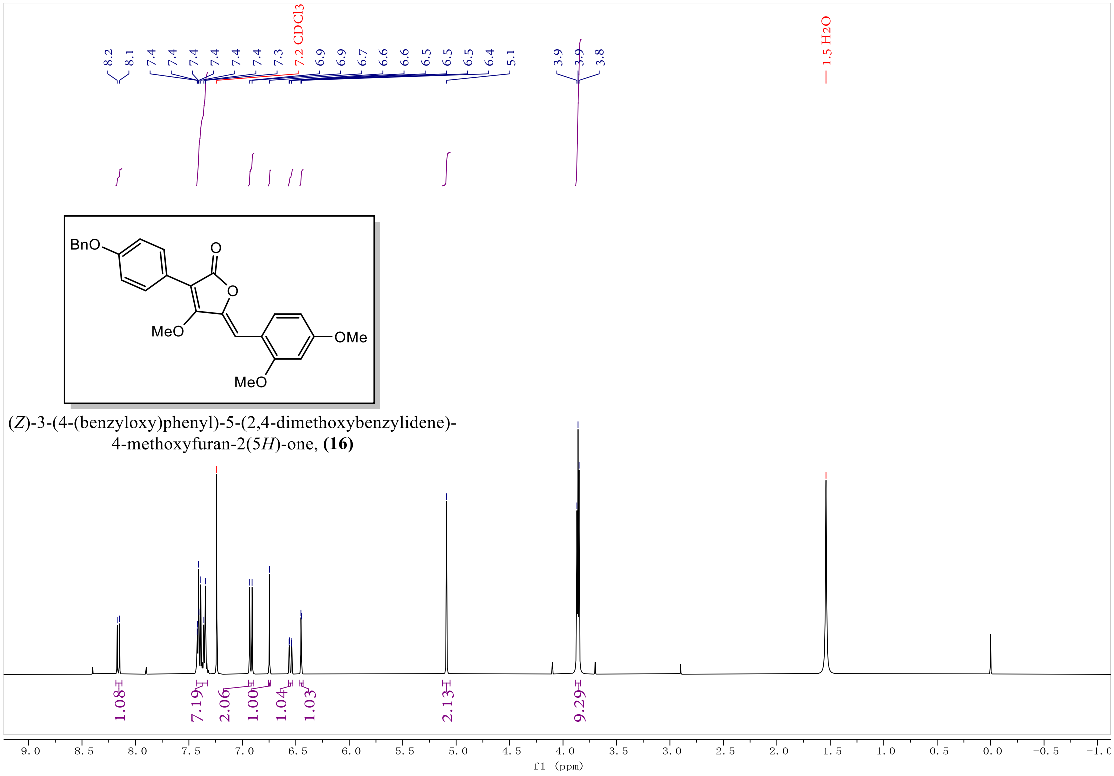

(*Z*)-3-(4-(benzyloxy)phenyl)-5-(2,4-dimethoxybenzylidene)-4-methoxyfuran-2(5*H*)-one, **(16)**

^{13}C NMR (101 MHz, Chloroform-*d*)

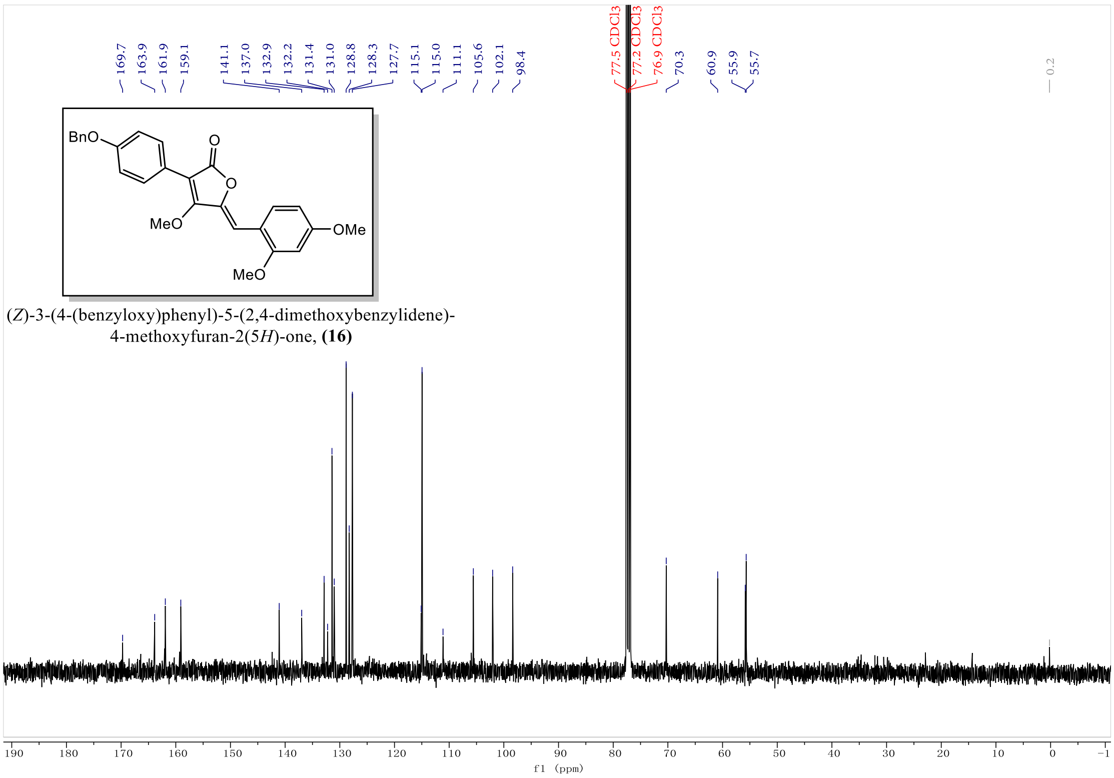

(*Z*)-3-(4-(benzyloxy)phenyl)-5-(2,4-dimethoxybenzylidene)-4-methoxyfuran-2(5*H*)-one, **(16)**

^{1}H NMR (400 MHz, Chloroform-*d*)

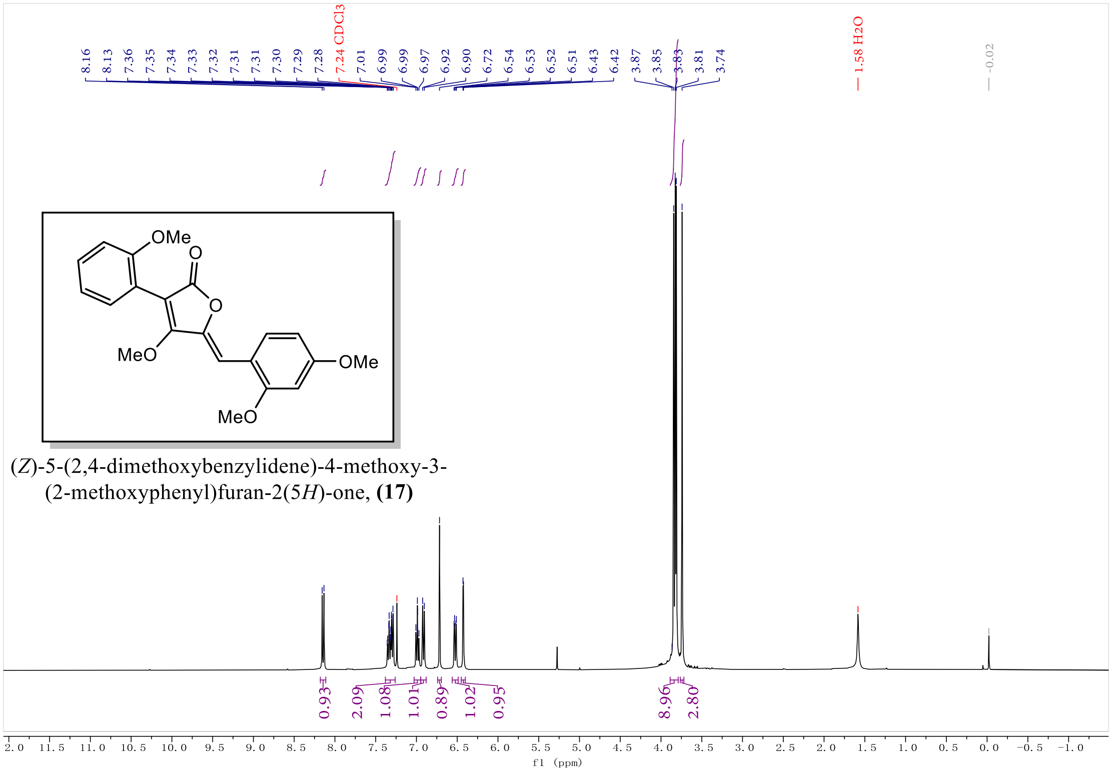

^{13}C NMR (101 MHz, Chloroform-*d*)

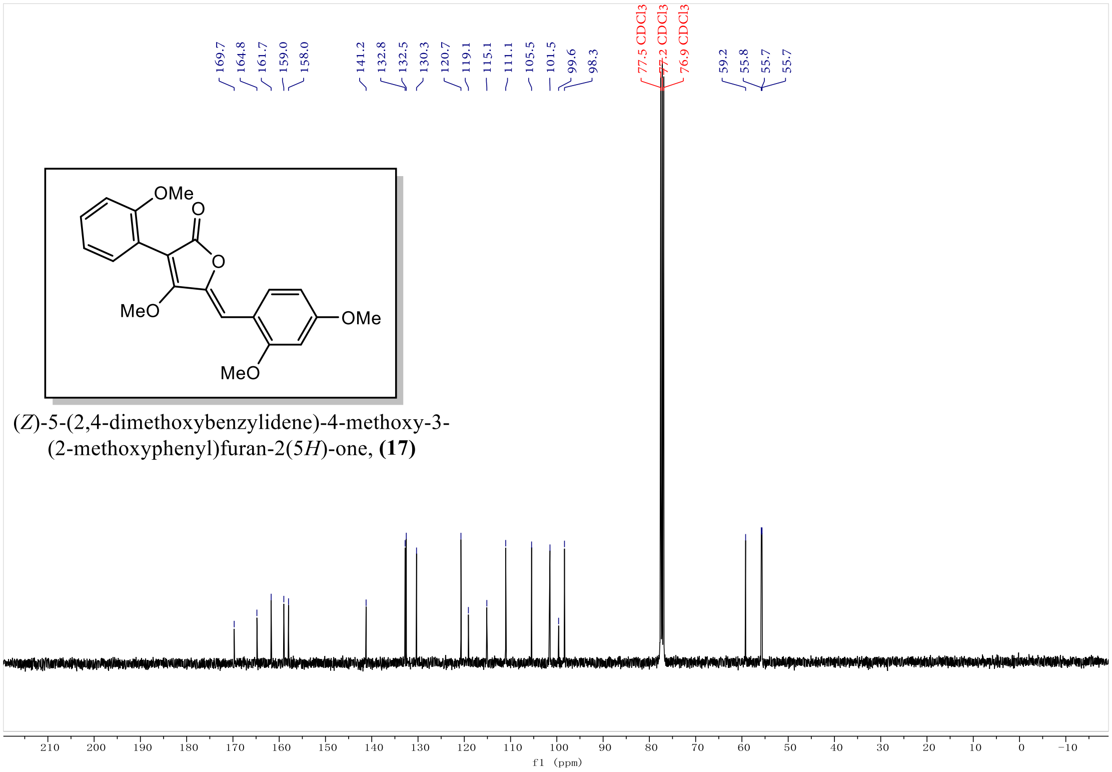

^{1}H NMR (400 MHz, Chloroform-*d*)

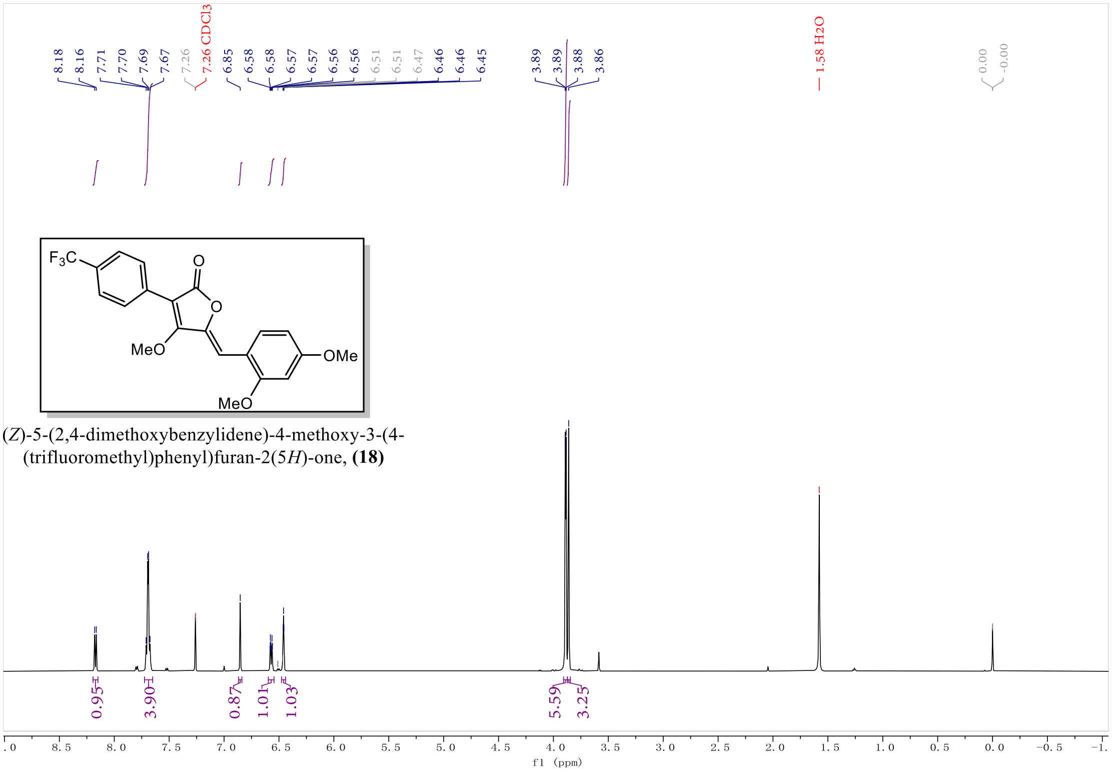

(*Z*)-5-(2,4-dimethoxybenzylidene)-4-methoxy-3-(4-(trifluoromethyl)phenyl)furan-2(5*H*)-one, **(18)**

^{13}C NMR (101 MHz, Chloroform-*d*)

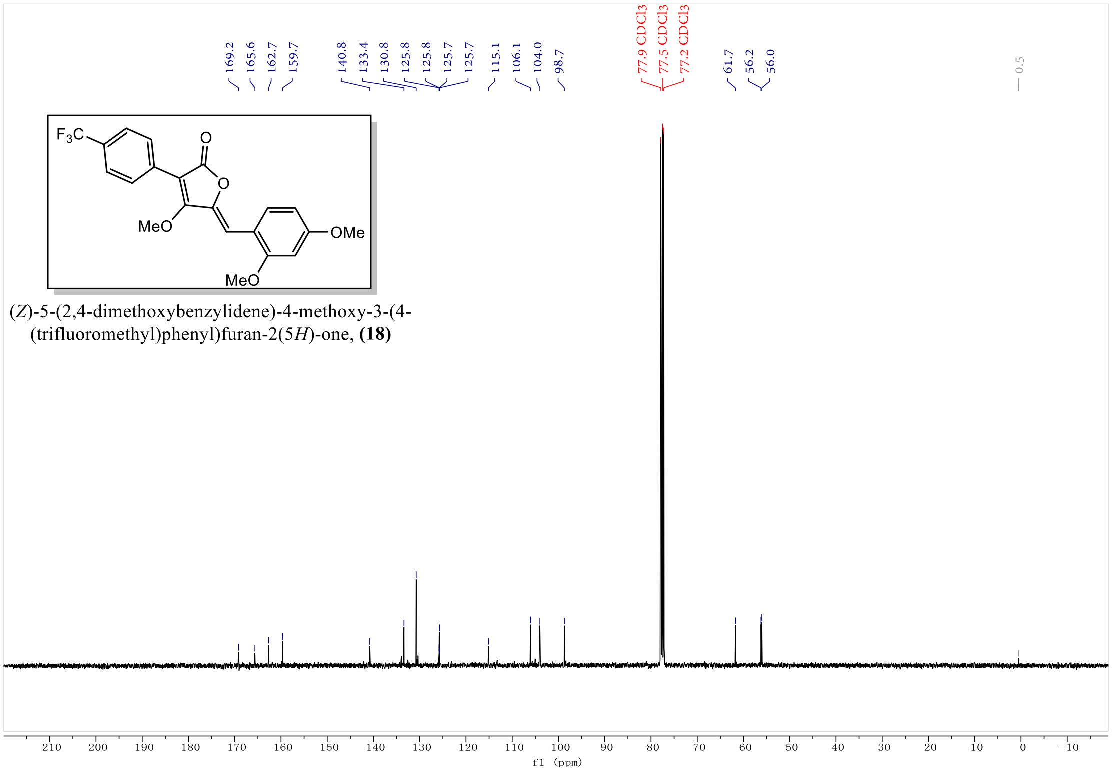

(*Z*)-5-(2,4-dimethoxybenzylidene)-4-methoxy-3-(4-(trifluoromethyl)phenyl)furan-2(5*H*)-one, **(18)**

^{19}F NMR (376 MHz, Chloroform-*d*)

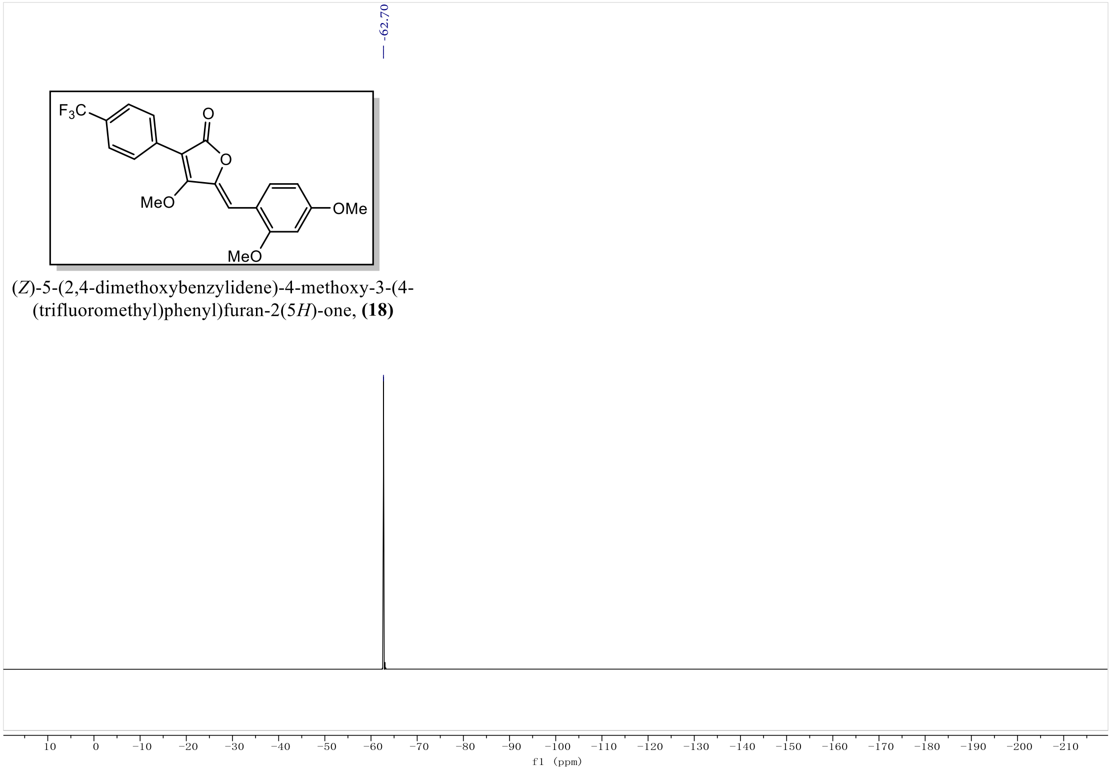

(*Z*)-5-(2,4-dimethoxybenzylidene)-4-methoxy-3-(4-(trifluoromethyl)phenyl)furan-2(5*H*)-one, **(18)**

^{1}H NMR (400 MHz, Chloroform-*d*)

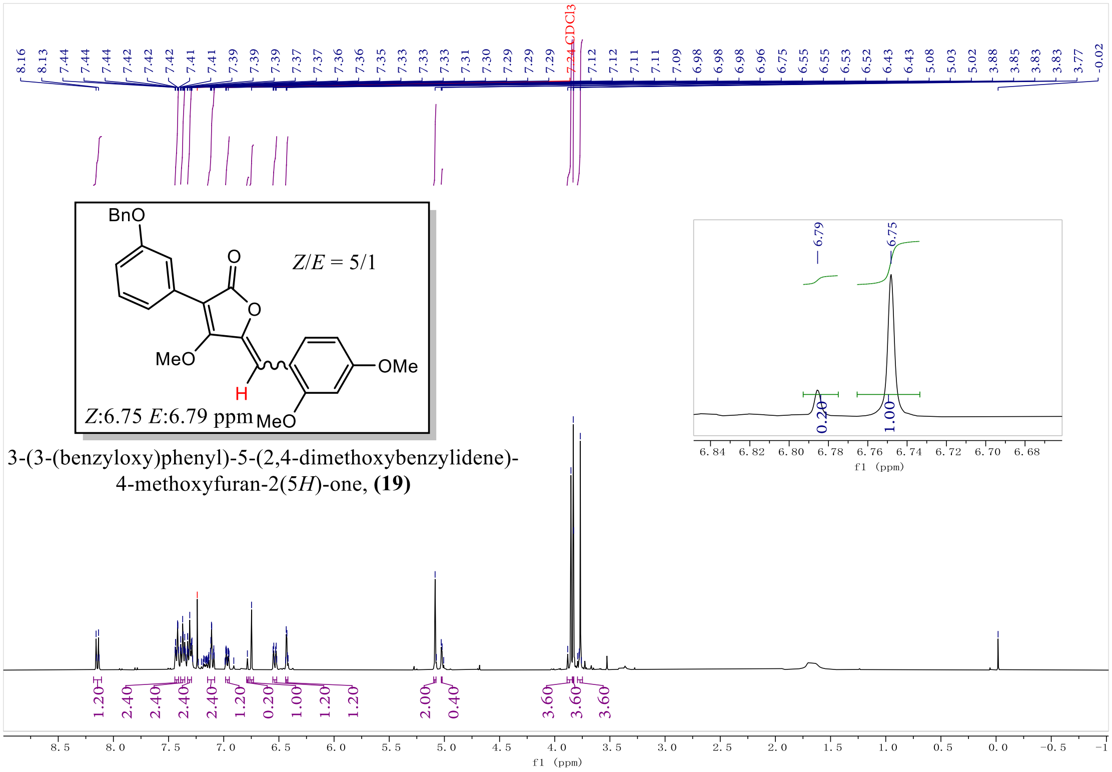

^{13}C NMR (101 MHz, Chloroform-*d*)

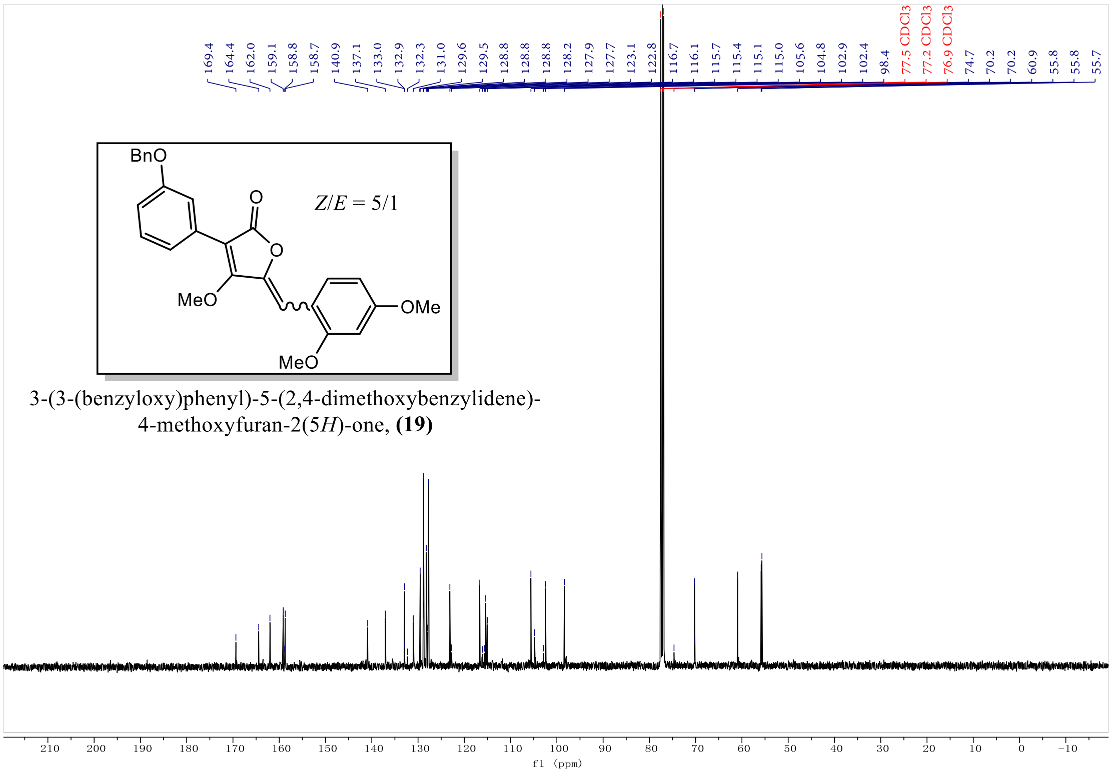

^{1}H NMR (400 MHz, Chloroform-*d*)

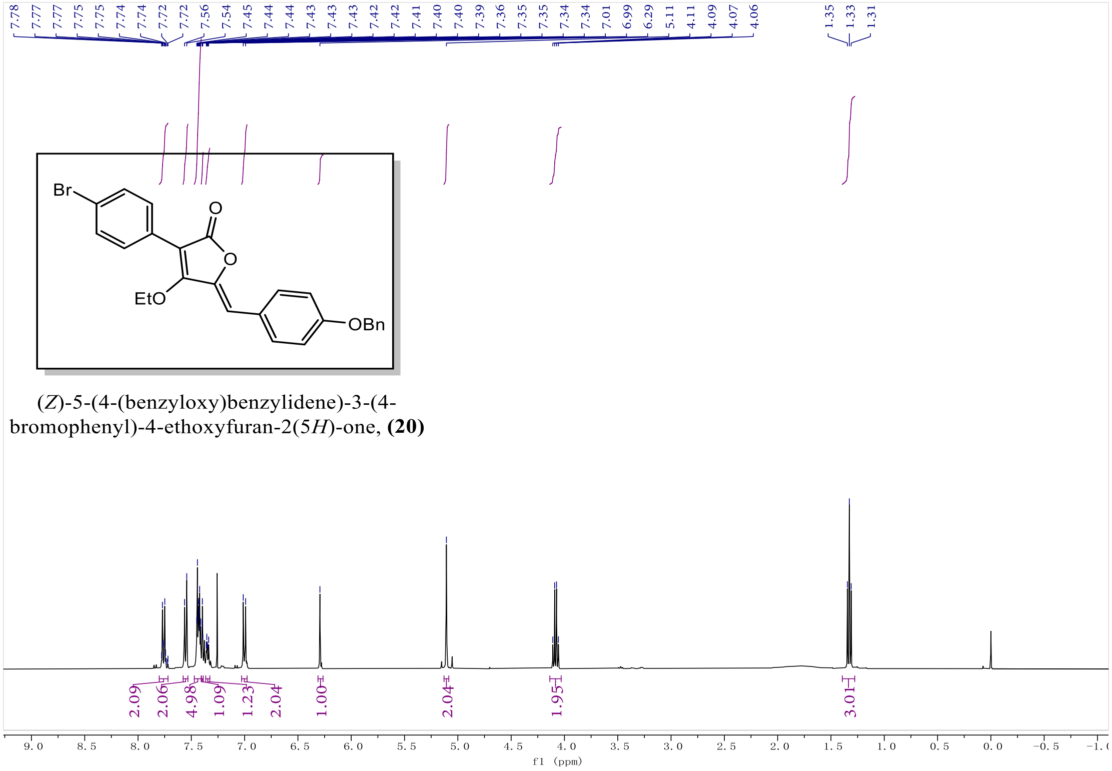

(*Z*)-5-(4-(benzyloxy)benzylidene)-3-(4-bromophenyl)-4-ethoxyfuran-2(5*H*)-one, **(20)**

^{13}C NMR (101 MHz, Chloroform-*d*)

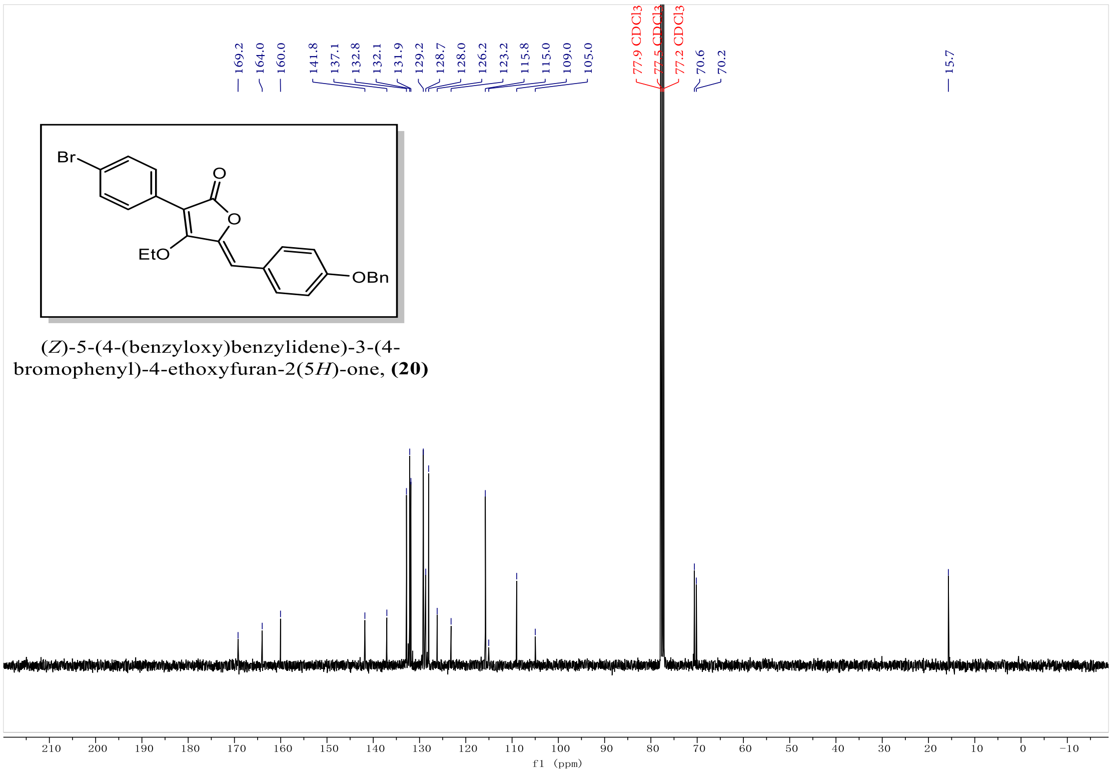

(*Z*)-5-(4-(benzyloxy)benzylidene)-3-(4-bromophenyl)-4-ethoxyfuran-2(5*H*)-one, **(20)**

^{1}H NMR (400 MHz, Chloroform-*d*)

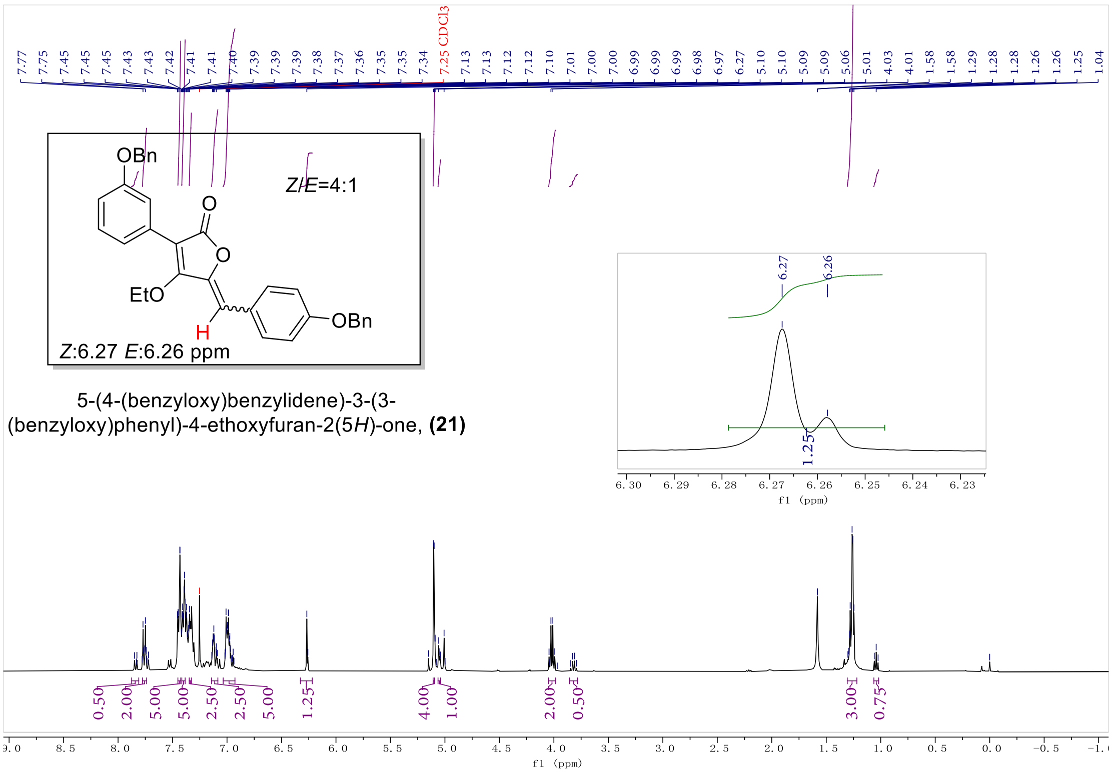

^{13}C NMR (101 MHz, Chloroform-*d*)

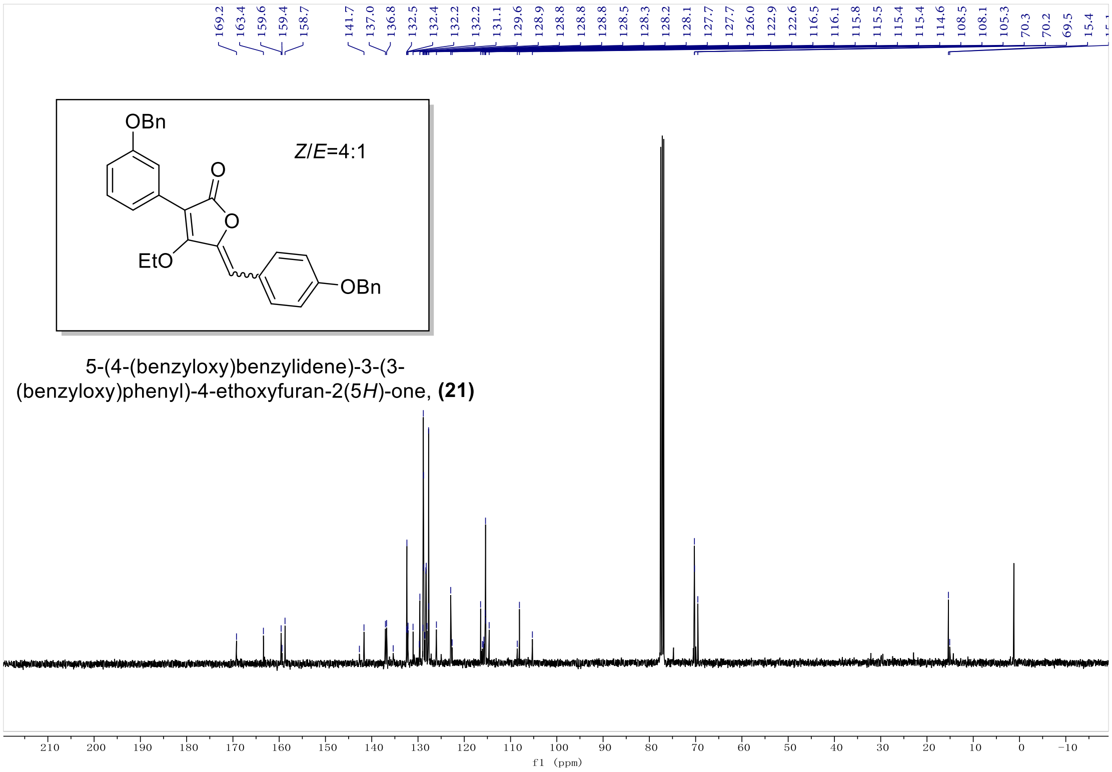

^{1}H NMR (600 MHz, Chloroform-d)

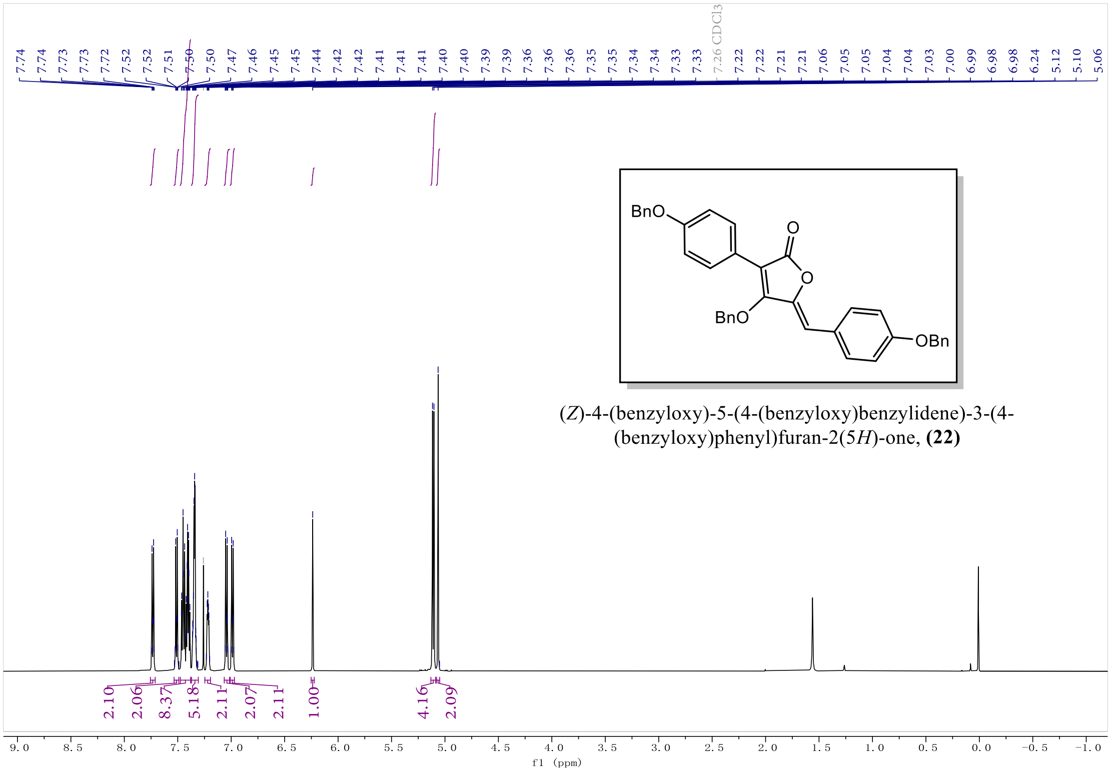

(*Z*)-4-(benzyloxy)-5-(4-(benzyloxy)benzylidene)-3-(4-(benzyloxy)phenyl)furan-2(5*H*)-one, **(22)**

^{13}C NMR (151 MHz, Chloroform-d)

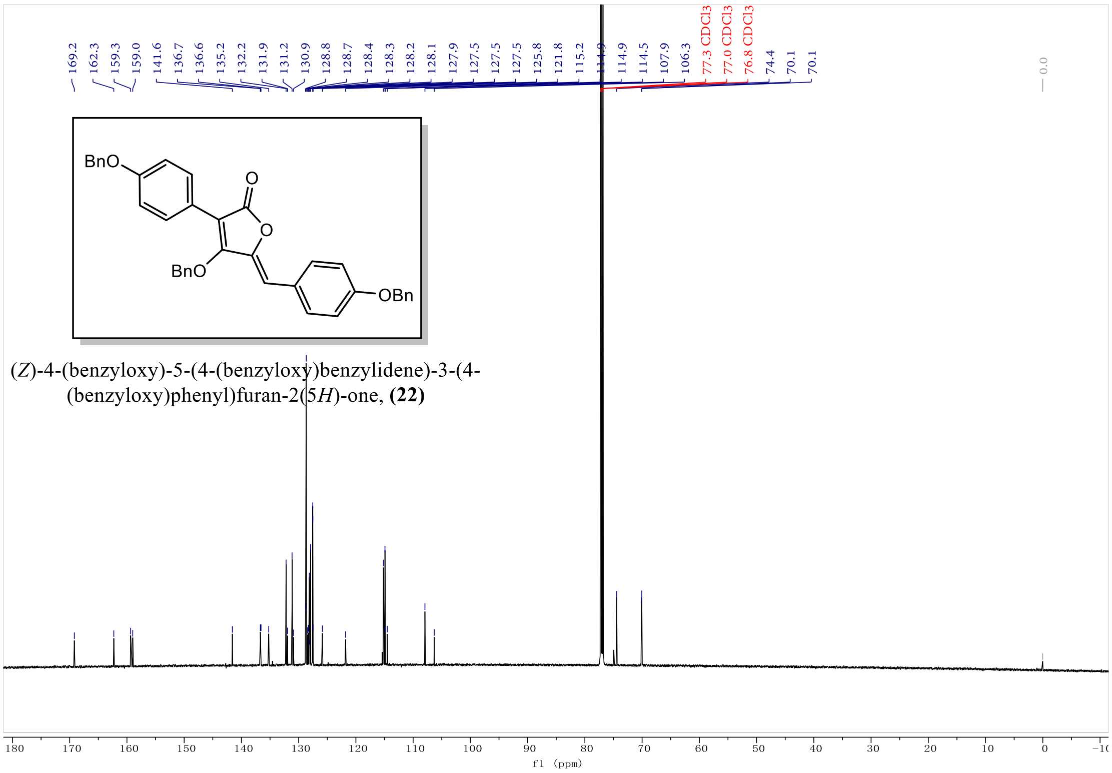

(*Z*)-4-(benzyloxy)-5-(4-(benzyloxy)benzylidene)-3-(4-(benzyloxy)phenyl)furan-2(5*H*)-one, **(22)**

^{1}H NMR (600 MHz, Chloroform-*d*)

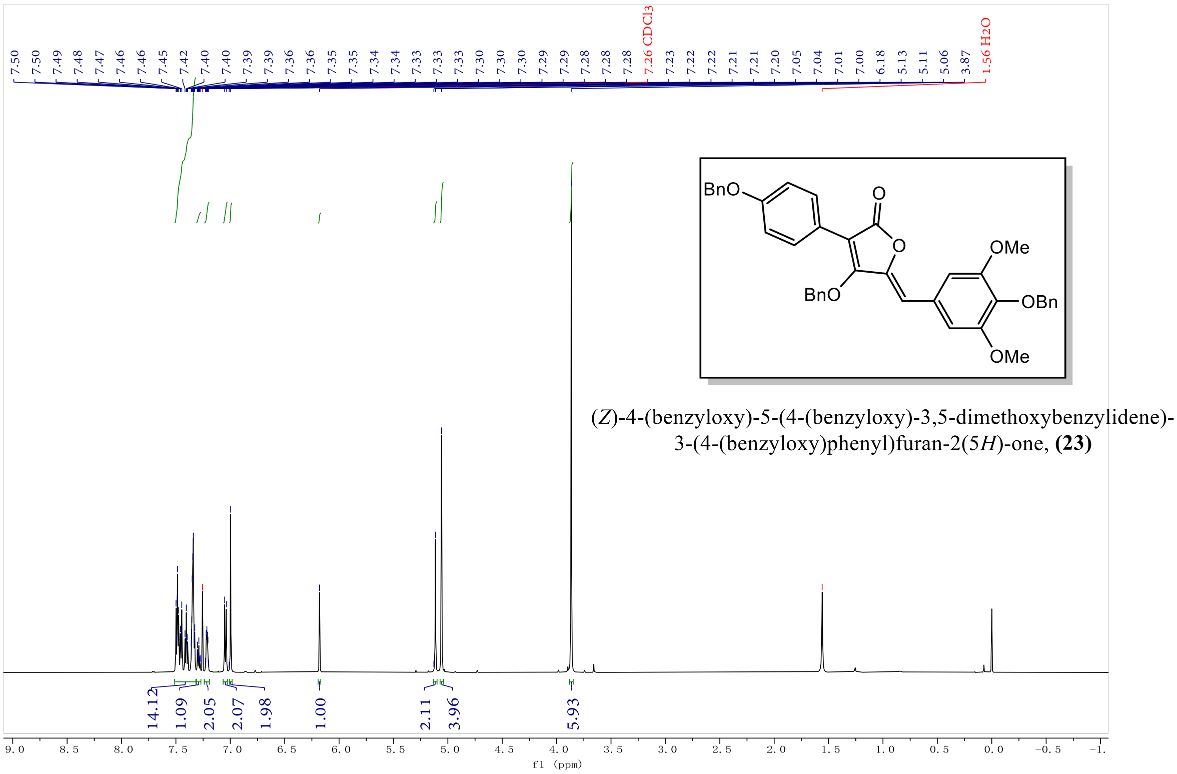

^{13}C NMR (151 MHz, Chloroform-*d*)

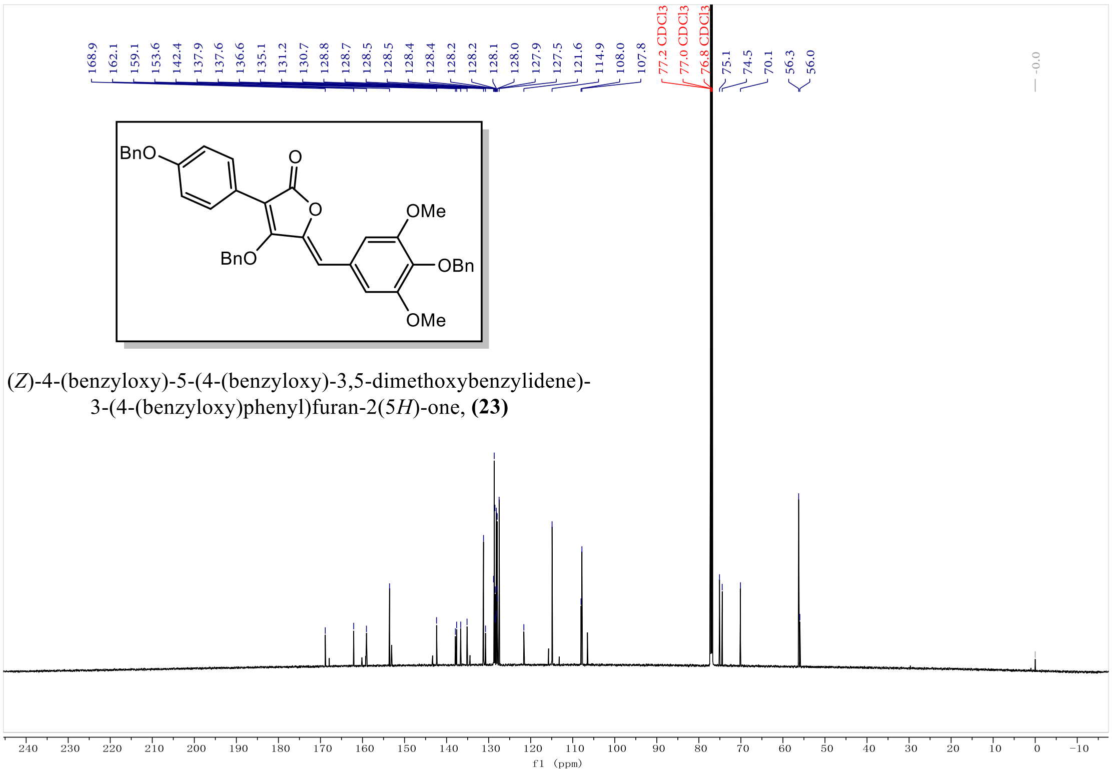

^{1}H NMR (600 MHz, Chloroform-*d*)

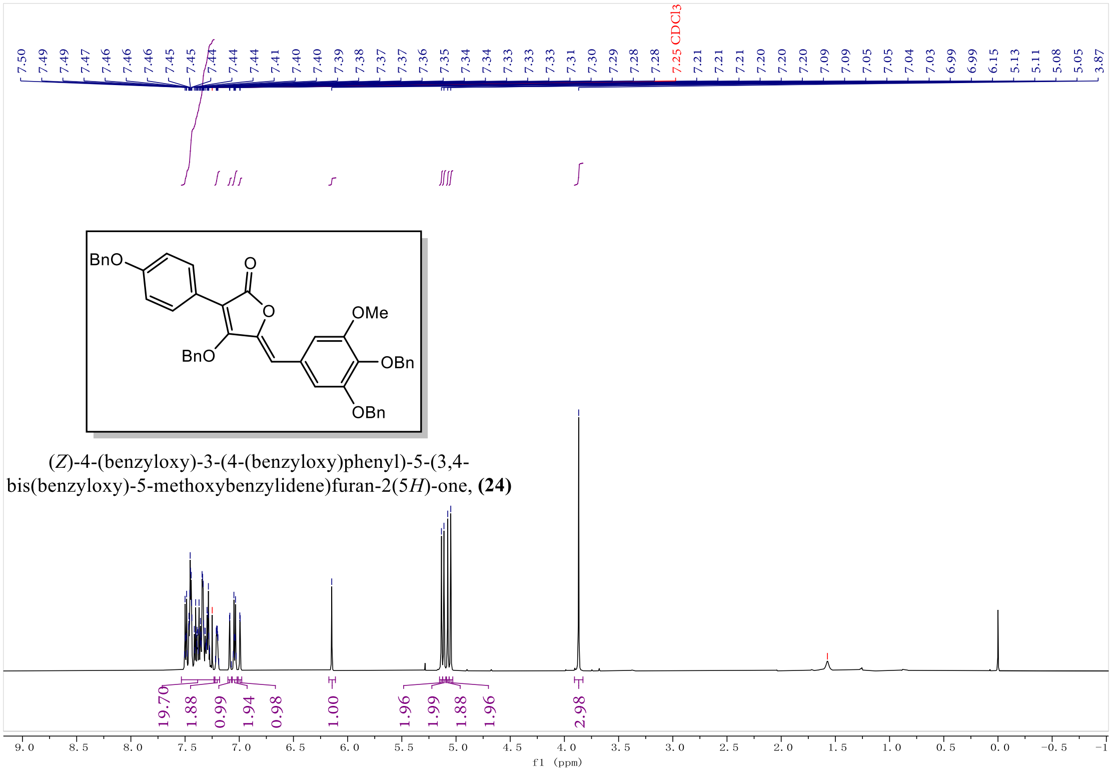

^{13}C NMR (151 MHz, Chloroform-*d*)

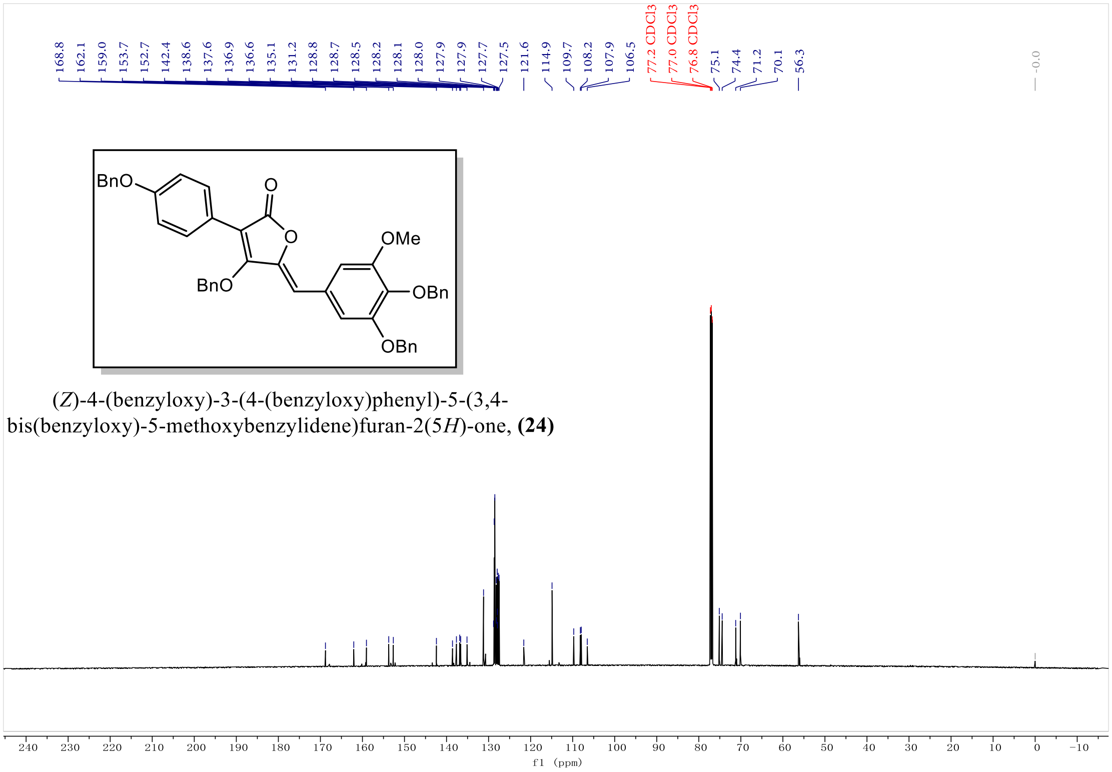

^{1}H NMR (600 MHz, Chloroform-*d*)

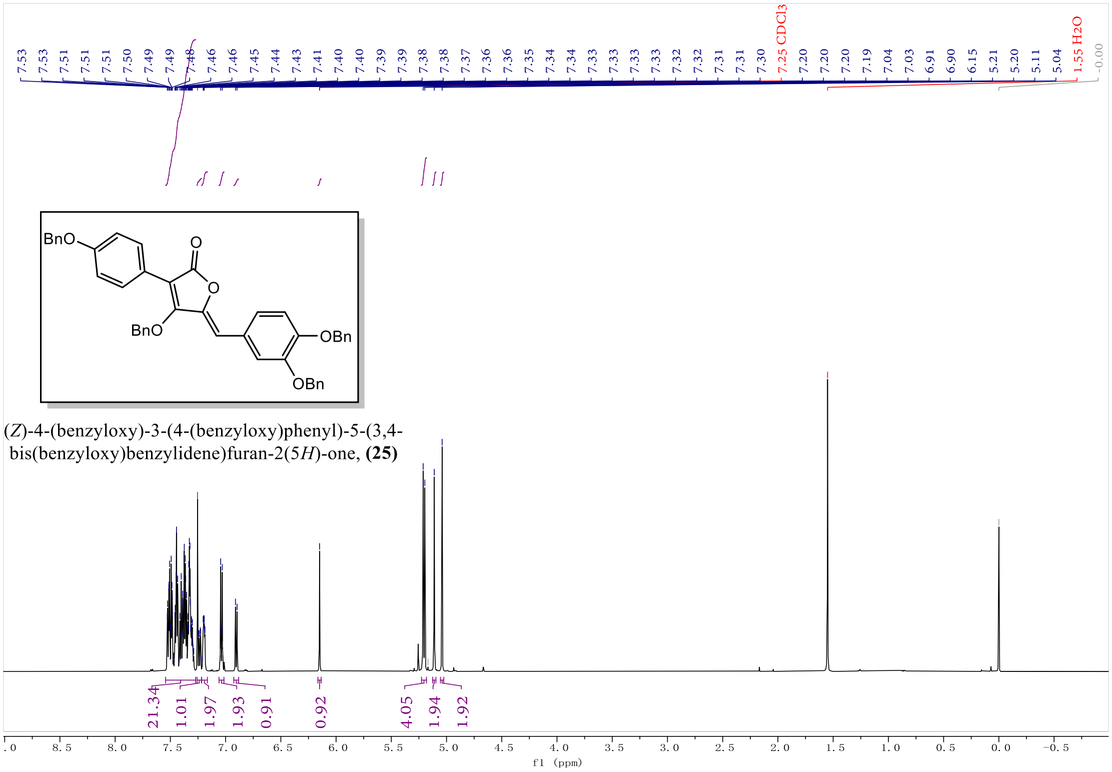

(*Z*)-4-(benzyloxy)-3-(4-(benzyloxy)phenyl)-5-(3,4-bis(benzyloxy)benzylidene)furan-2(5*H*)-one, **(25)**

^{13}C NMR (151 MHz, Chloroform-*d*)

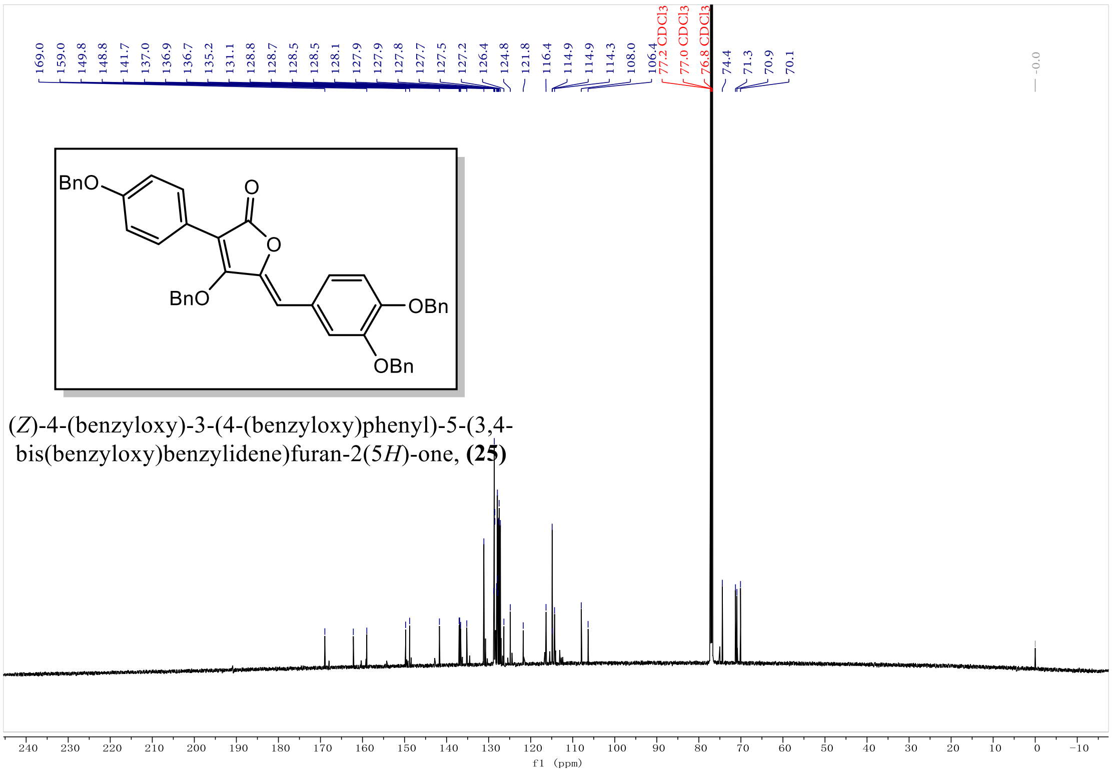

(*Z*)-4-(benzyloxy)-3-(4-(benzyloxy)phenyl)-5-(3,4-bis(benzyloxy)benzylidene)furan-2(5*H*)-one, **(25)**

^{1}H NMR (600 MHz, Chloroform-*d*)

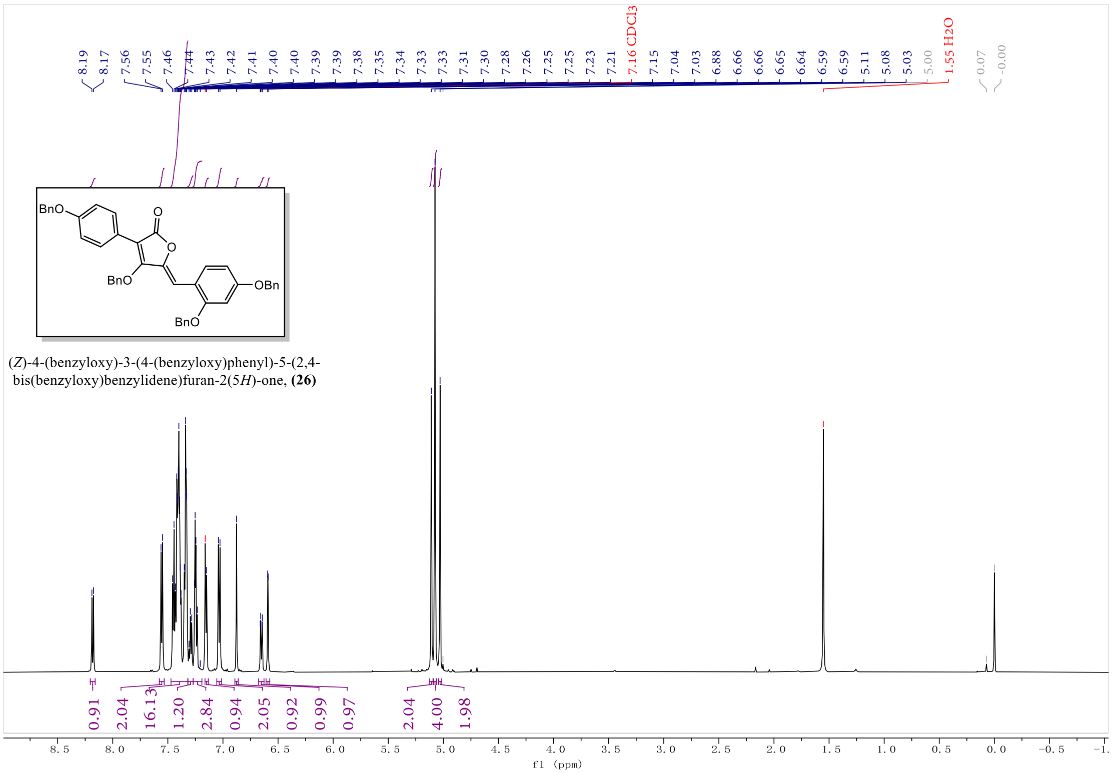

^{13}C NMR (151 MHz, Chloroform-*d*)

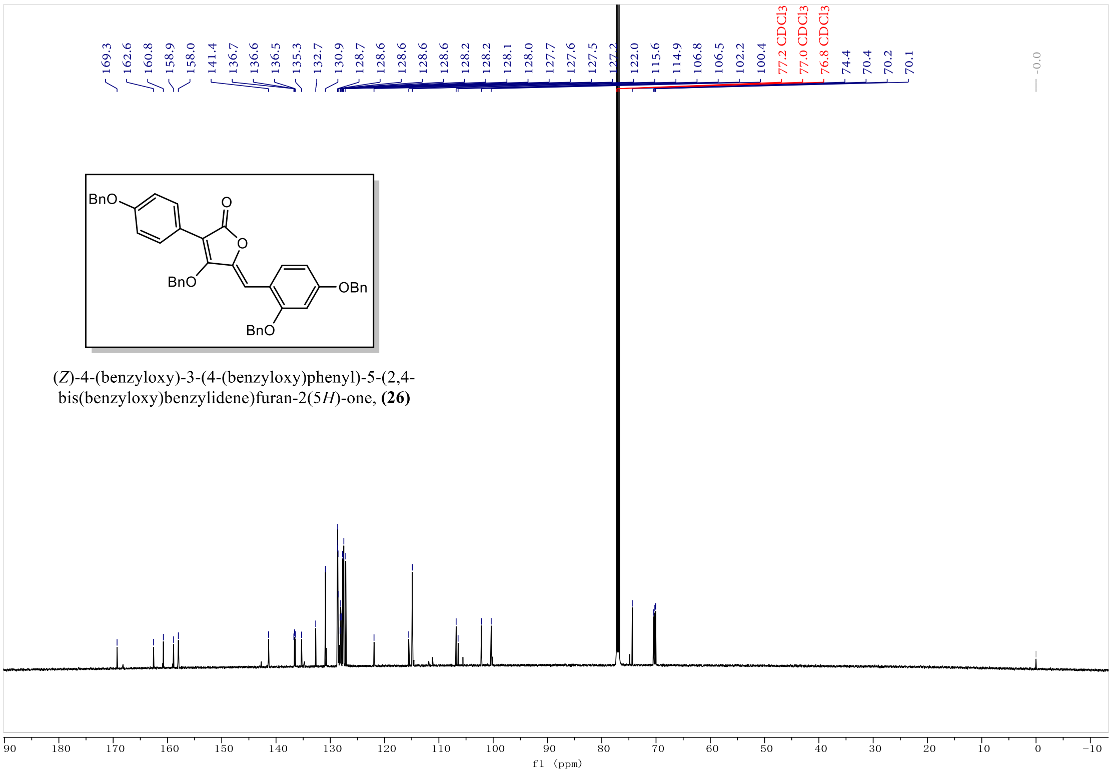

^{1}H NMR (600 MHz, Chloroform-*d*)

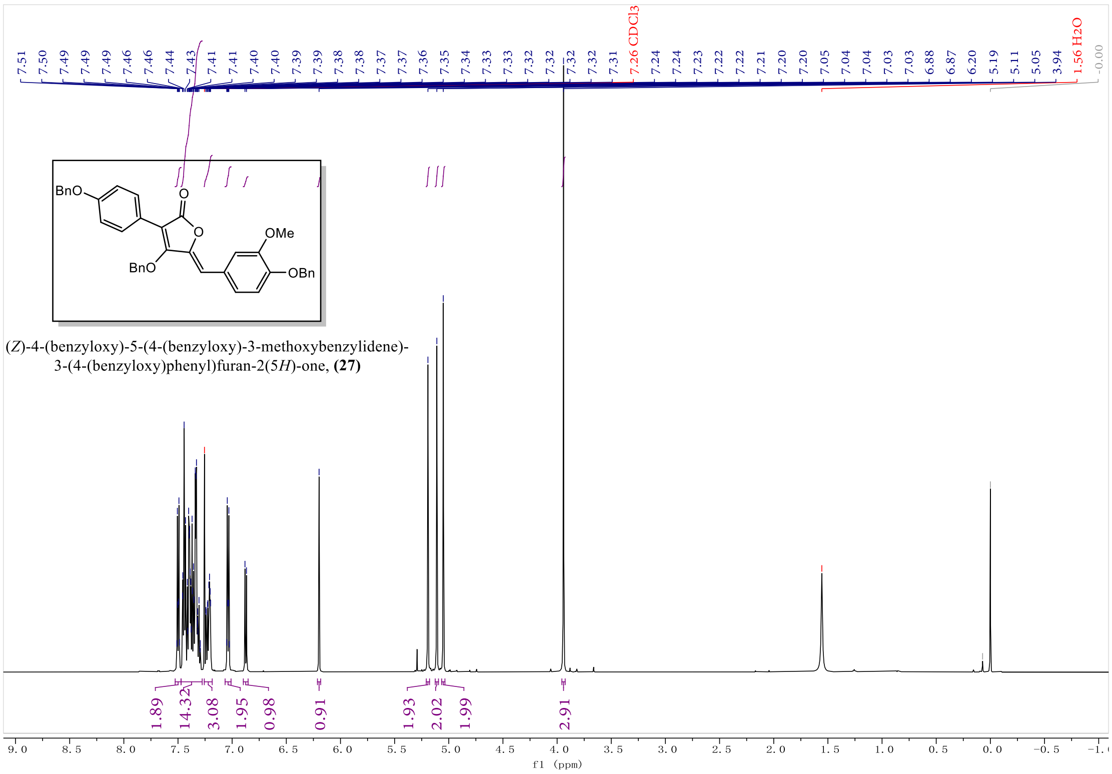

(*Z*)-4-(benzyloxy)-5-(4-(benzyloxy)-3-methoxybenzylidene)-3-(4-(benzyloxy)phenyl)furan-2(5*H*)-one, **(27)**

^{13}C NMR (151 MHz, Chloroform-*d*)

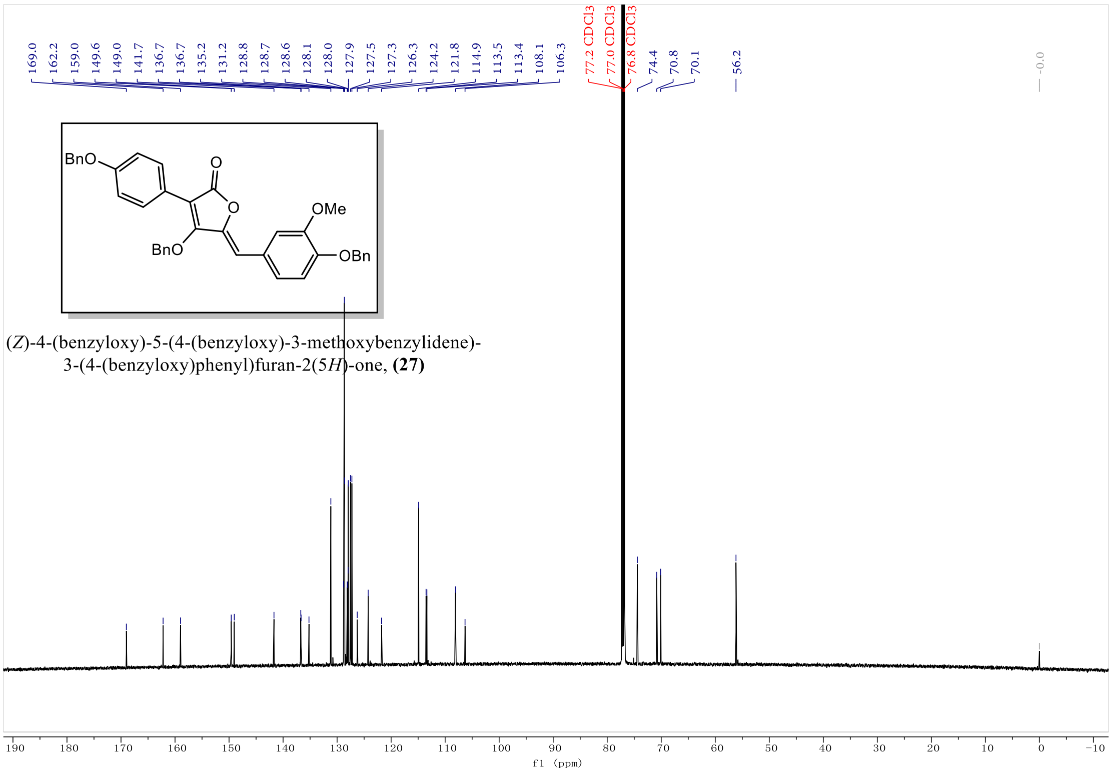

(*Z*)-4-(benzyloxy)-5-(4-(benzyloxy)-3-methoxybenzylidene)-3-(4-(benzyloxy)phenyl)furan-2(5*H*)-one, **(27)**

^{1}H NMR (400 MHz, Chloroform-*d*)

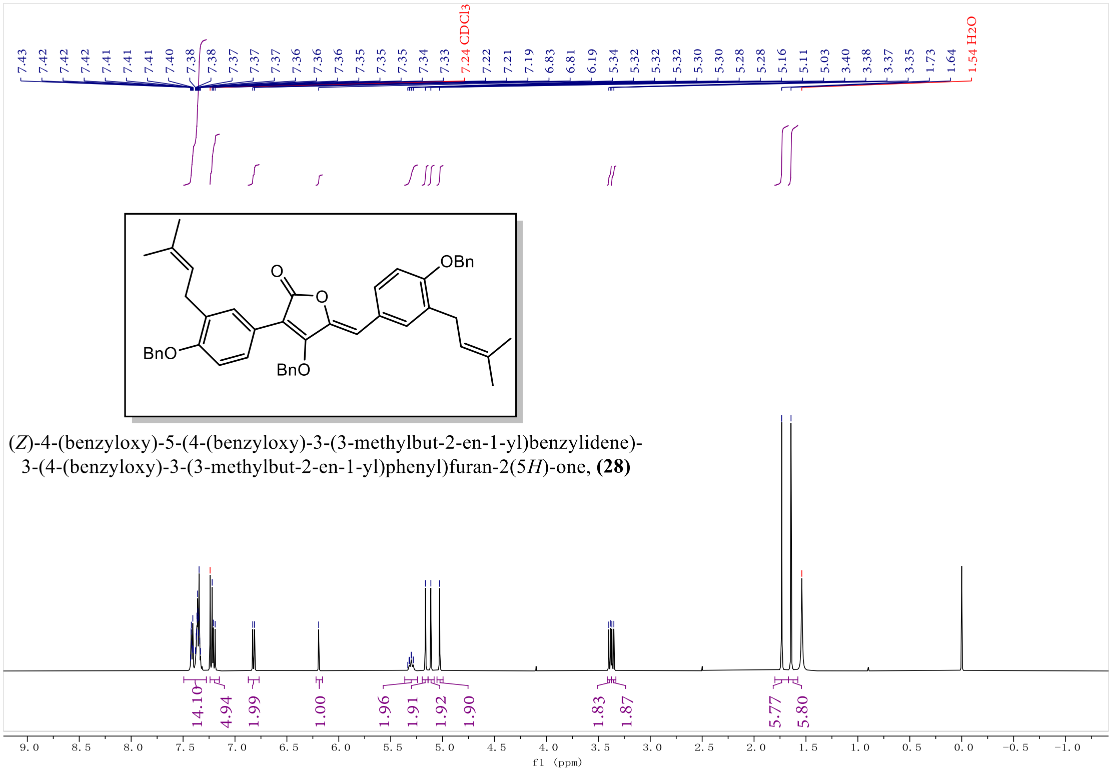

(*Z*)-4-(benzyloxy)-5-(4-(benzyloxy)-3-(3-methylbut-2-en-1-yl)benzylidene)-3-(4-(benzyloxy)-3-(3-methylbut-2-en-1-yl)phenyl)furan-2(5*H*)-one, **(28)**

^{13}C NMR (101 MHz, Chloroform-*d*)

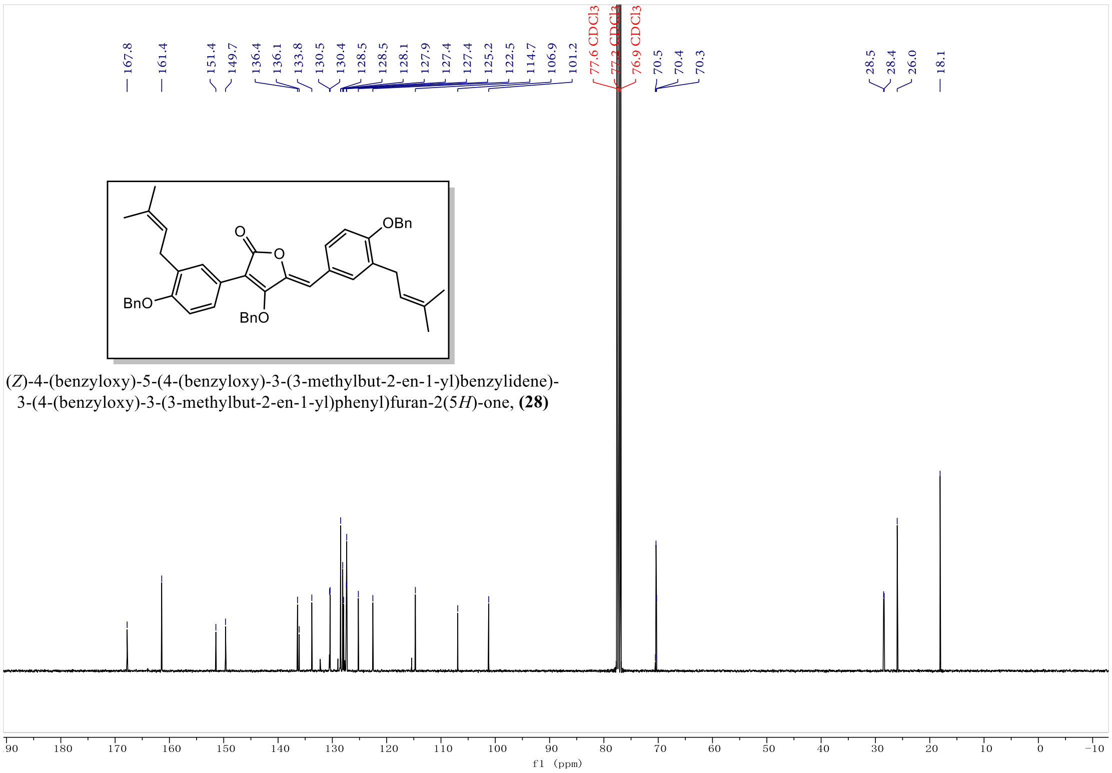

(*Z*)-4-(benzyloxy)-5-(4-(benzyloxy)-3-(3-methylbut-2-en-1-yl)benzylidene)-3-(4-(benzyloxy)-3-(3-methylbut-2-en-1-yl)phenyl)furan-2(5*H*)-one, **(28)**

^{1}H NMR (400 MHz, Chloroform-*d*)

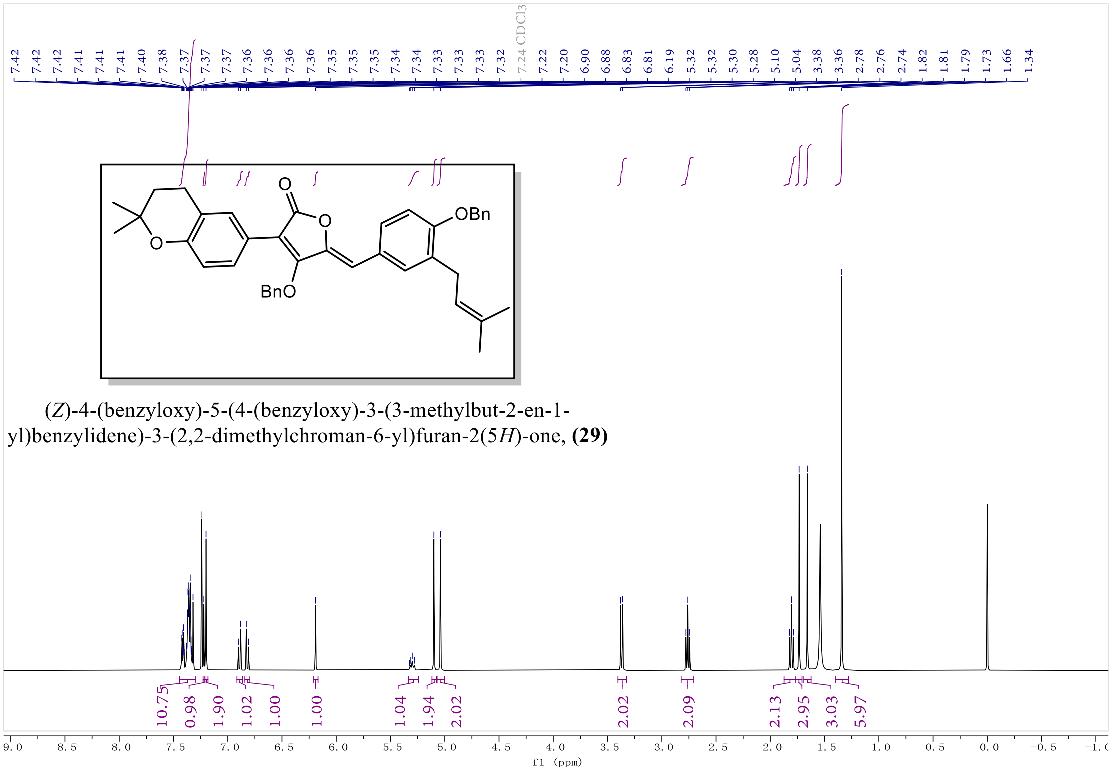

^{13}C NMR (101 MHz, Chloroform-*d*)

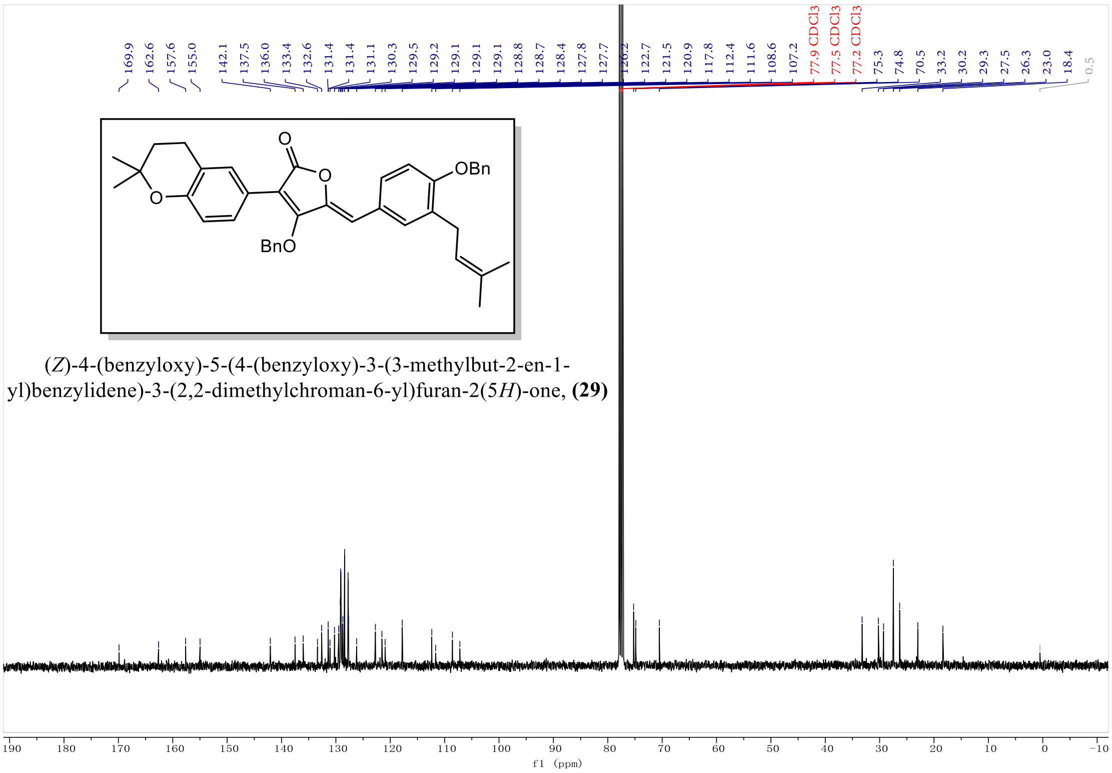

^{1}H NMR (400 MHz, Chloroform-*d*)

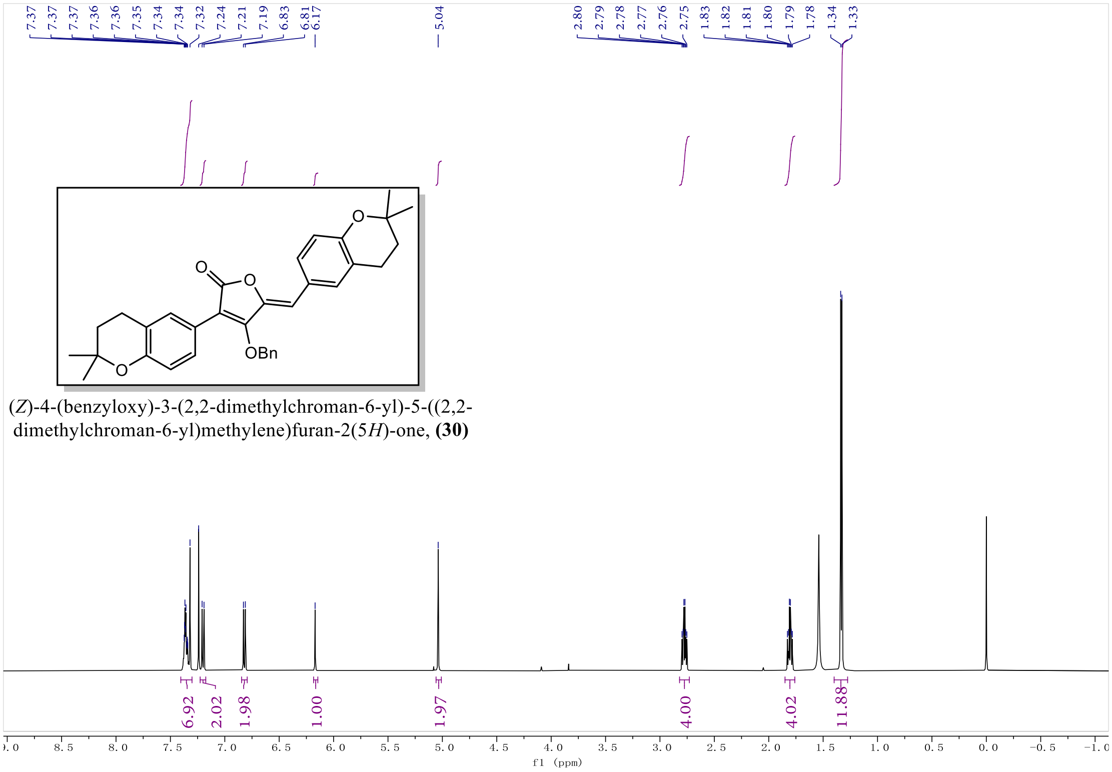

^{13}C NMR (101 MHz, Chloroform-*d*)

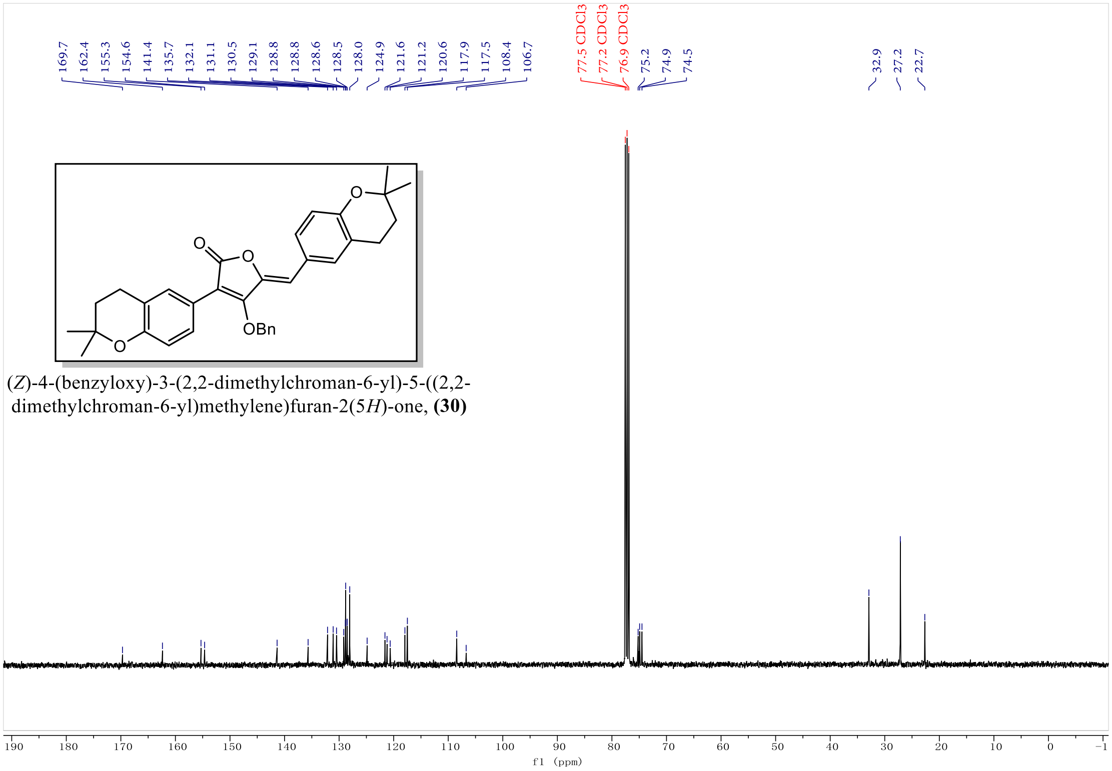

^{1}H NMR (400 MHz, Chloroform-*d*)

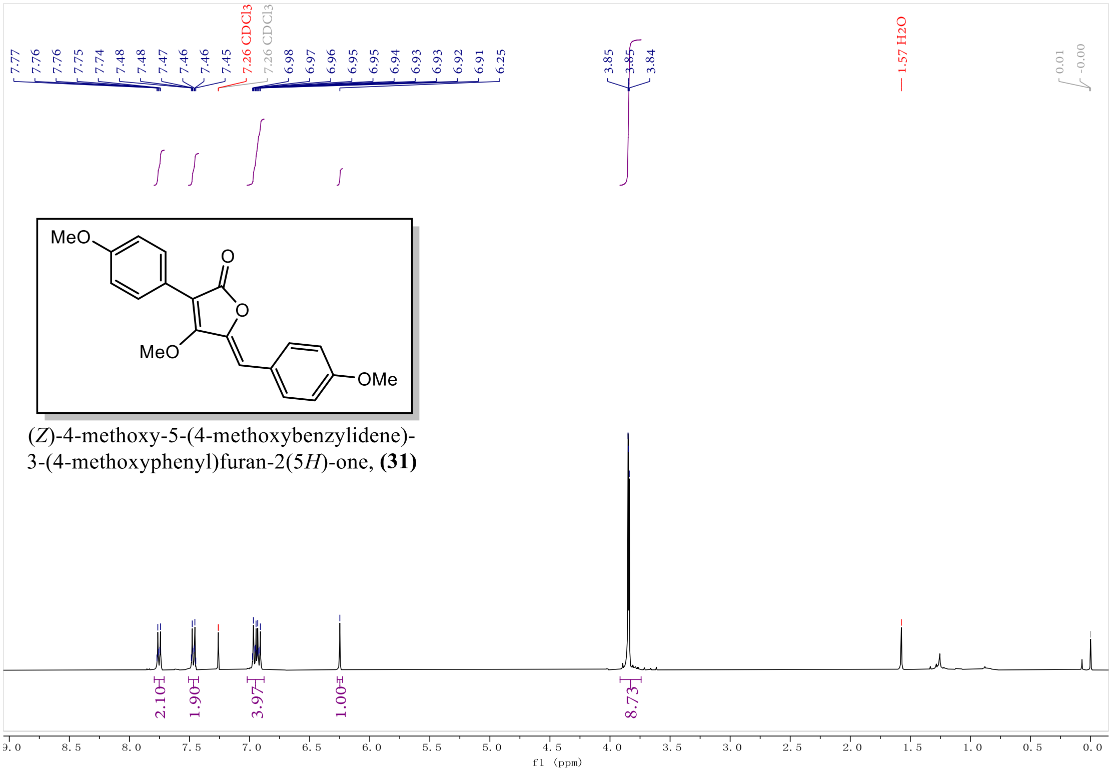

^{13}C NMR (101 MHz, Chloroform-*d*)

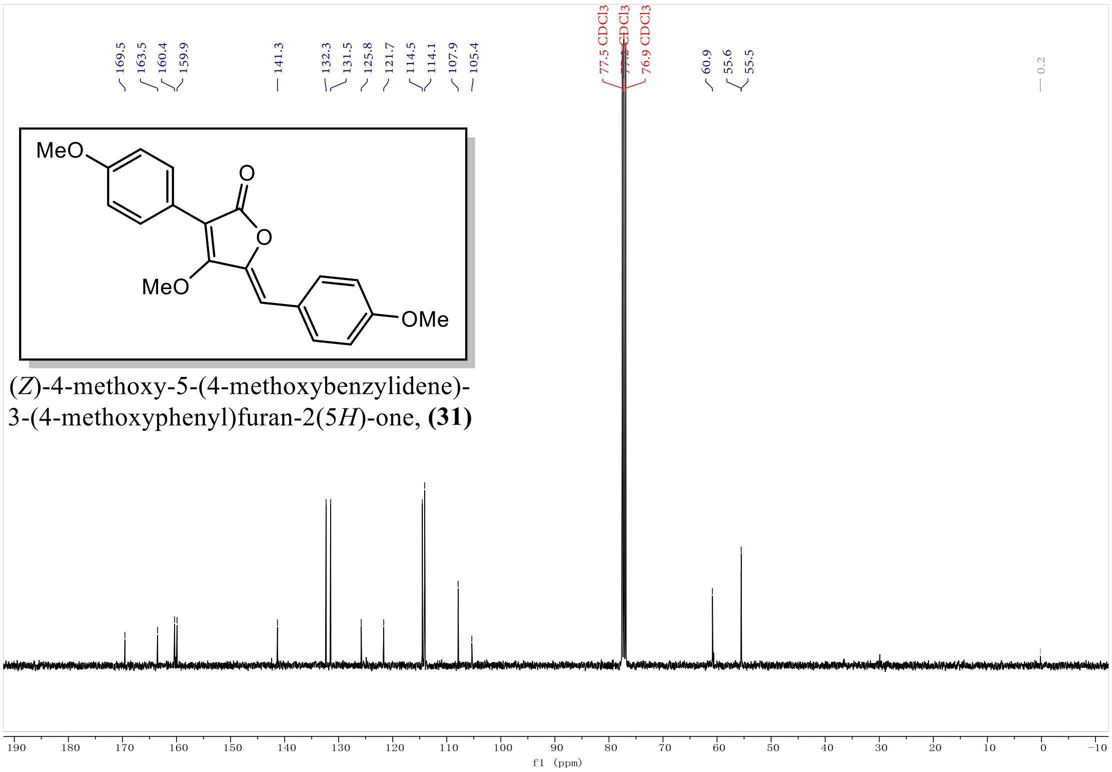

^{1}H NMR (400 MHz, Chloroform-*d*)

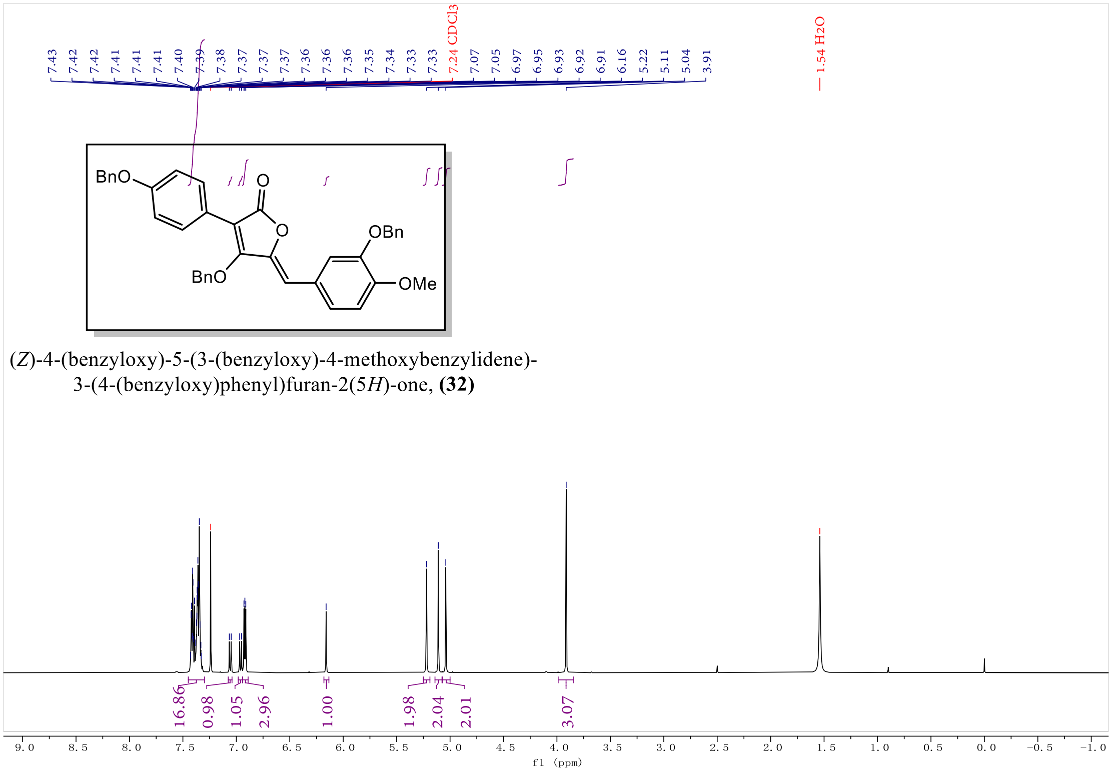

(*Z*)-4-(benzyloxy)-5-(3-(benzyloxy)-4-methoxybenzylidene)-3-(4-(benzyloxy)phenyl)furan-2(5*H*)-one, **(32)**

^{13}C NMR (101 MHz, Chloroform-*d*)

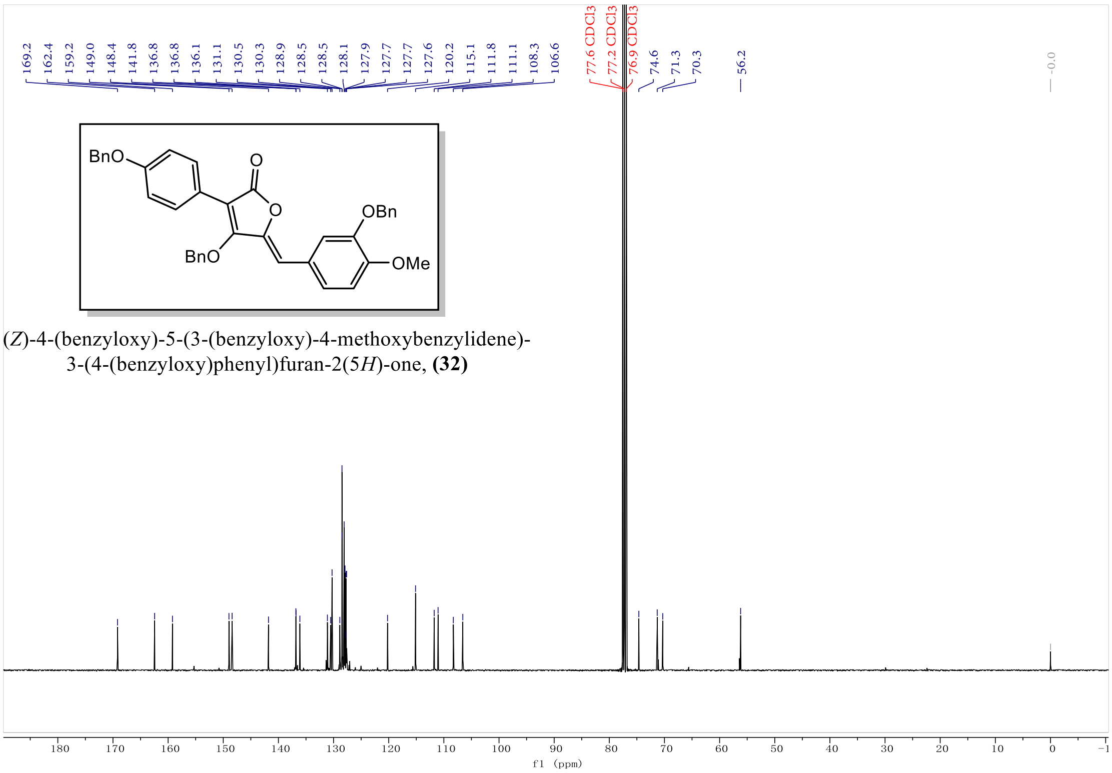

(*Z*)-4-(benzyloxy)-5-(3-(benzyloxy)-4-methoxybenzylidene)-3-(4-(benzyloxy)phenyl)furan-2(5*H*)-one, **(32)**

^{1}H NMR (400 MHz, Chloroform-*d*)

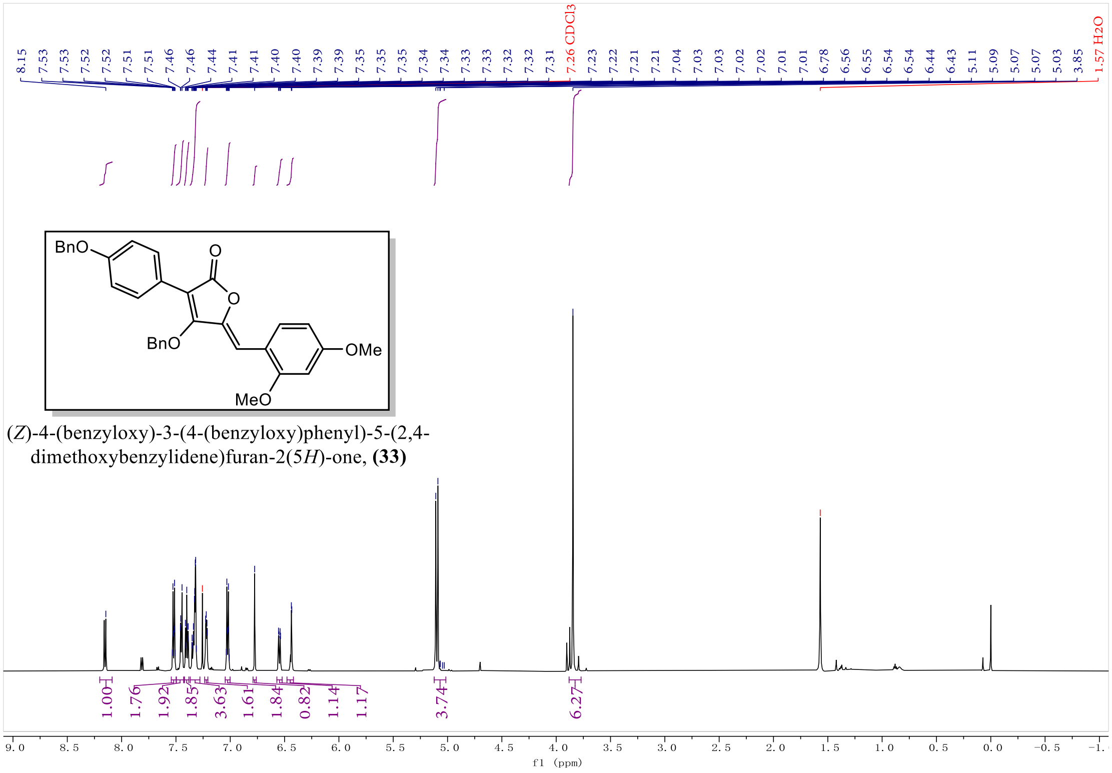

(*Z*)-4-(benzyloxy)-3-(4-(benzyloxy)phenyl)-5-(2,4-dimethoxybenzylidene)furan-2(5*H*)-one, **(33)**

^{13}C NMR (101 MHz, DMSO-d_6)

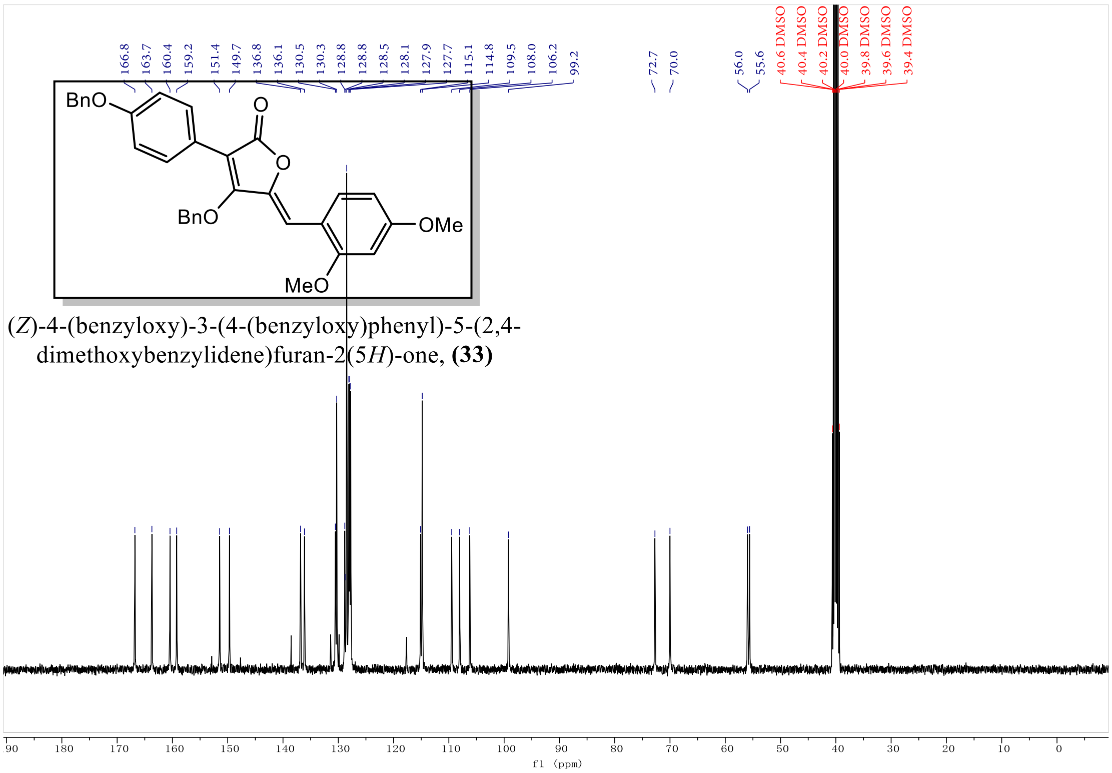

(*Z*)-4-(benzyloxy)-3-(4-(benzyloxy)phenyl)-5-(2,4-dimethoxybenzylidene)furan-2(5*H*)-one, **(33)**

^{1}H NMR (600 MHz, Chloroform-*d*)

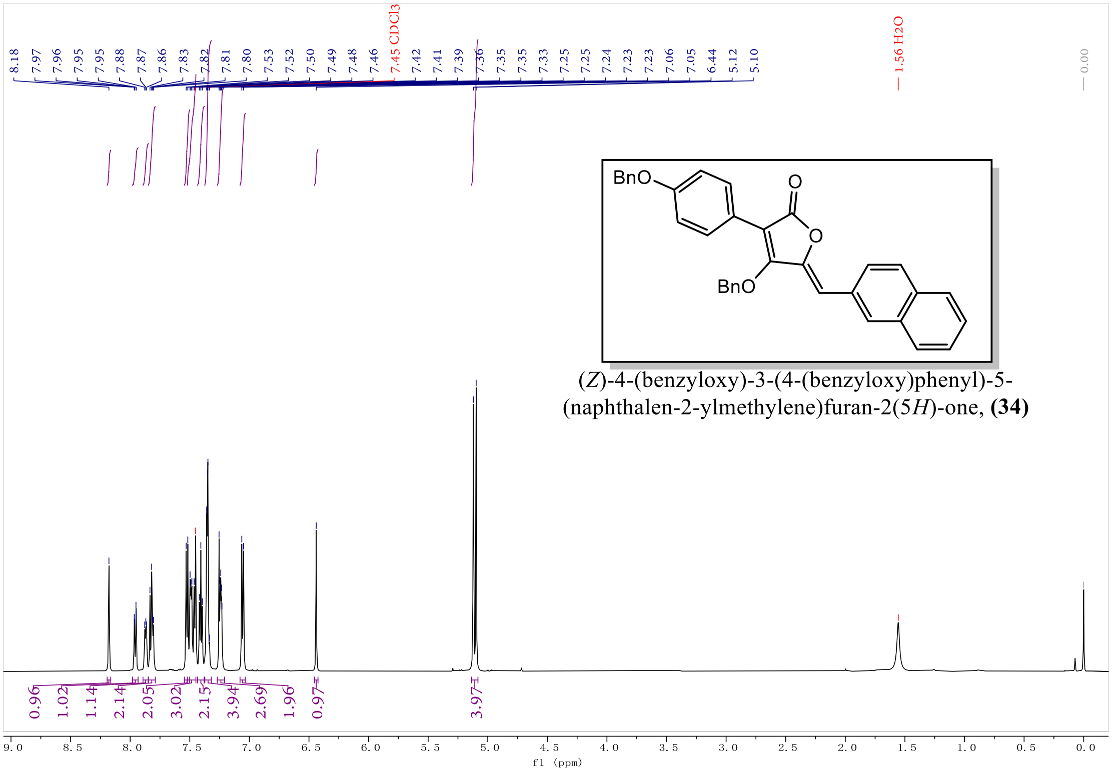

(*Z*)-4-(benzyloxy)-3-(4-(benzyloxy)phenyl)-5-(naphthalen-2-ylmethylene)furan-2(5*H*)-one, **(34)**

^{13}C NMR (151 MHz, Chloroform-*d*)

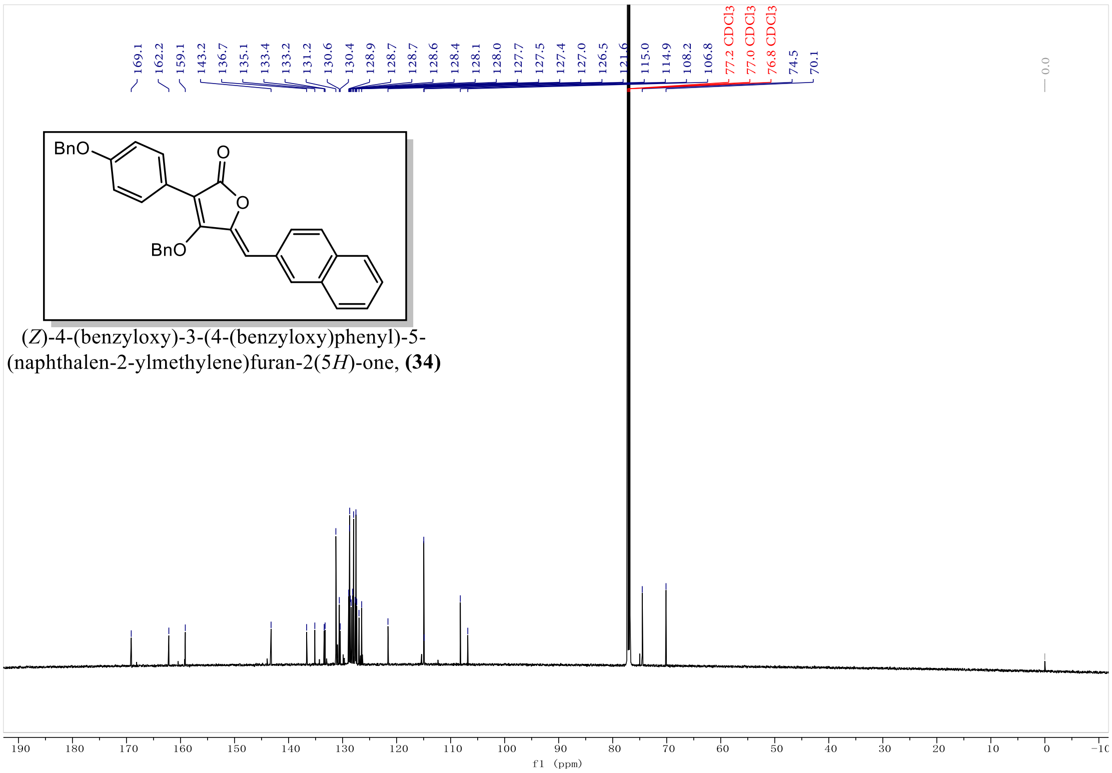

(*Z*)-4-(benzyloxy)-3-(4-(benzyloxy)phenyl)-5-(naphthalen-2-ylmethylene)furan-2(5*H*)-one, **(34)**

^{1}H NMR (400 MHz, Chloroform-*d*)

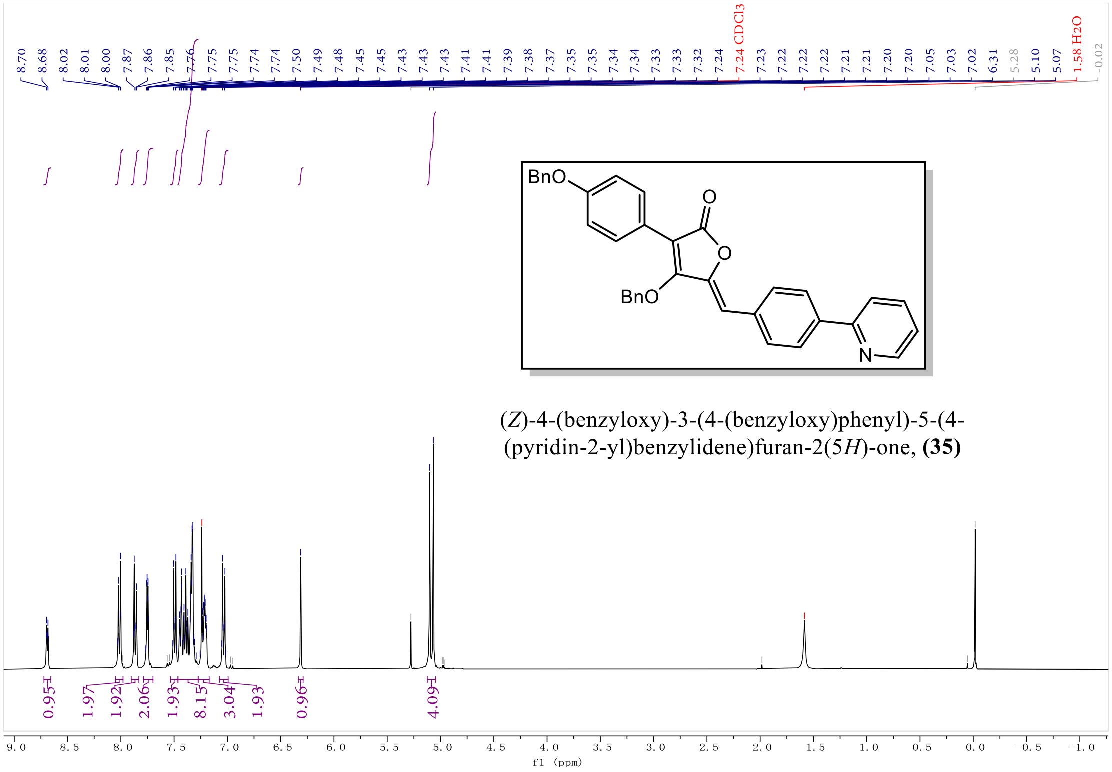

^{13}C NMR (101 MHz, Chloroform-*d*)

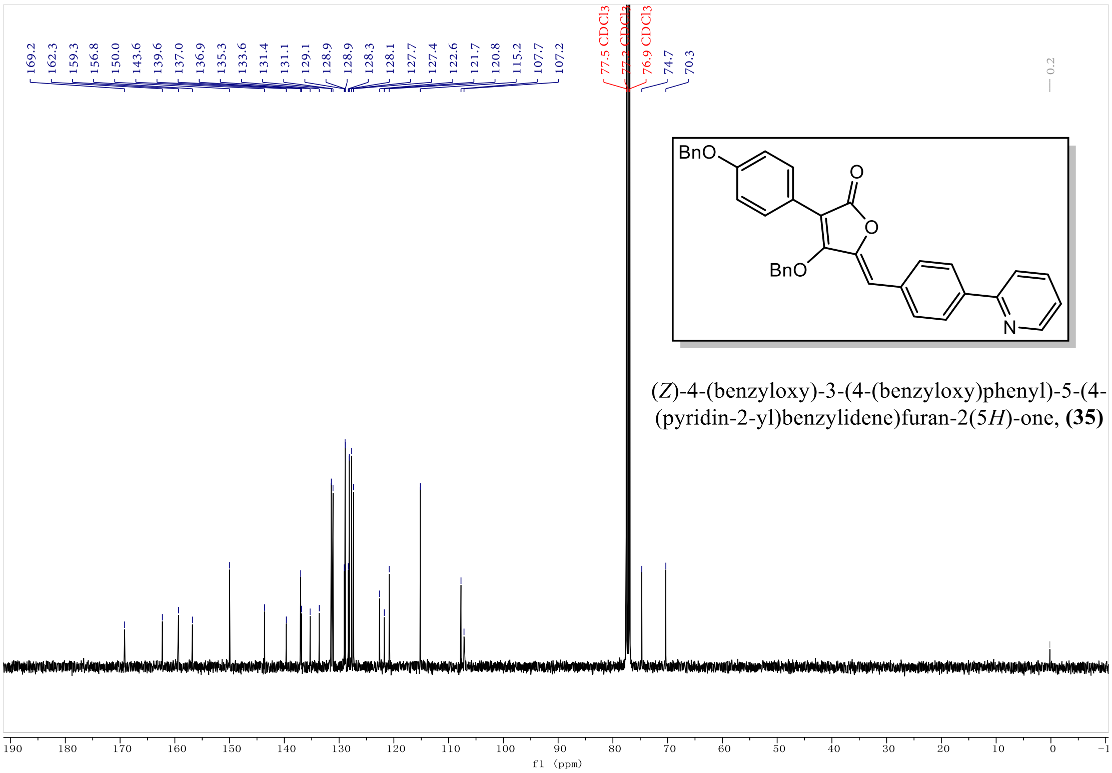

^{1}H NMR (600 MHz, Chloroform-*d*)

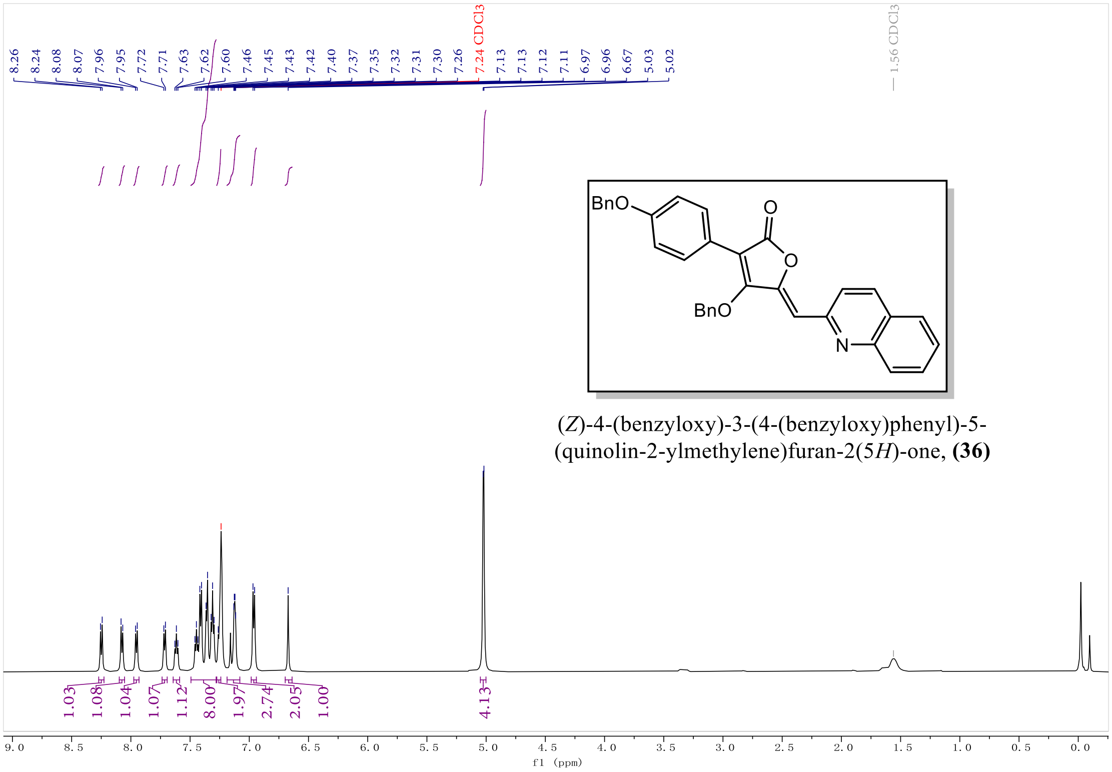

^{13}C NMR (151 MHz, Chloroform-*d*)

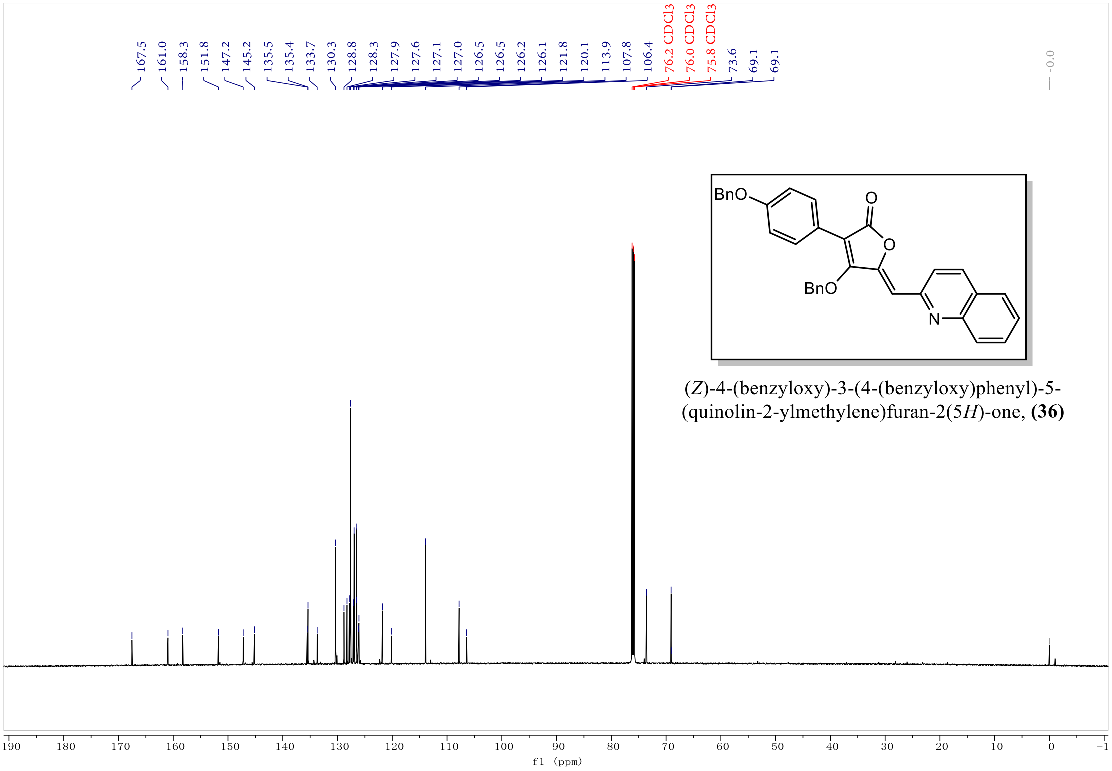

^{1}H NMR (600 MHz, Chloroform-*d*)

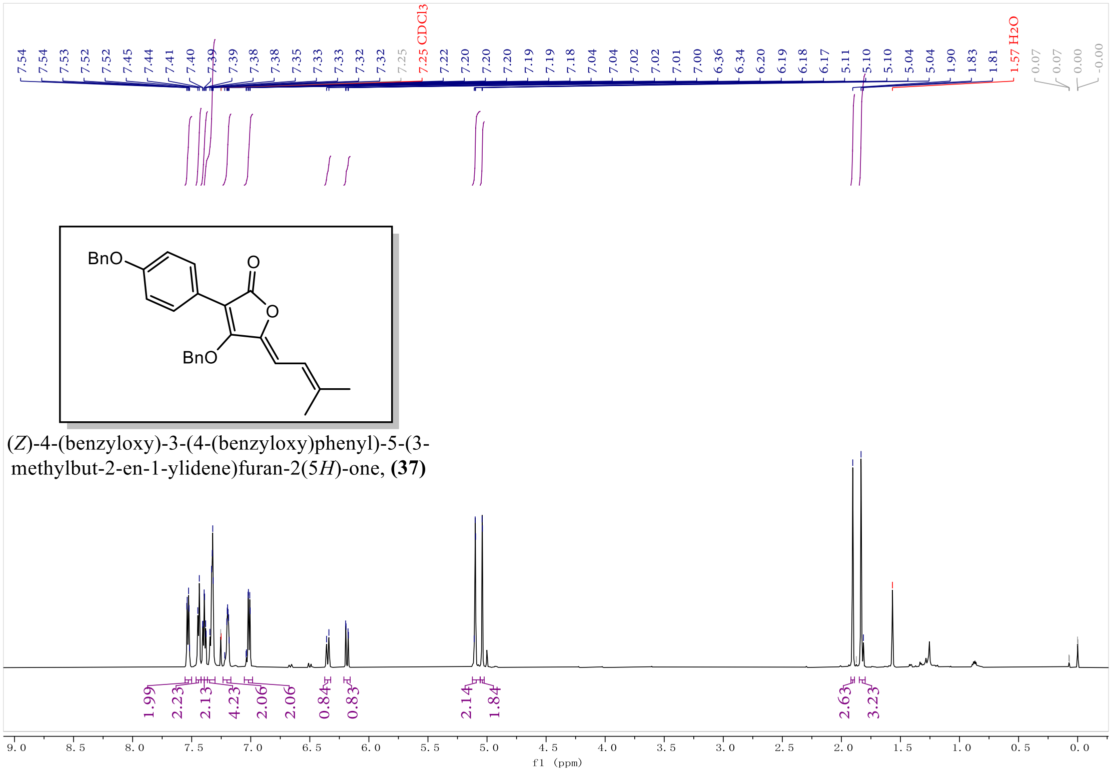

^{13}C NMR (151 MHz, Chloroform-*d*)

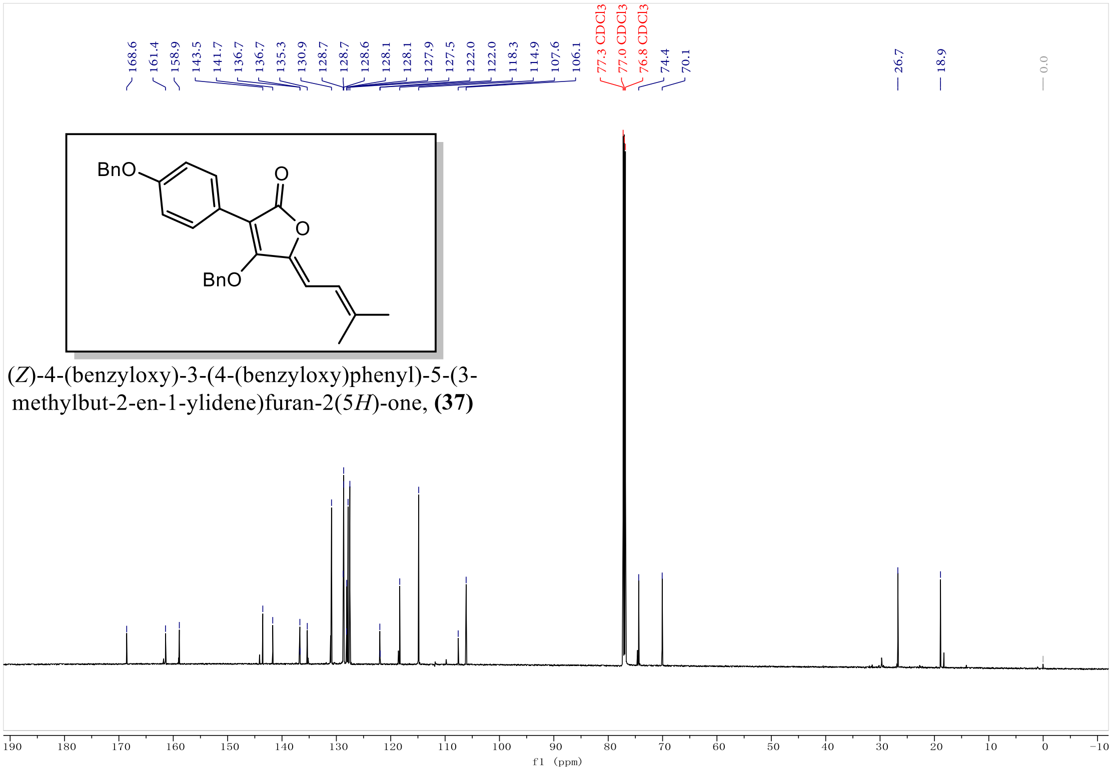

^{1}H NMR (600 MHz, Chloroform-*d*)

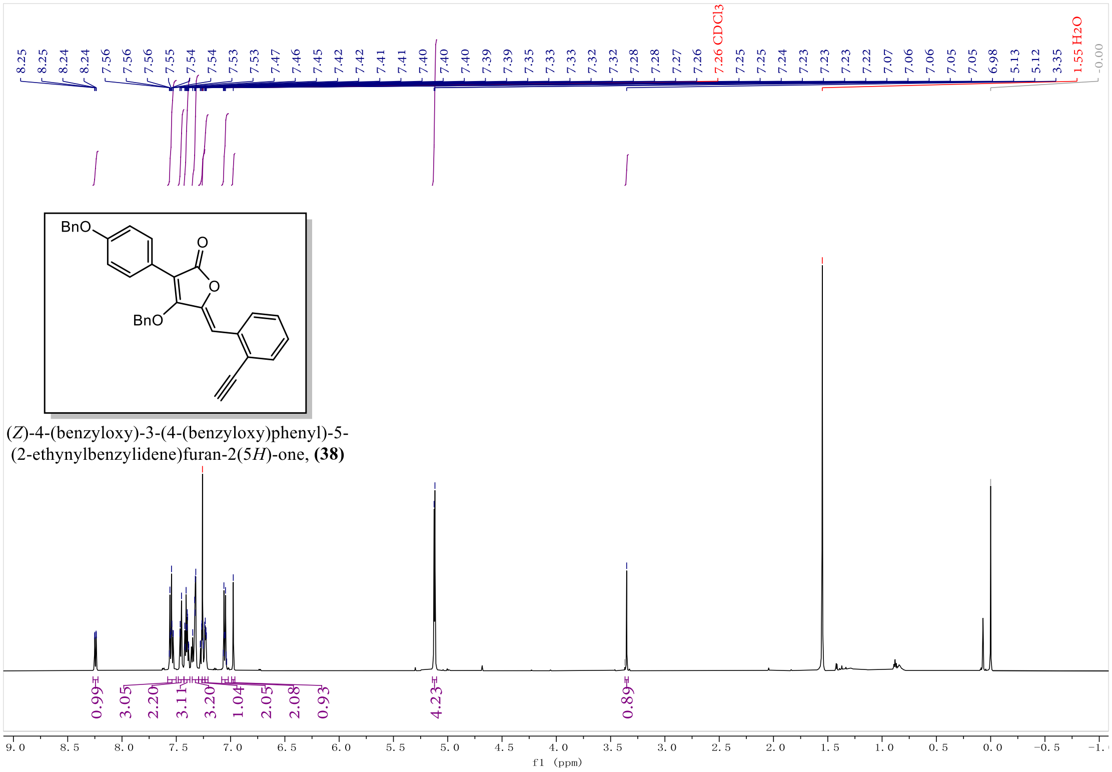

^{13}C NMR (151 MHz, Chloroform-*d*)

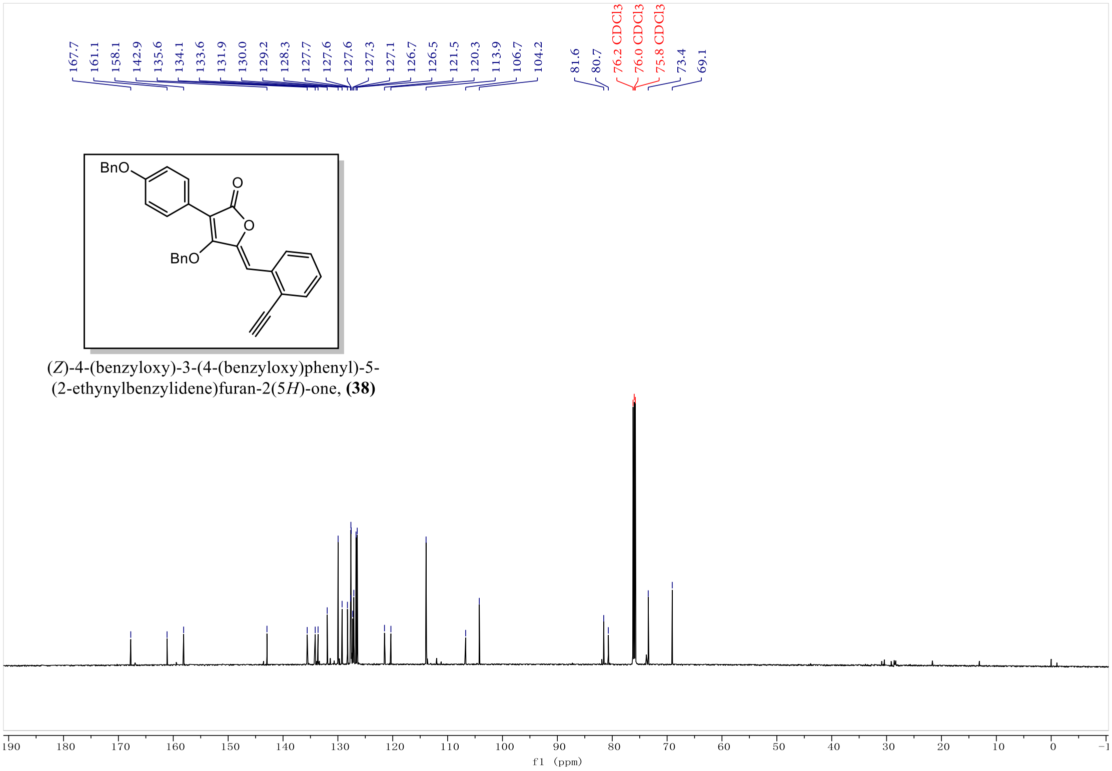

^{1}H NMR (400 MHz, Chloroform-*d*)

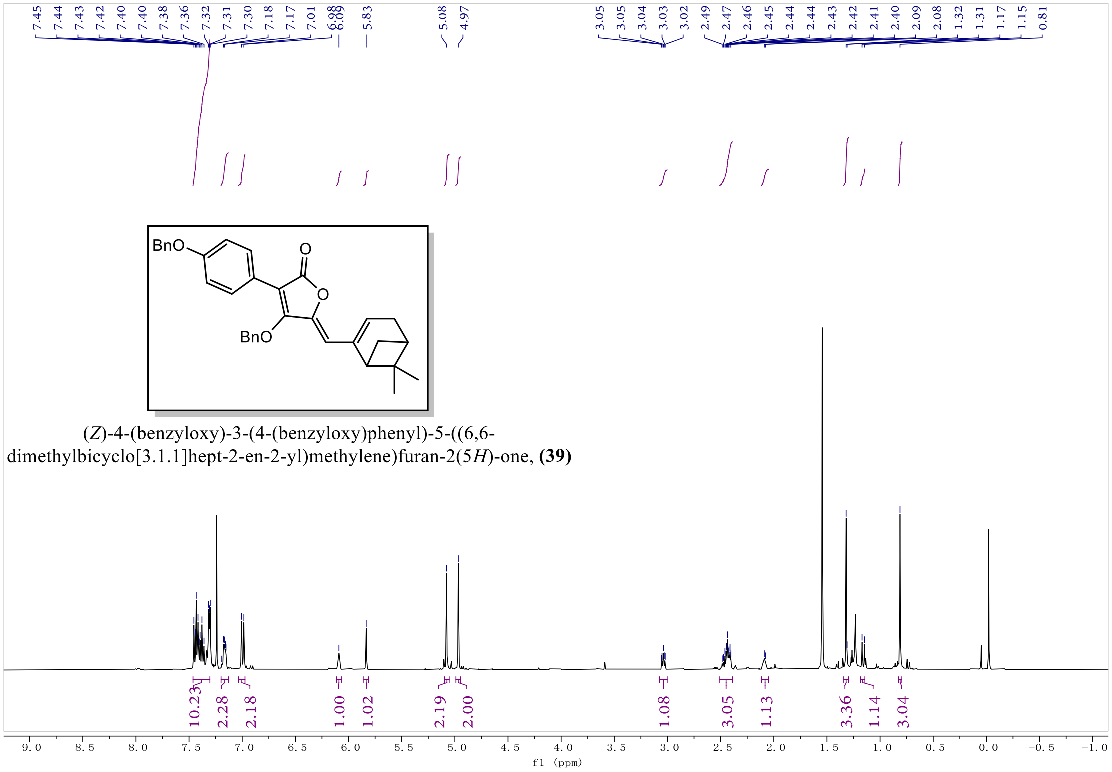

^{13}C NMR (101 MHz, Chloroform-*d*)

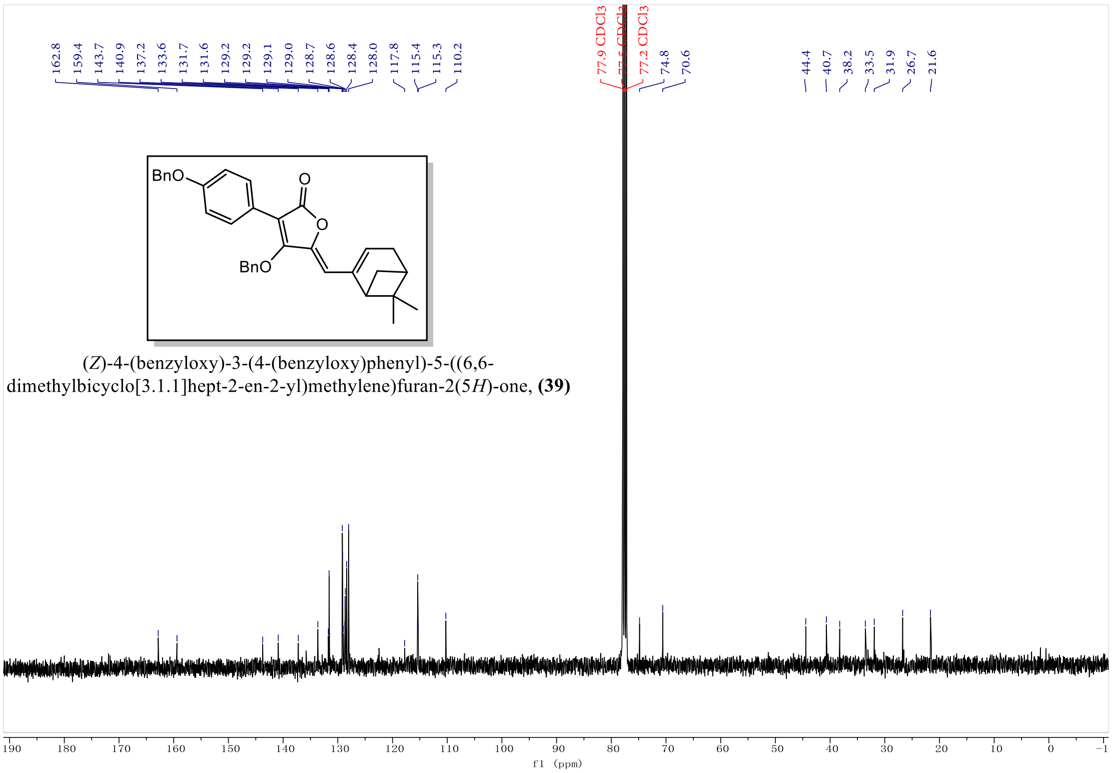

^{1}H NMR (400 MHz, Chloroform-*d*)

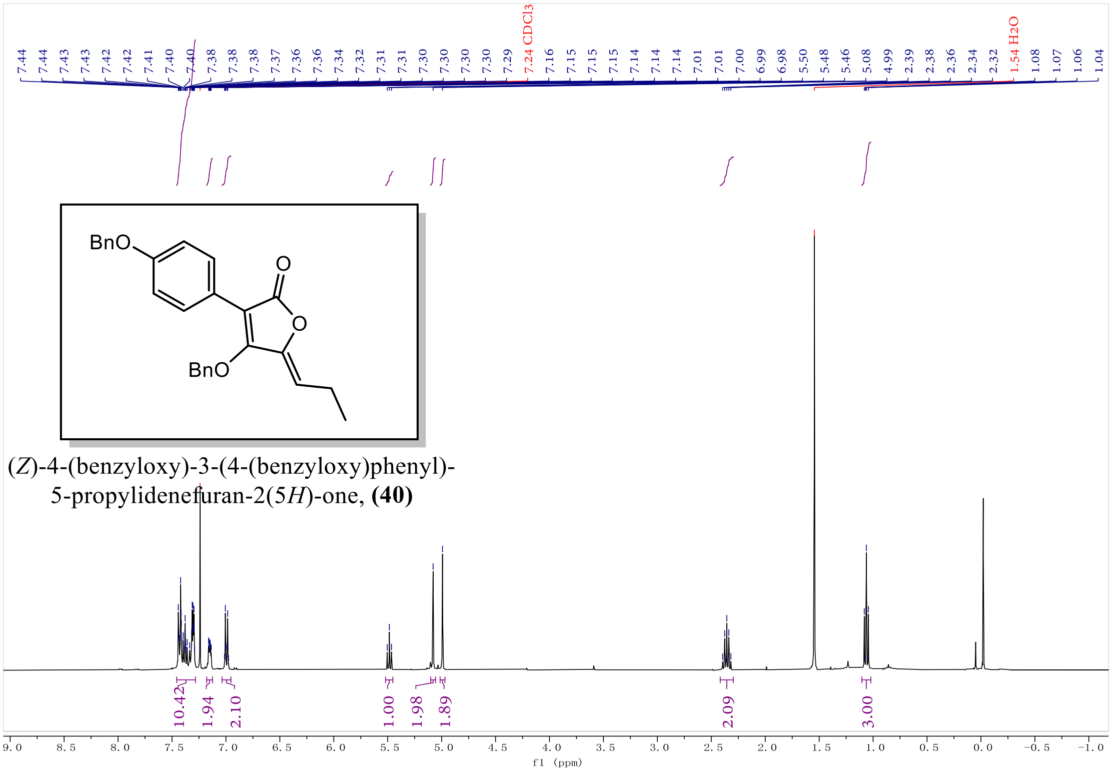

^{13}C NMR (101 MHz, Chloroform-*d*)

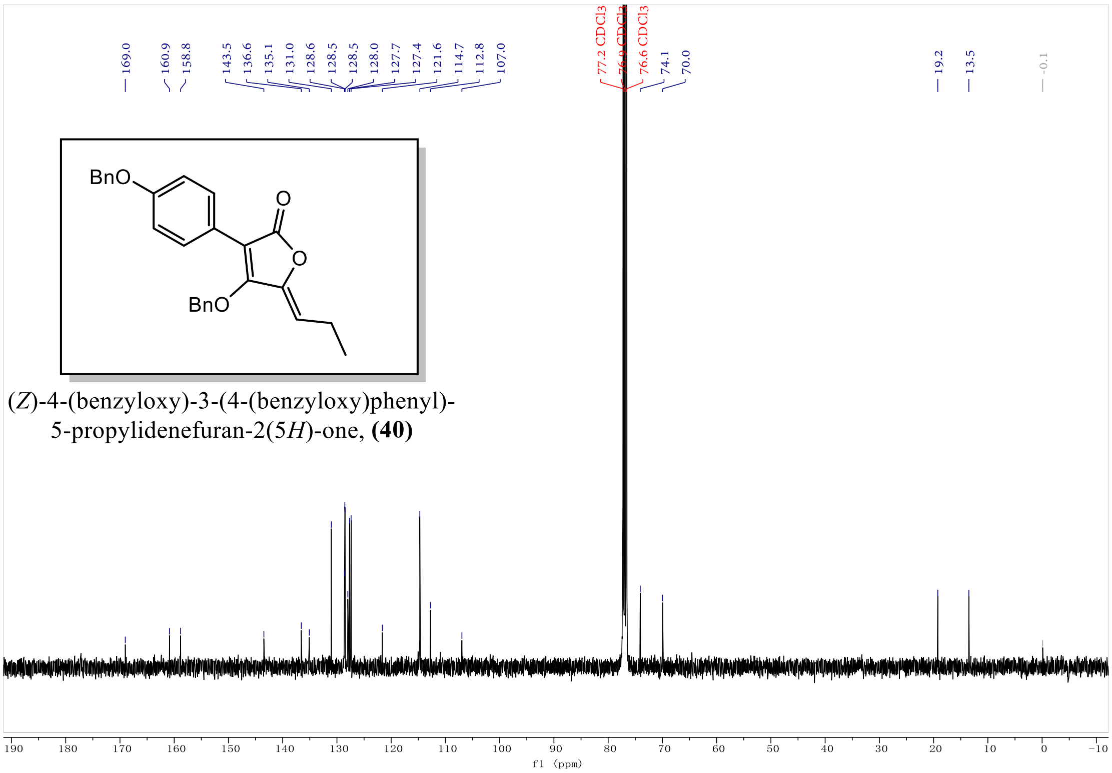

^{1}H NMR (400 MHz, Chloroform-*d*)

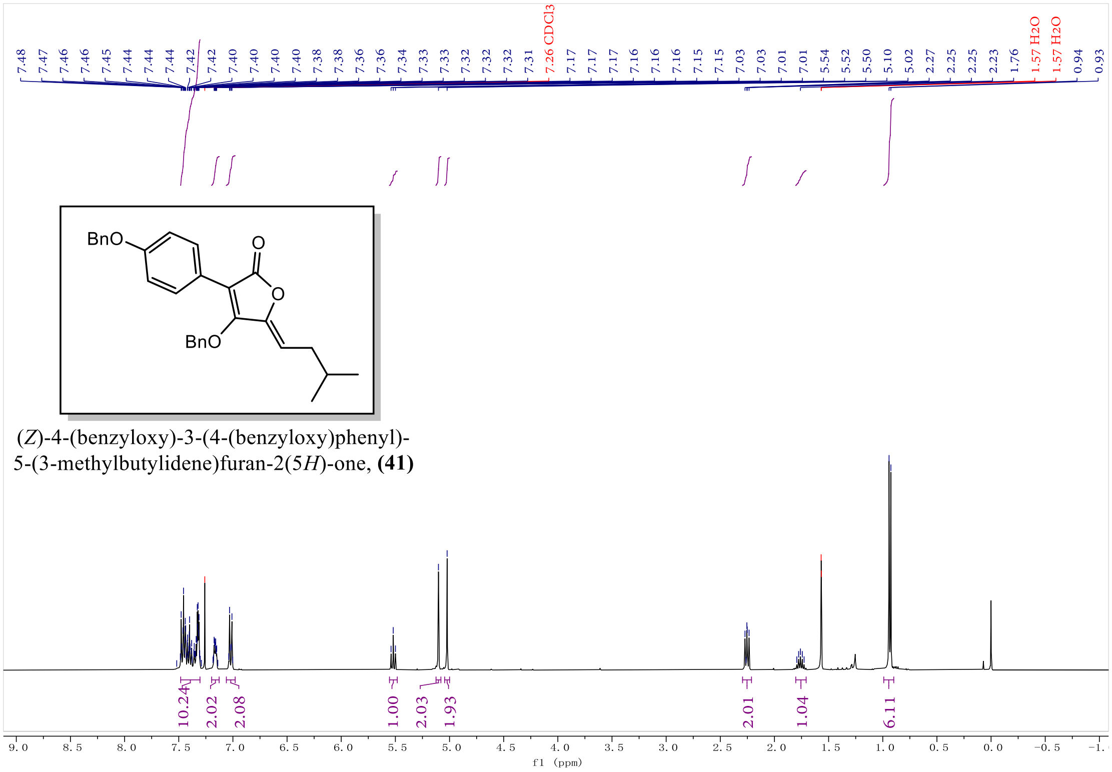

(*Z*)-4-(benzyloxy)-3-(4-(benzyloxy)phenyl)-5-(3-methylbutylidene)furan-2(5*H*)-one, **(41)**

^{13}C NMR (101 MHz, Chloroform-*d*)

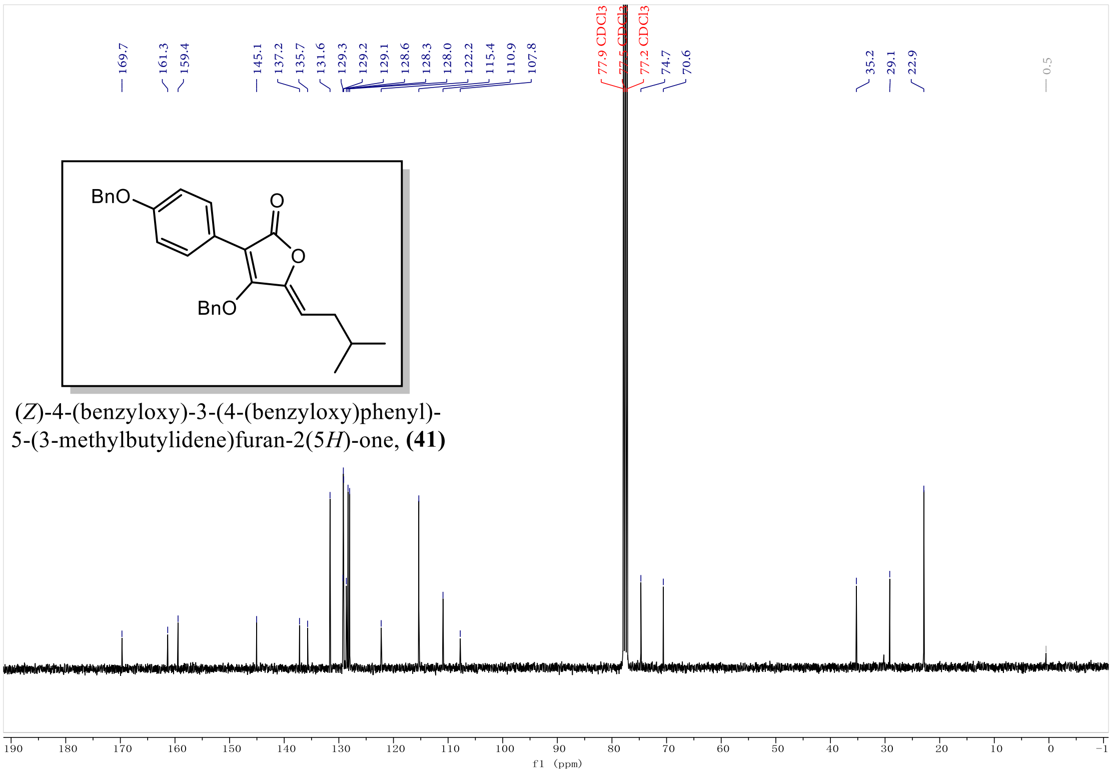

(*Z*)-4-(benzyloxy)-3-(4-(benzyloxy)phenyl)-5-(3-methylbutylidene)furan-2(5*H*)-one, **(41)**

^{1}H NMR (400 MHz, Chloroform-*d*)

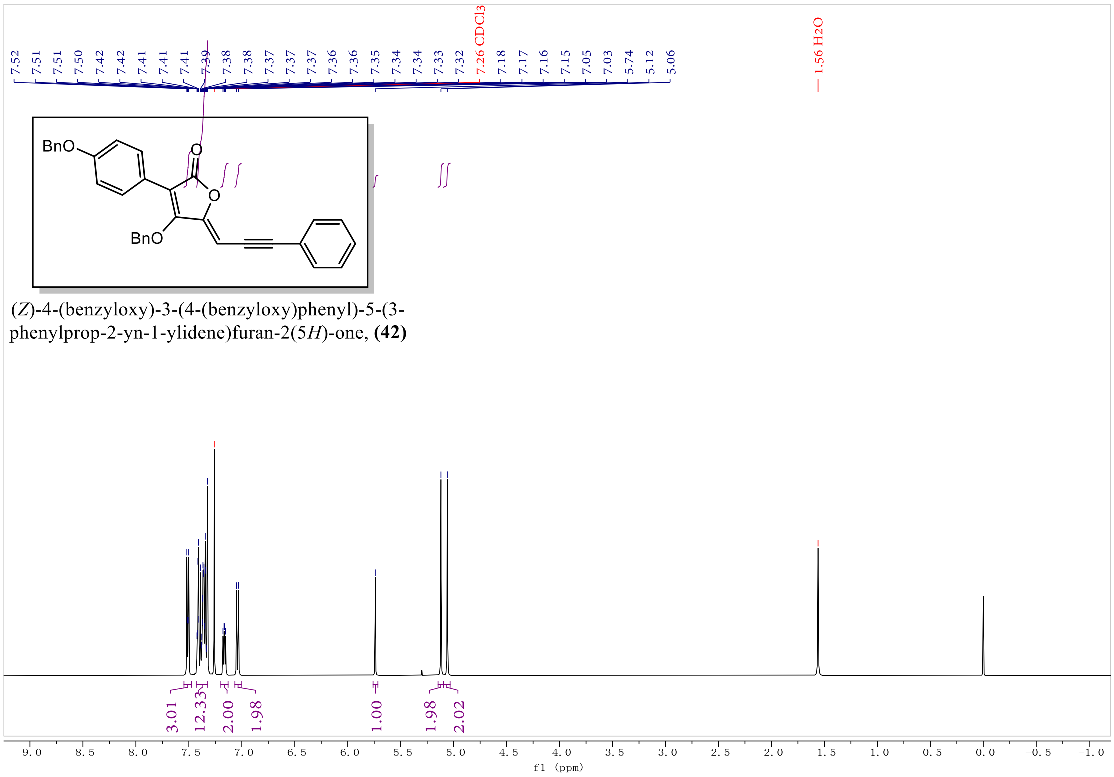

(*Z*)-4-(benzyloxy)-3-(4-(benzyloxy)phenyl)-5-(3-phenylprop-2-yn-1-ylidene)furan-2(5*H*)-one, **(42)**

^{13}C NMR (101 MHz, Chloroform-*d*)

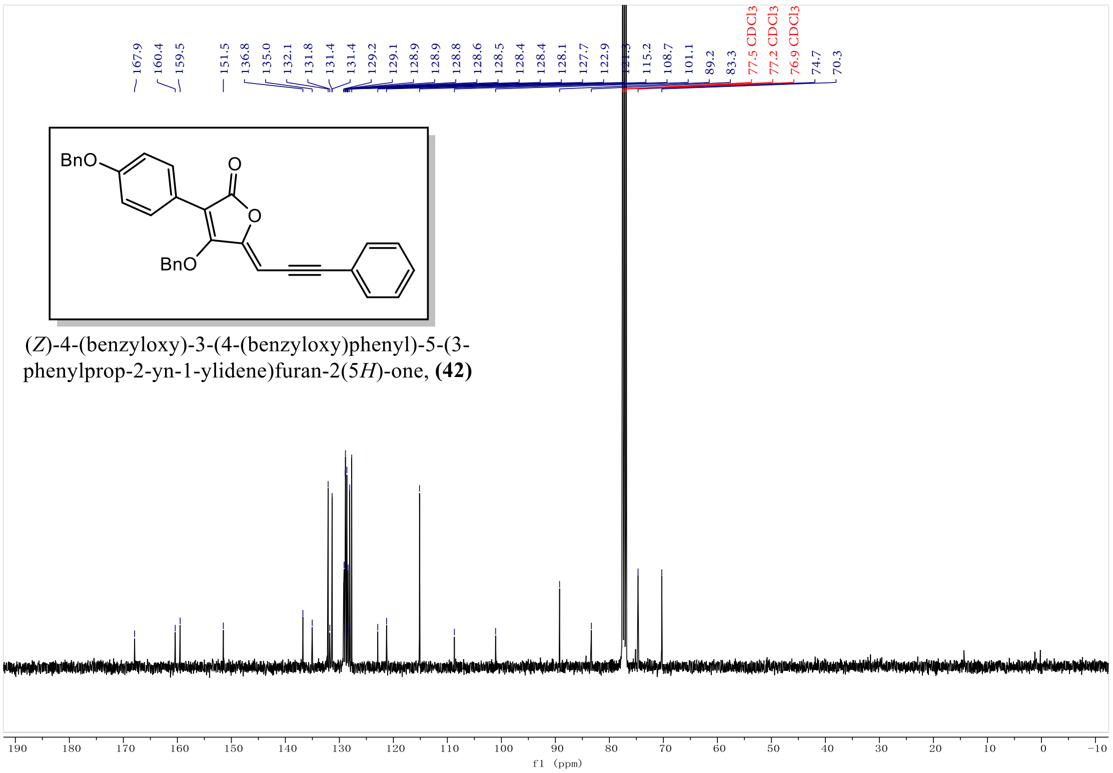

(*Z*)-4-(benzyloxy)-3-(4-(benzyloxy)phenyl)-5-(3-phenylprop-2-yn-1-ylidene)furan-2(5*H*)-one, **(42)**

^{1}H NMR (600 MHz, Chloroform-*d*)

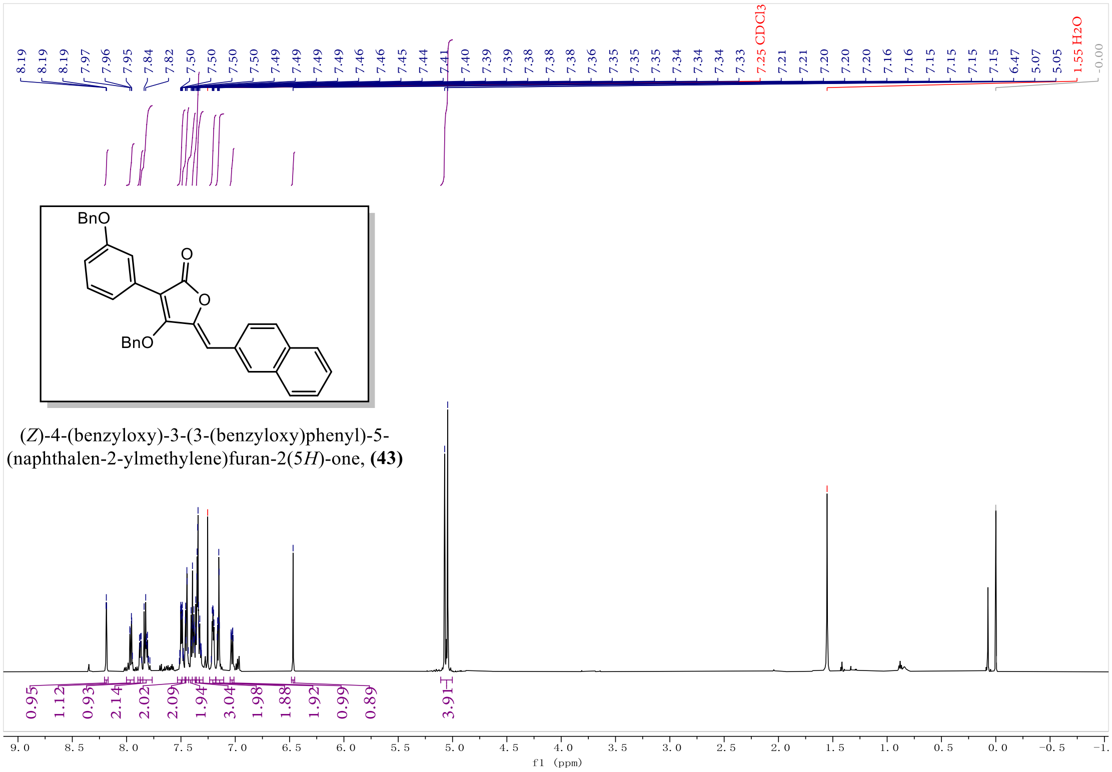

(*Z*)-4-(benzyloxy)-3-(3-(benzyloxy)phenyl)-5-(naphthalen-2-ylmethylene)furan-2(5*H*)-one, **(43)**

^{13}C NMR (101 MHz, Chloroform-*d*)

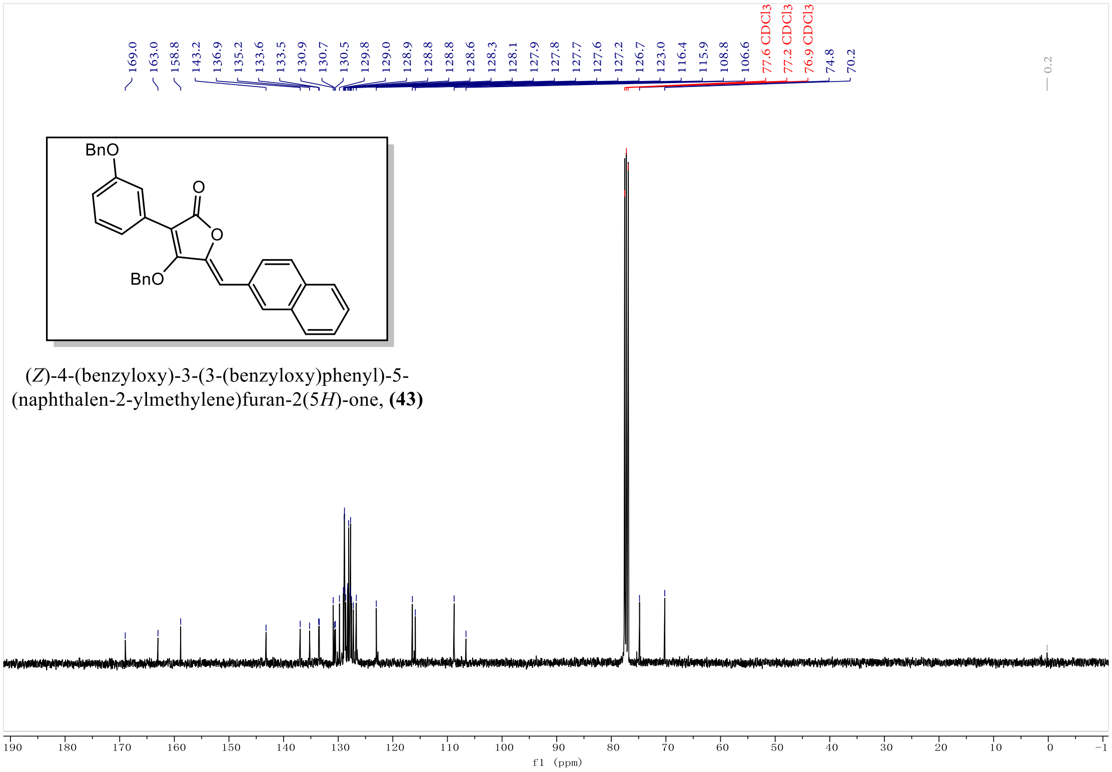

(*Z*)-4-(benzyloxy)-3-(3-(benzyloxy)phenyl)-5-(naphthalen-2-ylmethylene)furan-2(5*H*)-one, **(43)**

^{1}H NMR (600 MHz, Chloroform-*d*)

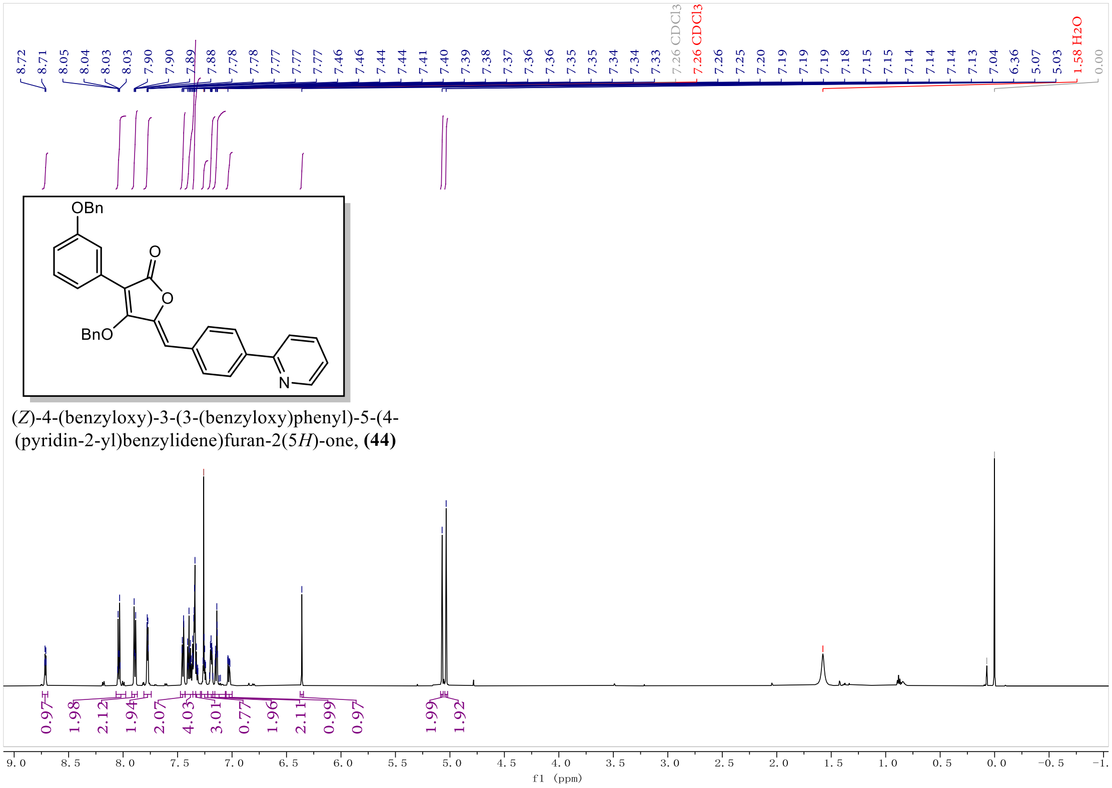

^{13}C NMR (101 MHz, Chloroform-*d*)

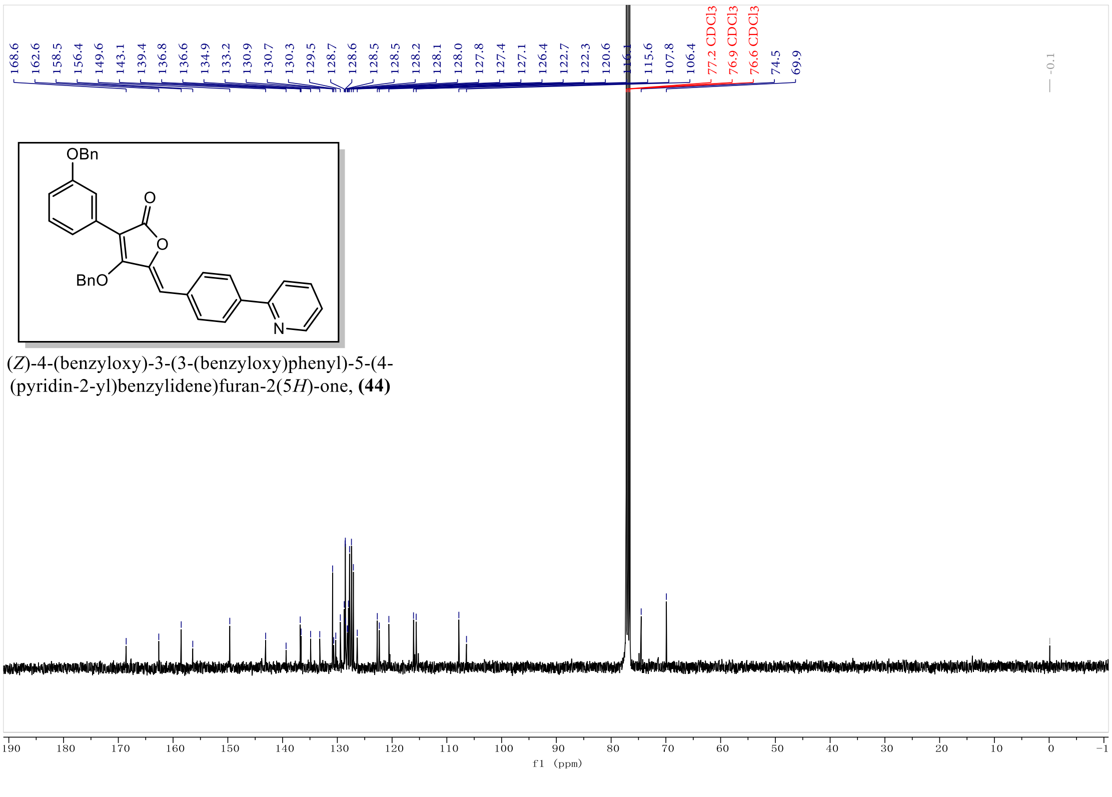

^{1}H NMR (600 MHz, Chloroform-*d*)

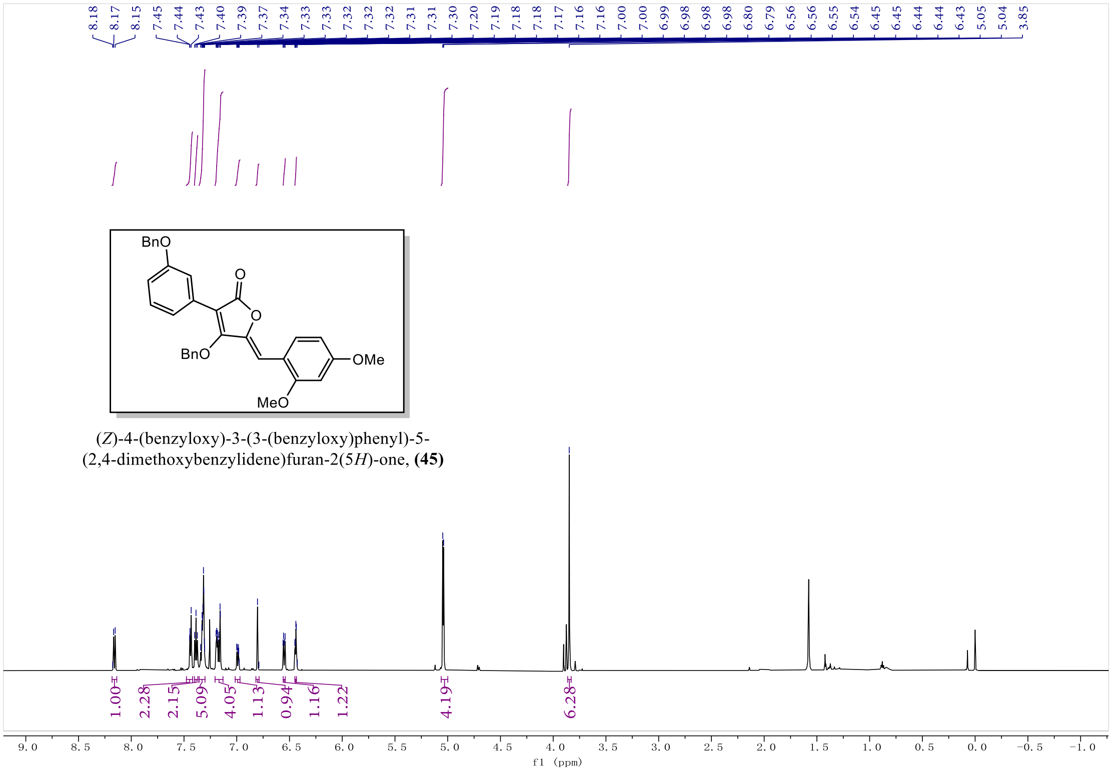

^{13}C NMR (101 MHz, DMSO-d_6)

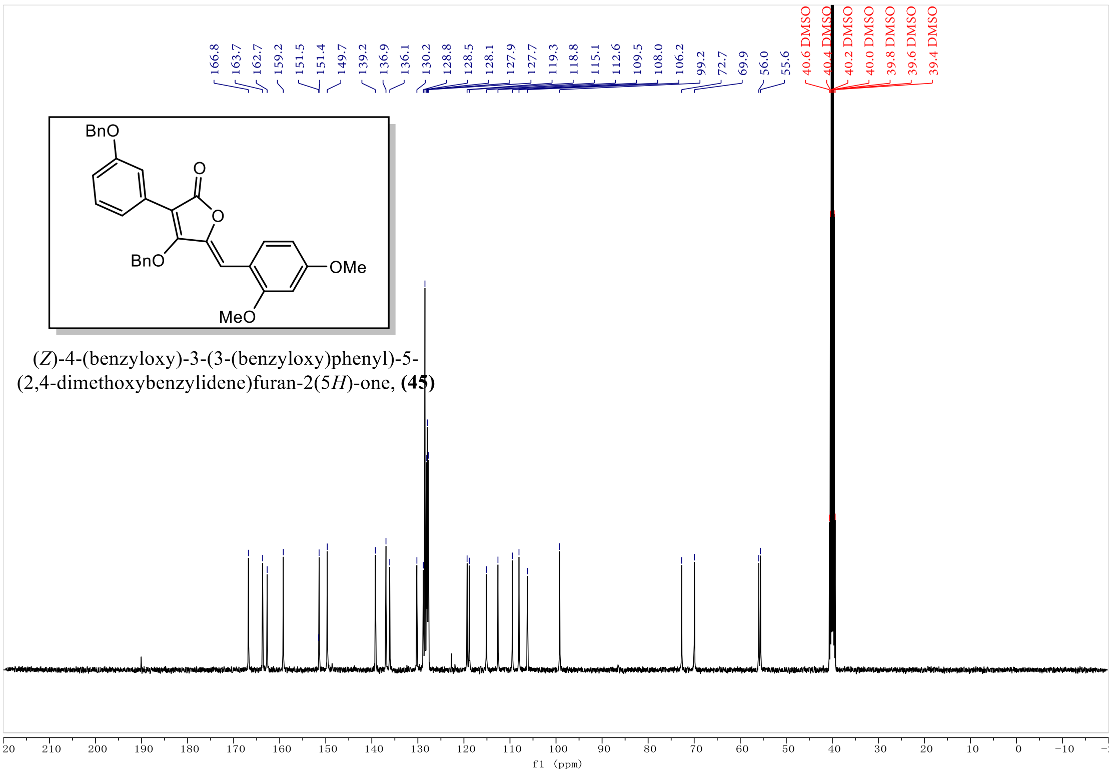

^{1}H NMR (600 MHz, Chloroform-*d*)

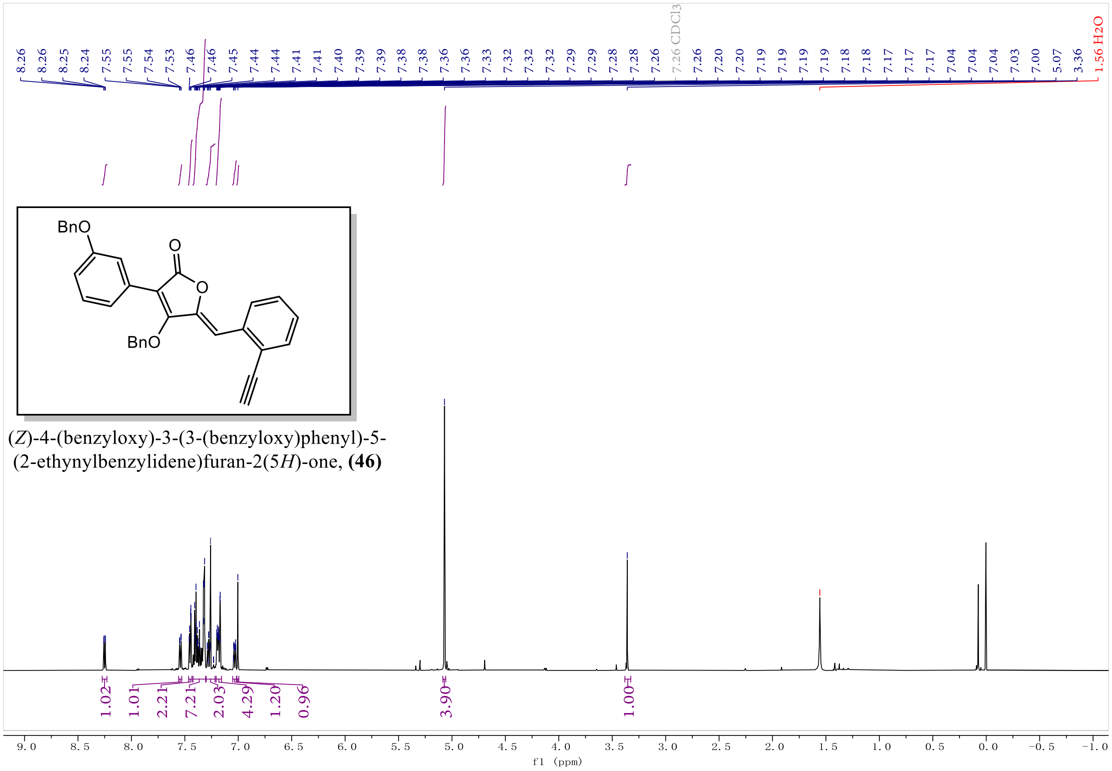

(*Z*)-4-(benzyloxy)-3-(3-(benzyloxy)phenyl)-5-(2-ethynylbenzylidene)furan-2(5*H*)-one, **(46)**

^{13}C NMR (101 MHz, Chloroform-*d*)

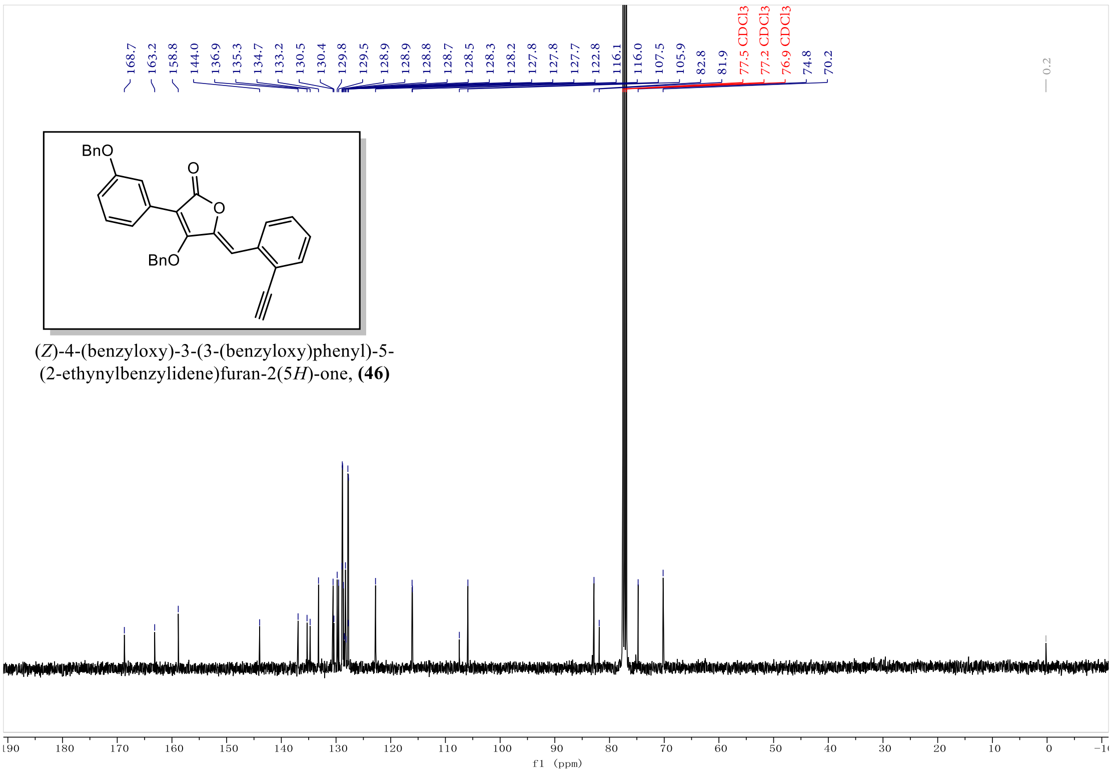

(*Z*)-4-(benzyloxy)-3-(3-(benzyloxy)phenyl)-5-(2-ethynylbenzylidene)furan-2(5*H*)-one, **(46)**

^{1}H NMR (600 MHz, Chloroform-*d*)

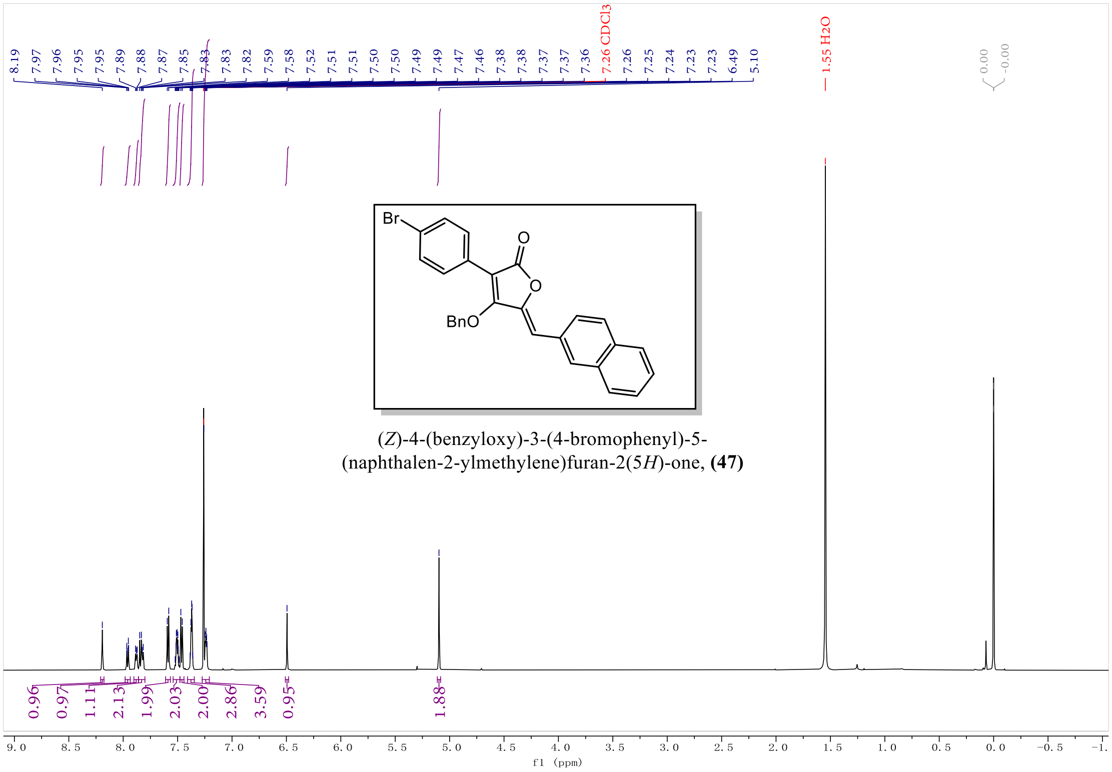

(*Z*)-4-(benzyloxy)-3-(4-bromophenyl)-5-(naphthalen-2-ylmethylene)furan-2(5*H*)-one, **(47)**

^{13}C NMR (101 MHz, Chloroform-*d*)

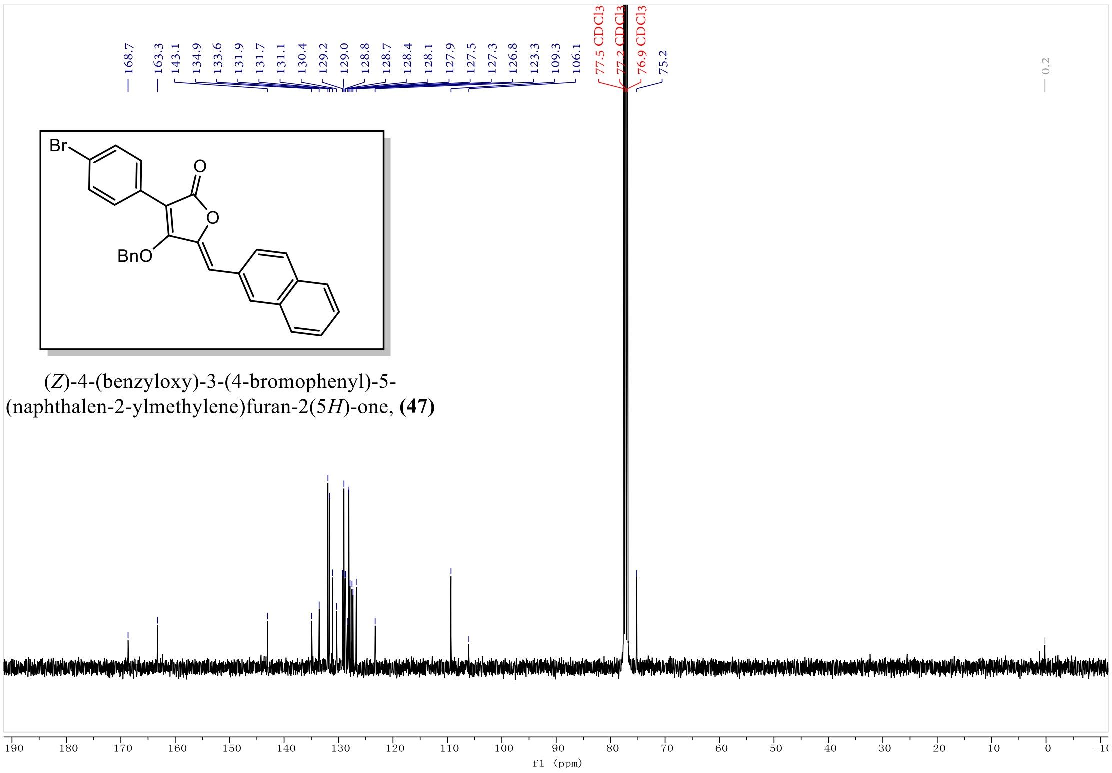

(*Z*)-4-(benzyloxy)-3-(4-bromophenyl)-5-(naphthalen-2-ylmethylene)furan-2(5*H*)-one, **(47)**

^{1}H NMR (600 MHz, Chloroform-*d*)

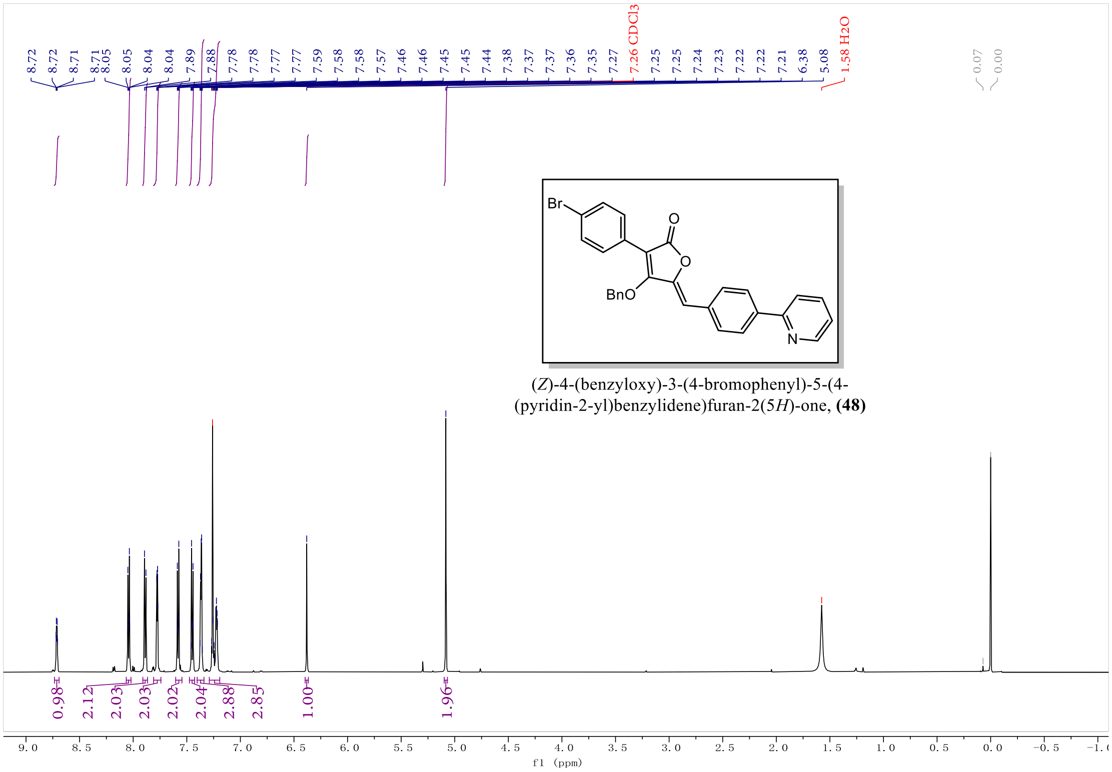

(*Z*)-4-(benzyloxy)-3-(4-bromophenyl)-5-(4-(pyridin-2-yl)benzylidene)furan-2(5*H*)-one, **(48)**

^{13}C NMR (101 MHz, Chloroform-*d*)

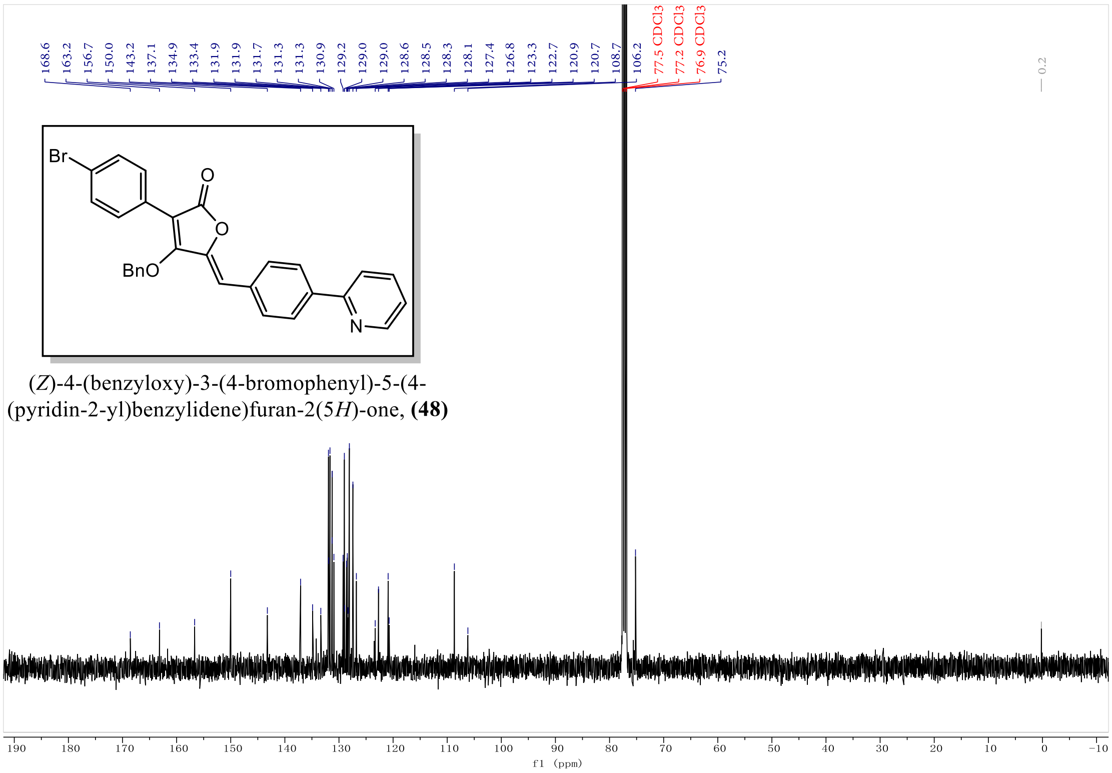

(*Z*)-4-(benzyloxy)-3-(4-bromophenyl)-5-(4-(pyridin-2-yl)benzylidene)furan-2(5*H*)-one, **(48)**

^{1}H NMR (600 MHz, Chloroform-*d*)

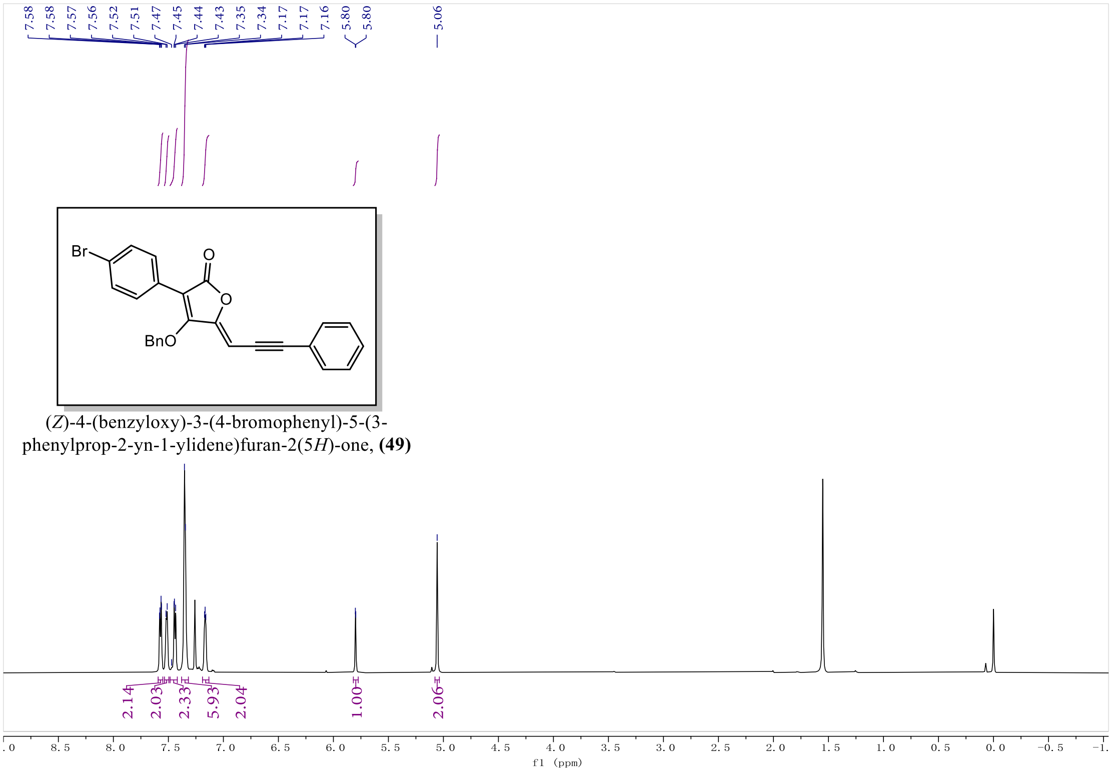

^{13}C NMR (101 MHz, Chloroform-*d*)

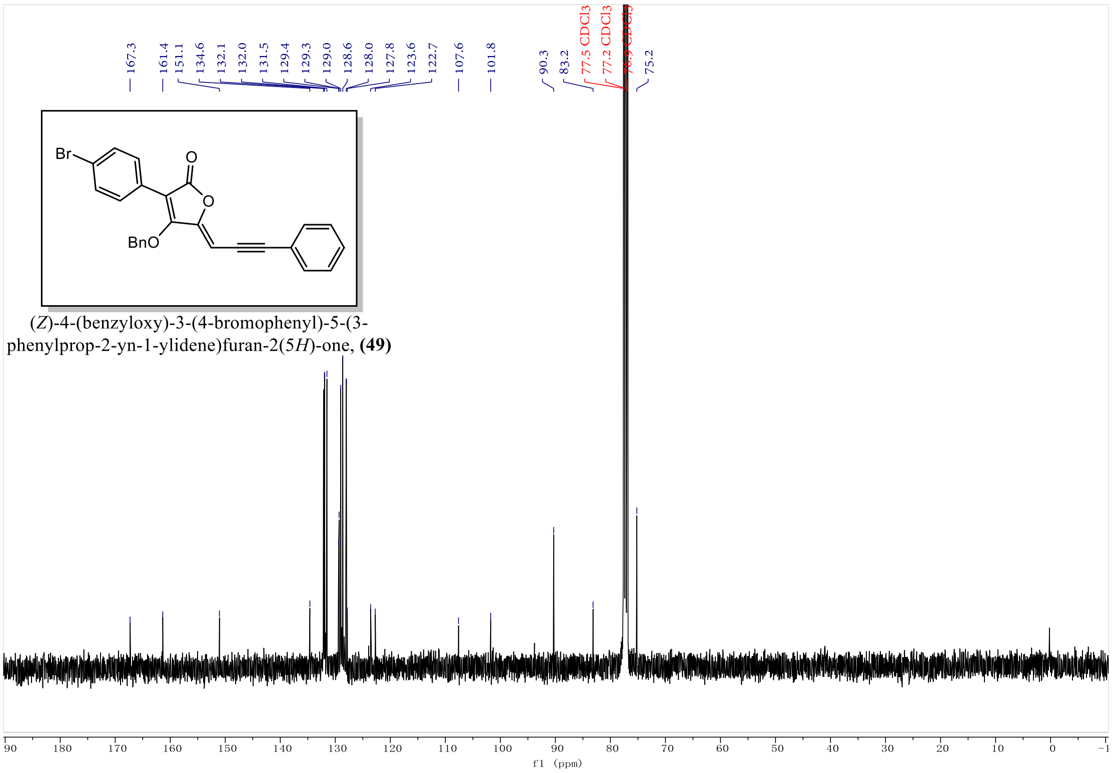

^{1}H NMR (600 MHz, Chloroform-*d*)

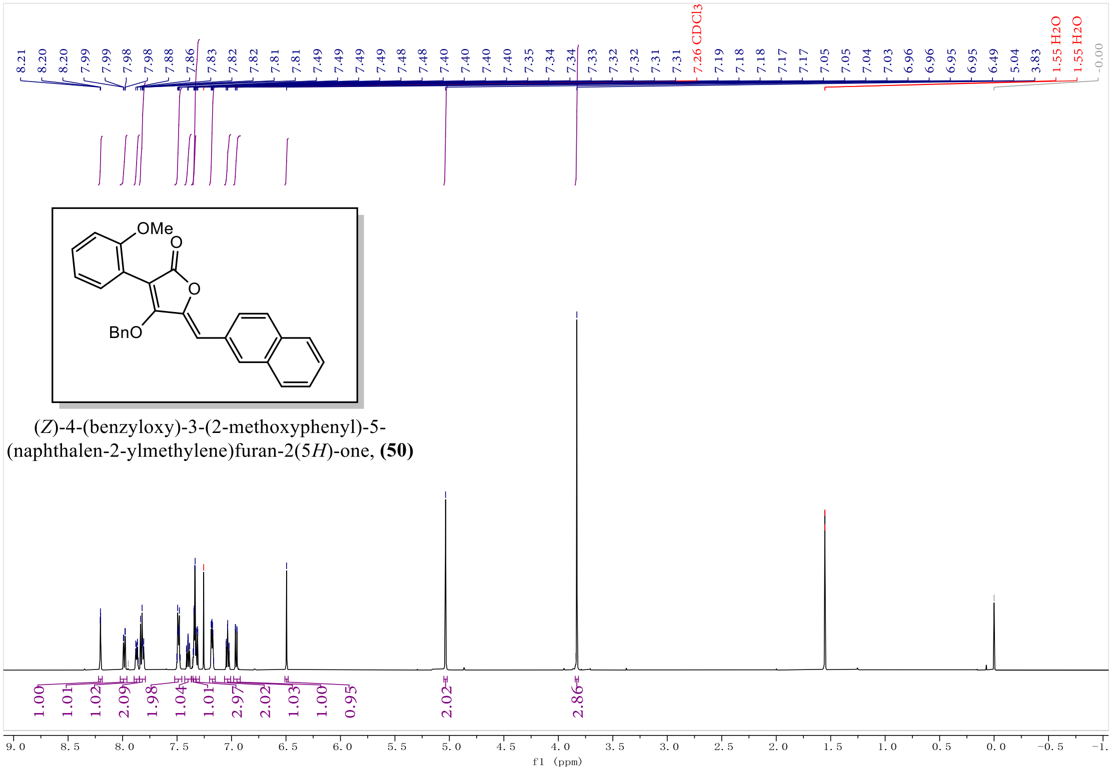

(*Z*)-4-(benzyloxy)-3-(2-methoxyphenyl)-5-(naphthalen-2-ylmethylene)furan-2(5*H*)-one, **(50)**

^{13}C NMR (101 MHz, Chloroform-*d*)

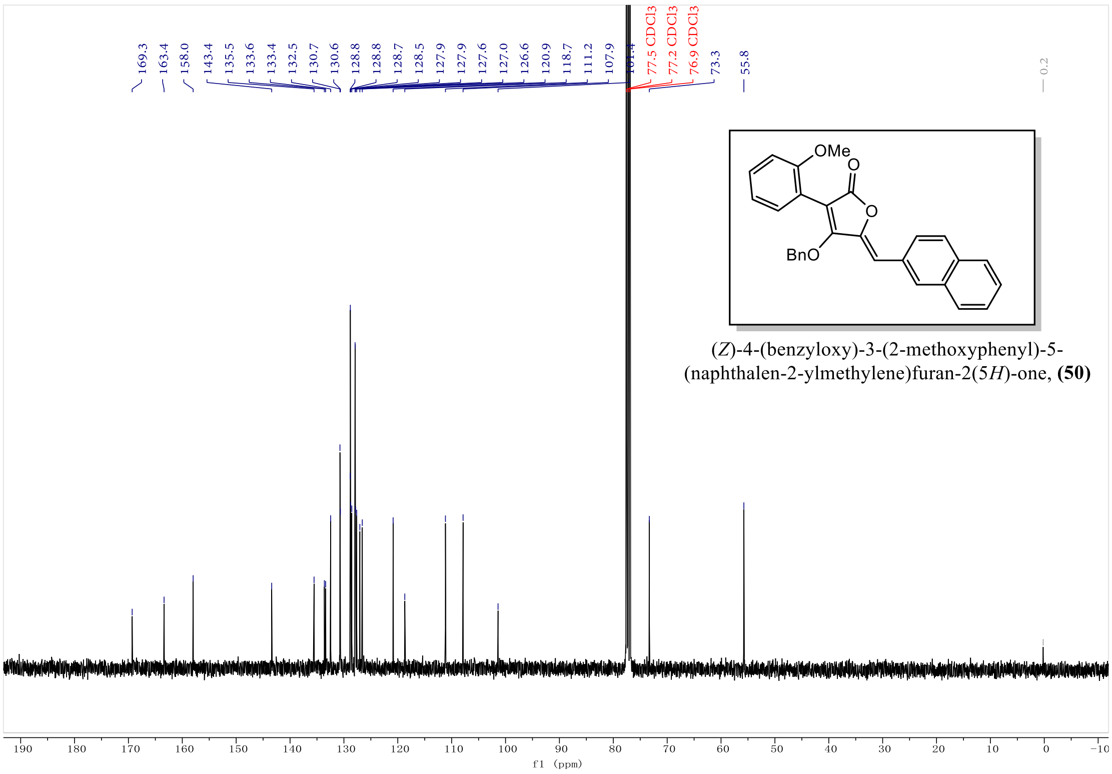

(*Z*)-4-(benzyloxy)-3-(2-methoxyphenyl)-5-(naphthalen-2-ylmethylene)furan-2(5*H*)-one, **(50)**

^{1}H NMR (600 MHz, Chloroform-*d*)

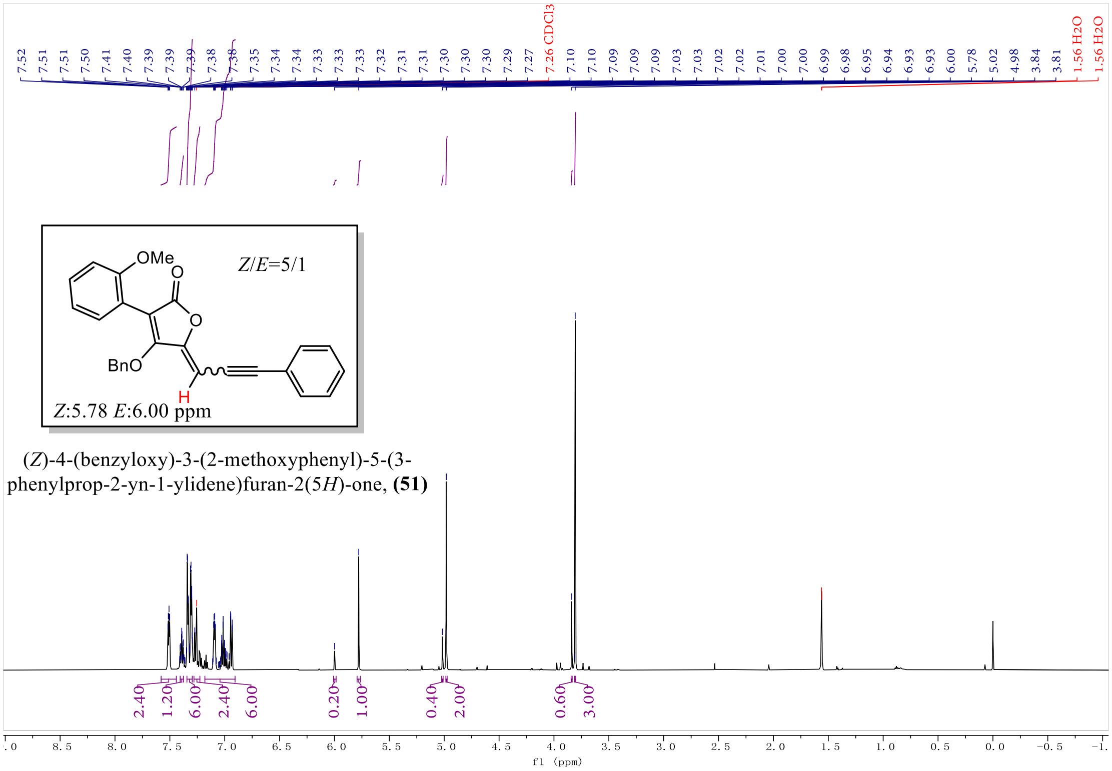

^1H NMR (600 MHz, Chloroform-*d*)

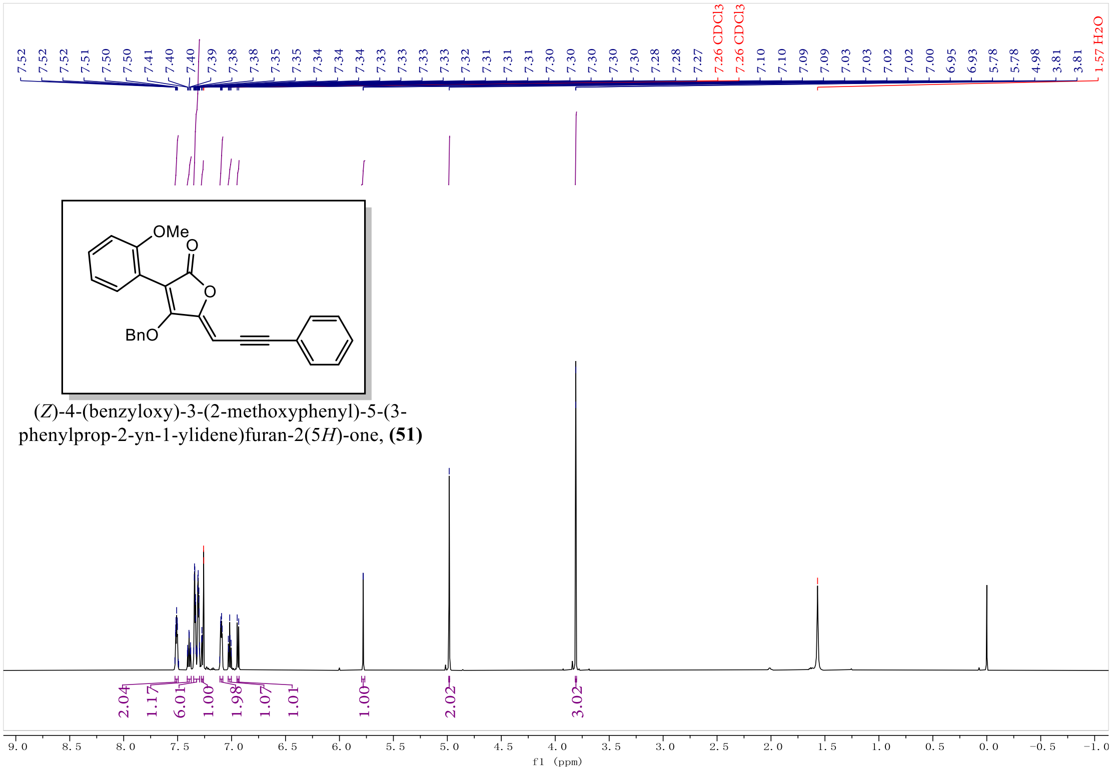

(*Z*)-4-(benzyloxy)-3-(2-methoxyphenyl)-5-(3-phenylprop-2-yn-1-ylidene)furan-2(5*H*)-one, **(51)**

^{13}C NMR (151 MHz, Chloroform-*d*)

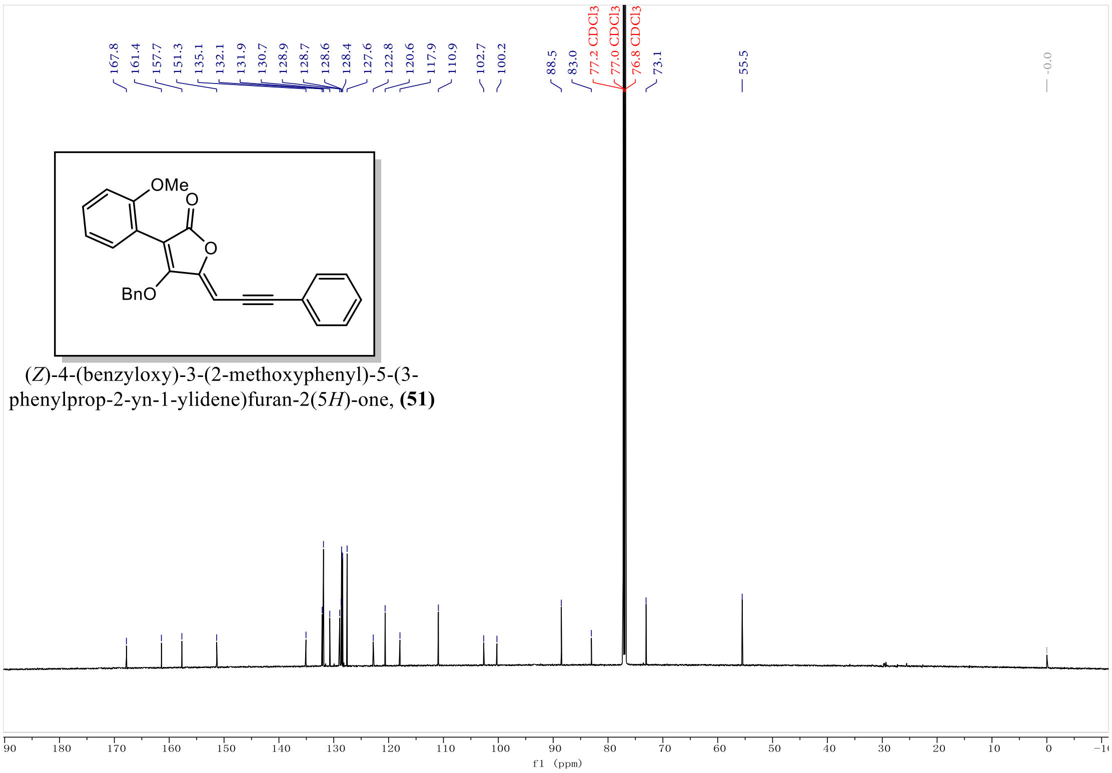

(*Z*)-4-(benzyloxy)-3-(2-methoxyphenyl)-5-(3-phenylprop-2-yn-1-ylidene)furan-2(5*H*)-one, **(51)**

^{1}H NMR (400 MHz, Chloroform-*d*)

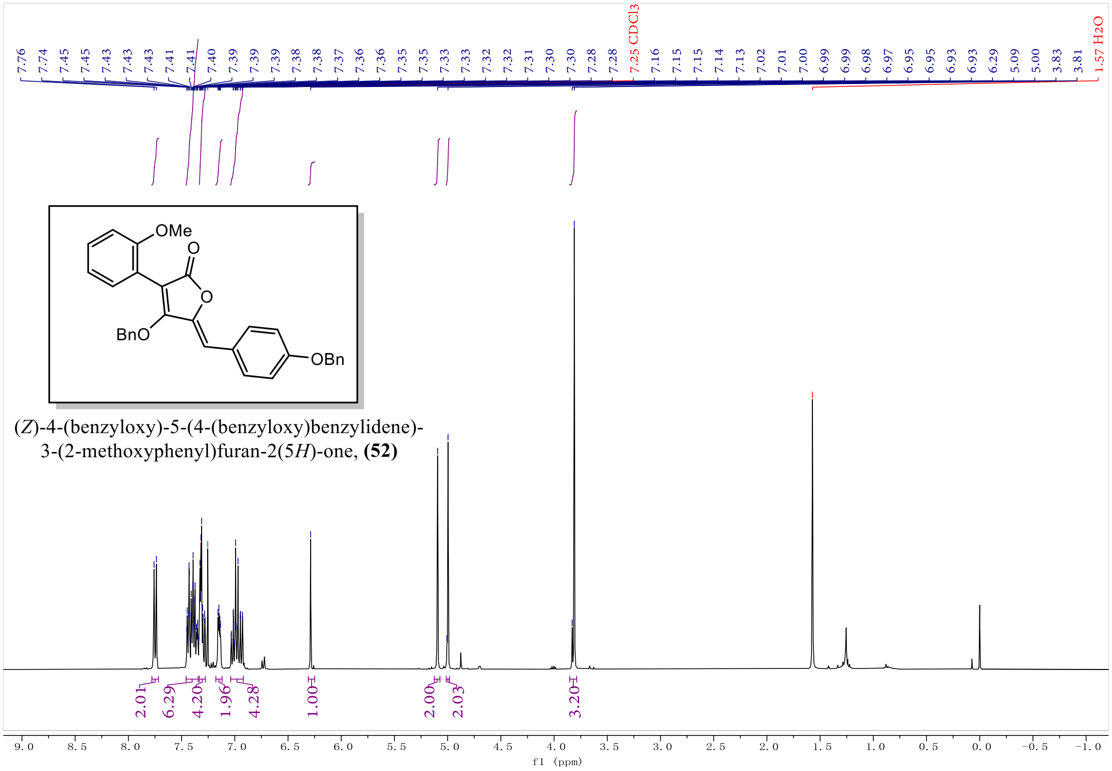

^{13}C NMR (101 MHz, Chloroform-*d*)

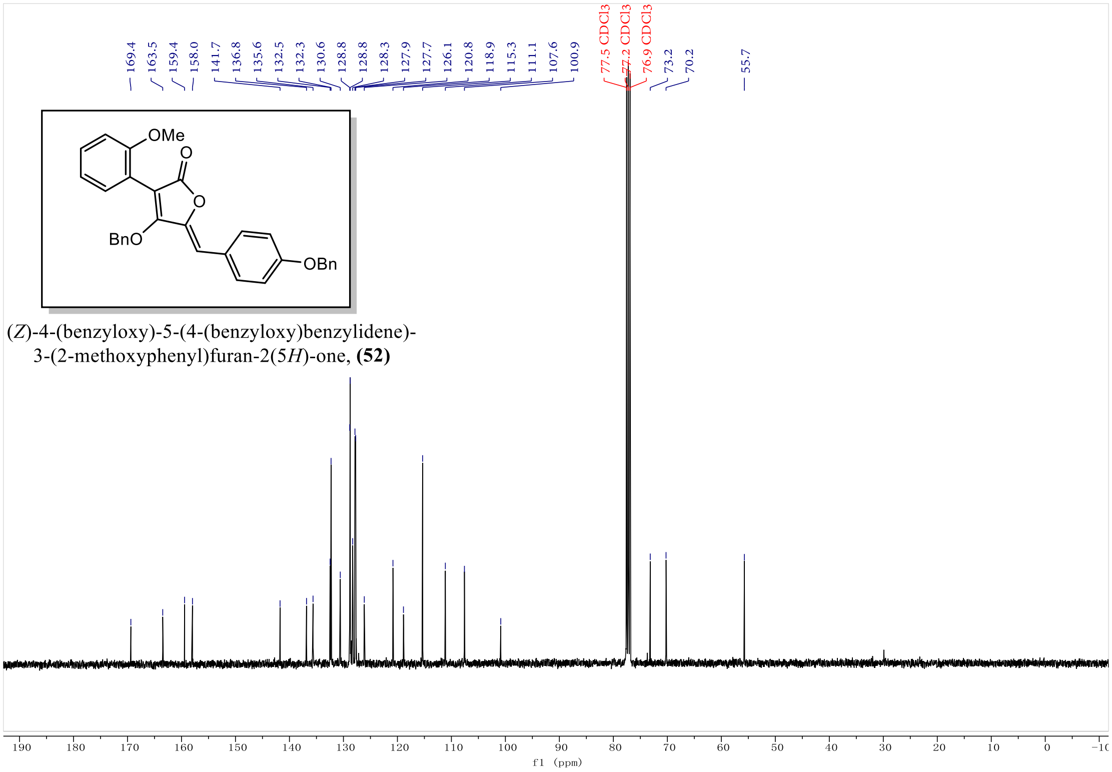

^{1}H NMR (600 MHz, Chloroform-*d*)

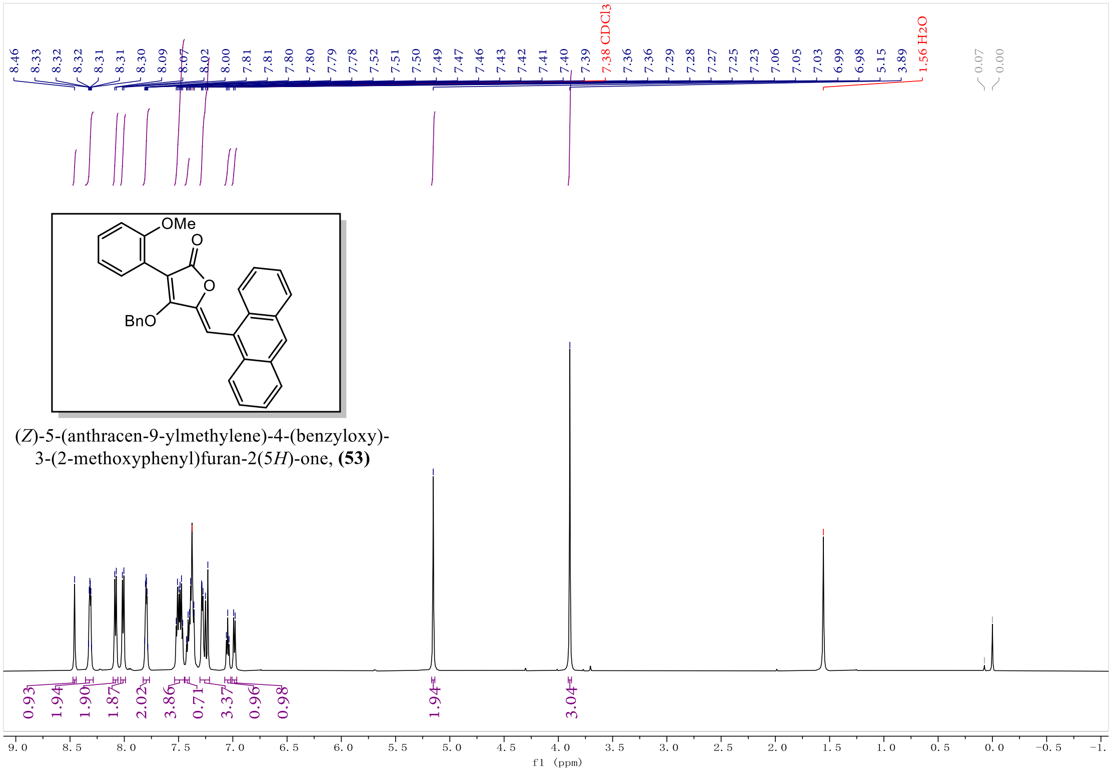

^{13}C NMR (101 MHz, Chloroform-*d*)

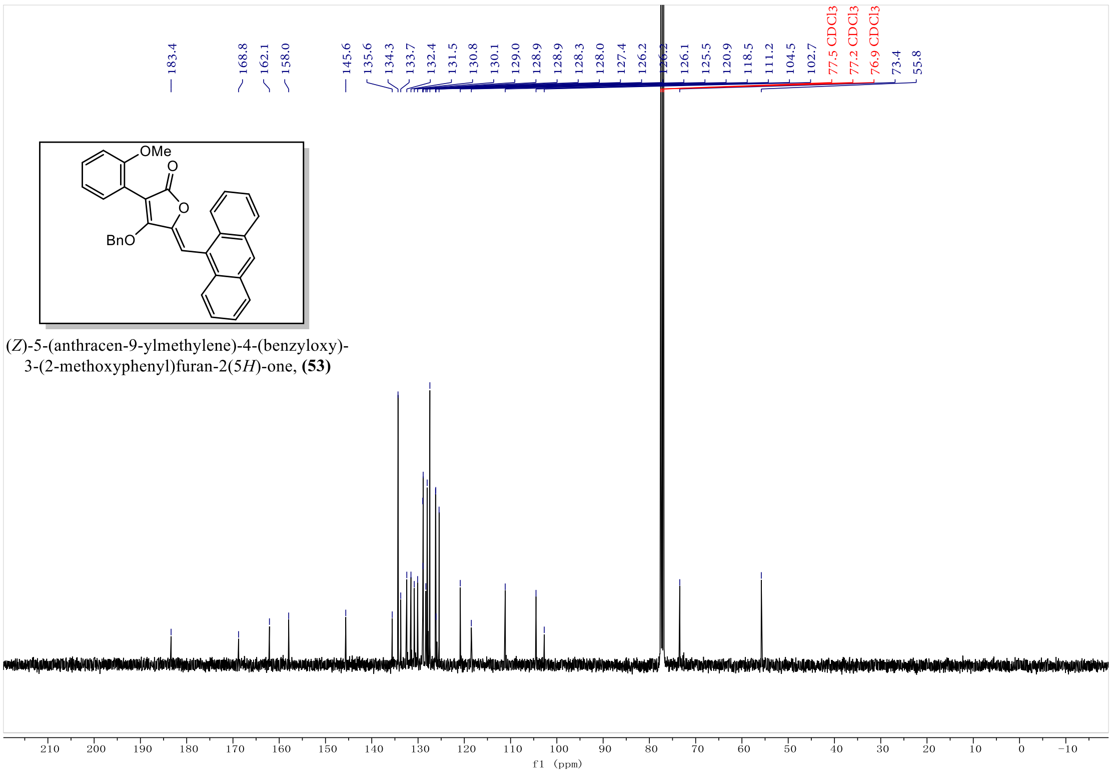

^{1}H NMR (600 MHz, Pyridine-d_5)

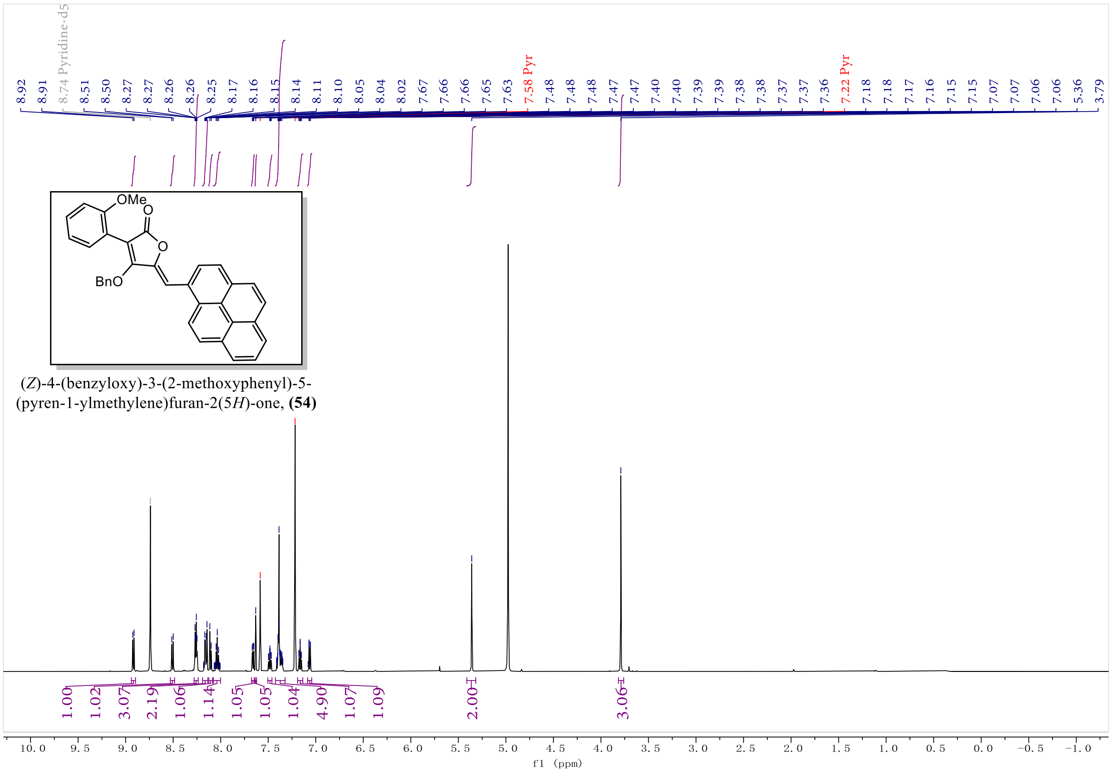

(*Z*)-4-(benzyloxy)-3-(2-methoxyphenyl)-5-(pyren-1-ylmethylene)furan-2(5*H*)-one, **(54)**

^{13}C NMR (151 MHz, Pyridine-d_5)

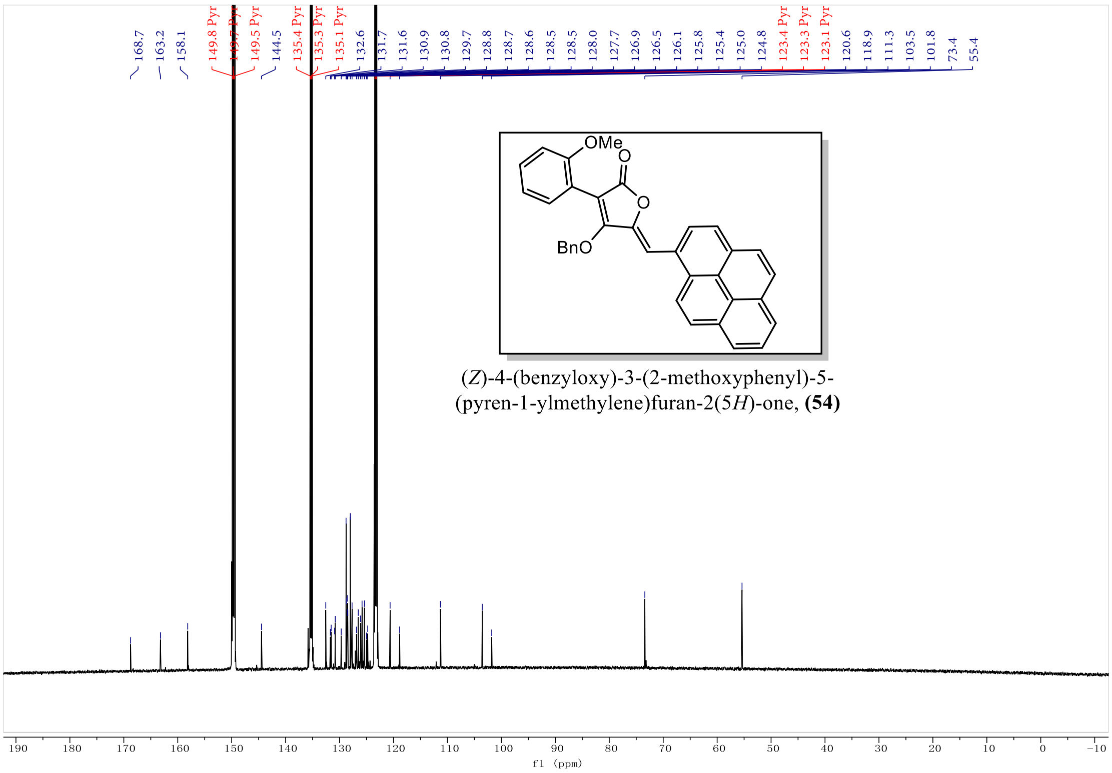

(*Z*)-4-(benzyloxy)-3-(2-methoxyphenyl)-5-(pyren-1-ylmethylene)furan-2(5*H*)-one, **(54)**

^{1}H NMR (600 MHz, Chloroform-*d*)

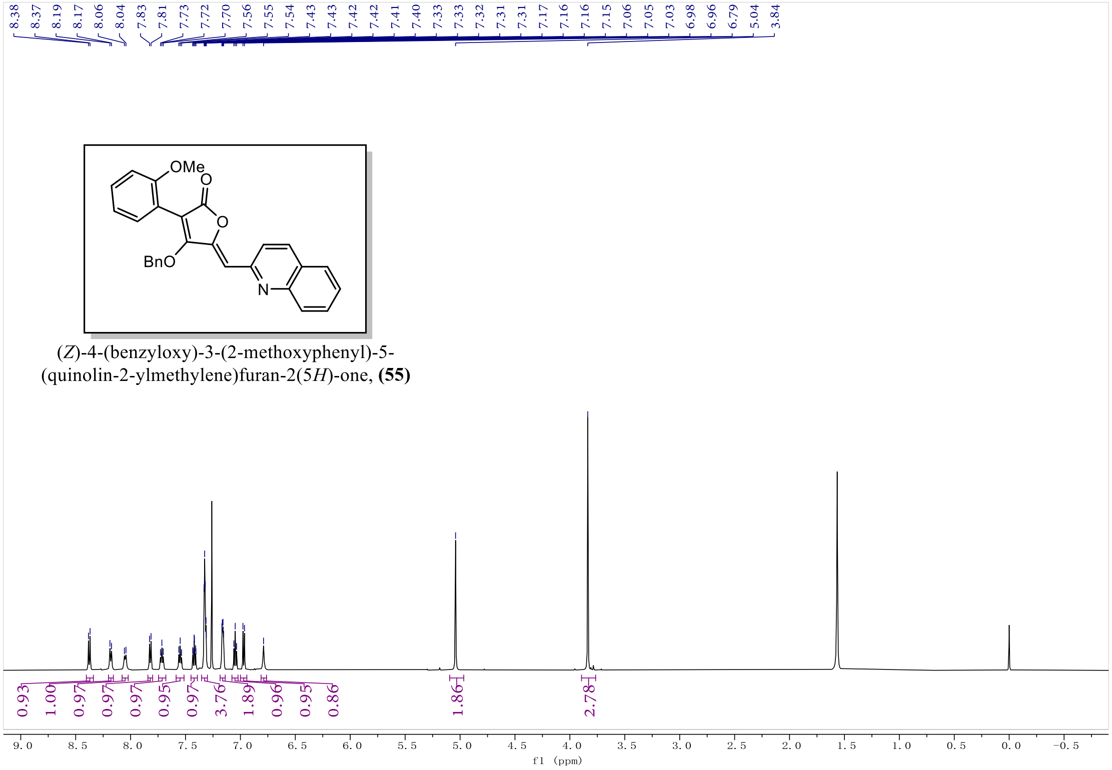

(*Z*)-4-(benzyloxy)-3-(2-methoxyphenyl)-5-(quinolin-2-ylmethylene)furan-2(5*H*)-one, **(55)**

^{13}C NMR (151 MHz, Chloroform-*d*)

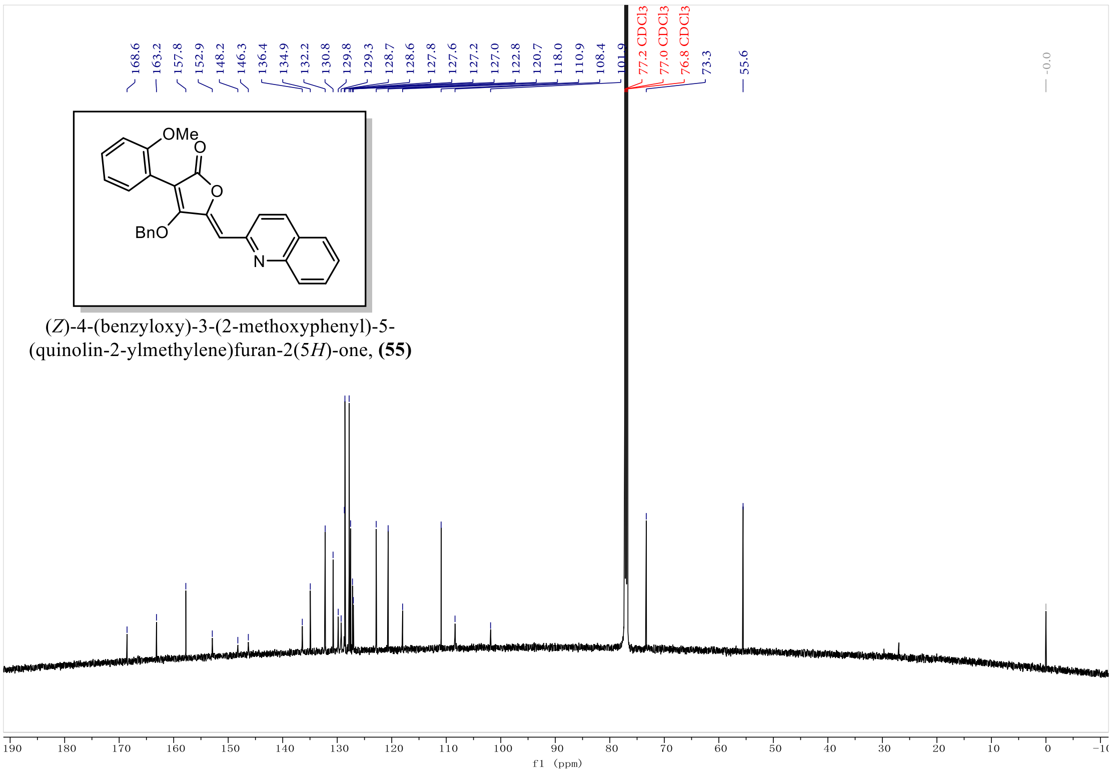

(*Z*)-4-(benzyloxy)-3-(2-methoxyphenyl)-5-(quinolin-2-ylmethylene)furan-2(5*H*)-one, **(55)**

^{1}H NMR (600 MHz, Chloroform-*d*)

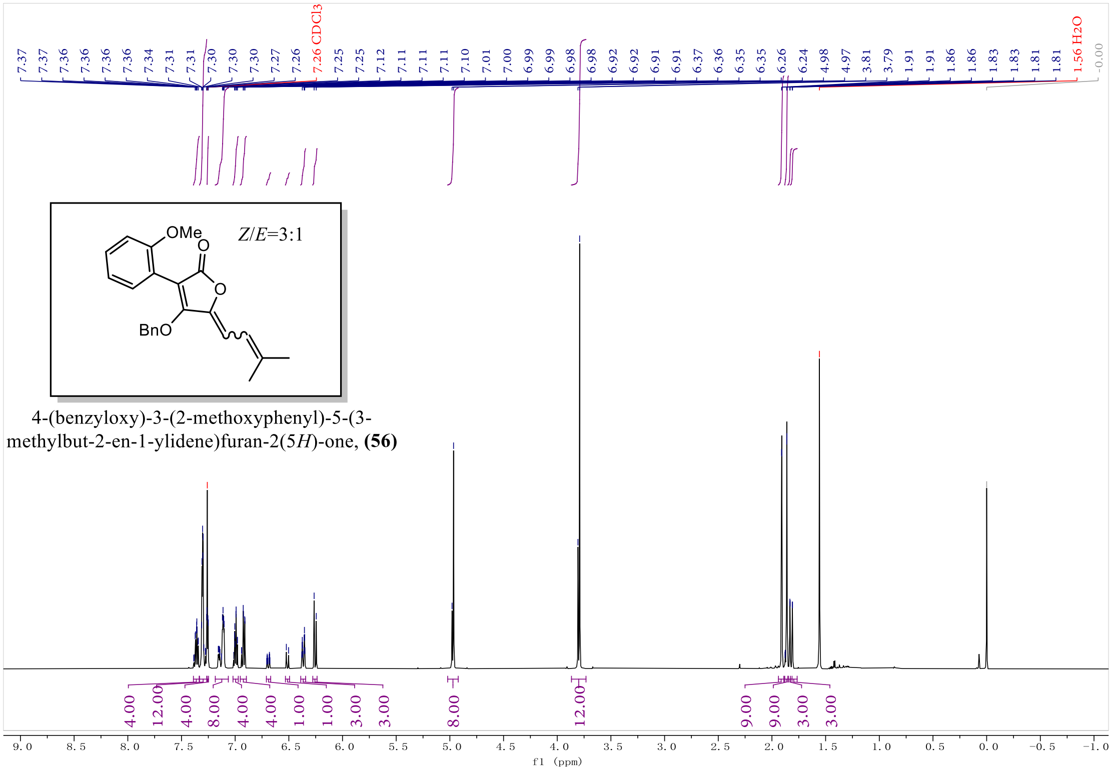

4-(benzyloxy)-3-(2-methoxyphenyl)-5-(3-methylbut-2-en-1-ylidene)furan-2(5*H*)-one, **(56)**

^{1}H NMR (600 MHz, Chloroform-*d*)

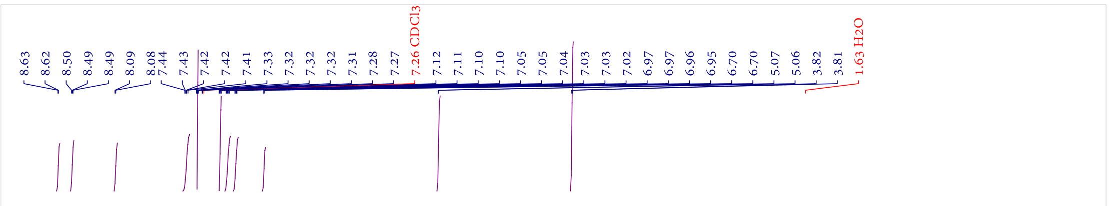

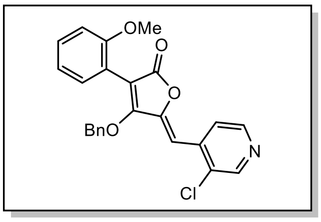

(*Z*)-4-(benzyloxy)-5-((3-chloropyridin-4-yl)methylene)-3-(2-methoxyphenyl)furan-2(5*H*)-one, **(57)**

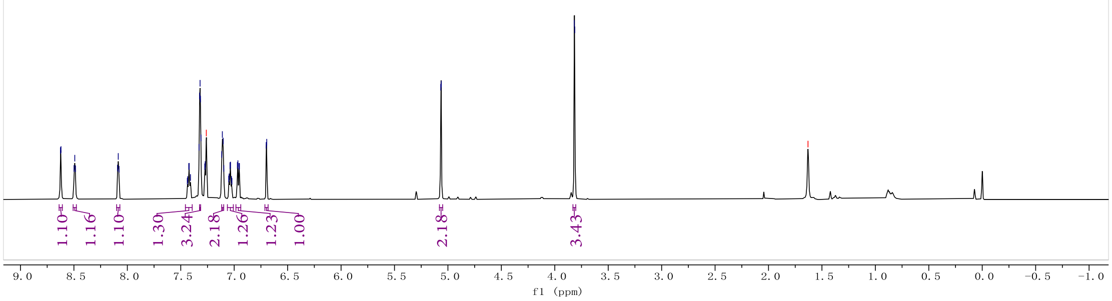

^{13}C NMR (101 MHz, Chloroform-*d*)

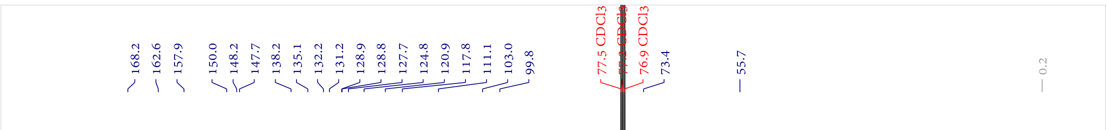

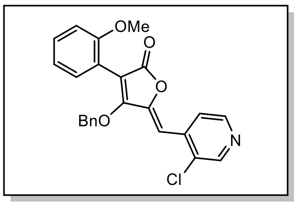

(*Z*)-4-(benzyloxy)-5-((3-chloropyridin-4-yl)methylene)-3-(2-methoxyphenyl)furan-2(5*H*)-one, **(57)**

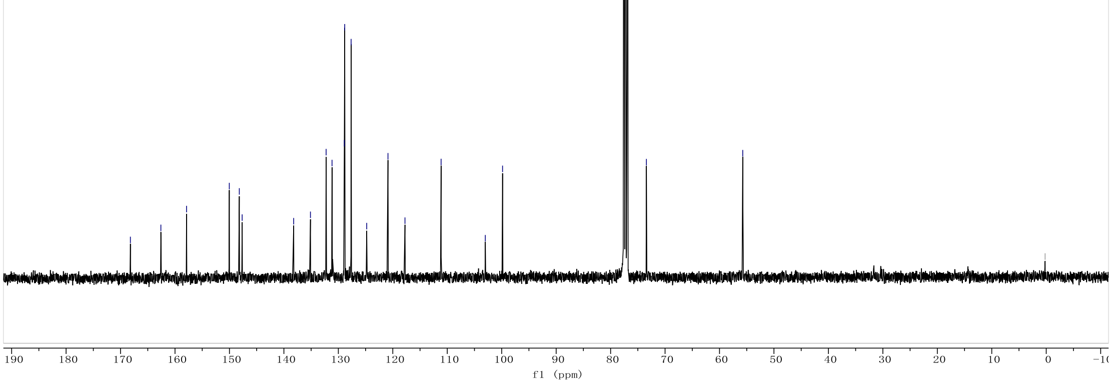

^{1}H NMR (600 MHz, Chloroform-*d*)

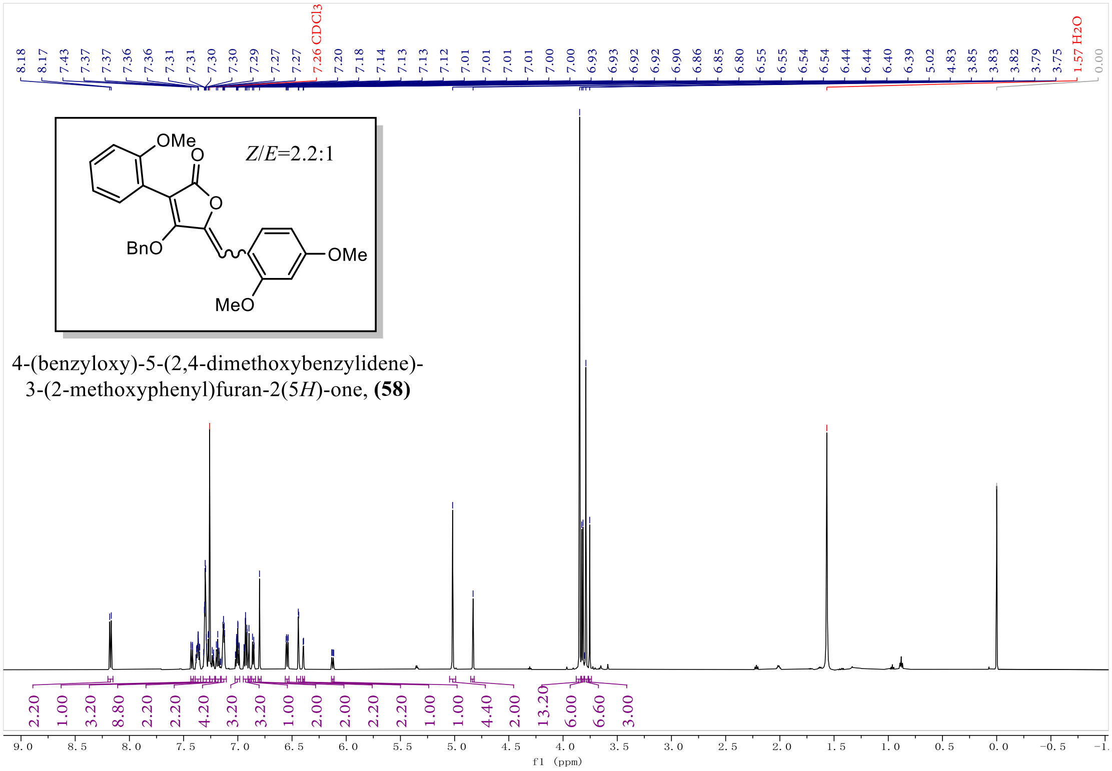

^{13}C NMR (101 MHz, Chloroform-*d*)

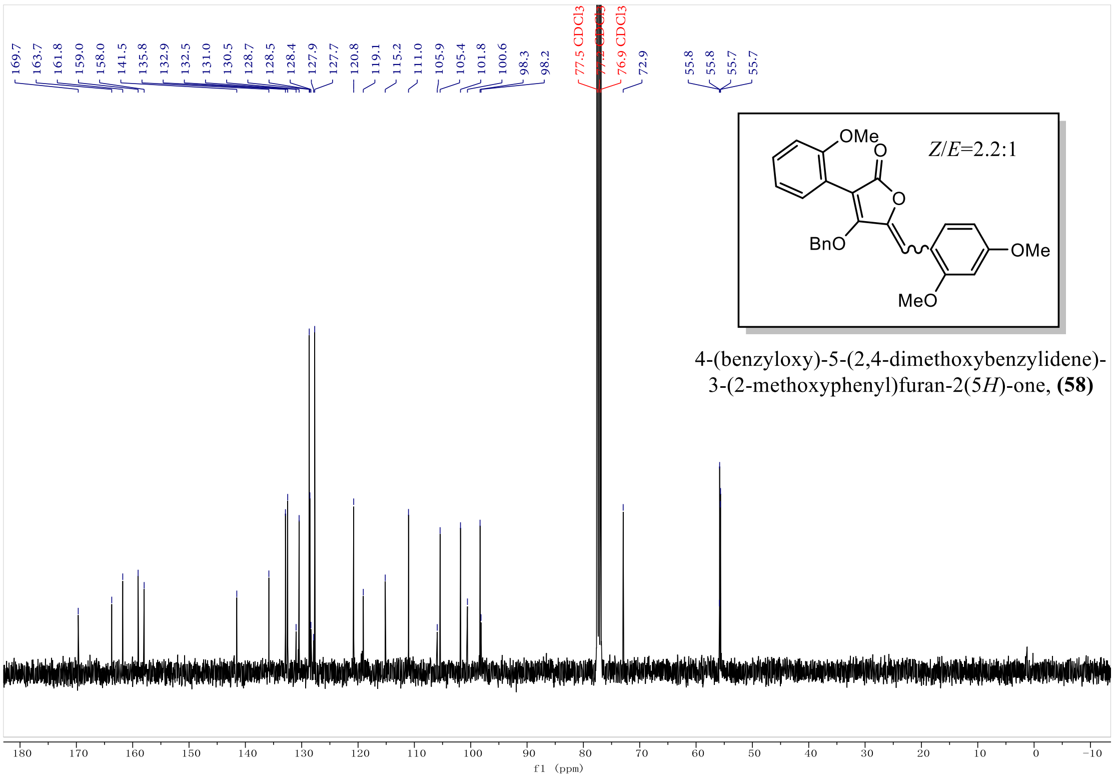

^{1}H NMR (600 MHz, Chloroform-*d*)

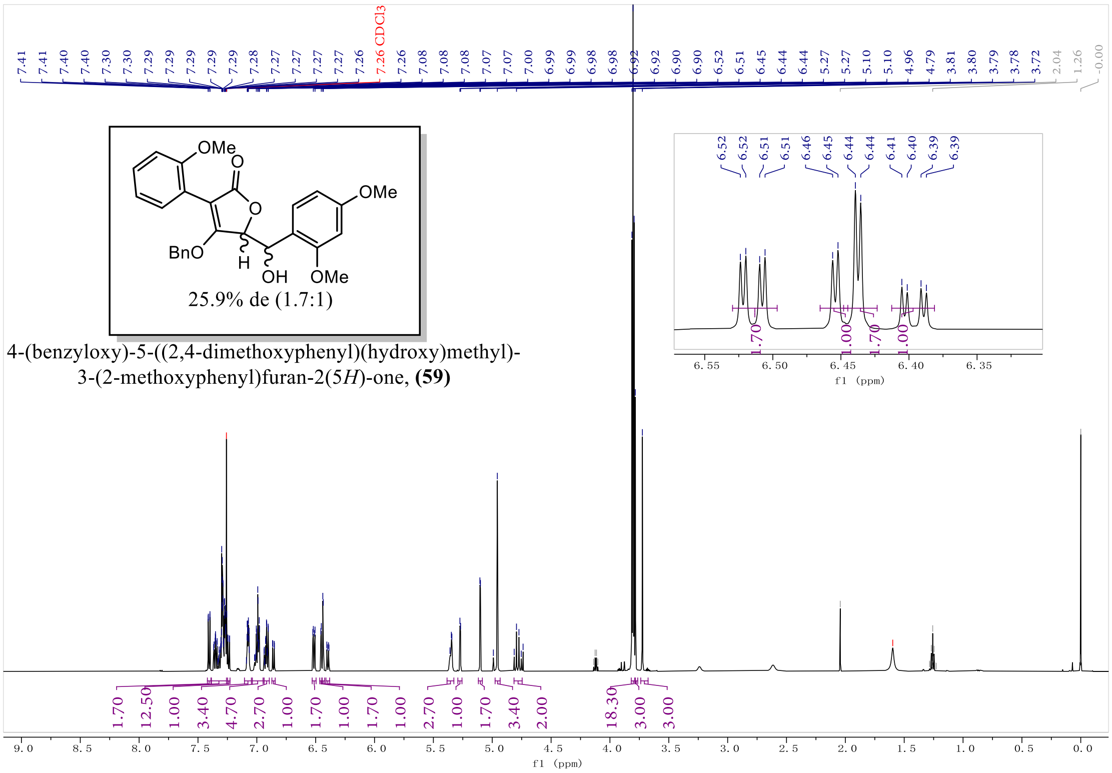

4-(benzyloxy)-5-((2,4-dimethoxyphenyl)(hydroxy)methyl)-3-(2-methoxyphenyl)furan-2(5*H*)-one, **(59)**

^{13}C NMR (101 MHz, Chloroform-*d*)

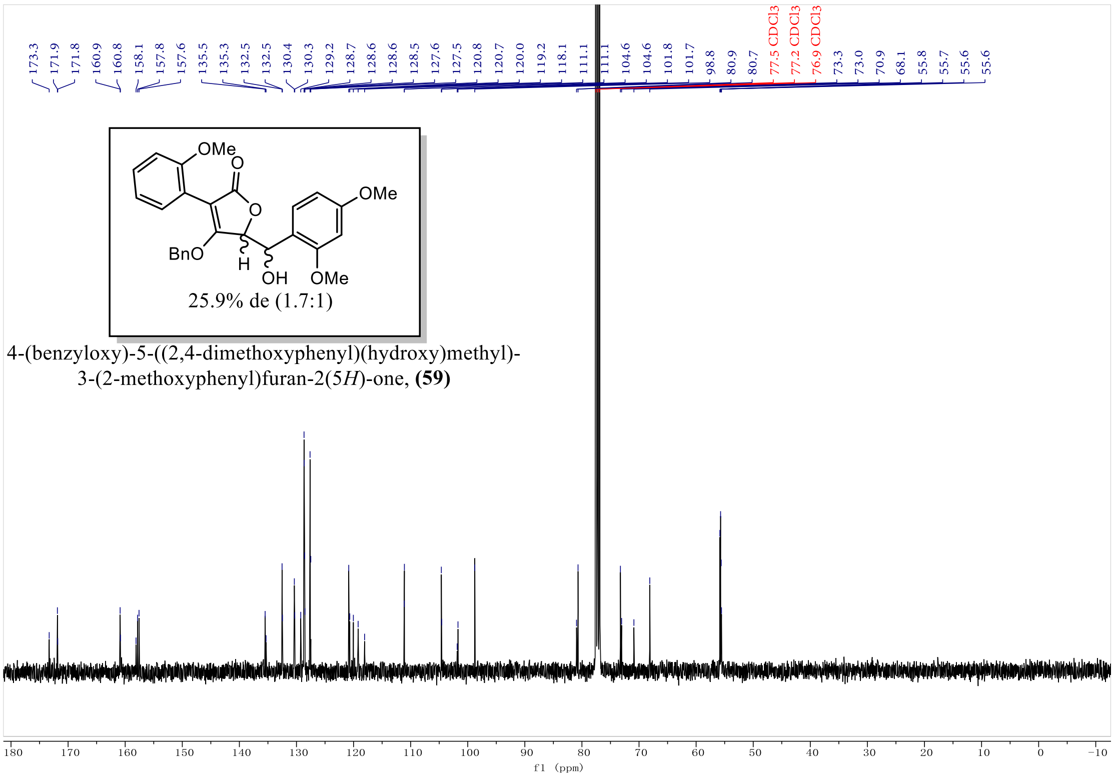

4-(benzyloxy)-5-((2,4-dimethoxyphenyl)(hydroxy)methyl)-3-(2-methoxyphenyl)furan-2(5*H*)-one, **(59)**

^{1}H NMR (600 MHz, Chloroform-*d*)

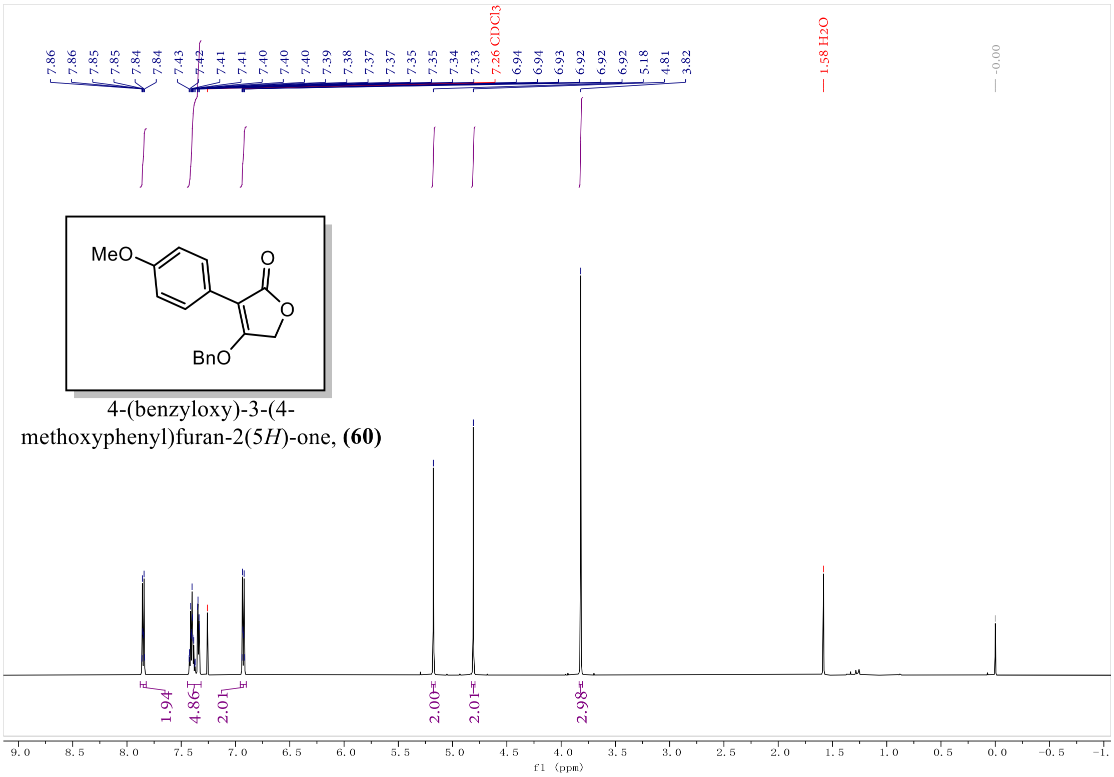

^{13}C NMR (151 MHz, Chloroform-*d*)

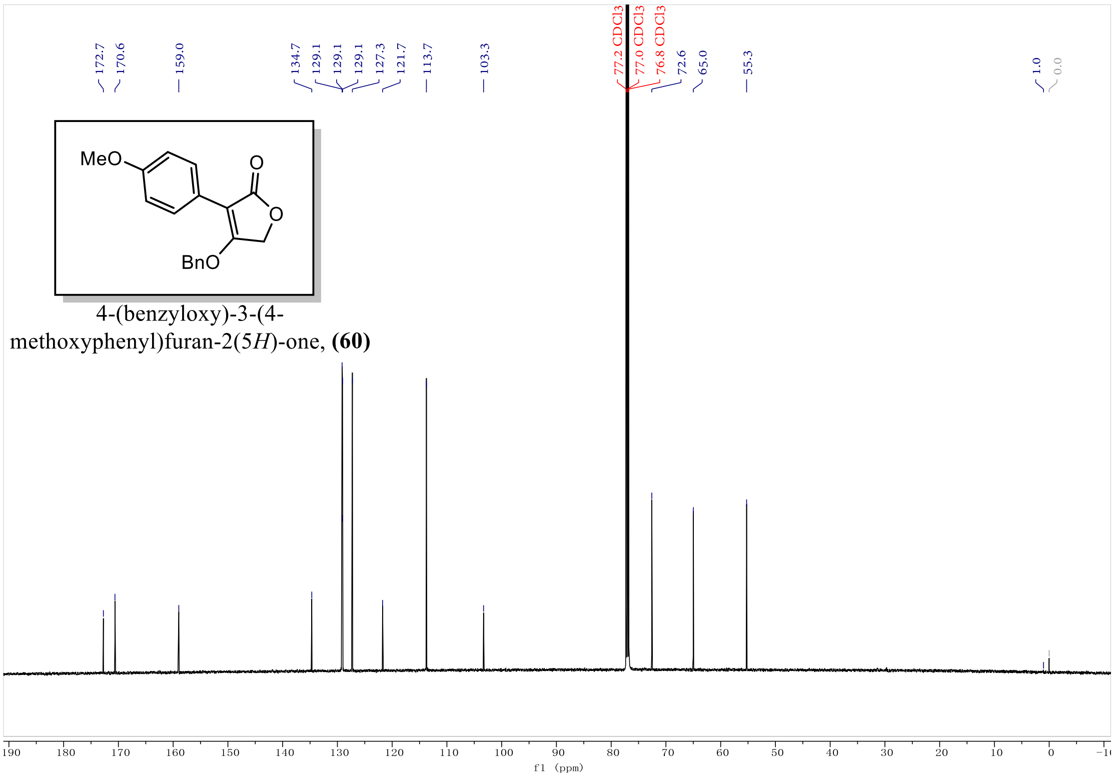

^{1}H NMR (600 MHz, Chloroform-*d*)

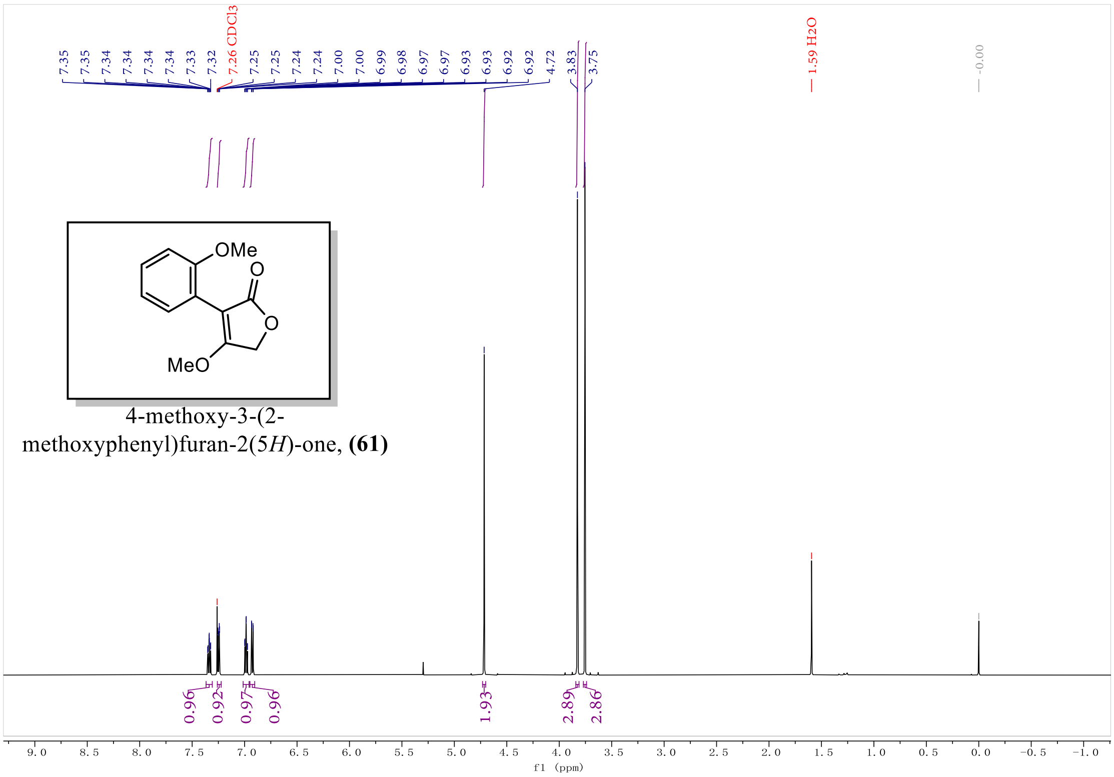

4-methoxy-3-(2-methoxyphenyl)furan-2(5*H*)-one, **(61)**

^{13}C NMR (151 MHz, Chloroform-*d*)

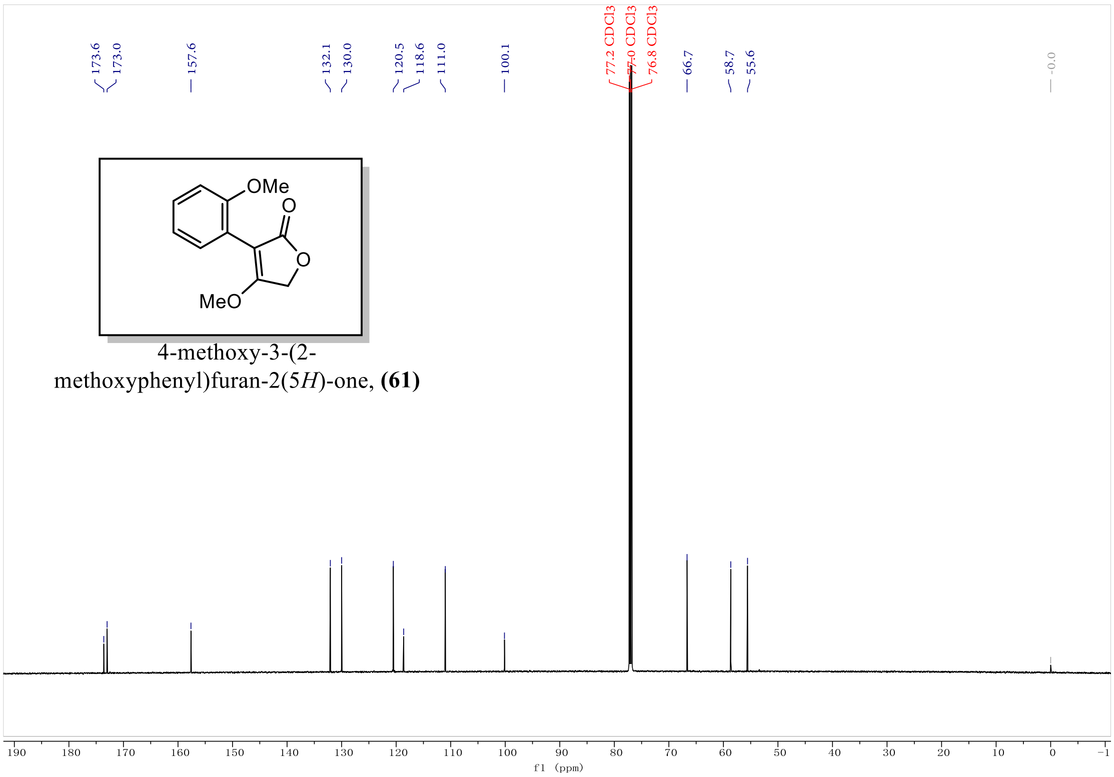

4-methoxy-3-(2-methoxyphenyl)furan-2(5*H*)-one, **(61)**

^{1}H NMR (400 MHz, DMSO-d_6)

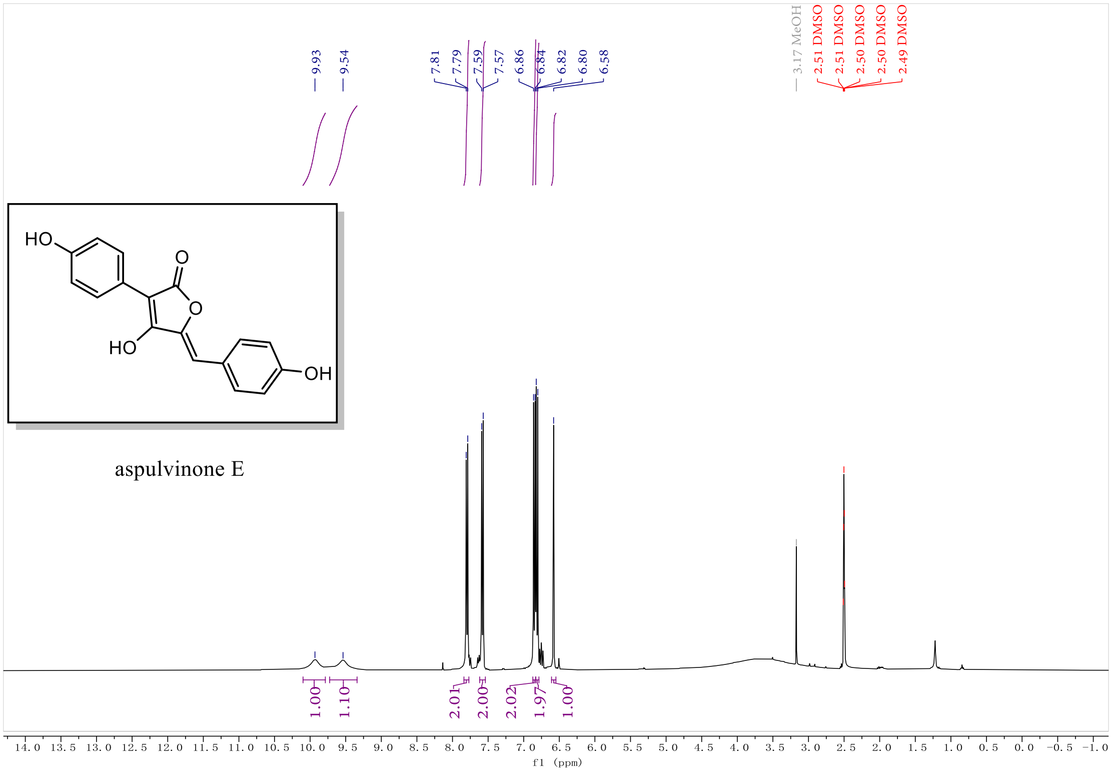

^{13}C NMR (101 MHz, DMSO-d_6)

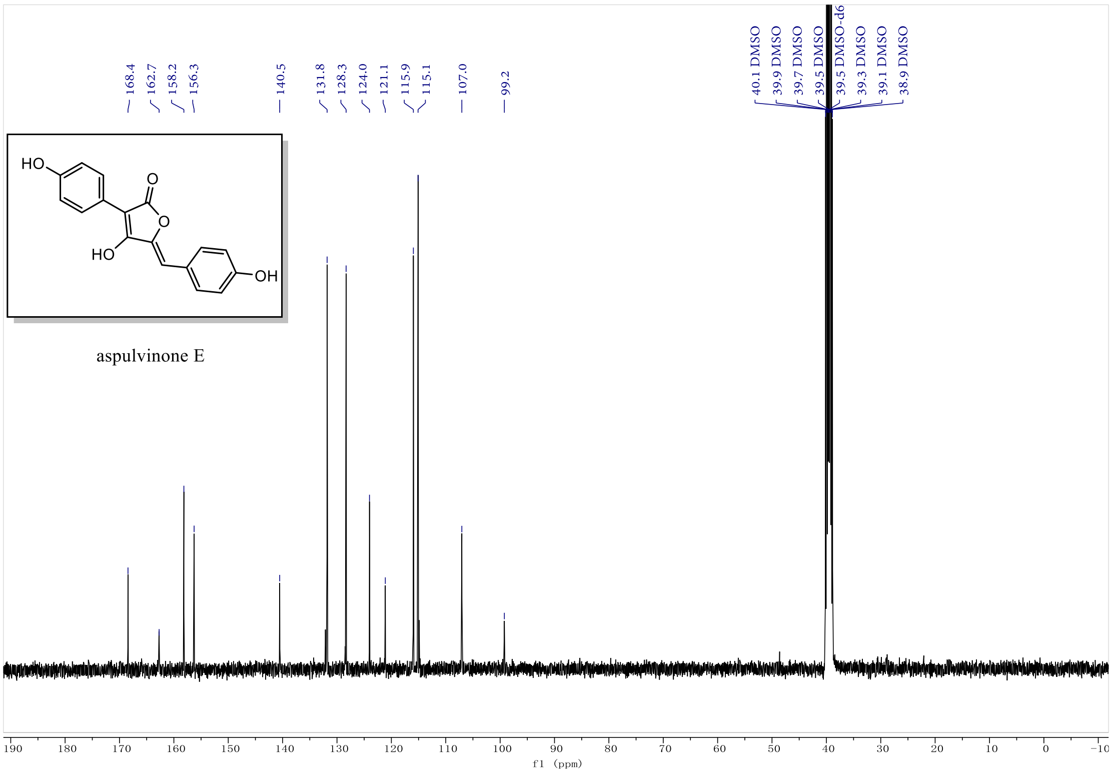

^{1}H NMR (400 MHz, DMSO-d_6)

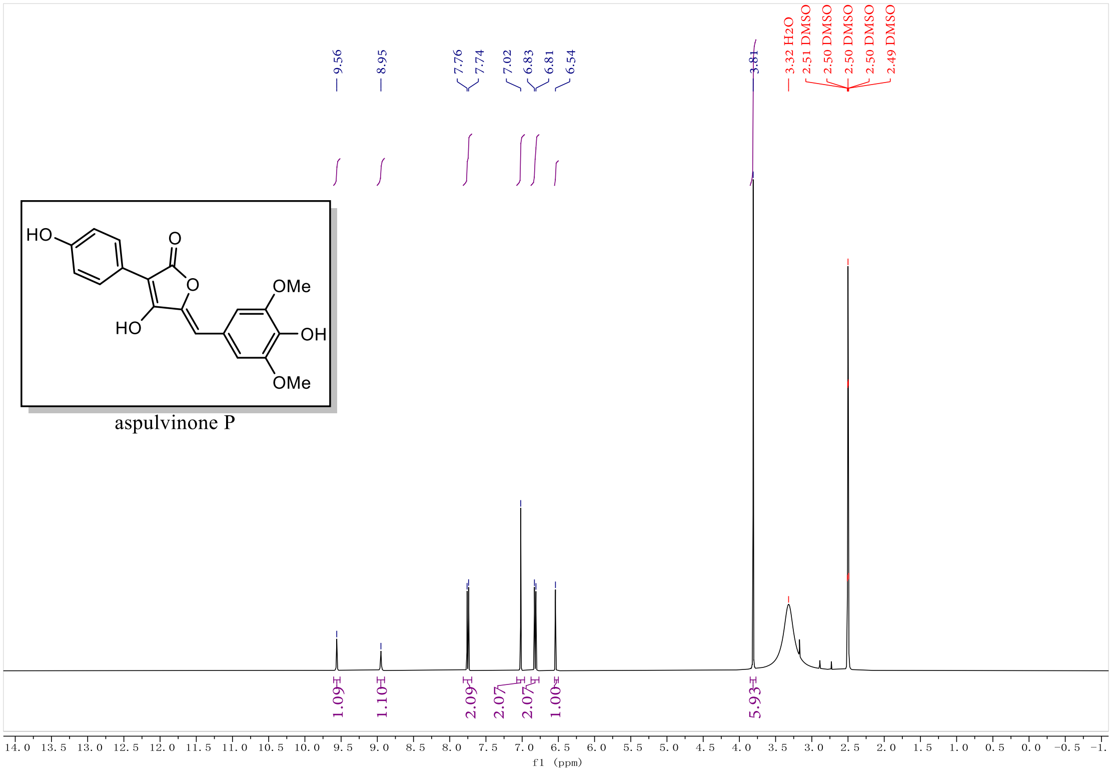

^{13}C NMR (101 MHz, DMSO-d_6)

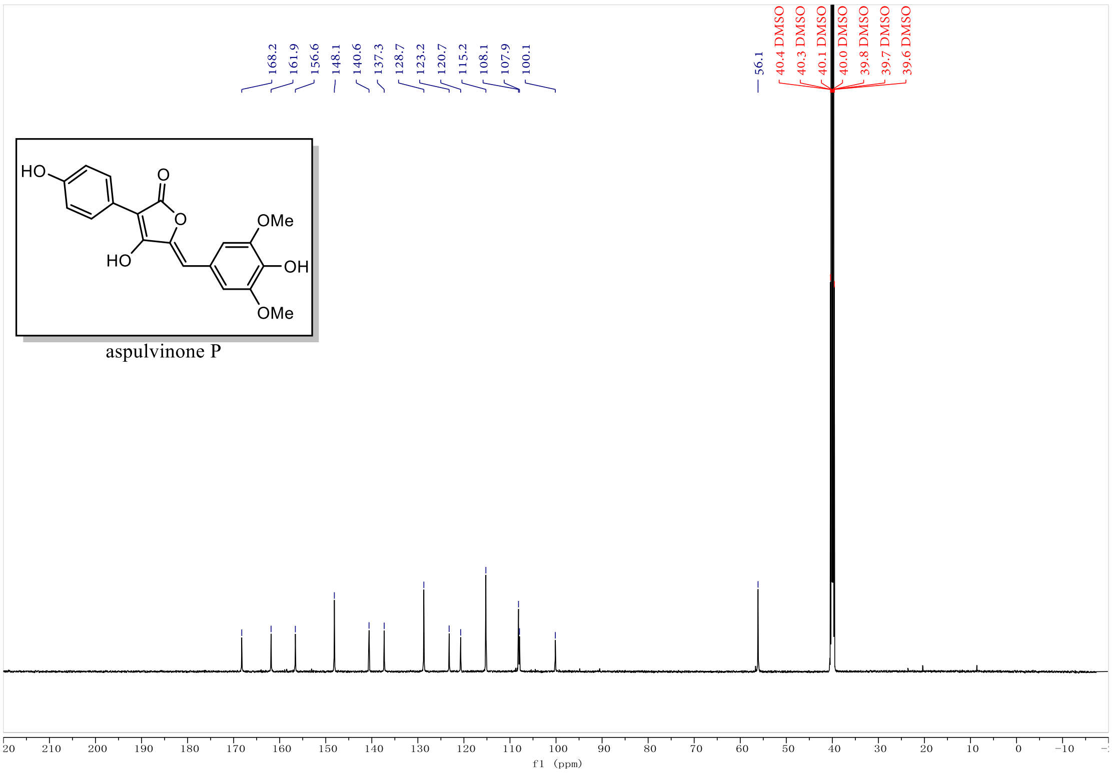

^{1}H NMR (600 MHz, DMSO-d_6)

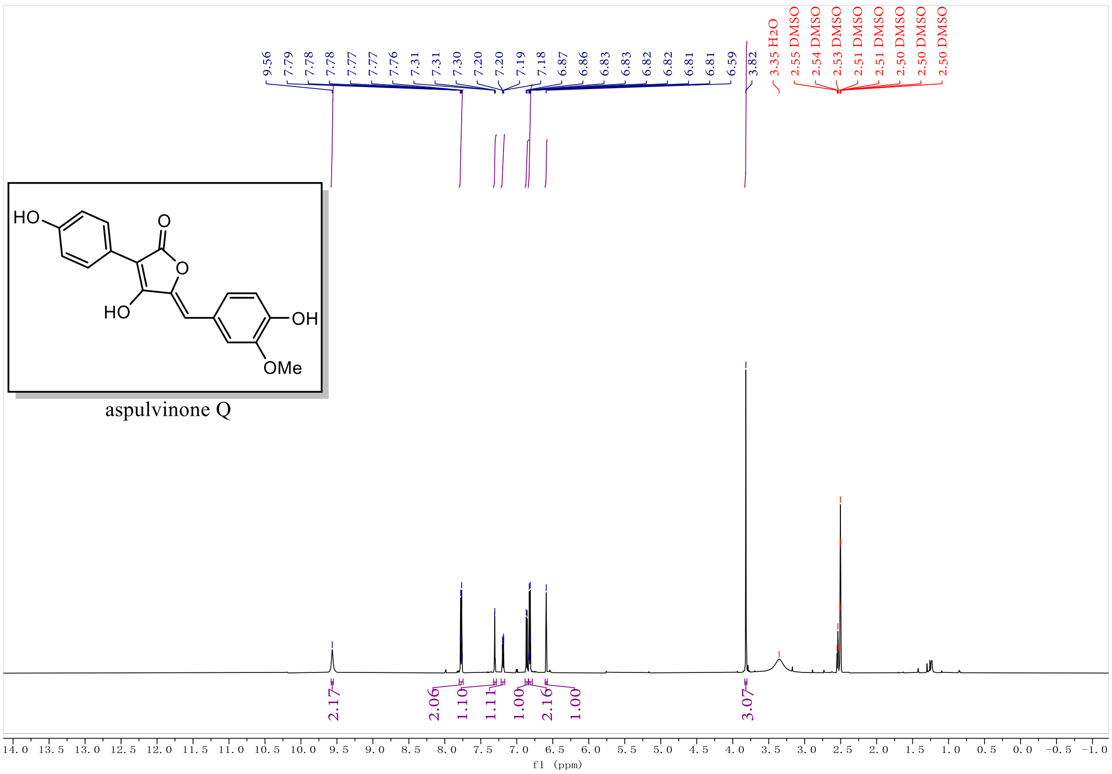

^{13}C NMR (101 MHz, DMSO-d_6)

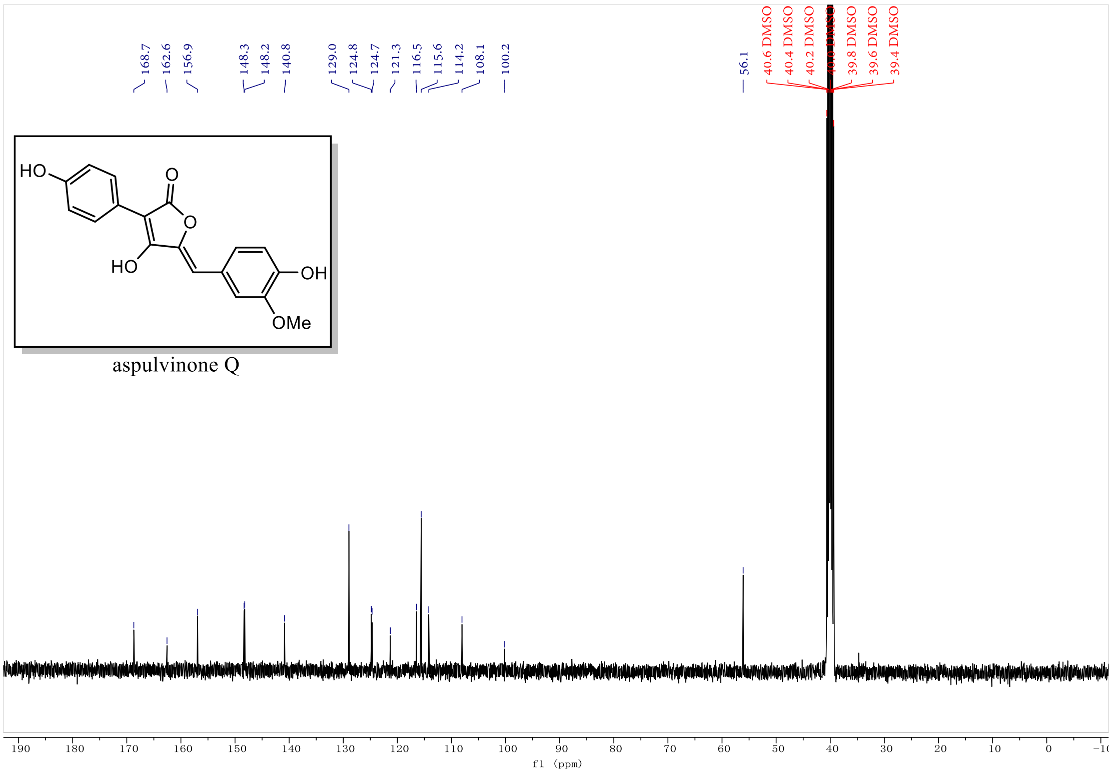

^{1}H NMR (400 MHz, DMSO-d_6)

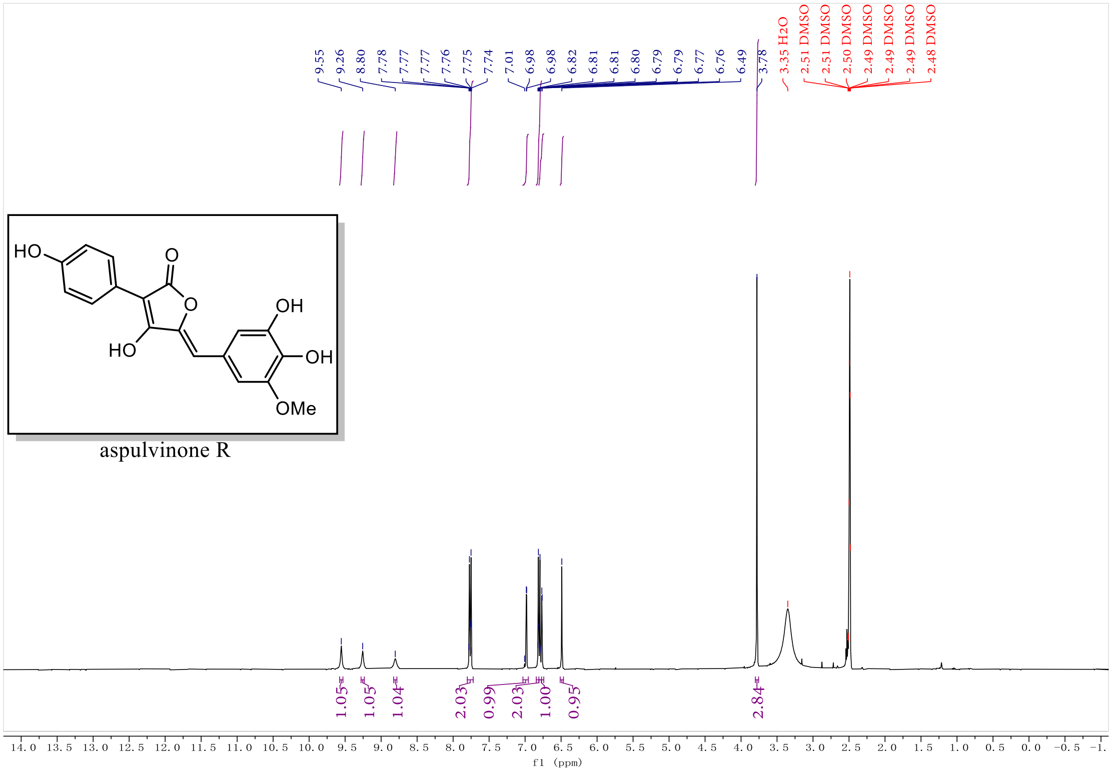

^{13}C NMR (151 MHz, DMSO-d_6)

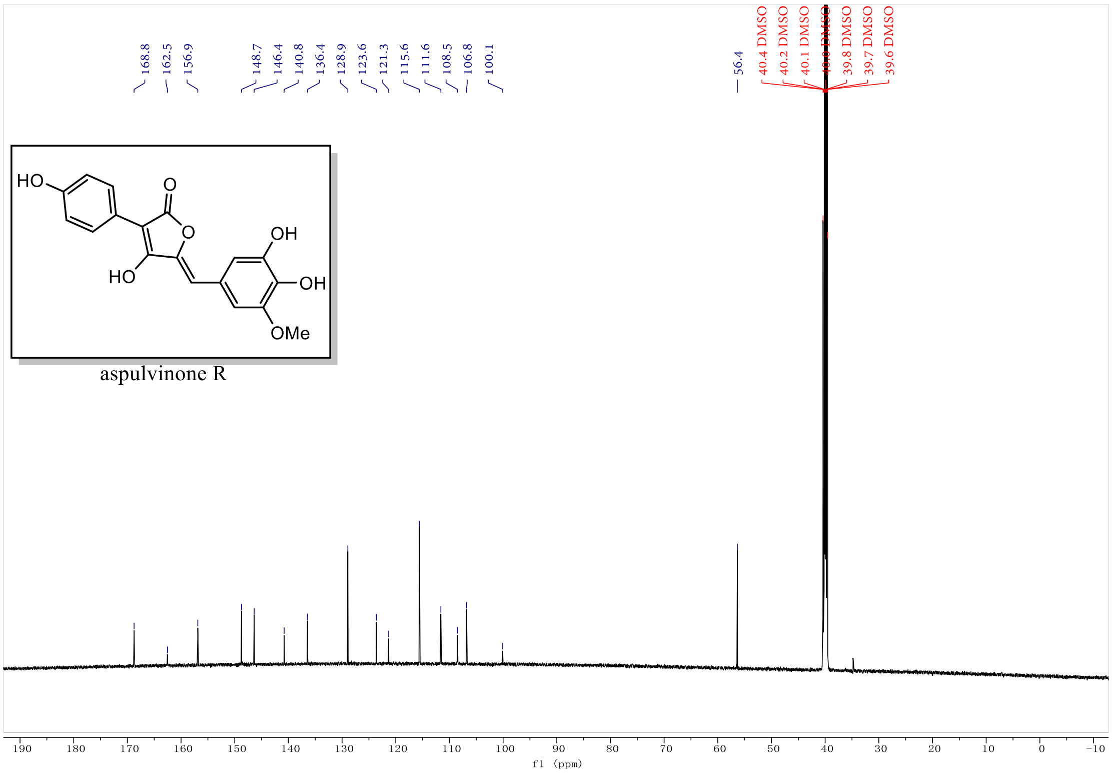

^{1}H NMR (400 MHz, DMSO-d_6)

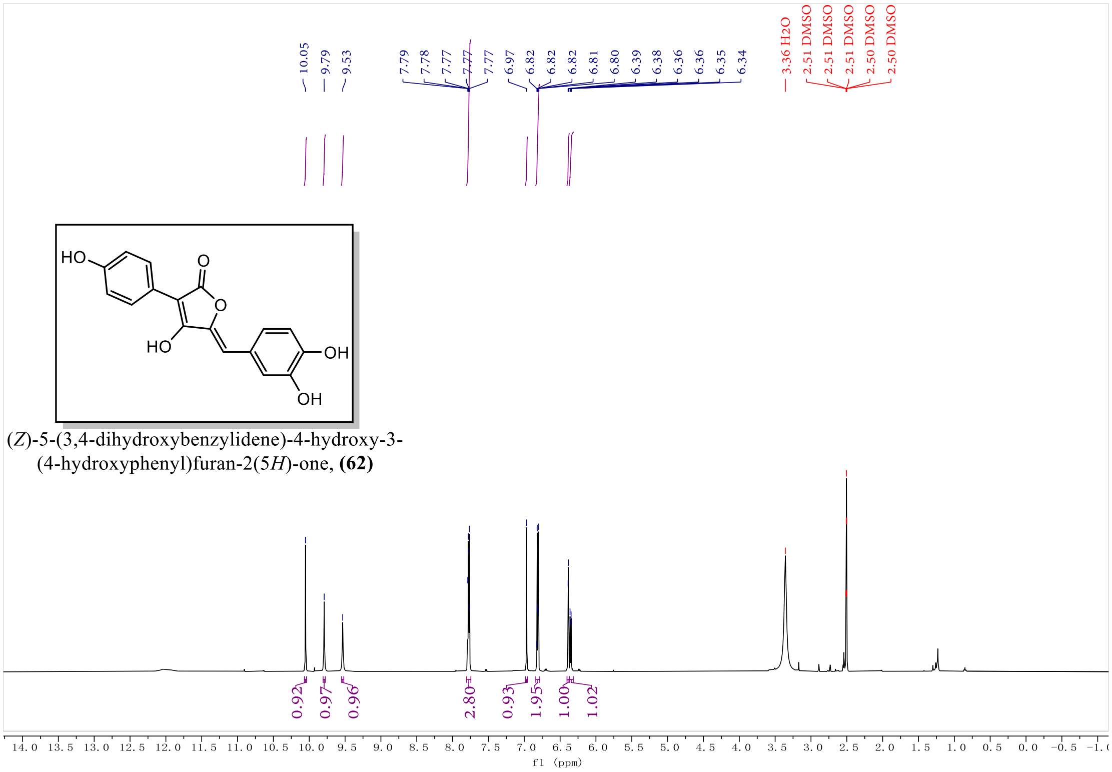

^{13}C NMR (101 MHz, DMSO-d_6)

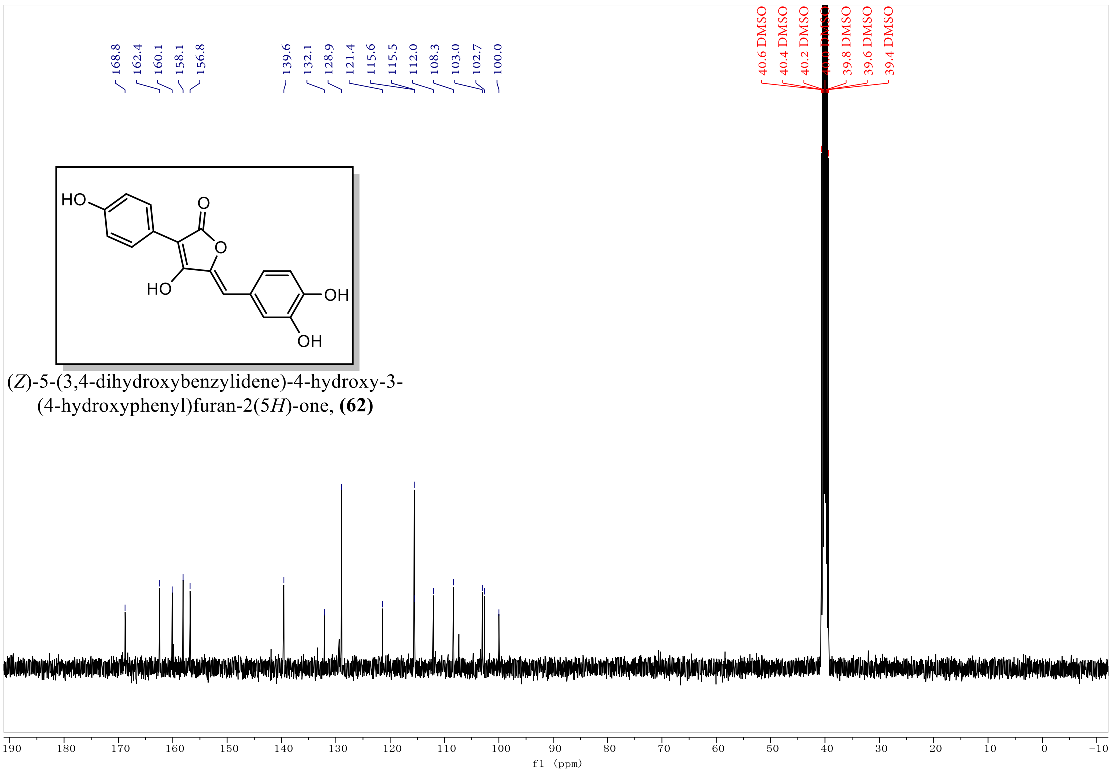

^{1}H NMR (600 MHz, Methanol-d_4)

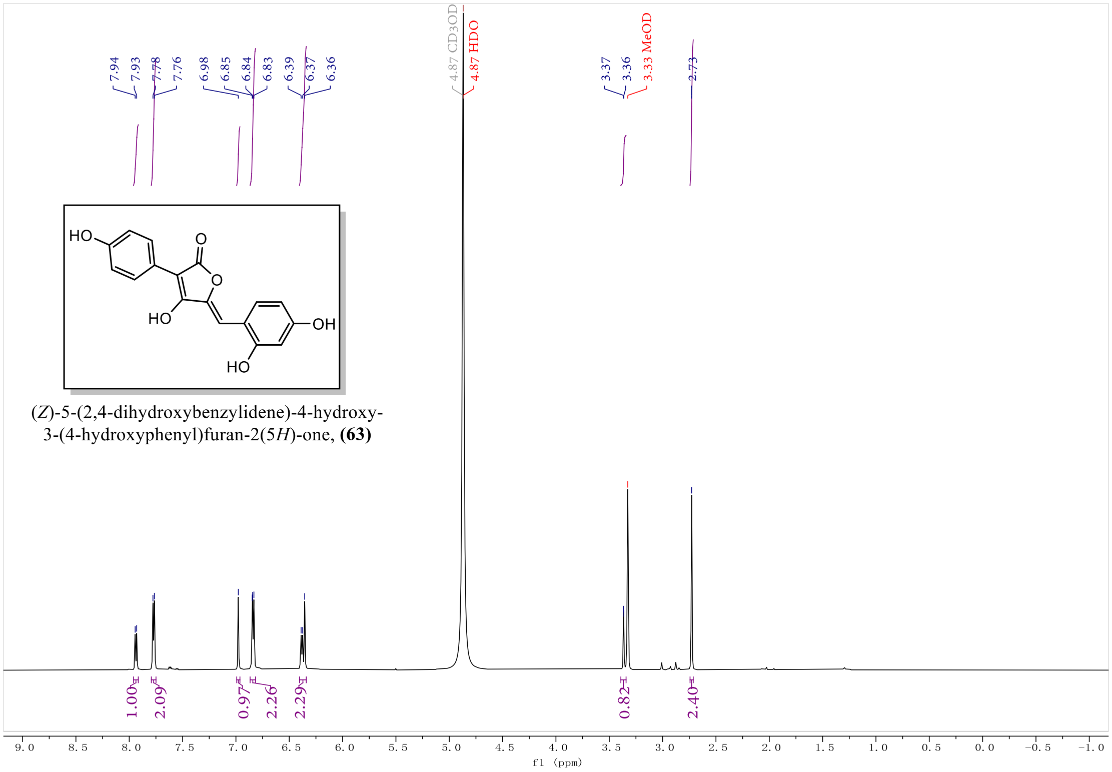

(*Z*)-5-(2,4-dihydroxybenzylidene)-4-hydroxy-3-(4-hydroxyphenyl)furan-2(5*H*)-one, **(63)**

^{13}C NMR (101 MHz, Methanol-d_4)

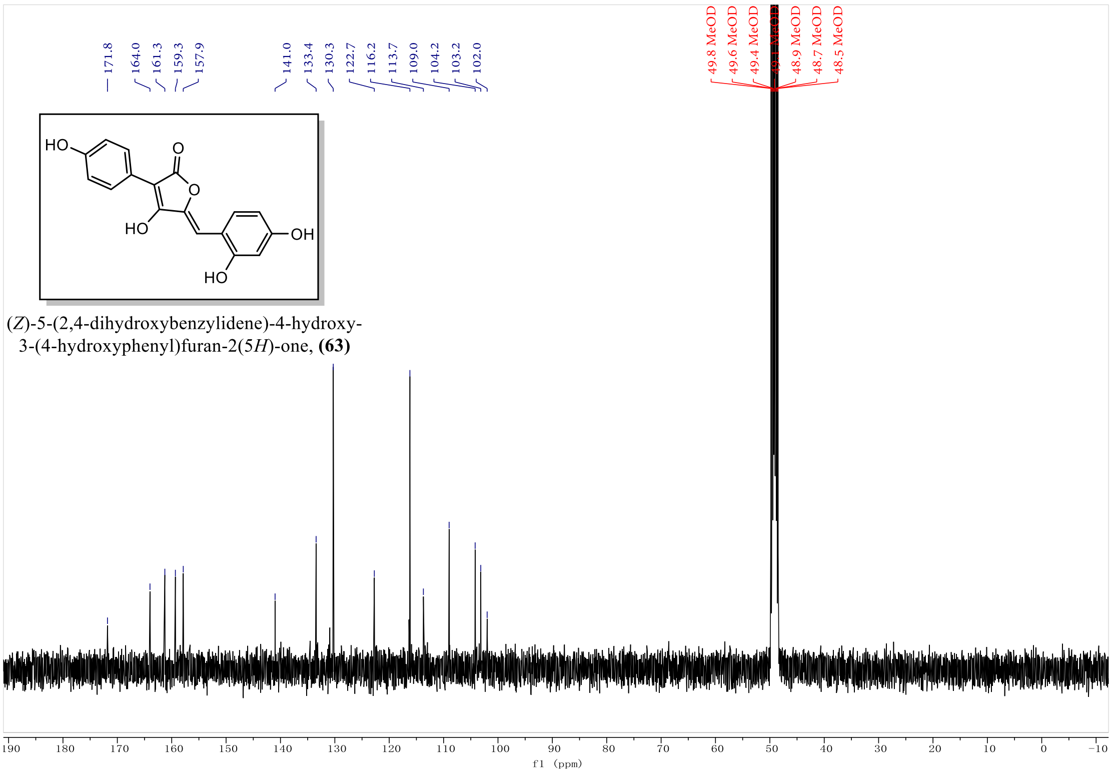

(*Z*)-5-(2,4-dihydroxybenzylidene)-4-hydroxy-3-(4-hydroxyphenyl)furan-2(5*H*)-one, **(63)**

^{1}H NMR (400 MHz, Acetone-d_6)

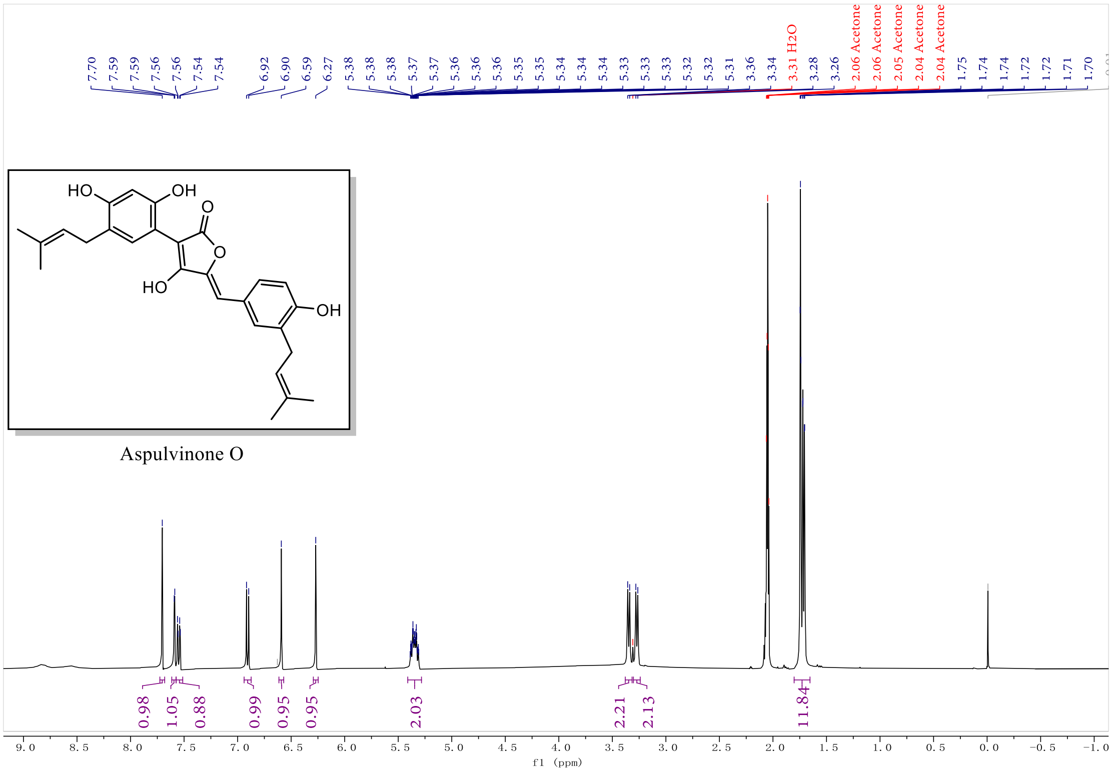

^{13}C NMR (151 MHz, Acetone-d_6)

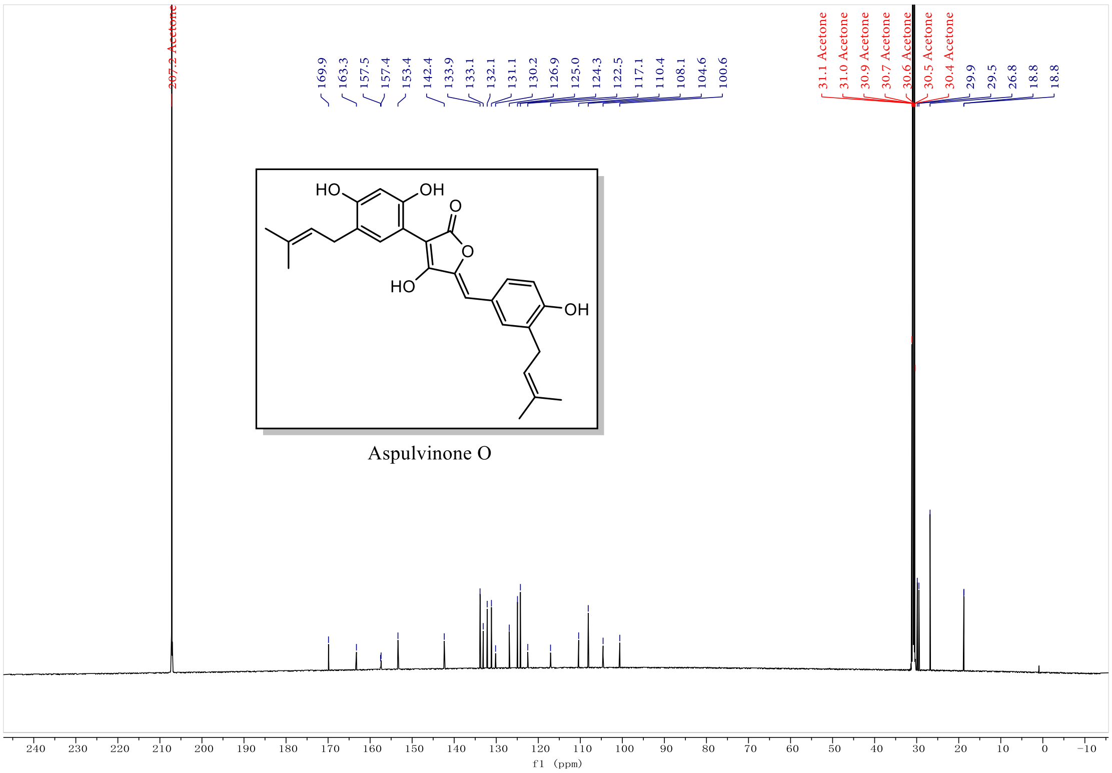

^{1}H NMR (400 MHz, DMSO-d_6)

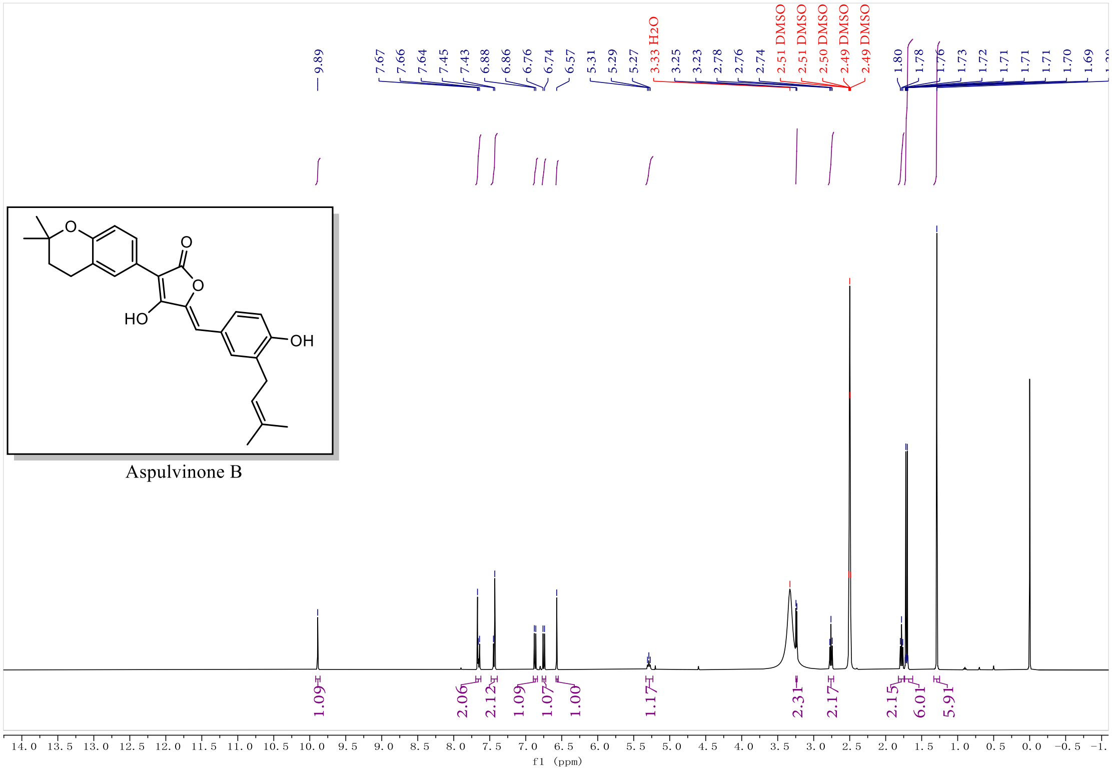

^{13}C NMR (101 MHz, DMSO-d_6)

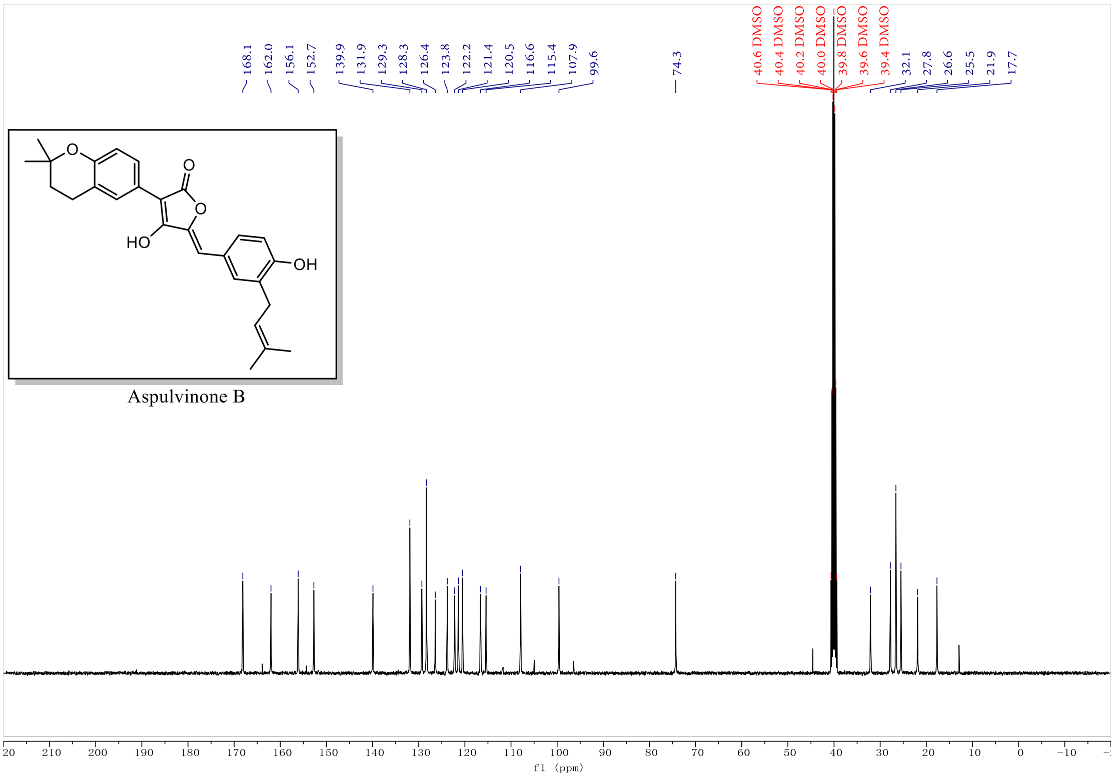

^{1}H NMR (400 MHz, Chloroform-*d*)

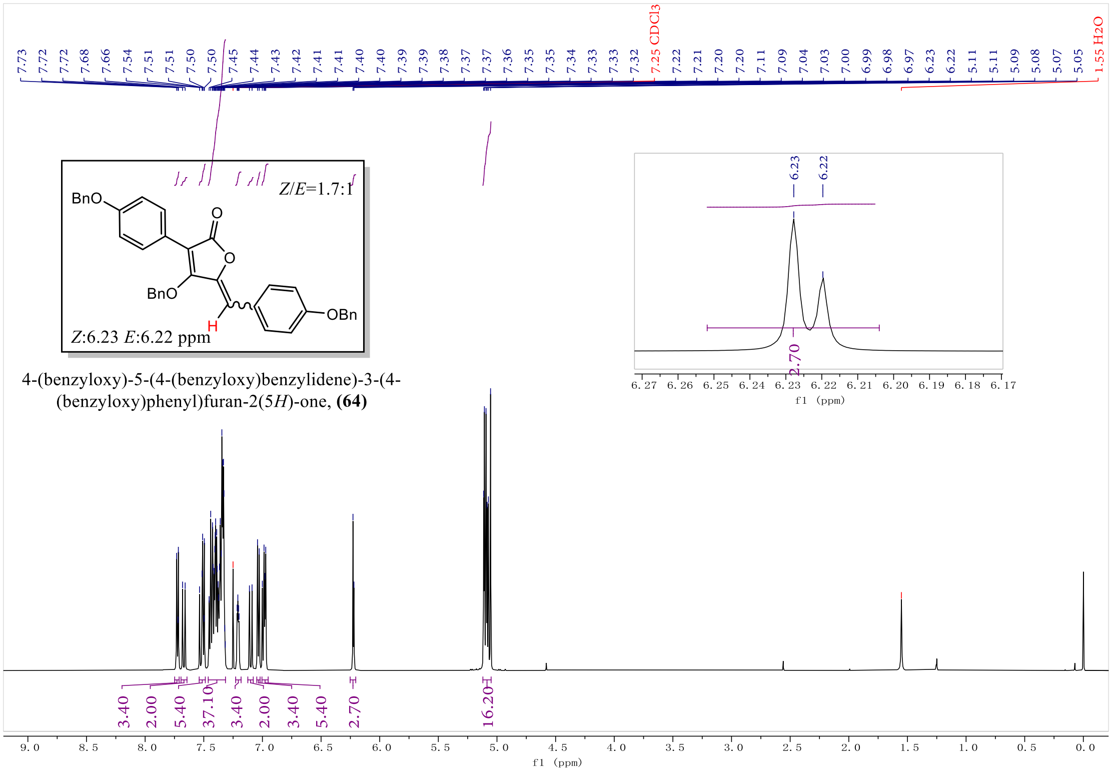

4-(benzyloxy)-5-(4-(benzyloxy)benzylidene)-3-(4-(benzyloxy)phenyl)furan-2(5*H*)-one, **(64)**

^{1}H NMR (400 MHz, Chloroform-*d*)

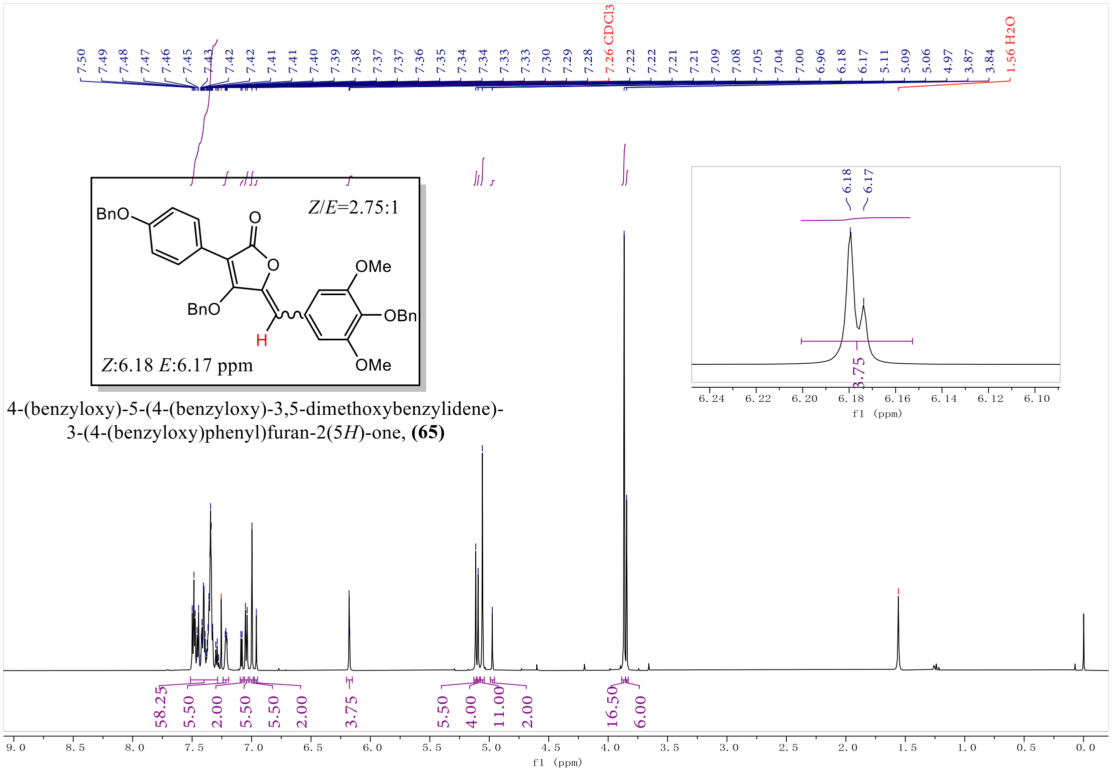

^{1}H NMR (600 MHz, DMSO-d_6)

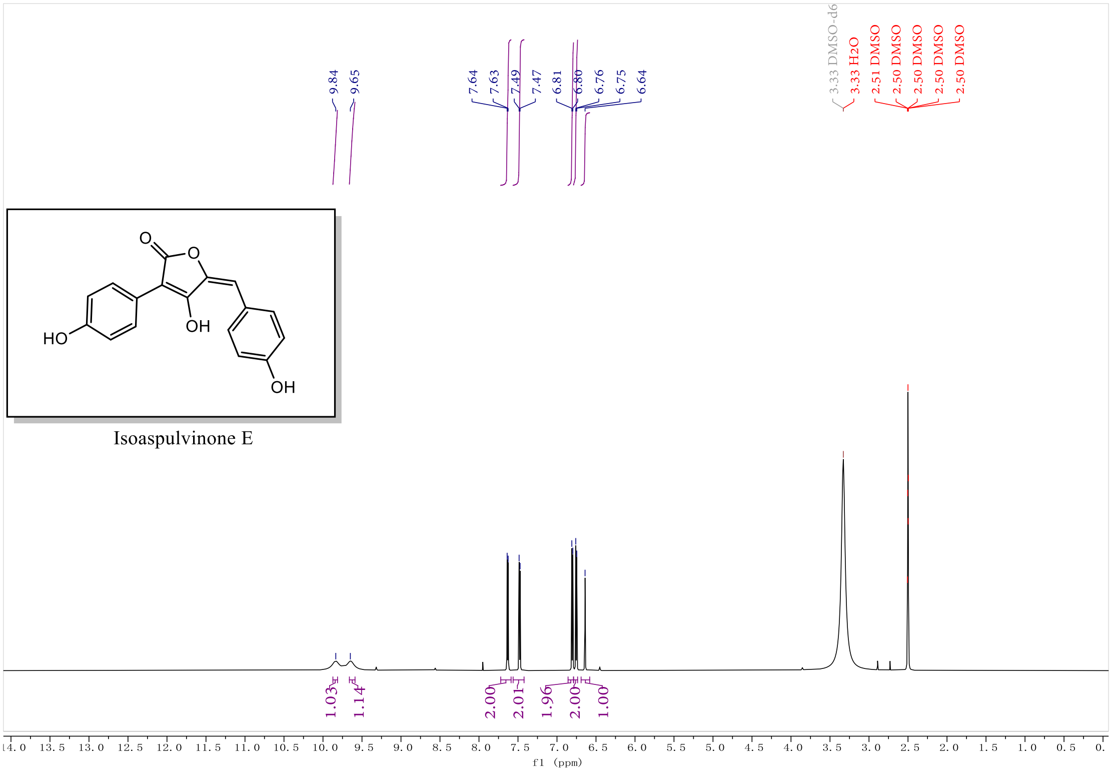

^{13}C NMR (101 MHz, DMSO-d_6)

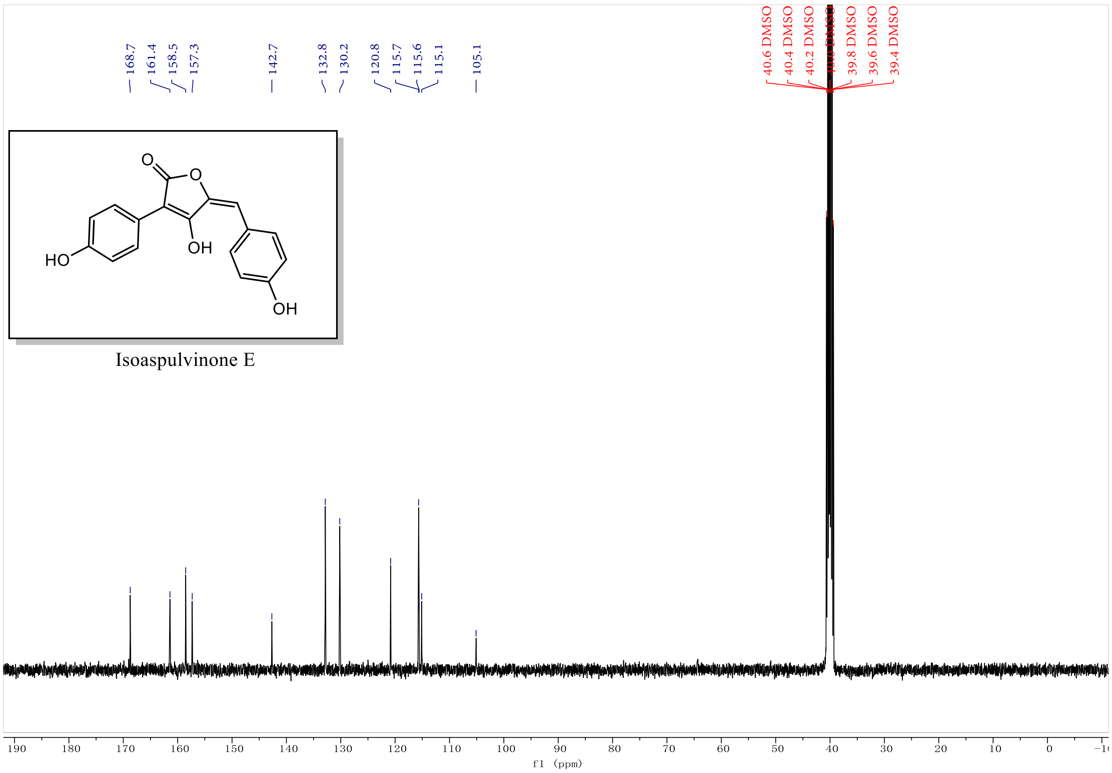

^{1}H NMR (400 MHz, DMSO-d_6)

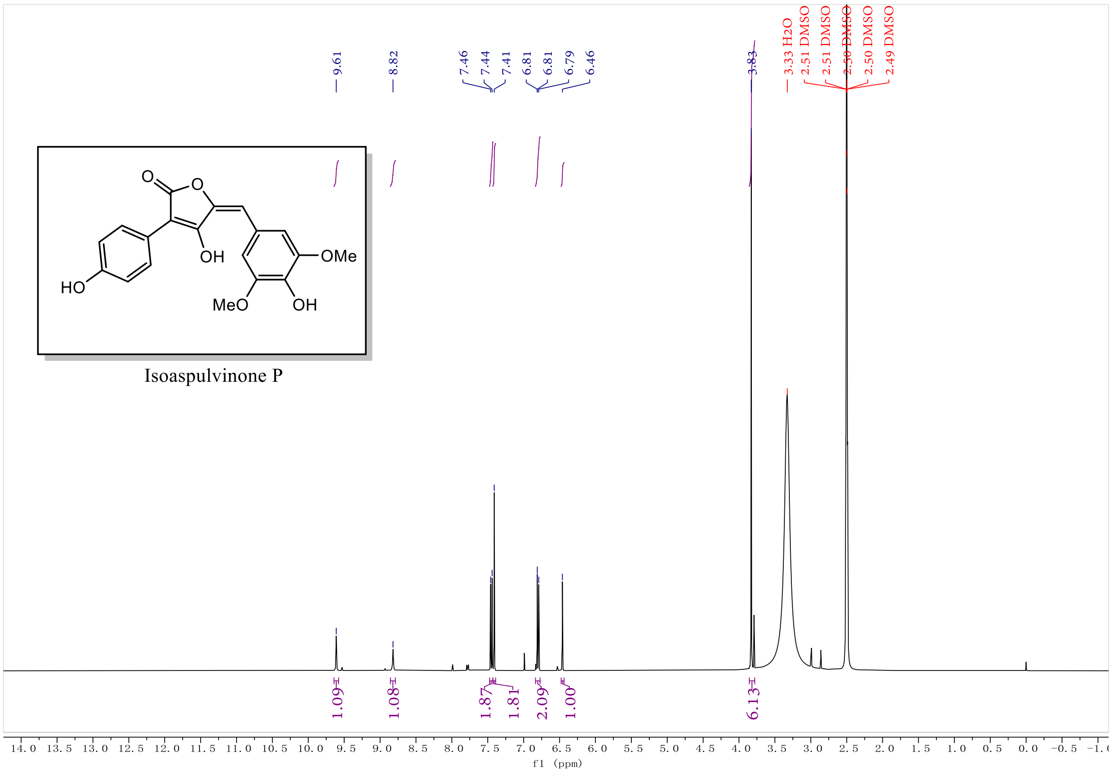

^{13}C NMR (151 MHz, DMSO-d_6)

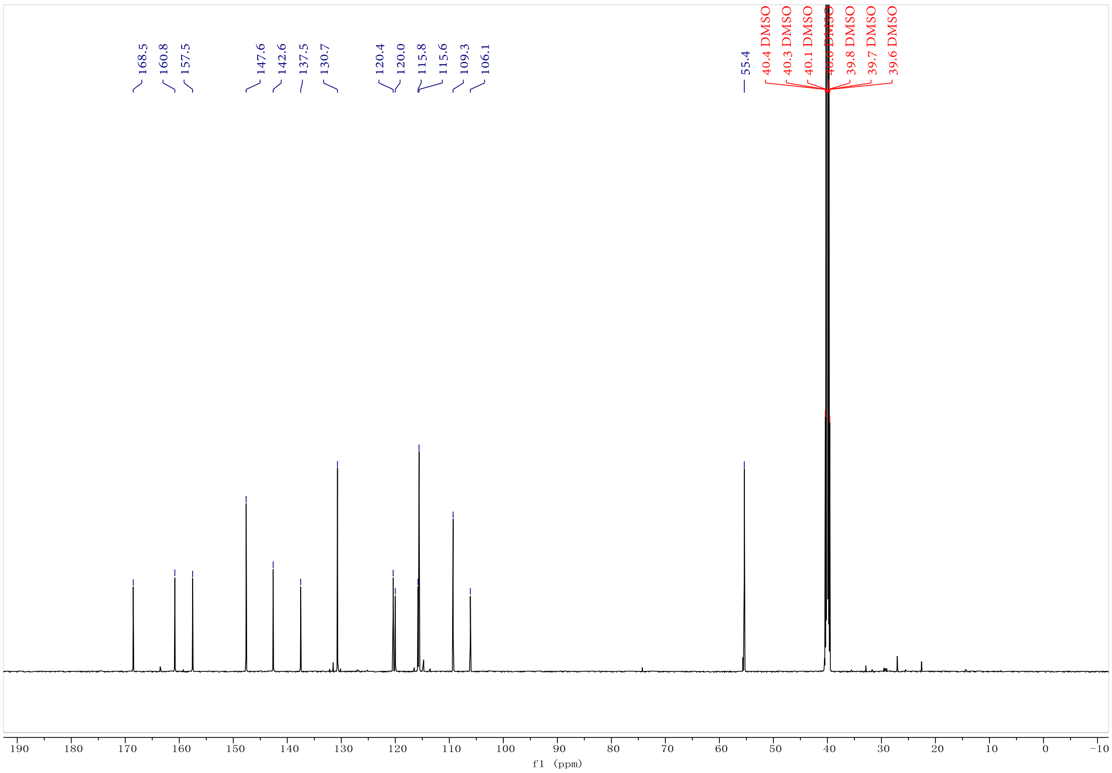

10 X-ray crystallographic analysis

10.1 X-ray structure of 23

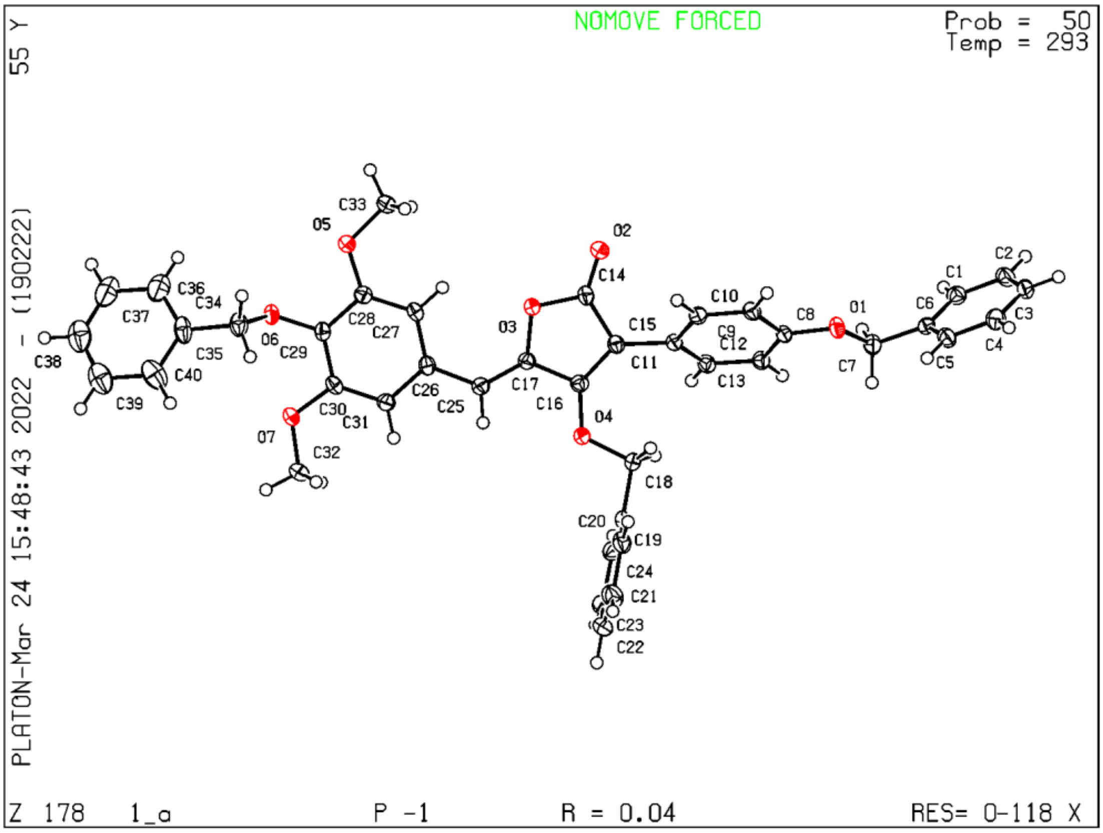

Table 1. Crystal data and structure refinement for 1_a.

Identification code	1_a	
Empirical formula	C40 H34 O7	
Formula weight	626.67	
Temperature	293(2) K	
Wavelength	1.54178 Å	
Crystal system	Triclinic	
Space group	P-1	
Unit cell dimensions	a = 9.5414(2) Å	α= 70.624(2)°.
	b = 13.4295(3) Å	β= 84.081(2)°.
	c = 13.5307(3) Å	γ = 82.783(2)°.
Volume	1619.12(6) $Å^3$	
Z	2	
Density (calculated)	1.285 Mg/m^3	
Absorption coefficient	0.710 mm^{-1}	

F(000)	660
Crystal size	0.200 x 0.200 x 0.200 mm^3
Theta range for data collection	3.470 to 66.590°.
Index ranges	-11<=h<=11, -15<=k<=15, -16<=l<=16
Reflections collected	36514
Independent reflections	5700 [R(int) = 0.0614]
Completeness to theta = 66.590°	99.6 %
Absorption correction	Semi-empirical from equivalents
Refinement method	Full-matrix least-squares on F^2
Data / restraints / parameters	5700 / 0 / 426
Goodness-of-fit on F^2	1.035
Final R indices [I>2sigma(I)]	R1 = 0.0432, wR2 = 0.1147
R indices (all data)	R1 = 0.0467, wR2 = 0.1173
Extinction coefficient	n/a
Largest diff. peak and hole	0.925 and -0.308 e.$Å^{-3}$

10.2 X-ray structure of 31

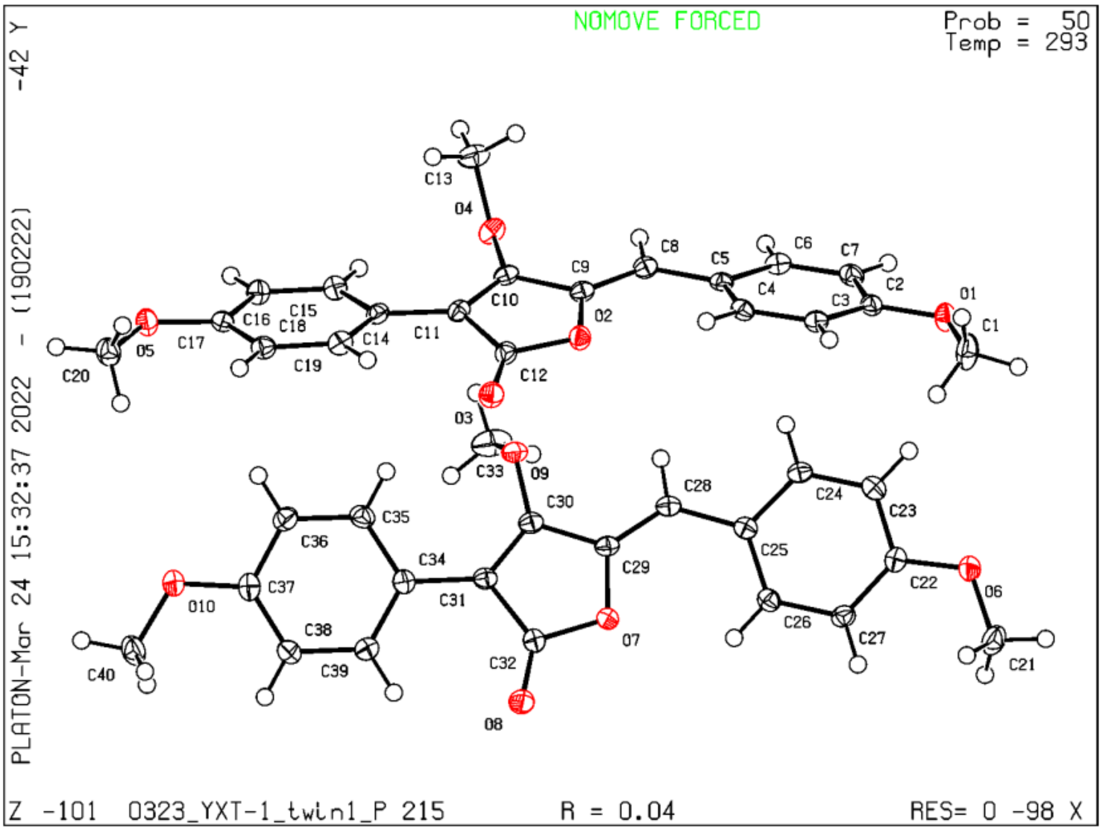

Table 1. Crystal data and structure refinement for 0323_YXT-1_twin1_hklf5.

Identification code	0323_YXT-1_twin1_hklf5

Empirical formula	C20 H18 O5	
Formula weight	338.34	
Temperature	293(2) K	
Wavelength	1.54178 Å	
Crystal system	Monoclinic	
Space group	$P2_1$	
Unit cell dimensions	a = 11.9556(2) Å	α= 90°.
	b = 7.1740(2) Å	β= 94.087(2)°.
	c = 18.9601(4) Å	γ = 90°.
Volume	1622.06(6) $Å^3$	
Z	4	
Density (calculated)	1.385 Mg/m^3	
Absorption coefficient	0.821 mm^{-1}	
F(000)	712	
Crystal size	0.200 x 0.200 x 0.200 mm^3	
Theta range for data collection	3.706 to 73.910°.	
Index ranges	-14<=h<=14, -8<=k<=8, -23<=l<=23	
Reflections collected	10514	
Independent reflections	10514 [R(int) = ?]	
Completeness to theta = 67.679°	100.0 %	
Absorption correction	Semi-empirical from equivalents	
Refinement method	Full-matrix least-squares on F^2	
Data / restraints / parameters	10514 / 1 / 458	
Goodness-of-fit on F^2	0.867	
Final R indices [I>2sigma(I)]	R1 = 0.0415, wR2 = 0.1172	
R indices (all data)	R1 = 0.0420, wR2 = 0.1179	
Absolute structure parameter	0.45(10)	
Extinction coefficient	n/a	
Largest diff. peak and hole	0.180 and -0.266 e.$Å^{-3}$	

10.3 X-ray structure of 58

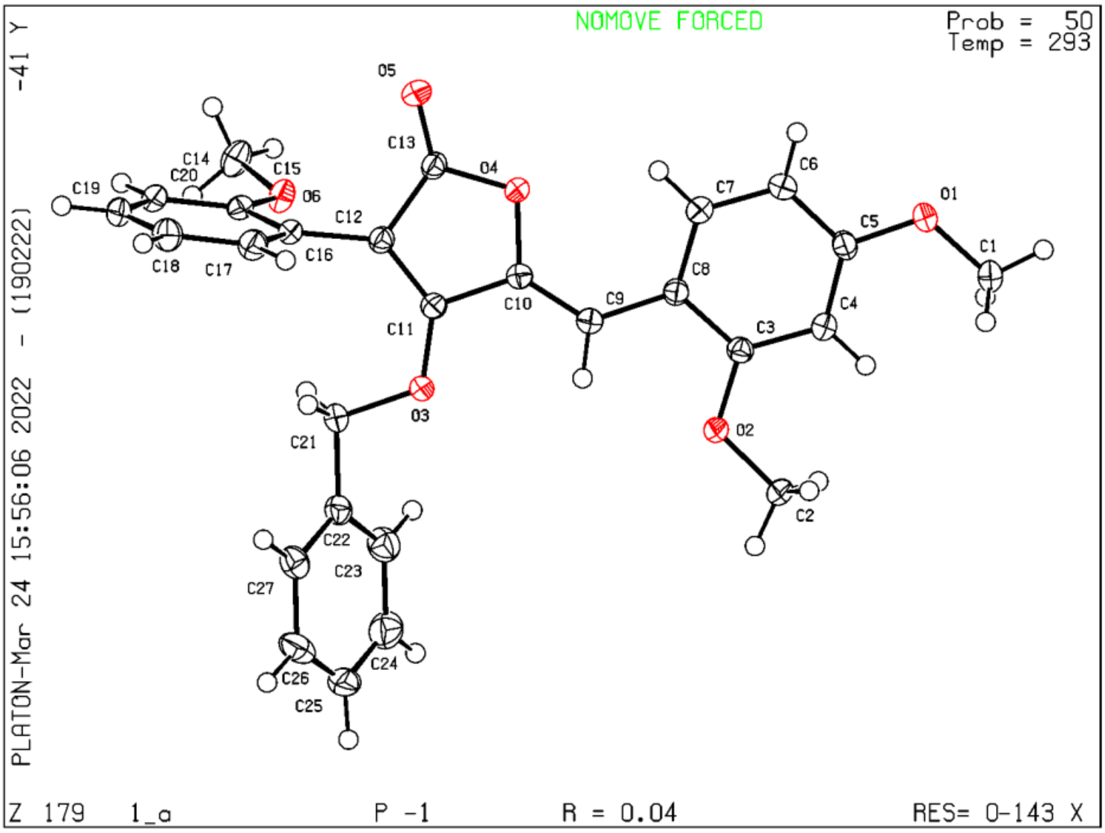

Table 1. Crystal data and structure refinement for 1_a.

Identification code	1_a	
Empirical formula	C27 H24 O6	
Formula weight	444.46	
Temperature	293(2) K	
Wavelength	1.54178 Å	
Crystal system	Triclinic	
Space group	P-1	
Unit cell dimensions	a = 10.4836(2) Å	α= 115.718(2)°.
	b = 10.8282(2) Å	β= 91.618(2)°.
	c = 11.0718(3) Å	γ = 101.288(2)°.
Volume	1101.30(5) $Å^3$	
Z	2	
Density (calculated)	1.340 Mg/m^3	
Absorption coefficient	0.775 mm^{-1}	
F(000)	468	
Crystal size	0.200 x 0.200 x 0.200 mm^3	
Theta range for data collection	4.336 to 73.901°.	

Index ranges	-13<=h<=12, -13<=k<=13, -13<=l<=13
Reflections collected	23562
Independent reflections	4375 [R(int) = 0.0292]
Completeness to theta = 67.679°	99.6 %
Absorption correction	Semi-empirical from equivalents
Refinement method	Full-matrix least-squares on F^2
Data / restraints / parameters	4375 / 0 / 301
Goodness-of-fit on F^2	1.058
Final R indices [I>2sigma(I)]	R1 = 0.0391, wR2 = 0.1021
R indices (all data)	R1 = 0.0398, wR2 = 0.1027
Extinction coefficient	n/a
Largest diff. peak and hole	0.230 and -0.334 e.Å^{-3}

10.4 X-ray structure of aspulvinone O

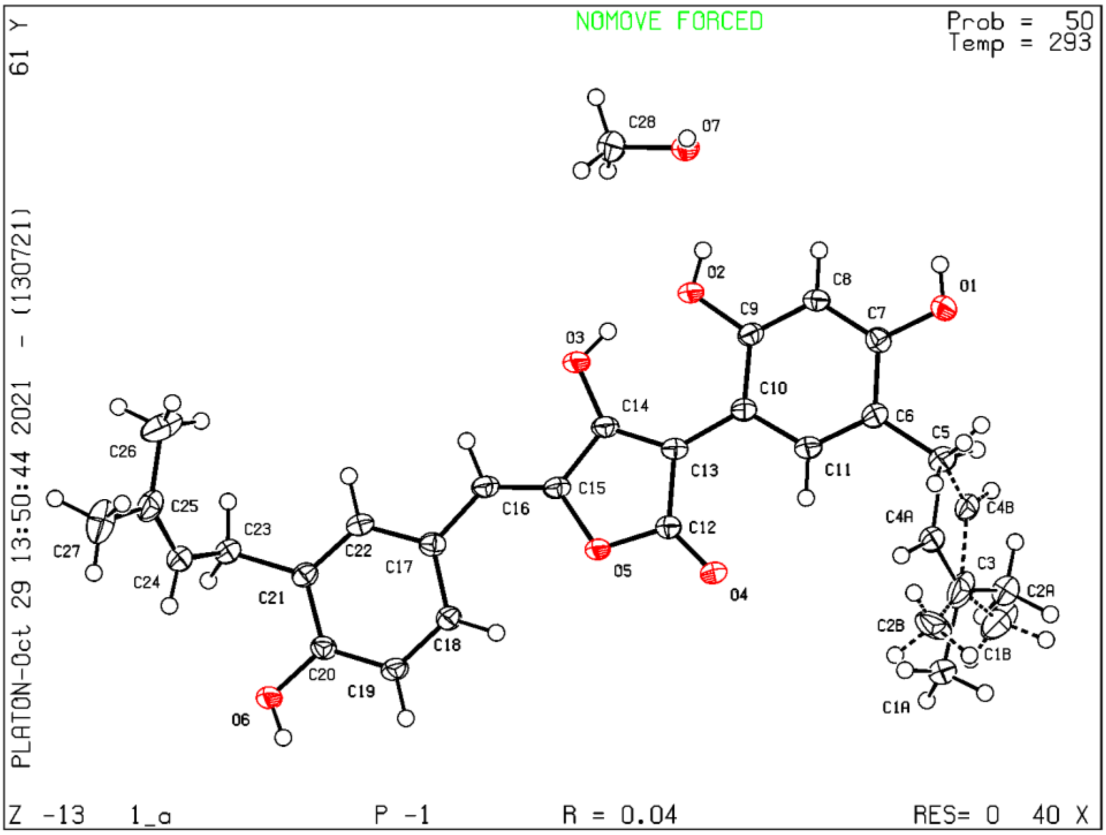

Table 1. Crystal data and structure refinement for 1_a.

Identification code	1_a
Empirical formula	C28 H32 O7
Formula weight	480.53

Temperature	293(2) K	
Wavelength	1.54178 Å	
Crystal system	Triclinic	
Space group	P-1	
Unit cell dimensions	a = 8.3502(2) Å	α= 105.807(2)°.
	b = 11.2071(2) Å	β= 99.945(2)°.
	c = 13.7253(3) Å	γ = 92.702(2)°.
Volume	1211.27(5) $Å^3$	
Z	2	
Density (calculated)	1.318 Mg/m^3	
Absorption coefficient	0.770 mm^{-1}	
F(000)	512	
Crystal size	0.400 x 0.200 x 0.050 mm^3	
Theta range for data collection	3.411 to 73.897°.	
Index ranges	-10<=h<=10, -13<=k<=13, -17<=l<=17	
Reflections collected	25818	
Independent reflections	4775 [R(int) = 0.0273]	
Completeness to theta = 67.679°	99.5 %	
Absorption correction	Semi-empirical from equivalents	
Refinement method	Full-matrix least-squares on F^2	
Data / restraints / parameters	4775 / 108 / 351	
Goodness-of-fit on F^2	1.051	
Final R indices [I>2sigma(I)]	R1 = 0.0398, wR2 = 0.1084	
R indices (all data)	R1 = 0.0419, wR2 = 0.1104	
Extinction coefficient	n/a	
Largest diff. peak and hole	0.275 and -0.238 e.$Å^{-3}$	

11 Reference

1. Kumar, J.; Gupta, A.; Bhadra, S., Pd-II-Catalyzed methoxylation of C(sp(3))-H bonds adjacent to benzoxazoles and benzothiazoles. *Organic & Biomolecular Chemistry* **2019,** *17*, 3314-3318.
2. Wang, Z. Y.; Hu, X. N.; Li, Y. Y.; Mou, X. D.; Wang, C. C.; Chen, X. L.; Tan, Y. Y.; Wu, C. L.; Liu, H. M.; Xu, H., Discovery and SAR Research for Antivirus Activity of Novel Butenolide on Influenza A Virus H1N1 In Vitro and In Vivo. *Acs Omega* **2019,** *4*, 13265-13269.
3. Bellina, F.; Marchetti, C.; Rossi, R., An Economical Access to 3,4-Diaryl-2(5H)-furanones and 4-Aryl-6-methyl-2(2H)-pyranones by Pd-Catalyzed Suzuki-Type Arylation of 3-Aryl-4-tosyloxy-2(5H)-furanones and 6-Methyl-4-tosyloxy-2(2H)-pyranones, Respectively. *Eur. J. Org. Chem.* **2009,** *2009*, 4685-4690.
4. Xiao, Z. P.; Ouyang, H.; Wang, X. D.; Lv, P. C.; Huang, Z. J.; Yu, S. R.; Yi, T. F.; Yang, Y. L.; Zhu, H. L., 4-Alkoxy-3-arylfuran-2(5H)-ones as inhibitors of tyrosyl-tRNA synthetase: Synthesis, molecular docking and antibacterial evaluation. *Bioorgan. Med. Chem.* **2011,** *19*, 3884-3891.
5. Mallinger, A.; Le Gall, T.; Mioskowski, C., 3-Aryltetronic Acids: Efficient Preparation and Use as Precursors for Vulpinic Acids. *J. Org. Chem.* **2009,** *74*, 1124-1129.
6. Willis, C.; Bodio, E.; Bourdreux, Y.; Billaud, C.; Le Gall, T.; Mioskowski, C., Flexible synthesis of vulpinic acids from tetronic acid. *Tetrahedron Lett.* **2007,** *48*, 6421-6424.
7. Vece, V.; Ricci, J.; Poulain-Martini, S.; Nava, P.; Carissan, Y.; Humbel, S.; Dunach, E., In-III-Catalysed Tandem C-C and C-O Bond Formation between Phenols and Allylic Acetates. *Eur. J. Org. Chem.* **2010,** *2010*, 6239-6248.
8. Lee, B. K.; Yoon, J. H.; Yoon, S.; Cho, B. K., Induced Eye-detectable Blue Emission of Triazolyl Derivatives via Selective Photodecomposition of Chloroform under UV Irradiation at 365 nm. *B Korean Chem. Soc.* **2014,** *35*, 135-140.
9. Bernier, D.; Bruckner, R., Novel synthesis of naturally occurring pulvinones: A heck coupling, transesterification, and Dieckmann condensation strategy. *Synthesis* **2007**, *15*, 2249-2272.
10. Dow, M.; Marchetti, F.; Abrahams, K. A.; Vaz, L.; Besra, G. S.; Warriner, S.; Nelson, A., Modular Synthesis of Diverse Natural Product-Like Macrocycles: Discovery of Hits with Antimycobacterial Activity. *Chem-Eur. J.* **2017,** *23*, 7207-7211.
11. Hong, B. H.; He, J. L.; Fan, C. C.; Tang, C.; Le, Q. Q.; Bai, K. K.; Niu, S. W.; Xiao, M. T., Synthesis and Biological Evaluation of Analogues of Butyrolactone I as PTP1B Inhibitors. *Mar. Drugs* **2020,** *18*.
12. Gao, H. Q.; Guo, W. Q.; Wang, Q.; Zhang, L. Q.; Zhu, M. L.; Zhu, T. J.; Gu, Q. Q.; Wang, W.; Li, D. H., Aspulvinones from a mangrove rhizosphere soil-derived fungus Aspergillus terreus Gwq-48 with anti-influenza A viral (H1N1) activity. *Bioorg. Med. Chem. Lett.* **2013,** *23*, 1776-1778.
13. Zhang, L. H.; Feng, B. M.; Zhao, Y. Q.; Sun, Y.; Liu, B.; Liu, F.; Chen, G.; Bai, J.; Hua, H. M.; Wang, H. F.; Pei, Y. H., Polyketide butenolide, diphenyl ether, and benzophenone derivatives from the fungus Aspergillus flavipes PJ03-11. *Bioorg. Med. Chem. Lett.* **2016,** *26*, 346-350.
14. Pang, X.; Zhao, J. Y.; Fang, X. M.; Zhang, T.; Zhang, D. W.; Liu, H. Y.; Su, J.; Cen, S.; Yu, L. Y., Metabolites from the Plant Endophytic Fungus Aspergillus sp CPCC 400735 and Their Anti-HIV Activities. *J. Nat. Prod.* **2017,** *80*, 2595-2601.
15. Nobutoshi Ojima, S. T., Shuichi Seto, Structures of pulvinone derivatives from Aspergillus

terreus. *Phytochemistry* **1975,** *14*, 573-576.
16. Manchoju, A.; Annadate, R. A.; Desquien, L.; Pansare, S. V., Functionalization of diazotetronic acid and application in a stereoselective modular synthesis of pulvinone, aspulvinones A-E, G, Q and their analogues. *Org. Biomol. Chem.* **2018,** *16*, 6224-6238.
17. Xue, X.; Yang, H.; Shen, W.; Zhao, Q.; Li, J.; Yang, K.; Chen, C.; Jin, Y.; Bartlam, M.; Rao, Z., Production of authentic SARS-CoV M(pro) with enhanced activity: application as a novel tag-cleavage endopeptidase for protein overproduction. *J. Mol. biol.* **2007,** *366*, 965-75.
